# Design Rules for Controlling Connectivity, Topology, and Sorting Using Hydrogen‐Bonded Pairs and Aromatic Components in Palladium(II)‐Based Interlocked and Foldameric Systems

**DOI:** 10.1002/anie.5763518

**Published:** 2026-04-10

**Authors:** Jess L. Algar, Jordan N. Smith, Dan Preston

**Affiliations:** ^1^ Research School of Chemistry Australian National University Canberra ACT 2601 Australia

**Keywords:** interlocked, metallo‐supramolecular, palladium(II), topology, π─π interactions

## Abstract

Nature's exquisite control over structure arises from its use of complex arrays of topologically complicated species capable of forming and functioning orthogonally. Chemists seek the same level of control in synthetic systems, and here we report the design rules for self‐assembly of systems integrating metal‐coordination to Pd(II), complementary geometries, interligand hydrogen bonding, and π─π interactions. This has been achieved by integrating hydrogen‐bonded complementary pairs, **AA:DD** and **DA:AD** (**A** = acceptor, **D** = donor), and complementary aromatic regions of high and low electron density into a series of ligands which controllably self‐assemble into defined three‐dimensional structures with Pd(II). These rules have allowed us to elucidate pathways to multiple molecular types with defined connectivity and topology, including metallo‐foldamers, cyclic species, and an interlocked clippane formed under thermodynamic control. We have also established that, despite similarities between their respective components, these diverse structures persist in a combinatorial mixture.

## Graphical abstract

We have established a set of design rules which combine Pd(II) metal coordination, complementary geometries, complementary interligand hydrogen bonding and π─π interactions for predictable self‐assembly of Pd(II)‐based species such as metallo‐foldamers, cyclic species a metallo‐interlocked clippane. The species persist in a combinatorial mixture due to heteromeric sorting of their constituent ligands.

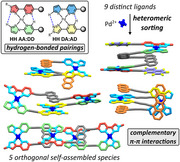

## 1 Introduction

The folded structure of proteins originates from the primary sequence of residues within it. Different amino acids have different side chains predisposed to direct the formation of alpha‐helices, beta‐pleated sheets or turns into a peptide depending upon the supramolecular interactions that can be established [1]. The resulting persistent topology directly confers function. For example, the substrate selectivity of enzymes stems from the specific three‐dimensional structure their active sites adopt [2, 3]. The beauty of this system is that nature accomplishes this with a set of simple unified building blocks (amino acids) that are highly similar in basic structure but in combination give rise to incredible diversity. Critical to this behavior is coexistence in solution of multiple different molecules simultaneously.

Supramolecular chemists want to replicate this behavior in artificial, self‐assembled systems, and have explored multiple avenues towards this goal. For example, strategies to control the conformation of flexible systems such as foldamers [4, 5] have been investigated, producing many beautiful molecules [6, 7]. A range of techniques have proved useful including hydrogen bonding [8, 9, 10], metal ion coordination [11, 12, 13], π─π interactions [14, 15, 16, 17] or a combination thereof [18, 19, 20, 21]. Interlocked architectures represent another route to higher topological complexity. Many mechanically interlocked molecules have been explored previously [22, 23, 24, 25], but of particular relevance to our report here is the formation of a [2]clippane by the Peris group [26], extending from the synthesis of U‐shaped molecular tweezers [27, 28]. Self‐recognition driven by π─π interactions accompanied by significant steric bulk across cofacial pyrene‐imidazolidene moieties linked by a bis‐alkynyl pentacyclic group results in the interlocking of two tweezer units, to produce what they term a [2]clippane. They were able to establish that this product was kinetically locked after formation. In this respect the structure has some similarities to organic foldamers, which maintain their secondary structure under thermodynamic control, but have their primary sequence created through stepwise, irreversible bond formation.

Another approach to access complex, artificial systems is to exploit the reversibility of metal‐ligand coordination bonds to create self‐assembled alternatives. Efficient mechanisms to controllably combine two or more components together at a metal centre are essential to do so. Control can be gained via the combination of ligands with complimentary geometries; however, this technique has previously generally been limited to the generation of discrete, cyclic structures with limited degrees of freedom [29, 30, 31, 32, 33, 34, 35, 36, 37, 38, 39]. More generally, complimentary denticities of ligands and/or the use of different metal ions can enable control over the coordination sphere [40, 41, 42, 43, 44, 45, 46, 47]. Additional control can be gained through modification of the coordination sphere, such as the addition of steric bulk and/or hydrogen bonding groups [31, 48, 49, 50, 51, 52].

Effective control over the self‐assembly of these species additionally presents the opportunity to generate dynamic combinatorial libraries, reminiscent of the coexisting mixtures of molecules found in biology. The ability to orthogonally maintain mixtures of species has been exemplified well previously by groups such as Chand [53], Clever [54, 55], Lehn [56, 57, 58], and others [59, 60]. The metallo‐supramolecular examples from Lehn cited above exploit the different denticity of ligands combined with different coordination preferences of different metal ions to ensure fidelity of sorting. Notably, the examples from Chand and Clever successfully incorporate the same ligand into different heteroleptic structures in a single solution, all formed with Pd(II). Examples of this second sort rely thus far on ligands with low degrees of freedom forming lantern‐shaped Pd_2_L_4_ cages.

The techniques for controlling identity and connectivity in rigid systems have been highly effective, but there is still work to do to establish strategies for control in topologically variable systems. It is worth asking what characteristics are worthy of pursuit in aiming for this goal. Taking inspiration from amino acids, which dictate the primary and secondary structure of proteins, we believe the building blocks should: 1) share similar characteristics and be modular, 2) be easily modified to create structural diversity, 3) be capable of forming a defined sequence through self‐assembly, 4) their combination should direct connectivity and identity, as well as persistent topology and conformation, and 5) they should be capable of forming species orthogonally to others in the same solution. The building blocks would thereby be analogous to amino acids, but unlike the primary sequence generated in proteins and peptides, form through self‐assembly.

**FIGURE 1:**
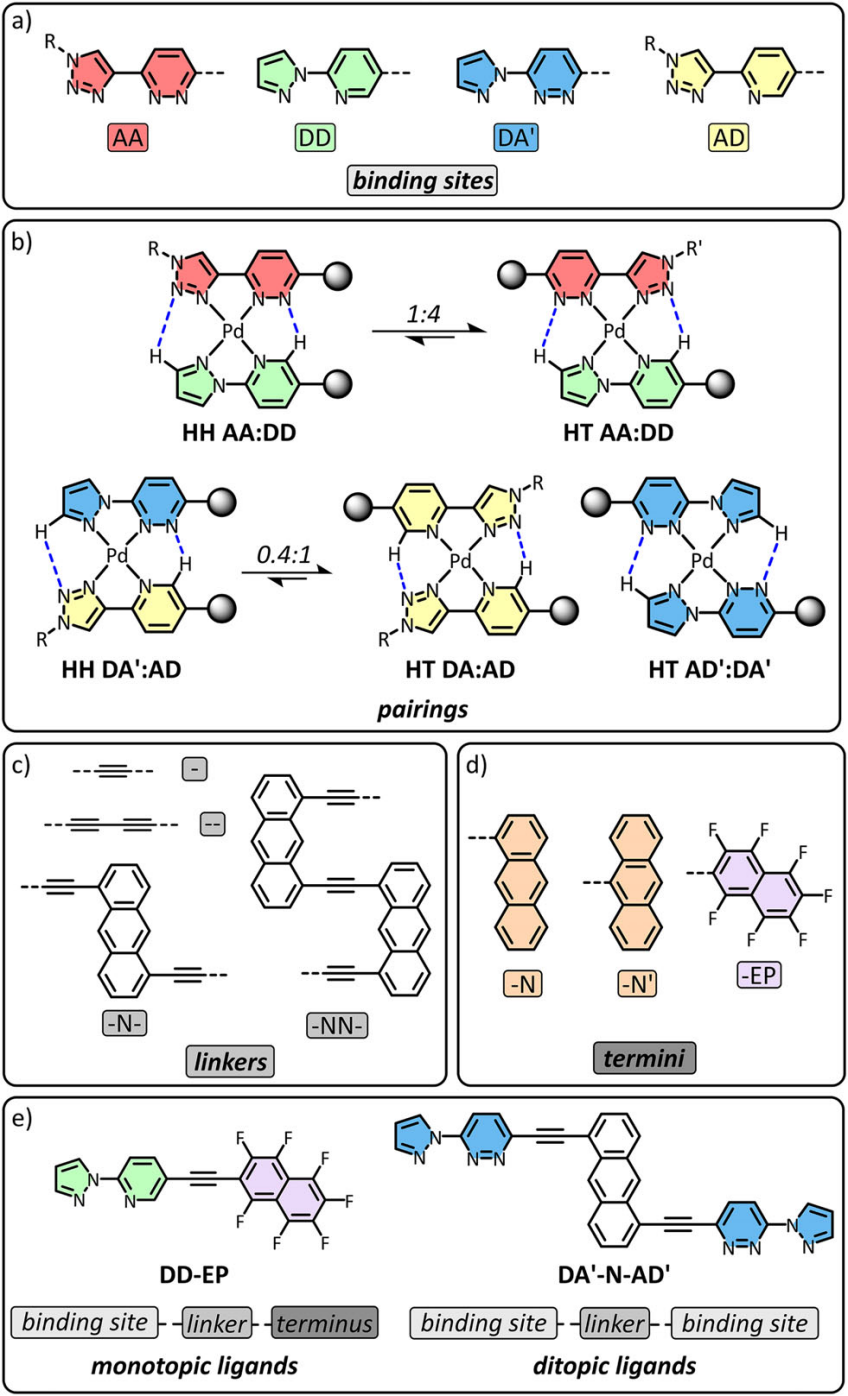
Chemical structures and cartoon representations of (a) binding sites, and the (b) pairings they can produce with equilibria included detailing their relative favorability, (c) linkers, and (d) termini of ligands, which make up either (e) monotopic or ditopic ligands exemplified by chemical structures of **DD‐EP** (*left*) and **DA′‐N‐AD′** (*right*). Hydrogen bonds are depicted in pairings through blue dotted lines.

With this in mind, we set ourselves the goal of establishing a robust set of design rules for the control over identity, connectivity, and secondary structure. Inspired by the complementary hydrogen‐bonding of deoxyribose nucleic acid (DNA) base‐pairs, we have previously reported [61, 62, 63] a set of bidentate coordination sites which are flanked by varied hydrogen bond donor (**D**) and hydrogen bond acceptor (**A**) sites, for example a pyridyl‐triazole motif (**‐DA**), a pyridyl‐pyrazole motif (**‐DD**), a pyridazinyl‐triazole (**‐AA**), and pyridazinyl‐pyrazole (**‐AD'**) (Figure 1a). When incorporated into ligands and combined with square planar Pd(II) which holds hydrogen‐bonding sites in a single plane, the connectivity can be predicted and potentially controlled by considering the hydrogen bonding complementarity between binding sites involved, allowing the formation of higher order structures [51, 64].

In terms of conformational control, aromatic π─π interactions are particularly favorably between regions of opposite electronic character [20, 62, 65, 66, 67, 68], i.e. electron rich and poor. We have previously used this strategy in combination with our pairings and Pd(II) to form self‐assembled foldamers [69]. Unlike most metallo‐foldamers, which use preformed organic sequences, the metal ions were integrated directly into the primary sequence [18, 70], and differed from metallo‐helicates [71, 72] in that they were single‐stranded, again due to integration of the metal ion into the sequence. While we achieved proof‐of‐concept, our previous work was limited in scope, complexity, molecular type, and in the provision of a set of guidelines for general applicability.

We report here a series of building blocks combining these elements and a set of robust design rules for control over identity, position, conformation and connectivity that can be applied to generate a diverse family of heteroleptic, sequence‐specific structures under thermodynamic control. Examples include the first [2]clippanes under thermodynamic control, non‐cyclic foldamers, and macrocyclic structures with integrated topological control. Using ligands with hydrogen‐bonding capability of varied topicity at the chelating sites and linkers or termini of different shape, length and aromatic character, we have carried out varied complexations with Pd(II). Often in the current literature, there is a focus on only reporting positive results. We purposefully include here both what works and what does not, allowing us to elucidate the design rules of the system. We also demonstrate that combinations of these structures can coexist in solution, with heteromeric sorting shown by the sharing of one ligand between two different heteroleptic species.

## 2 Results and Discussion

### 2.1 Ligand Design and Nomenclature

Previously, we [62] have established that homoleptic combinations of **AA‐** and **DD‐** based ligands produce a mixture of PdL and HT and HH PdL_2_ type complexes as the symmetrical nature of the hydrogen bond donors or acceptors in each pairing results in a loss of isomeric selectivity. By the same reasoning, heteroleptic combinations of **AA‐** and **DD‐** based ligands with Pd(II) in a 1:1:1 ratio exclusively produces both HH and HT heteroleptic complexes in a 1:4 ratio respectively, but no homoleptic complexes, as hydrogen bonding is only maximized in either heteroleptic combination (Figure 1b). The homoleptic combination of **AD‐** or **DA'‐** based ligands in a 2:1 ratio with Pd(II) produces PdL_2_ type complexes with a head to tail (HT) conformation to maximize hydrogen bonding [73, 74, 75]. However, we have shown [62, 63] that the heteroleptic combination of **AD‐** and **DA'‐** based ligands with Pd(II) in a 1:1:1 ratio produces a mixture of head‐to‐head (HH) heteroleptic and HT homoleptic complexes, as hydrogen bonding is maximized in all three of these complexes (Figure 1b). The equilibria however, may be manipulated depending on the larger secondary structure the pairings may be part of.

Expanding upon these complementary pairings, a series of 21 ligands have been designed, synthesized and characterized. In keeping with our goals, they are bound by certain similarities but also have controlled diversity. The ligands were either 1) monotopic: consisting of a single pairing, linker and terminus or, 2) ditopic: consisting of two pairings joined by a single linker. Monotopic ligands act as a ‘capping’ ligand used in the formation of non‐cyclic complexes whereas ditopic ligands either extend noncyclic species or form part of cyclic species.

The ligands each have linkers between either two binding sites or a binding site and a terminus. Linker identity is either 1) a monoalkyne (denoted by a single dash, ‐) giving a short ligand with pairings/termini aligned, 2) a dialkyne (–) giving a slightly longer ligand also with pairings/termini aligned, 3) a *mon*o‐anthracene unit (**‐N‐**) giving a longer, ‘staggered’ ligand in which the pairings/termini are offset by the length of the anthracene group, or 4) a *bis*‐anthracene unit (**‐NN‐**) gives a longer, ‘looped’ ligand providing a single full helical turn (Figure 1c). Importantly, **‐N‐** and **‐NN‐** incorporate aromatic panels with high electron density, that may recognize electron deficient regions.

In monotopic ligands, aromatic moieties are instead incorporated at the termini existing as an electron‐rich 1‐ or 9‐ substituted anthracene group (**‐N** and **‐N'** respectively) or an electron‐poor perfluorinated naphthalene group (**‐EP**) (Figure 1d). The ligands are named accordingly through combination of the abbreviations for pairing(s), linker and terminus (if applicable) in their respective order. For example, a monotopic ligand featuring a **DD** binding site, alkyne spacer (‐) and electron‐poor perfluorinated naphthalene terminus (**‐EP**) is named **DD‐EP**, whereas a ditopic ligand featuring **DA'**‐ binding sites linked by a *mono*‐anthracene spacer (**‐N‐**) is named **DA'‐N‐AD'** (Figure 1e). Note that in this terminology, acceptor (**A**) and donor (**D**) sites are listed with *directionality*, consistent with connection to the linker. As the latter example has the hydrogen‐bond acceptor (the pyridazine) connected to the linker, the A is denoted proximal to the linker (**‐N‐**) on both the right and left. For simplicity, the pyrazolyl‐pyridazine moiety will additionally be indicated by a prime symbol (i.e. as **‐AD'** or **DA'‐**). Complexes have been denoted as the sequence of ligands present separated by ‘•’ to represent the Pd(II) centres. Solubilising chains have been omitted for clarity, for **AD**, R = ‐(CH_2_CH_2_O)_2_OCH_3_, for **AA**, R = CH_2_CH(OCH_3_)_2_, and for **AA**', R = CH_2_CH_2_OCH_3_ (see Supporting Information). The different solubilising chains were incorporated for their usefulness in 2D NOESY NMR analysis and characterisation. Some ligands (**AD‐DA, DD‐DD, AD‐AA, DD‐EP**, and **AA‐EP**) were previously synthesised by us [62, 63], the other 15 are novel. Characterisation data for all ligands is found in the Supporting Information (S1.3–1.6).

### 2.2 Complex Formation and Characterization

Complexes were formed through combination of ligands and [Pd(CH_3_CN)_4_](BF_4_)_2_ in [D_6_]DMSO. Results were analyzed in situ via nuclear magnetic resonance (NMR) techniques, with complexes forming at room temperature either in the time taken to analyse via ^1^H NMR or overnight. ^1^H DOSY NMR was used to confirm a single species was produced in each case with HR ESI‐MS confirming the identity of each species. Significant downfield shifts of select proton environments were used as an indication of metal coordination and hydrogen bonding, while significant upfield shifts indicated the potential for π─π interactions. Analysis of through‐space NOESY correlations has enabled an understanding of the isomerism and three‐dimensional conformation adopted by each species. Two X‐ray crystal structures were obtained, and all complexes were computationally modelled using GFN2‐xtb [76]. Full characterization data are detailed in the (S1.7–1.9).

### 2.3 Complexes from Monotopic Ligands

**FIGURE 2:**
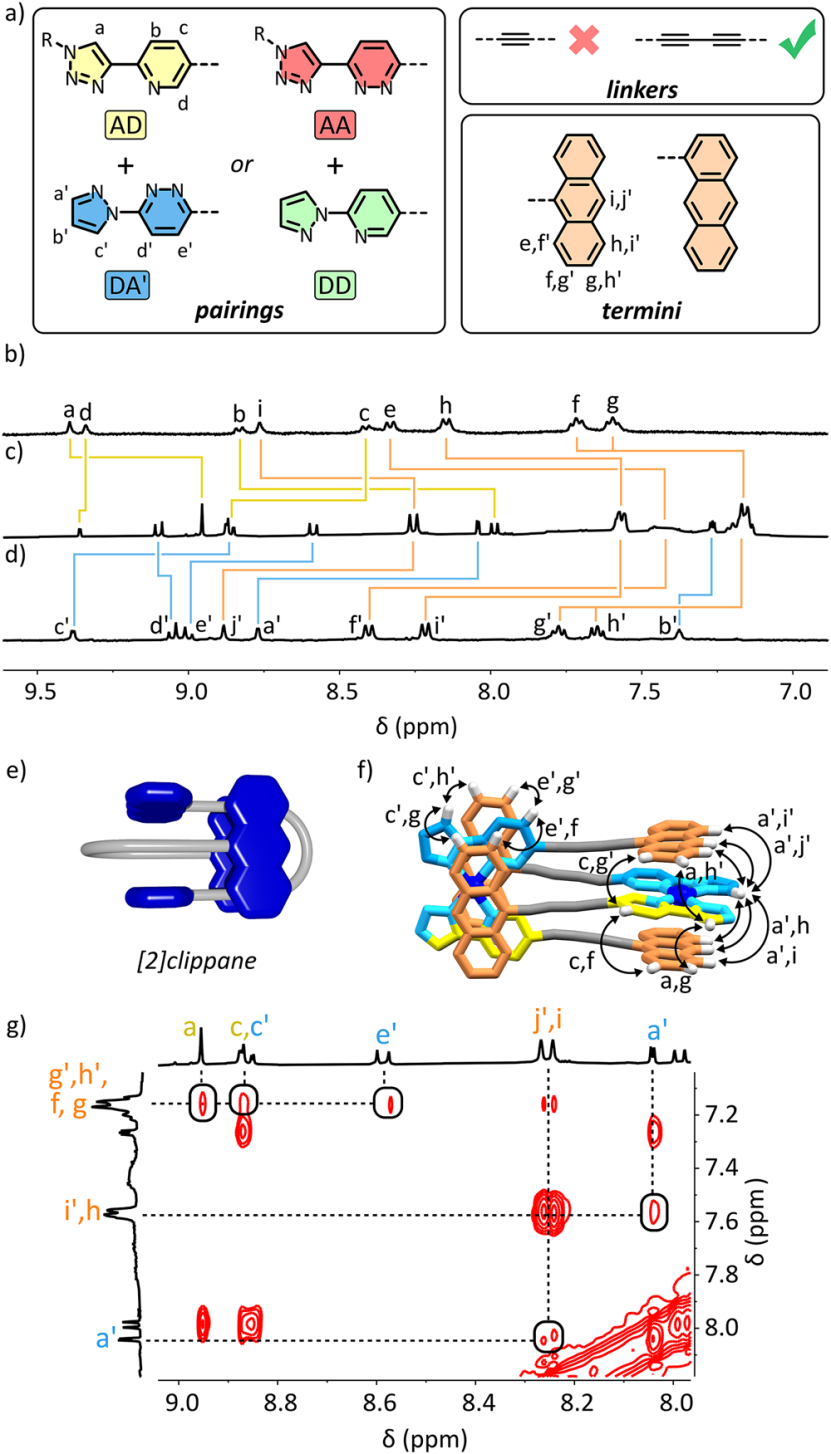
a) Chemical structures of the pairings, linkers and termini used in ligands designed to make [2]clippane species. Partial ^1^H NMR spectra ([D_6_]DMSO, 400 MHz, 298 K) of (b) [Pd(**AD–N'**)_2_](BF_4_)_2_, (c) [[Pd(**DA'–N'**)(**AD–N'**)]_2_](BF_4_)_4_, and (d) [Pd(**DA'–N'**)_2_](BF_4_)_2_. A (e) cartoon representation of a generalised [2]clippane is presented with the (f) x‐ray crystal structure of [[Pd(**DA'–N'**)(**AD–N'**)]_2_](BF_4_)_4_ highlighting through‐space NOE correlations observed in (g) a partial ^1^H NOESY NMR spectrum (400 MHz, [D_6_]DMSO, 298 K, 400 ms) of [[Pd(**DA'–N'**)(**AD–N'**)]_2_](BF_4_)_4_.

In some of our previous work [62], the addition of electron rich aromatic groups beyond the coordination sphere was found to not affect the isomerism or speciation in each case. However, we found that π─π interactions between electron rich aromatic groups and electron poor cationic panels where Pd(II) is bound, are enthalpically favorable [69, 77]. Based on this, we predicted that ‘paired’ monotopic ligands of a pairing linked to an electron‐rich 9‐substituted anthracene terminus (**‐N'**) could produce HH heteroleptic complexes able to interlock and effectively ‘sandwich’ coordination sites between anthracene groups. If accessible, the combination of the established pairings enthalpic favorability of the ‘cation’‐π interactions would drive the formation of the dimeric species over any HT heteroleptic or homoleptic complexes. We speculated that the steric bulk of the anthracene groups would create a mechanical bond between the two PdL_2_ complexes, creating a pseudo‐interlocked ‘[2]clippane’ (Figure 2e).

We investigated the effect of linker length in the formation of the [2]clippanes, synthesising eight monotopic ligands, combining a single binding site (**AD**, **DA'**, **AA** or **DD**) with a 9‐substituted anthracene terminus (**‐N'**) by one of two possible linkers, a monoalkyne (‐) or dialkyne (–) (Figure 2a). The ligands were combined in four sets of complimentary pairings, **AD:DA'** and **AA:DD** for each linker length.

The combination of the shorter‐linked **AD‐N'**, **DA'‐N'** and Pd(II) in a 1:1:1 ratio produced a complex mixture of species in the ^1^H NMR spectrum (S2.1.1). In pleasing contrast, the 1:1:1 combination of the longer‐linked **AD–N'** and **DA'–N'** with Pd(II) produced a ^1^H NMR spectrum containing a single set of peaks immediately (Figure 2c). ^1^H DOSY NMR spectroscopy confirmed only a single species was present (*D* = 0.86 × 10^−10^ m^2^ s^−1^). Relative to the homoleptic HT Pd(**AD–N'**)_2_ species (Figure 2b), the significant downfield shift of H_d_ was maintained at 9.36 ppm from 9.34 ppm (Figure 2c), indicating H_d_ continues to act as a hydrogen bond donor. This indicates that the HH isomer of a single heteroleptic species persists. Relative to the respective homoleptic complexes, [Pd(**AD–N'**)_2_](BF_4_)_2_ and [Pd(**DA'–N'**)_2_](BF_4_)_2_ (Figure 2b and d), significant upfield shifts of proton environments of the anthracenyl termini suggest the presence of π─π interactions. Critically, through‐space NOE correlations between proton environments of the aromatic terminus (H_f_, H_g_, H_h_, H_g'_, H_h_, and H_i'_) and those of the pairing, (H_a_, H_c_, H_a'_, H_c'_, and H_e'_) of the ligand demonstrate spatial proximity and suggest that this species exists as the intended interlocked [2]clippane (Figure 2g). Further evidence of this was the observation of the dimeric species by Nanospray high‐resolution mass spectrometry (HRMS), *m/z* = 474.8598 [[Pd(**DA'–N'**)(**AD–N'**)]_2_]^4+^ (calc. for C_110_H_76_N_16_O_4_Pd_2_^4+^, 474.8580) and *m/z* = 948.7203 [[Pd(**DA'–N'**)(**AD–N'**)]**_2_** – 2H^+^]^2+^ (calc. for C_110_H_74_N_16_O_4_Pd_2_^2+^, 948.7088) [78]. Similar data was obtained for the [[Pd(**AA'–N'**)(**DD–N'**)]_2_](BF_4_)_4_ analogue confirming the formation of a second [2]clippane, irrespective of pairing.

Surprisingly, an analogous [2]clippane, [[Pd(**DA'–N**)(**AD–N'**)]_2_](BF_4_)_4_ can even form when combining ligands with different termini: **AD–N'** and **DA'–N**. While the anthracenyl moiety of **DA'–N** could exist in two different orientations, we only observe a single isomer which based on the through‐space NOE correlations, prefers to *π*—stack over the more electron‐deficient pyridyl ring of **AD–N'**, consistent with our other findings.

Finally, crystals [79] of [[Pd(**DA'–N'**)(**ADN'**)]_2_]^4+^ were grown through diffusion of diethyl ether into a CH_3_CN/DMSO solution of the complex. Single crystal X‐ray diffraction (SCXRD; I $\bar{4}$2d, Figure 2f) reveals an interlocked species like that predicted. Due to disorder by symmetry in the structure, the formation of homoleptic versus heteroleptic complexes in the solid‐state is ambiguous. Nevertheless, the crystal structure clearly demonstrates interlocking into a [2]clippane, and in combination with NMR data gives proof‐of‐structure.

The combination of the [[Pd(**AA'–N'**)(**DD–N'**)]_2_](BF_4_)_4_ [2]clippane with **AD–N'** and **DA'–N'** in a 1:2:2 ratio resulted in a mixture of products. In competition for Pd(II), there is liberation of some **DD–N'** and **AA'–N'** and formation of [Pd(**AD–N'**)(**DA'–N'**)]_2_(BF_4_)_4_ and scrambled clippanes. This indicates these [2]clippanes are not kinetically locked as such but rather are the first examples of [2]clippanes formed under thermodynamic control (S3.1.1). Variable temperature NMR up to 100°C showed no evidence of dissociation into monomeric species, indicating the high enthalpic favorability of the interlocked species.

We have demonstrated that cation‐π interactions can overcome the entropic cost of interlocking. Linker length is crucial in this case potentially for multiple reasons: a longer linker could 1) reduce the steric crowding of the interlocked species; 2) be flexible enough to reduce the entropic cost of interlocking; and 3) increase the distance between similarly positively charged Pd(II) centres.

### 2.4 Complexes from the Combination of Monotopic Ligands and Ditopic Ligands

**FIGURE 3:**
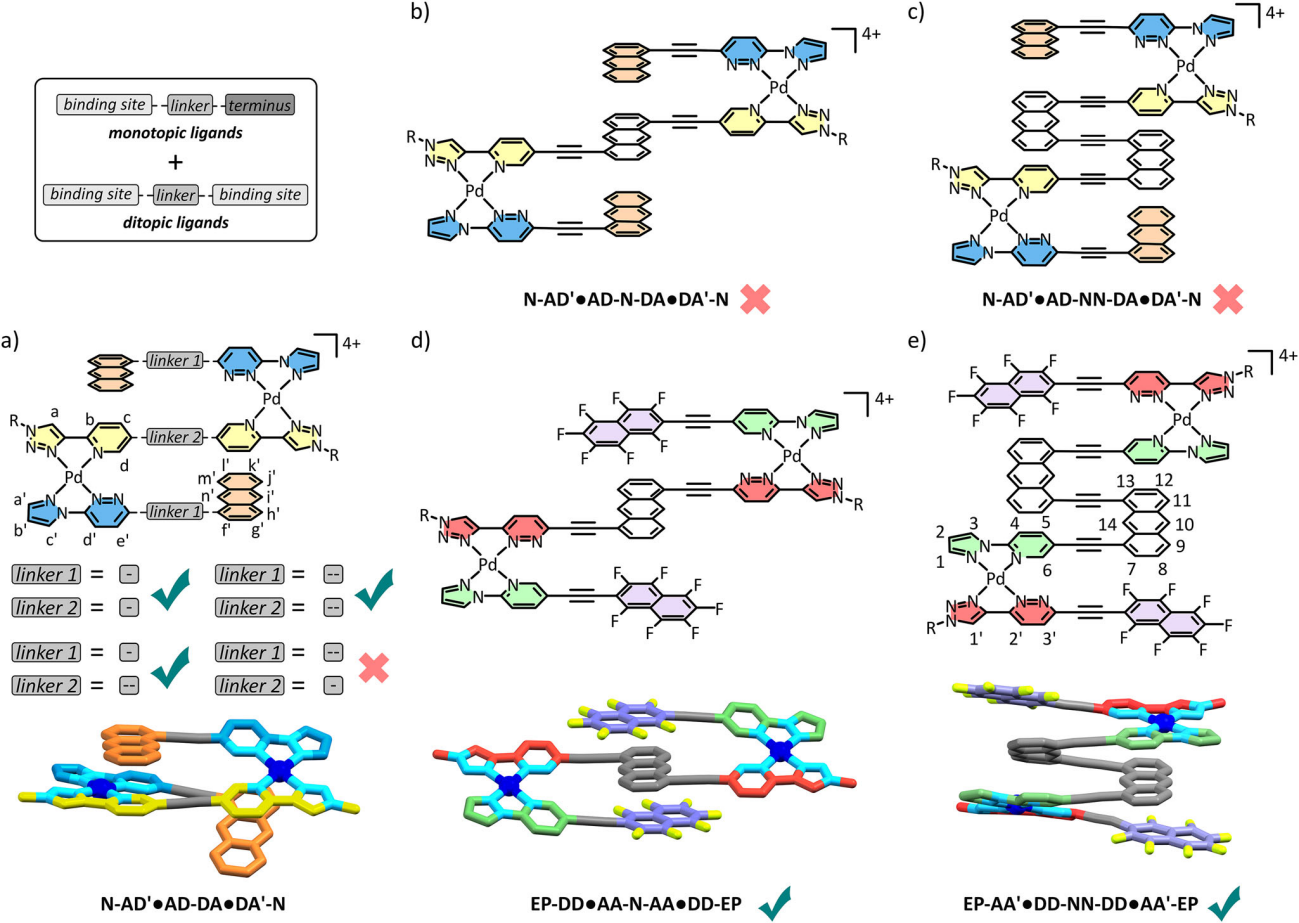
Proposed chemical structures of (a) **N‐AD'•AD‐N‐DA•DA'‐N** and (b) **N‐AD'•AD‐NN‐DA•DA'‐N** which do not self‐assemble cleanly, compared to chemical structures and structural representations of (c) **N‐AD'•AD‐DA•DA'‐N, N–AD'•AD–DA•DA'–N** and **N‐AD'•AD–DA•DA'‐N (d) EP‐DD•AA‐N‐AA•DD‐EP** and (e) **EP‐AA'•DD‐NN‐DD•AA'‐EP** (x‐ray).

We next hypothesized that a similar approach could be extended to produce noncyclic foldamers from a combination of monotopic and ditopic ligands (Figure 3). Monotopic ligands were designed to incorporate a pairing, alkynyl linker (‐ or –) and aromatic terminus of varied electronic character (**‐N** or **‐EP**; Figure 1). Unlike the ligands in the clippane, the anthracene unit used was substituted at the 1‐position, as preliminary investigations suggested that this was required for maximisation of π─π interactions. Ditopic ligands consisted of two of the same type of pairing linked by a variety of linkers, both alkynyl (‐ or –) and aromatic (**‐N‐** or **‐NN‐**; Figure 1). Different combinations of ligands were combined to understand the characteristics required to controllably self‐assemble non‐cyclic foldamers.

**FIGURE 4:**
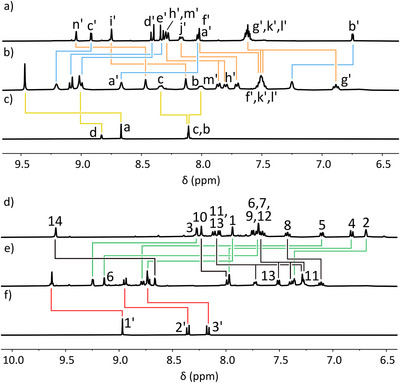
Partial ^1^H NMR spectra ([D_6_]DMSO, 400 MHz, 298 K) of (a) **DA'‐N**, (b) **N‐AD'•AD‐DA•DA'‐N**, (c) **AD‐DA** and (d) **DD‐NN‐DD**, (e) **EP‐AA'•DD‐NN‐DD•AA'‐EP** and (f) **AA'‐EP**. Proton environments are labelled on the chemical structures in Figure 3.

The combination of **DA'‐N**, **AD‐DA** and Pd(II) in a 2:1:2 ratio resulted in the formation of a single species demonstrated by a single set of peaks in the ^1^H NMR spectrum (Figure 4b) and confirmed by ^1^H DOSY NMR spectroscopy (*D* = 0.92 × 10^−10^ m^2^ s^−1^). A Pd_2_(**AD‐DA**)(**DA'‐N**)_2_ type species was observed by HR ESI‐MS *m/z* = 799.1472 [M + 2BF_4_^−^]^2+^ (calc. for C_72_H_58_N_16_O_4_Pd_2_B_2_F_8_^2+^, 799.1502). Relative to the free ligands (Figure 4a and c), the chemical shifts of the proton environments associated with the binding sites generally shift downfield (blue and yellow lines), confirming coordination of Pd(II), whereas chemical shifts of the aromatic termini shift upfield, suggesting the presence of π─π interactions (orange lines; Figure 4a–c). The heteroleptic **AD:DA** pairing can be expected to prefer a HH orientation, but this is also confirmed by a through‐space NOE correlation between H_a'_ and H_e_ protons on the different sites. As predicted, the favorable cation‐π interactions between anthracene termini and Pd(II) cationic panels enables the self‐assembly of the targeted heteroleptic species, **N‐AD'•AD‐DA•DA'‐N** (Figure 3c). The analogous, **N–AD'•AD–DA•DA'–N** species, differentiated by dialkynyl linkers can also be accessed in the same manner (Figure 3c). Interestingly, the combination of **DA'‐N** and **AD–DA** produced a single foldamer in solution yet the combination of **DA'–N** and **AD‐DA** gave a complex mixture of products.

Linker length is evidently important and although it can be altered, sufficient overlap between electron rich aromatic termini and the Pd(II) cationic panels must exist for successful self‐assembly of the expected foldamers. We note that the long central linker with short terminal linkers positions the anthracene terminus over the more electron deficient six‐membered rings, while the short central linker and long terminal linkers gives a position more over the more electron rich five‐membered rings. Similarly, altering either the active π‐surface, combining **AD‐DA** with **DA'‐N'**, or the identity of the π‐surface, combining **DD‐DD** with **AA'‐EP**, does not give clean formation of analogous foldamers, demonstrating the importance of maximizing π─π interactions to access these structures. Depending on the system, orientational control at the pairing may be lost or homoleptic complexes can preferentially form (S2.1.2 and 2.1.3).

If instead the linker of the ditopic ligand (**AD‐DA**/**AD–DA**) is replaced by an aromatic, offset linker, **‐N‐** or **‐NN‐**, the analogous noncyclic foldamers no longer form cleanly (Figure 3a and b). The spectra obtained were broad, with multiple peaks, suggesting complex equilibrium mixtures. The difference in this case is the potential structures now have the direct overlay of electron‐rich anthracene units. We propose that either the system does not allow for the electron‐rich aromatic groups to stack offset to one another, and/or this type of π─π interaction is simply not strong enough to form a single species. To explore the requirements of the ‘aromatic matching’ further, monotopic ligands with electron poor, perfluorinated naphthalene termini (**‐EP**) were also assessed. DD/AA pairings were used in this case (**DD‐EP** and **AA'‐EP**) [62] and so combined with the corresponding AA/DD ditopic, **‐N‐** or **‐NN‐** linked ligands (**AA‐N‐AA** or **DD‐NN‐DD**) and Pd(II) in a 2:1:2 ratio as before (Figure 3d and e). Similar results were obtained for both combinations so for simplicity, only **EP‐AA'•DD‐NN‐DD•AA'‐EP** will be discussed here (see S1.8.4). A single set of peaks in the ^1^H NMR spectrum (Figure 4e), and ^1^H DOSY NMR spectroscopy (*D* = 0.90 × 10^−10^ m^2^ s^−1^) confirmed a single heteroleptic species was formed (see S1.8.5). The expected heteroleptic complex was additionally identified by HR ESI‐MS, *m/z* = 472.0539 [M]^4+^ (calc. for C_92_H_48_N_16_O_2_F_14_Pd_2_^4+^, 472.0509), *m/z* = 658.4019 [M + BF_4_^−^]^3+^ (calc. for C_92_H_48_N_16_O_2_BF_18_Pd_2_^3+^, 658.4023), *m/z* = 1031.1014 [M + 2BF_4_^−^]^2+^ (calc. for C_92_H_48_N_16_O_2_B_2_F_22_Pd_2_^2+^, 1031.1053). Again, relative to the free ligands (Figure 4d and f), the chemical shifts of the proton environments associated with the binding sites shift downfield (green and red lines), confirming coordination of Pd(II), whereas chemical shifts of the aromatic linker shift upfield, suggesting additional π─π interactions (black lines; Figure 4d–f). As discussed previously, hydrogen bonding across the **AA:DD** pairing is maximized in both the HH or HT isomer, but through‐space NOE correlations between H_1_ and H_4'_ and H_1_ and H_5'_ indicate the HH isomer is formed.

Excitingly, crystals of **EP‐AA'•DD‐NN‐DD•AA'‐EP** were grown through diffusion of THF into a toluene/DMSO solution of the complex, confirming the predicted folded structure through SCXRD (P $\bar{1}$, Figure 3e). The folded arrangement suggests π─π interactions exist between the electron‐rich anthracene linkers and the electron‐poor termini or Pd(II) cationic panels. We conclude that in these systems, favorable π─π interactions can effectively control sequence identity, connective orientation and conformation in noncyclic foldamers, as long as they exist between electron‐rich and electron‐poor aromatic groups.

### 2.5 Complexes from Staggered, Ditopic Ligands

The combination of ditopic ligands with Pd(II) generally encourages the formation of the smallest possible cyclic structure that may form without undue strain. We have shown previously that manipulating the complimentary pairings can affect the energetic landscape of self‐assembly to give rise to mismatched or polymeric species [63]. Here we aimed to instead probe how altering enthalpic contributions beyond the coordination sphere such as geometric constraints and π─π interactions affects self‐assembly.

**FIGURE 5:**
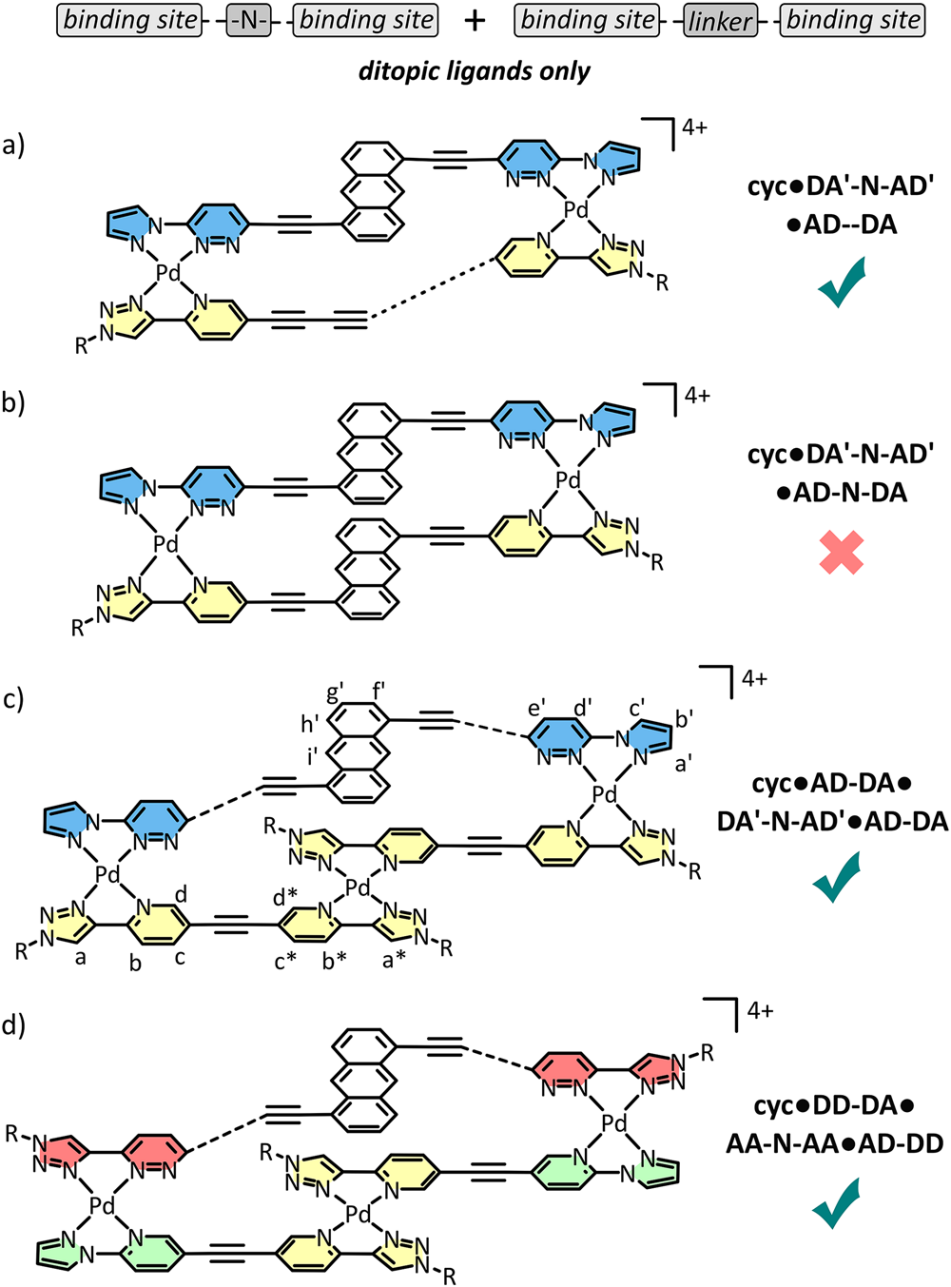
Chemical structures of (a) **cyc•DA'‐N‐AD'•AD–DA**, (b) **cyc•DA'‐N‐AD'•AD‐N‐DA** which only formed as 22% of the total species, (c) **cyc•AD‐DA•DA'‐N‐AD'•AD‐DA**, and (d) **cyc•DD‐DA•AA‐N‐AA•AD‐DD**.

The incorporation of a ‐**N**‐ linker to these ligands maintains their general linearity, but binding sites offset to one another might introduce significant strain in entropically favored 1+1 macrocycles. The simplest example of this is in the combination of **DA'‐N‐AD' with AD–DA** and Pd(II) in a 1:1:2 ratio (Figure 5a). In this case, a single set of peaks were observed in the ^1^H NMR spectrum, and ^1^H DOSY NMR spectroscopy confirmed the presence of a single species (*D* = 0.91 × 10^−10^ m^2^ s^−1^). Despite the significant amount of strain in this complex, the entropic product preferentially forms over homoleptic species or larger cyclic foldamers, as supported by HR ESI‐MS identifying the 1+1 ***cyc*•AD–DA•DA'‐N‐AD'**, *m/z* = 423.0875 [M—H^+^]^3+^ (calc. for C_60_H_47_N_16_O_4_Pd_2_, 423.0688).

The combination of two paired, staggered ligands, such as **DA'‐N‐AD'** and **AD‐N‐DA** could generate an analogous 1+1 macrocycle potentially with less strain due to the alignment of binding sites between ligands (Figure 5b). However, the electron rich anthracene groups present in both ligands also align, resulting in π─π stacking between the two electron‐rich units which is enthalpically disfavoured, as observed previously. As predicted, a 1:1:2 ratio of **DA'‐N‐AD'**, **AD‐N‐DA**, and Pd(II) gave a mixture of the 1+1 macrocycle as a minor component (22% of total species), among other (likely polymeric) species (see S3.1.2). We have previously demonstrated that the entropic product ‐a 1+1 macrocycle‐ can still form without a complimentary pairing at each Pd(II) metal ion, in other words in a mismatch [63]. To probe the importance of the pairings in this case where ancillary factors are affecting self‐assembly, other staggered ligands in combinations requiring mismatching (for example, the combination of **AD‐N‐DA** with **AA‐N‐AA**, S3.1.2) were analyzed via quantitative ^1^H NMR relative to an internal standard. The relative proportion of the 1+1 macrocycle decreased with increasing number of clashes between hydrogen atoms and the coordination sphere (19% ‐ 0%), with hydrogen‐hydrogen clashes paying a higher penalty than lone pair‐lone pair clashes. These data reiterate that complimentary hydrogen bonding is important at each pairing but must be combined with favorable ancillary interactions to reliably produce single heteroleptic complexes.

**FIGURE 6:**
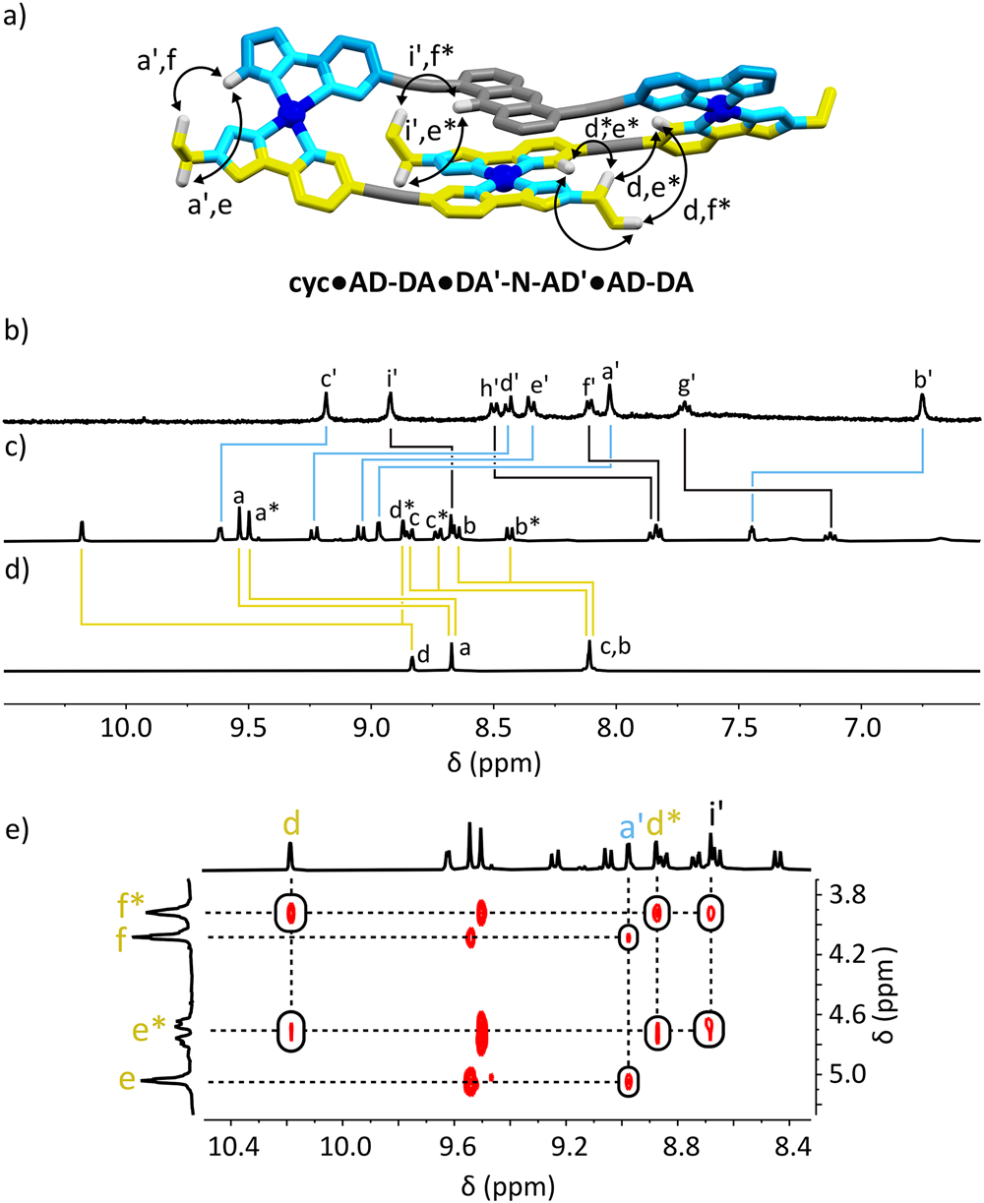
(a) model (GFN2‐xtb) of **cyc•AD‐DA•DA'‐N‐AD'•AD‐DA** depicting select proton environments for which through‐space NOE correlations exist, (b) partial ^1^H NMR spectra ([D_6_]DMSO, 400 MHz, 298 K) of b) **DA'‐N‐AD'**, (c) **cyc•AD‐DA•DA'‐N‐AD'•AD‐DA** and (d) **AD‐DA**, and (e) a partial ^1^H NOESY NMR spectrum (400 MHz, [D_6_]DMSO, 298 K, 200 ms) of **cyc•AD‐DA•DA'‐N‐AD'•AD‐DA**.

In comparison to the 1+1 ***cyc*•AD–DA•DA'‐N‐AD'** species, when the shorter **AD‐DA** ligand was combined with **DA'‐N‐AD'** and Pd(II) in a 1:1:1 ratio an untidy spectrum was obtained that only merged into a single species upon alteration of the ratio to 2:1:3 (Figure 6c). ^1^H DOSY NMR spectroscopy confirmed the peaks corresponded to a single species (*D* = 0.82 × 10^−10^ m^2^ s^−1^). The chemical shifts of proton environments associated with the binding sites of both **AD‐DA** (Figure 6d, yellow lines) and **DA'‐N‐AD'** ligands (Figure 6b, blue lines) shift downfield, indicating coordination of Pd(II). Most notably the number of chemical shifts associated with **AD‐DA** in the new species split into two environments of equal intensity, meaning the ligand was desymmetrized. Through space NOE correlations between H_a'_ and (H_e_ and H_f_) confirm a HH connection between each **AD‐DA** ligand and **DA'‐N‐AD'** about Pd(II) (Figure 6a). Further through space NOE correlations between H_d*_ and (H_e*_ and H_f*_) indicate the two **AD‐DA** ligands also coordinate about Pd(II) at the binding sites in the centre of the complex, in a homoleptic HT orientation, accounting for the observed desymmetrisation (Figure 6a). Upfield shifting of the chemical shifts associated with the anthracene linker of **DA'‐N‐AD'** (Figure 6b and c, black lines) combined with through‐space NOE correlations between H_i'_ and (H_e*_ and H_f*_) suggest π─π interactions between the anthracene group and the central ‘yellow’ **AD:DA** cationic panel in the complex. The **cyc•AD‐DA•DA'‐N‐AD'•AD‐DA** macrocyclic foldamer was additionally identified by HR ESI‐MS, *m/z* = 378.0719 [M + F^−^]^5+^ (calc. for C_84_H_78_N_24_O_8_Pd_3_F^5+^, 378.0682), *m/z* = 510.8406 [M + 2BF_4_^−^]^4+^ (calc. for C_84_H_78_N_24_O_8_Pd_3_B_2_F_8_^4+^, 510.8418), *m/z* = 710.4587 [M + 3BF_4_^−^]^3+^ (calc. for C_84_H_78_N_24_O_8_Pd_3_B_3_F_12_^3+^, 710.4598), *m/z* = 1109.1791 [M + 4BF_4_^−^]^2+^ (calc. for C_84_H_78_N_24_O_8_Pd_3_B_4_F_16_^2+^, 1109.1879).

The approach can be extended for use with other pairings, such as in the formation of **cyc•DD‐DA•AA‐N‐AA•AD‐DD** (Figure 5d), or even some mismatched variants (see S1.9.3 and 1.9.4), as long as the central homoleptic Pd(AD)(DA) configuration is maintained. This highlights that the favorability between electronically matched components can also be used to drive the preferred stoichiometry in the system.

### 2.6 1 + 1 complexes From Looped AD:DA Ligands

Although the anthracene spacer, ‐**N**‐, introduced additional complexity by offsetting the binding sites, it is relatively limited in the potential conformations it may adopt. With an extra rotatable bond, ligands with an **‐NN**‐ linker could exist in multiple conformations, meaning the offset between binding sites is variable. We were interested in assessing how the incorporation of the **‐NN‐** linker to give ‘looped’ ligands would affect the formation of potential cyclic foldameric macrocycles.

**FIGURE 7:**
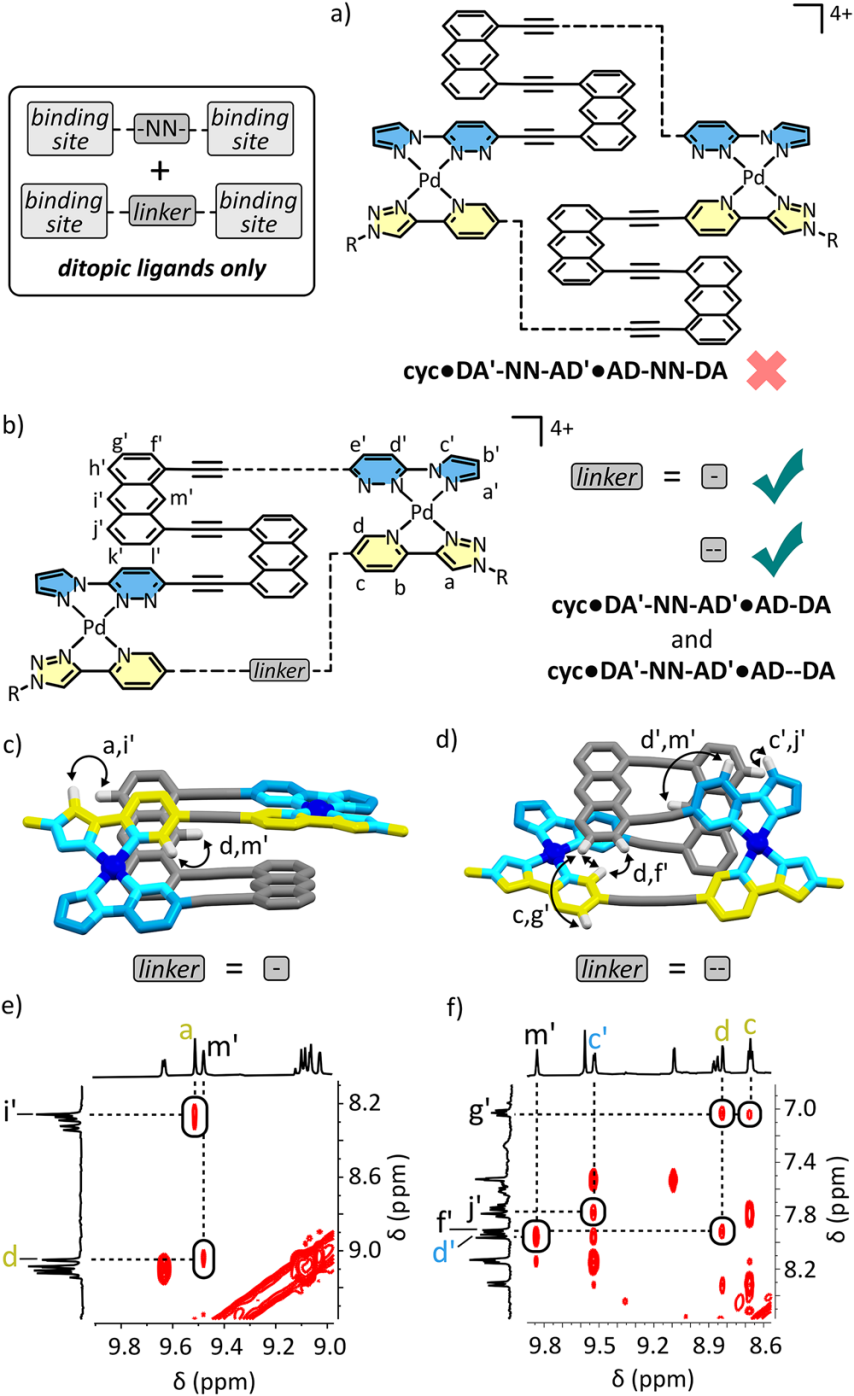
Chemical structures of the (a) proposed **cyc•DA'‐NN‐AD'•AD‐NN‐DA**, and (b) the observed **cyc•AD‐DA•DA'‐NN‐AD'** and **cyc•AD–DA•DA'‐NN‐AD'** species. Models of (c) **cyc•AD‐DA•DA'‐NN‐AD'** and d) **cyc•AD–DA•DA'‐NN‐AD'** are also shown highlighting through‐space NOE correlations observed in (e) a partial ^1^H NOESY NMR spectrum (400 MHz, [D_6_]DMSO, 298 K, 200 ms) of **cyc•AD‐DA•DA'‐NN‐AD'** and (f) a partial ^1^H NOESY NMR spectrum (400 MHz, [D_6_]DMSO, 298 K, 200 ms) of **cyc•AD–DA•DA'‐NN‐AD'**.

The simple combination of **‐NN‐** type ligands, for example, **DA'‐NN‐AD'** and **AD‐NN‐DA** with Pd(II) in a 1:1:2 ratio respectively, gave a complicated mixture of products. Based on the broad nature of peaks in the ^1^H NMR and the ESI‐MS data it is likely that a mixture of polymeric species form preferentially (S2.1.8). These observations are in line with previous findings given the smallest cyclic structure that could be expected from this combination, **cyc•DA'‐NN‐AD'•AD‐NN‐DA**, relies on overlap between electron‐rich anthracene panels (Figure 7a).

In contrast, the combination of **DA'‐NN‐AD'** with ligands that lack this aromatic linker, **AD‐DA or AD–DA** and Pd(II), again in a 1:1:2 ratio respectively, each produced a single set of peaks in their respective ^1^H NMR spectra and a single species, as confirmed by ^1^H DOSY NMR spectroscopy (*D* = 0.91 × 10^−10^ m^2^ s^−1^, *D* = 0.85 × 10^−10^ m^2^ s^−1^). HR ESI‐MS indicated the expected Pd_2_LL' type macrocycles (Figure 7b) were produced in each case, with *m/z* = 361.5722 [**cyc•AD‐DA•DA'‐NN‐AD'**]^4+^ (calc. for C_74_H_56_N_16_O_4_Pd_2_^4+^, 361.5696), and *m/z* = 367.5667 [**cyc•AD–DA•DA'‐NN‐AD'**]^4+^ (calc. for C_76_H_56_N_16_O_4_Pd_2_^4+^, 367.5696). Interestingly, the ^1^H NOESY spectra for these two complexes differed substantially, suggesting different topological arrangement. With the shorter **AD‐DA** ligand, through‐space NOE correlations were observed between H_a_ and H_i'_ and between H_d_ and H_m'_ (Figure 7c and e). Both correlations suggest the preferred conformation places the cationic Pd(II) panel near the electron‐rich anthracene linker of **DA'‐NN‐AD'**, as expected. However, they suggested different respective orientations to the two pairings within each structure (Figure 7c). As with the **AD‐DA** complex, through‐space NOE correlations between **‐NN‐** aromatic protons environments and the binding sites of both ligands were observed in **cyc•AD–DA•DA'‐NN‐AD'**. However, the exact identity of the correlations differs completely between species.

In this case, NOE correlations were observed between H_c_ and H_g'_, H_d_ and H_g'_, H_d_ and H_f'_, H_d'_ and H_m'_, and between H_c'_ and H_j'_ (Figure 7d and f). The summation of the data indicates that with the shorter linker on the linear ligand, the two heteroleptic coordination sites are orientated in opposite direction to one another, while with the longer dialkynyl linker they both have the same respective orientation (Figure 7f). Further, the combination of analogous ligands with ‘mismatched’ binding sites, for example **DD‐NN‐DD** with **DD‐DD** and **DD‐NN‐DD** with **AD‐DA**, did not result in the clean formation of the intended cyclic foldamers (S2.1.4, S2.1.5). In summary, across the five attempted complexations, the spatial importance of regions of opposite electronic character is reaffirmed, as is the importance of alkynyl versus dialkynyl linkers and matched hydrogen‐bonded pairings.

### 2.7 2 + 2 complexes from AA:DD Pairings

Above we observed how the **AD‐** binding site can give different connectivity by virtue of the ability to form both homoleptic and heteroleptic pairings. We were interested in how the **AA:DD** pairing, which lacks this ability but has instead the capacity to maintain hydrogen bonding capability in both the HH and HT configurations, may behave in analogous systems in which only **AA‐** and **DD‐** binding sites are present.

**FIGURE 8:**
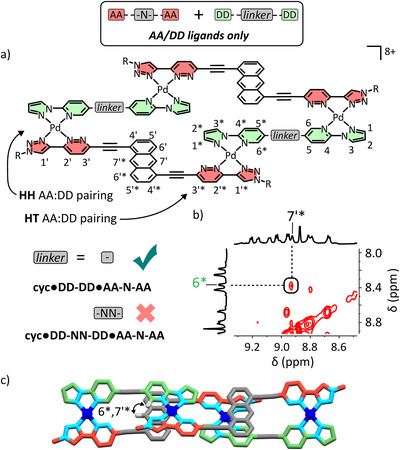
Chemical structures of (a) **cyc•DD‐DD•AA‐N‐AA** and **cyc •DD‐NN‐DD•AA‐N‐AA**, and (b) a partial ^1^H NOESY NMR spectrum (400 MHz, [D_6_]DMSO, 298 K, 200 ms) of **cyc•DD‐DD•AA‐N‐AA** with a select through‐space correlation depicted in (c) a computational model of **cyc•DD‐DD•AA‐N‐AA**.

The combination of **AA‐N‐AA** with **DD‐NN‐DD** and Pd(II) in a 1:1:2 ratio produced a complex mixture of products in solution (Figure 8). This is in line with previous findings given the electron rich anthracene linkers must align in the proposed thermodynamic product. In contrast, the combination of **AA‐N‐AA** with **DD‐DD** and Pd(II) in a 1:1:2 ratio did give a single species in solution as confirmed by ^1^H DOSY spectroscopy (*D* = 0.84 × 10^−10^ m^2^ s^−1^). Relative to the free ligands, the number of peaks in the new species doubled, indicating desymmetrization of the **AA‐N‐AA** and **DD‐DD** ligands, suggesting the formation of a larger species than a simple Pd_2_LL' type foldamer. HR ESI‐MS confirmed the species was instead of the form Pd_4_(**AA‐N‐AA**)_2_(**DD‐DD**)_2_. Through‐space NOE correlations between the alkyl chain of **AA‐N‐AA** and H_1_ indicate one of the two sets of environments corresponds to a HH‐ connection between **DD‐DD** and **AA‐N‐AA** about Pd(II) (see Figure S135). A through‐space NOE correlation was also observed between H_6*_ of the opposite **DD‐** binding site and H_7'*_ of the **‐N‐** linker, confirming these groups are close in space (Figure 8b and c). Favorable ‘cation’‐π interactions between these groups likely contributes to the enthalpic favorability of generating **cyc•DD‐DD•AA‐N‐AA**. The culmination of these data suggests **cyc•DD‐DD•AA‐N‐AA** adopts a conformation depicted in the model presented in Figure 8c, where each ligand is ‘split’ into two environments and possesses both a HH‐ **AA**:**DD** pairing and a HT **AA**:**DD** pairing π‐stacking with a **‐N‐** linker.

This is a different outcome from the preferred trinuclear Pd_3_(**DA'‐N‐AD')(AD‐DA**)_2_ product formed from combining **DA'‐N‐AD'** and **AD‐DA**, despite the fact that in both cases the combined ligands have complementary binding sites, and also in both cases one ligand has an alkyne linker while the other has a **‐N‐** linker. We attribute this to the fact that with **AA‐** and **DD‐** based ligands, the ability to form homoleptic environments is lost but the ability to form both HH and HT heteroleptic **AA:DD** pairings is gained. This capability in combination with the linker lengths of the ligands means the thermodynamic product in this case is not the Pd_3_(**AA‐N‐AA)(DD‐DD**)_2_, but the observed tetranuclear folded macrocycle. This example exemplifies the exploitation of the the isomerism of this pairing in order to overcome entropy and direct the self‐assembly of larger species.

### 2.8 Combinatorial Studies

**FIGURE 9:**
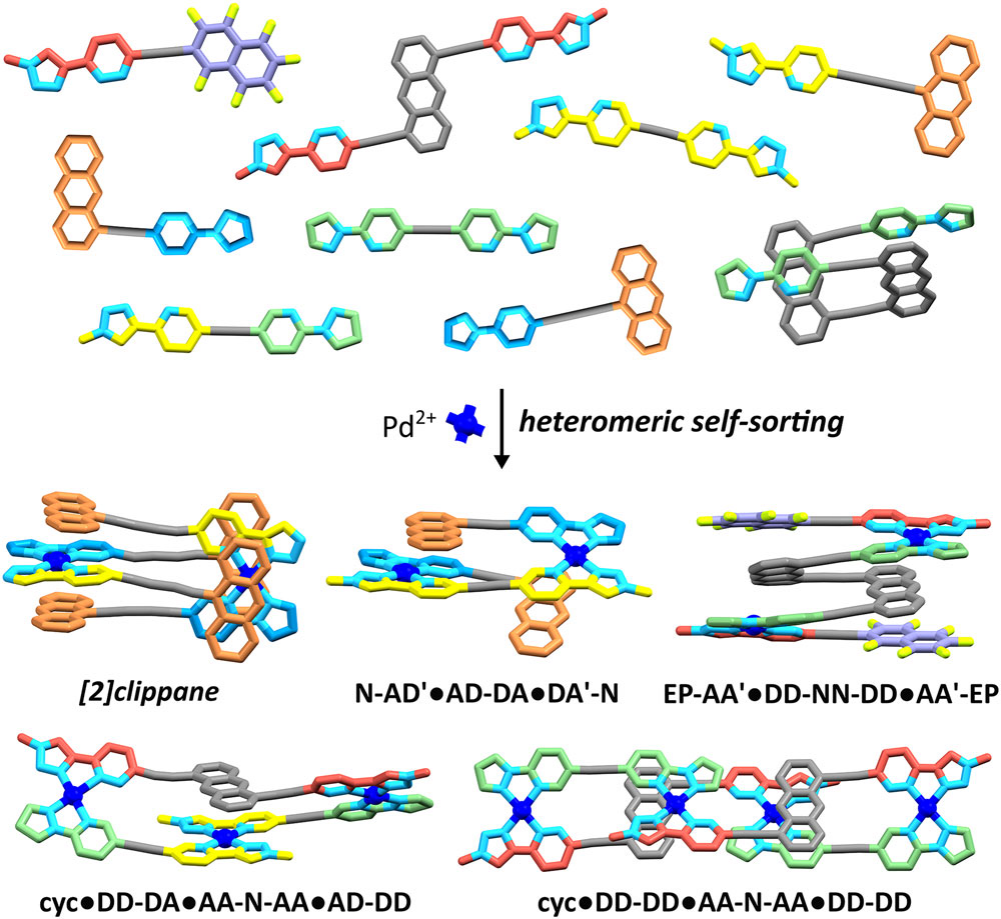
Molecular representations of the nine ligands that when combined in the correct stoichiometries with Pd(II) self‐assemble into five discrete complexes including: an interlocked architecture: [Pd(**AD–N')(DA'–N'**)]_2_; noncyclic foldamers: **N‐AD'•AD‐DA•DA'‐N** and **EP‐AA'•DD‐NN‐DD•AA'‐EP**; and foldameric macrocycles: **cyc•DD‐DA•AA‐N‐AA•AD‐DD** and **cyc•DD‐DD•AA‐N‐AA**.

Having established the requirements for the successful self‐assembly of individual Pd(II)‐based foldamers, we wanted to assess the orthogonality of the different complexes (Figure 9).

To do so, NMR‐scale solutions of an interlocked architecture: [Pd(**AD–N'**)(**DA'–N'**)]_2_; non‐cyclic foldamers: **N‐AD'•AD‐DA•DA'‐N** and **EP‐AA'•DD‐NN‐DD•AA'‐EP**; and macrocycles: **cyc•DD‐DA•AA‐N‐AA•AD‐DD** and **cyc•DD‐DD•AA‐N‐AA**, were combined into a single solution.

**FIGURE 10:**
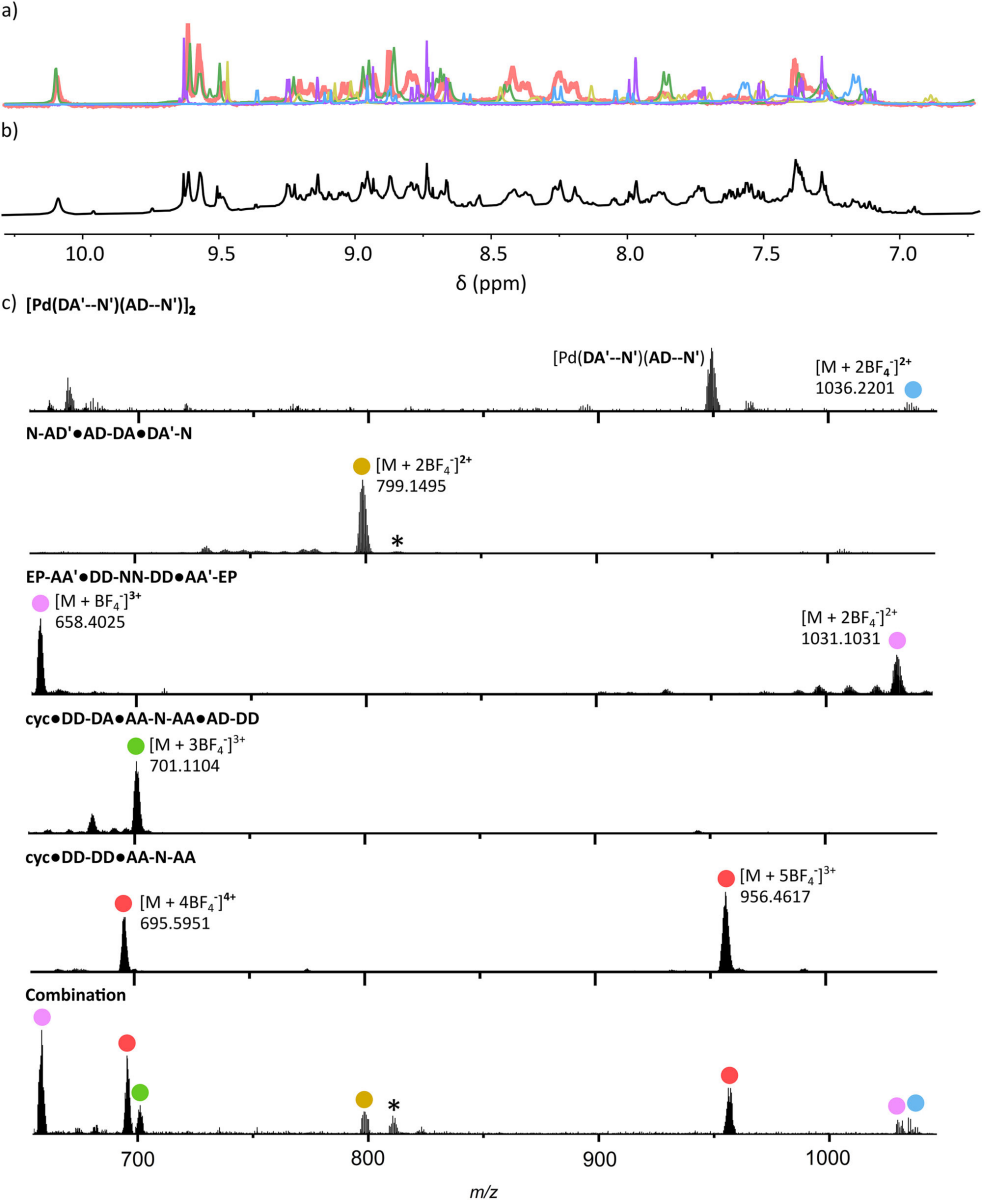
(a) The superimposed partial ^1^H NMR spectra ([D_6_]DMSO, 400 MHz, 298 K) of [Pd(**AD–N′**)(**DA′–N′**)]_2_, **N‐AD′•AD‐DA•DA′‐N, EP‐AA′•DD‐NN‐DD•AA′‐EP**, **cyc•DD‐DA•AA‐N‐AA•AD‐DD** and **cyc•DD‐DD•AA‐N‐AA**, b) partial ^1^H NMR spectra ([D_6_]DMSO, 400 MHz, 298 K) of the combination of solutions of the above species and c) the stacked HR ESI‐MS of each of these complexes as labelled relative to the combination of the complexes. *Note at *m/z* = 810.6442 a peak was observed 24 mass units above that seen for [**N‐AD′•AD‐DA•DA′‐N** + 5BF_4_^−^]^3+^ that could not be assigned. MS/MS of this species revealed further fragmentation into species containing both the **DA′‐N** and **AD‐DA** ligands, and no other ligand could be identified. No other combination of ligands with **DA′‐N** and **AD‐DA** gives a species with this molecular weight. Furthermore, small traces of the same adduct were observed in the mass spectrum of independently prepared **N‐AD′•AD‐DA•DA′‐N** (also denoted *) indicating that this adduct does not arise from complex scrambling.

Resolved peaks corresponding to the original ^1^H NMR spectra of the complexes (Figure 10a, color‐coded by complex) remained and little to no scrambling was evident (Figure 10b). Further, HR ESI‐MS of the combined solution demonstrated the presence of each of the individual complexes with no new species present aside from various adducts and fragments of the expected complexes (Figure 10c). Despite their modularity, with all ligands having **AA**, **DD**, **AD** or **DA'** hydrogen‐bonding sites, no scrambling is observed. Likewise, ligands such as **AD‐DA** and **DD‐DD** have the same linkage spacing, and even where the same ligand, **AA‐N‐AA** has been incorporated into two different complexes, the original complexes remain with no scrambling evident. combination presents the exciting ability to extend the combined use of pairings and π─π interactions to not only access complicated heteroleptic supramolecules but orthogonal mixtures of such species. The same is observed when Pd(II) is added to a DMSO solution of the free ligands, meaning orthogonal self‐assembly is possible in situ (Figure 9).

## 3 Conclusion

Our work here has established a set of design rules for controlling identity, position and topology across a broad range of structural types including an interlocked clippane, non‐cyclic foldamers, and macrocycles with persistent secondary structure. This control is imparted through the combination of coordination chemistry with square planar Pd(II), inter‐ligand hydrogen bonding, and π─π interactions. While these structures are diverse, all of these molecules have Pd(II) integrated directly into their primary sequences, where it is in fact essential for structural information transfer due to its planarity. The design rules are: • linker length must be such that it enables favorable π─π interactions between electron‐rich anthracene groups and cationic Pd(II)‐binding sites, and with two monotopic ligands will drive the formation of novel interlocked architectures when di‐ rather than mono‐alkynyl; • π─π interactions are not sufficiently enthalpically favorable when poorly aligned or existing between electron‐rich aromatic groups (anthracene only). They are however sufficiently favorable between electron‐rich aromatic groups (anthracene) and electron poor aromatic groups (perfluorinated naphthalene or cationic metal coordination environments); • In the formation of cyclic foldamers, alignment of electron‐rich aromatic groups prevents formation of the expected smallest, cyclic species. In some cases, cation‐π interactions drive self‐pairing of **AD‐** binding sites. This is enabled by the capacity of sites with both acceptor and donor capability to form homoleptic as well as heteroleptic coordination environments; • The balance between the entropic drive to the smallest cyclic species and enthalpic drive to cation‐π interactions can produce surprisingly strained species in which the linker length can greatly affect the preferred conformation of the resulting complexes; • The ability for the **AA:DD** pairing to maintain maximal hydrogen bonding in both HH and HT isomers but avoid self‐pairing can be exploited to form species where maximizing cation‐π interactions outweighs the formation of the smallest cyclic structure possible. • The same combination of coordination bonds, hydrogen bonds, π─π interactions and appropriate geometric lengths that gives rise to clean formation of the individual species is also sufficient to direct their individuated assembly out of mixtures. Together, these findings improve our ability to controllably form self‐assembled structures with persistent secondary structure. Analogous to the relationship between primary, secondary and tertiary structure of proteins, we can predict the preferred combination and folding of these Pd(II)‐based species based on the ligand identities. Further, akin to how proteins can exist orthogonally in biological systems, a selection of these synthetic molecules persist in solution even when combined or formed in situ.

## Conflicts of Interest

The authors declare no conflicts of interest.

## Supporting information



The authors have cited additional references with the Supporting Information [62, 63, 76, 80, 81, 82, 83, 84, 85, 86, 87, 88, 89, 90, 91, 92, 93, 94, 95, 96, 97, 98, 99, 100].**Supporting File 1**: anie72111‐sup‐0001‐SuppMat.pdf.

## Data Availability

The data that support the findings of this study are available from the corresponding author upon reasonable request.
