# Supplementary material for: Design Rules for Controlling Connectivity, Topology, and Sorting Using Hydrogen‐Bonded Pairs and Aromatic Components in Palladium(II)‐Based Interlocked and Foldameric Systems
Source: Angew Chem Int Ed Engl. 2026 Apr 10;65(22):e5763518. doi: 10.1002/anie.5763518 (PMC13206510; doi:10.1002/anie.5763518)
Supplement: Supplementary file 1 — The authors have cited additional references with the Supporting Information [62, 63, 76, 80, 81, 82, 83, 84, 85, 86, 87, 88, 89, 90, 91, 92, 93, 94, 95, 96, 97, 98, 99, 100].Supporting File 1: anie72111‐sup‐0001‐SuppMat.pdf. [file ANIE-65-e5763518-s001.pdf]

Supporting Information

Jess L. Algar,[a] Jordan N. Smith,[a] Dan Preston[a]

[a]Research School of Chemistry, The Australian National University, Canberra, ACT 2600, Australia.

***daniel.preston@anu.edu.au**

Contents

1. Experimental 5
  1.1. General 5
    1.1.1. General complexation details 5
    1.1.2. NMR pulse programs and parameters 5
  1.2. Precursors 6
    1.2.1. General preparation of diiodoanthracenes 6
    1.2.2. A 6
    1.2.3. B 7
    1.2.4. C 8
    1.2.5. AA'-H 9
    1.2.6. AD'-Br 10
  1.3. AD ligands 12
    1.3.1. AD--DA 12
    1.3.2. AD-N-DA 13
    1.3.3. AD-NN-DA 14
    1.3.4. AD'-N' 16
    1.3.5. AD--N' 17
  1.4. DA' ligands 18
    1.4.1. DA'-N 18
    1.4.2. DA'--N 20
    1.4.3. DA'-N-AD' 21
    1.4.4. DA'-NN-AD' 22
    1.4.5. DA'-N' 23
    1.4.6. DA'--N' 24
  1.5. DD ligands 26
    1.5.1. DD-NN-DD 26
    1.5.2. DD--N' 27
  1.6. AA ligands 28
    1.6.1. AA-N-AA 28
    1.6.2. AA'--N' 29
  1.7. Interlocked Architectures 30
    1.7.1. $[Pd(DA'\text{-}N')_2](BF_4)_2$ 30
    1.7.2. $[Pd(AD'\text{-}N')_2](BF_4)_2$ 32

1.7.3. [Pd(DA'--N')$_2$](BF$_4$)$_2$ 34

1.7.4. [Pd(AD--N')$_2$](BF$_4$)$_2$ 36

1.7.5. [Pd(DA'--N')(AD--N')]$_2$(BF$_4$)$_4$ 40

1.7.6. [Pd(DA'--N)(AD--N')]$_2$(BF$_4$)$_4$ 43

1.7.7. [Pd(DD--N')(AA--N')]$_2$(BF$_4$)$_4$ 46

1.8. Non cyclic foldamers 49

1.8.1. N-AD'●AD-DA●DA'-N 49

1.8.2. N--AD'●AD--DA●DA'--N 52

1.8.3. N-AD'●AD--DA●DA'-N 55

1.8.4. EP-DD●AA-N-AA●DD-EP 58

1.8.5. EP-AA●DD-NN-DD●AA-EP 61

1.9. Macrocycles 64

1.9.1. *cyc*●AD--DA●DA'-N-AD' 64

1.9.2. *cyc*●AD-DA●DA'-N-AD'●AD-DA 67

1.9.3. *cyc*●DD-DA●AA-N-AA●AD-DD 70

1.9.4. *cyc*●AD-DA●AA-N-AA●AD-DA 73

1.9.5. *cyc*●AD-DA●DA'-NN-AD' 76

1.9.6. *cyc*●AD--DA●DA'-NN-AD' 79

1.9.7. *cyc*●DD-DD●AA-N-AA 82

2. Unsuccessful self-assemblies 85

2.1.1. [Pd(DA'-N')(AD-N')]$_2$(BF$_4$)$_4$ 85

2.1.2. N'-AD'●AD-DA●DA'-N' 86

2.1.3. EP-AA'●DD-DD●AA'-EP 87

2.1.4. *cyc*●DD-DD●DD-N-DD 89

2.1.5. *cyc*●AD-DA●DD-N-DD 90

2.1.6. N-DA'●AD-N-DA●DA'-N 92

2.1.7. N-DA'●AD-NN-DA●DA'-N 93

2.1.8. *cyc*●DA'-NN-AD'●AD-NN-DA 95

3. Stacked Spectra 96

3.1.1. [2]Clippane mixing studies 96

3.1.2. Quantitative NMR for -N- type complexes 97

4. Combinatorial Studies 98

5. Calculations 98

6. Crystallography 99

6.1. Experimental data 99
7. References 102

1. Experimental

1.1. General

Unless otherwise stated, all reagents were purchased from commercial sources and used without further purification, except for:

1-ethynylanthracene[1]
4-(1-anthracenyl)-2-methyl-3-butyn-2-ol[1]
2-(2-Methoxyethoxy)ethyl p-toluenesulfonate[2]
5-Bromo-2-[2-(trimethylsilyl)ethynyl]pyridine[3]
3,6-Bis[2-(trimethylsilyl)ethynyl]pyridazine[4]
9-(1,3-Butadiyn-1-yl)anthracene[5]

which were synthesised according to literature procedures.

AD-Br, AD-I, AD-TMS, DD-TMS, DA'-Br, DA'-I, DA'-TMS, AA-H, AD-DA, DD-DD, AD-DD and AD-AA were all synthesised as we have reported previously here,[6] and AA-EP and DD-EP here.[7]

Solvents were laboratory reagent grade. Petroleum ether refers to the fraction of petrol boiling in the range 40 – 60 °C. Abbreviations: dichloromethane (DCM), ethylenediaminetetraacetate (EDTA), tetrahydrofuran (THF), triethylamine (TEA), dimethyl sulfoxide (DMSO), dimethylformamide (DMF). ^{1}H and ^{13}C NMR spectra were recorded on either a Bruker Avance 400 MHz, 600 MHz or 700 MHz spectrometer. Chemical shifts are reported in parts per million and referenced to *residual solvent peaks*, labelled in spectral Figure Ss for the fully deuterated solvent used ($CDCl_3$: ^{1}H δ 7.26 ppm, ^{13}C δ 77.16 ppm; [D_6]DMSO: ^{1}H δ 2.50 ppm, ^{13}C δ 39.52 ppm). Coupling constants (*J*) are reported in Hertz (Hz). Standard abbreviations indicating multiplicity were used as follows: m = multiplet, q = quartet, quin = quintet, t = triplet, dt = double triplet, d = doublet, dd = double doublet, s = singlet, br = broad. Electrospray mass spectra (HR ESI-MS) were collected on a Bruker micrOTOF-Q spectrometer or a Waters Synapt G2-S1 HDMS spectrometer. Nanospray MS were collected on a Fusion Orbitrap spectrometer.

CAUTION: WHILE NO PROBLEMS WERE ENCOUNTERED DURING THIS WORK, AZIDES ARE EXPLOSIVE AND CARE SHOULD BE TAKEN WHEN DEALING WITH THEM.

1.1.1. General complexation details

For NMR studies, the complexes were generated and characterised *in situ*.

In *[D_6]dimethylsulfoxide*: the ligand and $[Pd(CH_3CN)_4](BF_4)_2$ were combined in the correct stoichiometries using prepared stock solutions to enable accuracy and administered using a micropipette.

1.1.2. NMR pulse programs and parameters

All ^{1}H 1D TOCSY NMR spectra were obtained using the selmlgp pulse program[8] within Topspin.

All ^{1}H 2D NOESY NMR spectra were obtained using the noesygpphpp pulse program[9] within Topspin. Relaxation time was set to 200 ms.

All ^{1}H DOSY NMR spectra were obtained using the convection compensated dstebpgp3s pulse sequence[10] within Topspin. The spectra were obtained with δ = 2.0 ms, Δ = 100 ms, and g = 2% – 95% in [D_6]DMSO. Processing was carried out in MestreNova.

1.2. Precursors

1.2.1. General preparation of diiodoanthracenes

1,5-diiodoanthracene and 1,8-diiodoanthracene were prepared from commercially available dibrominated precursors according to the following general procedure. 1,X-dibromoanthracene (1 eq.), potassium iodide (10 eq.), CuI (0.5 eq.) and *N,N*-dimethylethylenediamine (0.5 eq.) were stirred in dioxane in a sealed tube under N_2 at 140°C for two nights with the exclusion of light. After cooling and adding DCM (50 mL) the organic layer was washed with water (2 x 100 mL), collected and dried under vacuum. Quantitative conversion was achieved. Spectral data is consistent with that previously reported for 1,5-diiodoanthracene[11] and 1,8-diiodoanthracene.[12]

1.2.2. A

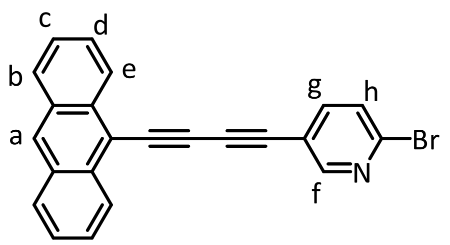

2-bromo-5-iodopyridine (1.51 g, 5.30 mmol), 9-(1,3-Butadiyn-1-yl)anthracene[5] (1.00 g, 4.42 mmol), tris(dibenzylideneacetone)dipalladium(0) (202 mg, 0.221 mmol), triphenylphosphine (232 mg, 0.884 mmol), copper(I) iodide (84.2 mg, 0.442 mmol) were added to a deoxygenated solution of 2:1 THF/TEA (15 mL) against the flow of N_2. This was stirred at room temperature for 18 hours under a nitrogen atmosphere with the exclusion of light. After adding DCM (50 mL) and 0.1 M aqueous EDTA/NH_4OH (50 mL) and stirring vigorously for 30 minutes, the organic layer was removed under vacuum, the residue taken up again in DCM (100 mL), washed with water (100 mL), and removed under vacuum. Purification by column chromatography on silica (PET to 1:1 PET/DCM) gave the product as a yellow powder (1.26 g, 3.30 mmol, 75%).

^{1}H NMR (400 MHz, $CDCl_3$, 298 K) δ: 8.60 (1H, br, H_f), 8.54 (2H, d, J = 8.8 Hz, H_e), 8.49 (1H, s, H_a), 8.03 (2H, d, J = 8.8 Hz, H_b), 7.71 (1H, dd, J = 8.5 Hz, 2.3 Hz, H_h), 7.63 (2H, t, J = 7.30 Hz, H_d), 7.55-7.50 (3H, m, H_c, H_g).

^{13}C NMR (100 MHz, $CDCl_3$, 298 K) δ: 153.2, 142.0, 141.1, 134.2, 131.0, 129.5, 129.0, 127.8, 127.5, 126.3, 126.0, 118.7, 114.8, 83.9, 80.9, 79.5, 78.9.

IR v (cm^{-1}) 3053, 2923, 2853, 2202, 1739, 1671, 1600, 1573, 1538, 1455, 1356, 1320.

HR ESI-MS (DCM/methanol) m/z = 382.0224 $[MH]^+$ (calc. for $C_{23}H_{13}NBr$, 382.0231).

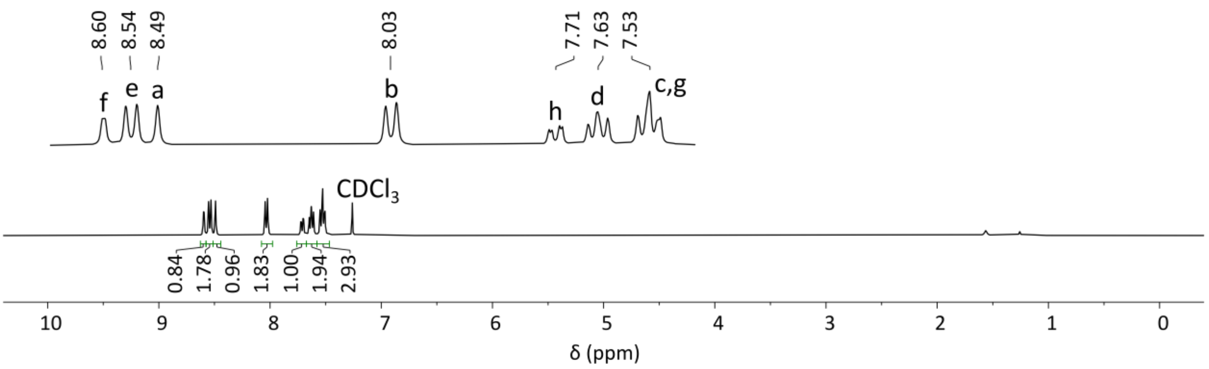

Figure S1 ^{1}H NMR spectrum (400 MHz, $CDCl_3$, 298 K) of **A**.

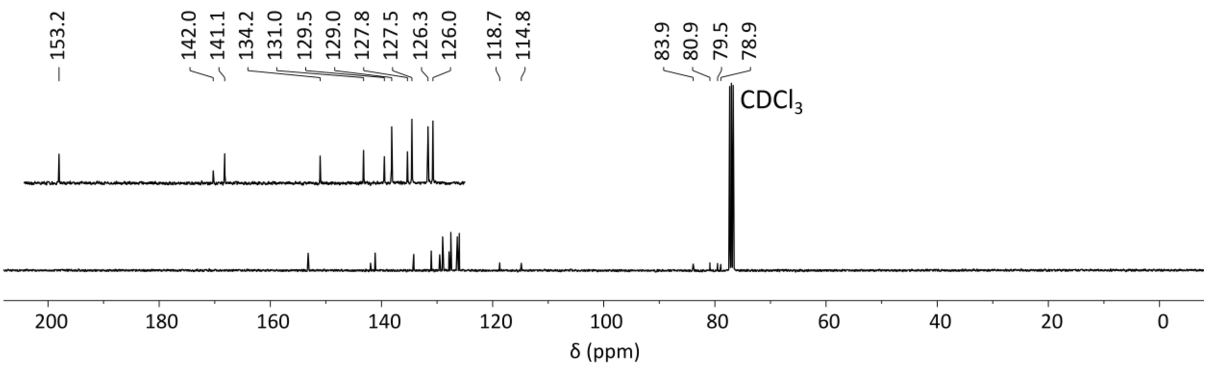

Figure S2 ^{13}C NMR spectrum (100 MHz, $CDCl_3$, 298 K) of **A**.

1.2.3. B

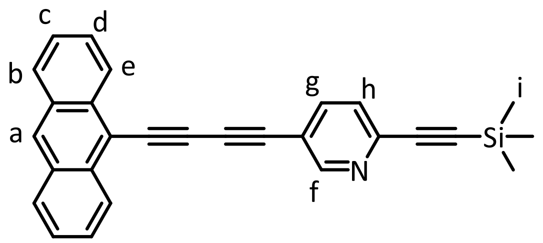

A (420 mg, 1.10 mmol), [$Pd(PPh_3)_2Cl_2$] (39 mg, 0.055 mmol) and CuI (21 mg, 0.11 mmol) were added to a deoxygenated solution of 1:1 THF/TEA (10 mL) against the flow of N_2. Ethynyl trimethysilane (162 mg, 1.65 mmol) was added last, the flask sealed and heated at 40°C overnight with stirring. The reaction mixture was added to DCM (100 mL) and 0.1 M EDTA/NH_4OH (0.1 M, 100 mL) and stirred vigorously for 30 minutes. The organic layer was washed with water (100 mL) and the solvent removed under vacuum. Purification by column chromatography on silica (DCM to 1:5 acetone/DCM) gave the product as a yellow powder (400 mg, 1.00 mmol, 91%).

^{1}H NMR (400 MHz, $CDCl_3$, 298 K) *δ*: 8.79 (1H, br, H_f), 8.55 (2H, d, *J* = 8.8 Hz, H_e), 8.49 (1H, s, H_a), 8.04 (2H, d, *J* = 8.8 Hz, H_b), 7.83 (1H, dd, *J* = 8.1 Hz, 2.0 Hz, H_h), 7.63 (2H, t, *J* = 7.40 Hz, H_d), 7.53 (2H, t, *J* = 7.90 Hz, H_c), 7.47 (1H, d, *J* = 8.1 Hz, H_g), 0.29 (9H, s, H_i).

^{13}C NMR (100 MHz, $CDCl_3$, 298 K) *δ*: 153.5, 142.5, 139.5, 134.5, 131.4, 129.8, 129.3, 127.8, 126.9, 126.7, 126.3, 118.9, 115.3, 103.6, 98.4, 84.4, 81.5, 81.0, 79.7, 0.0007.

IR v (cm^{-1}) 3054, 2960, 2925, 2900, 2853, 2163, 1736, 1670, 1582, 1540, 1466, 1457, 1366.

HR ESI-MS (DCM/methanol) m/z = 400.1522 $[MH]^+$ (calc. for $C_{28}H_{22}NSi$, 400.1522).

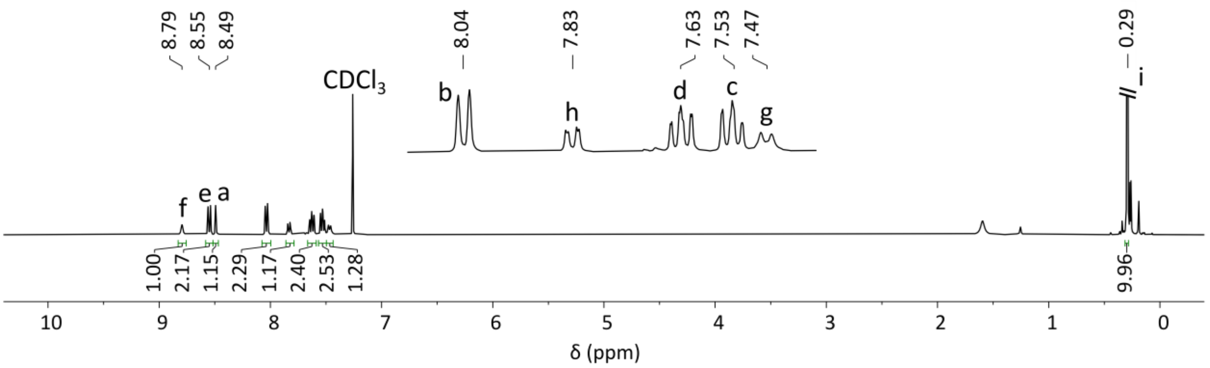

Figure S3 ^1H NMR spectrum (400 MHz, $CDCl_3$, 298 K) of **B**.

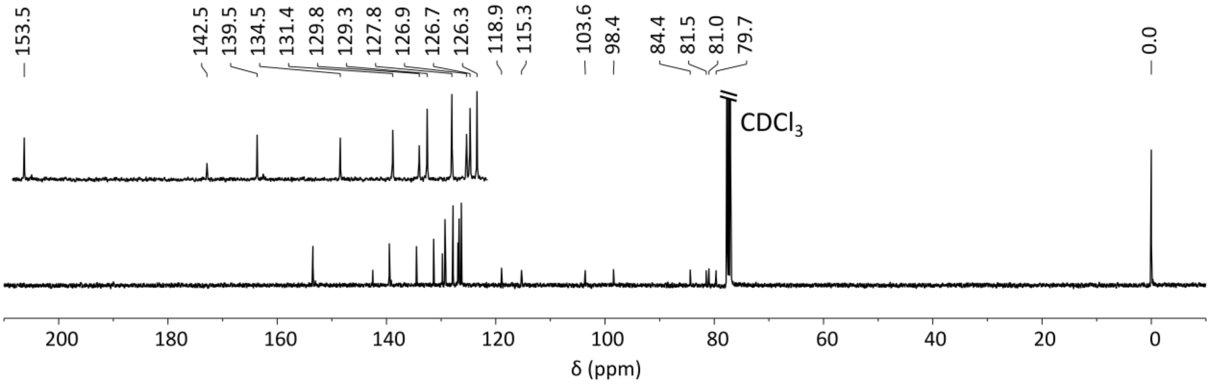

Figure S4 ^{13}C NMR spectrum (100 MHz, $CDCl_3$, 298 K) of **B**.

1.2.4. C

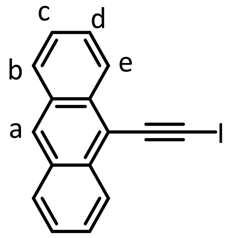

A solution of 9-(triisopropylsilyl)ethynyl anthracene[13] (2000 mg, 5.58 mmol) and TBAF (1.0 M in THF, 8.40 mL, 8.37 mmol) in THF was stirred at room temperature for two hours. DCM (100 mL) was added, and the organic layer washed with distilled water (1 x 100 mL) and the volatiles removed under reduced pressure, as per literature procedures.[14] The resulting residue was added in crude form to a mixture of *N*-iodosuccinimide (1510 mg, 6.69 mmol), and silver(I) fluoride (778 mg, 6.14 mmol) in deoxygenated acetonitrile (40 mL). This was stirred at room temperature for 21 hours under a nitrogen atmosphere with the exclusion of light. The resulting mixture was filtered through celite with DCM (100 mL), washed with a saturated solution of $Na_2CO_{3(aq)}$ (2 x 100 mL) and volatiles removed under reduced pressure to afford the product as a dark red solid (1800 mg, 1.20 mmol, 98%). A small

impurity was visible by NMR but thought to not affect the compounds reactivity in subsequent steps if used in excess.

^{1}H NMR (400 MHz, $CDCl_3$, 298 K) δ: 8.51 (2H, d, J = 8.6 Hz, H_e), 8.39 (1H, s, H_a), 7.98 (2H, d, J = 8.6 Hz, H_b), 7.60-7.56 (2H, m, H_d), 7.51-7.47 (2H, m, H_c).
^{13}C NMR (100 MHz, $CDCl_3$, 298 K) δ: 133.8, 131.0, 128.9, 128.4, 127.7, 127.1, 126.5, 125.8, 91.5, 18.3.

HR ESI-MS (MeOH) m/z = 328.9821 $[MH]^+$ (calc. for $C_{16}H_9I$, 328.9827).

IR ν (cm^{-1}) 3051, 2959, 2943, 2866, 1947, 1709, 1622, 1544.

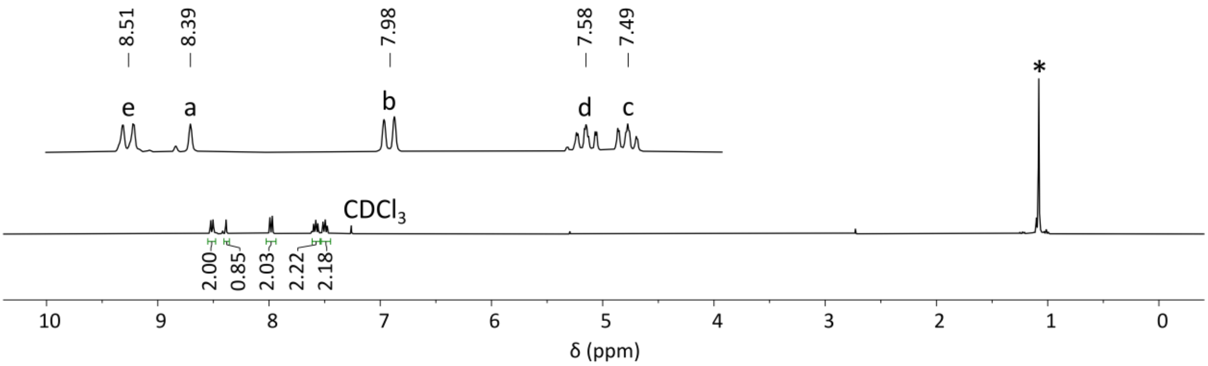

Figure S5 ^{1}H NMR spectrum (400 MHz, $CDCl_3$, 298 K) of **C**. *An impurity from the TIPS protected starting material was unable to be separated from the product but did not affect the reactivity of the compound.

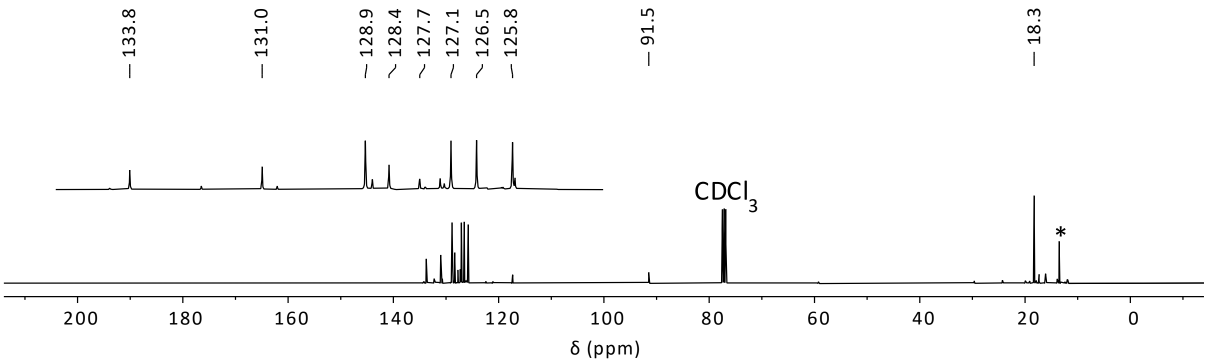

Figure S6 ^{13}C NMR spectrum (100 MHz, $CDCl_3$, 298 K) of **C**. *An impurity from the TIPS protected starting material was unable to be separated from the product but did not affect the reactivity of the compound.

1.2.5. AA'-H

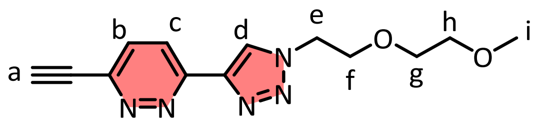

The combination of 2-(2-methoxyethoxy)ethyl p-toluenesulfonate[2] (225 mg, 0.820 mmol) and NaN_3 (41.0 mg, 0.631 mmol) in DMF (5 mL) was heated at 110 °C for 1.5 hours. This was added to 3,6-bis[2-(trimethylsilyl)ethynyl]pyridazine[4] (430 mg, 1.58 mmol), sodium carbonate (73.5 mg, 0.694 mmol), sodium ascorbate (62.5 mg, 0.315 mmol) and $Cu(SO_4)_2{\cdot}5H_2O$ (78.7 mg, 0.315 mmol) in DMF/H_2O (7:1, 10 mL) and the reaction was stirred overnight at 50°C. After adding DCM (100 mL) and 0.1 M aqueous EDTA/NH_4OH (100 mL) and stirring vigorously for 30 minutes, the organic layer was

removed under vacuum, the residue taken up again in DCM (100 mL), washed with water (100 mL), and removed under vacuum. Purification by column chromatography on silica (DCM to 1:4 acetone/DCM) gave the product as a white solid (92.6 mg, 0.341 mmol, 54%).

^{1}H NMR (400 MHz, $CDCl_3$, 298 K) δ: 8.62 (1H, s, H_d), 8.30 (1H, d, J = 8.7 Hz, H_c), 8.30 (1H, d, J = 8.7 Hz, H_b), 4.67 (2H, t, J = 4.9 Hz, H_e), 3.94 (2H, t, J = 4.9 Hz, H_f), 3.65-3.63 (2H, m, H_g), 3.54-3.52 (2H, m, H_h), 3.44 (1H, s, H_a), 3.38 (3H, s, H_i).

^{13}C NMR (100 MHz, $CDCl_3$, 298 K) δ: 152.1, 146.0, 145.0, 130.6, 124.7, 122.7, 82.0, 80.3, 72.0, 70.9, 69.5, 59.3, 50.8.

HR ESI-MS (MeOH) m/z = 274.1307 $[MH]^+$ (calc. for $C_{13}H_{15}N_5O_2$, 274.1304).

IR ν (cm^{-1}) 3227, 3118, 3075, 3054, 2926, 2878, 2831, 2112, 1591, 1455, 1426.

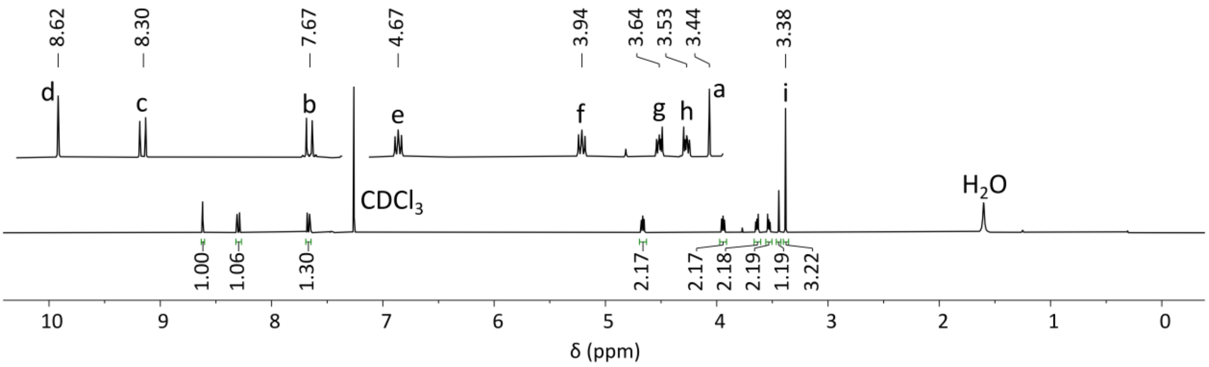

Figure S7 ^{1}H NMR spectrum (400 MHz, $CDCl_3$, 298 K) of **AA'-H**.

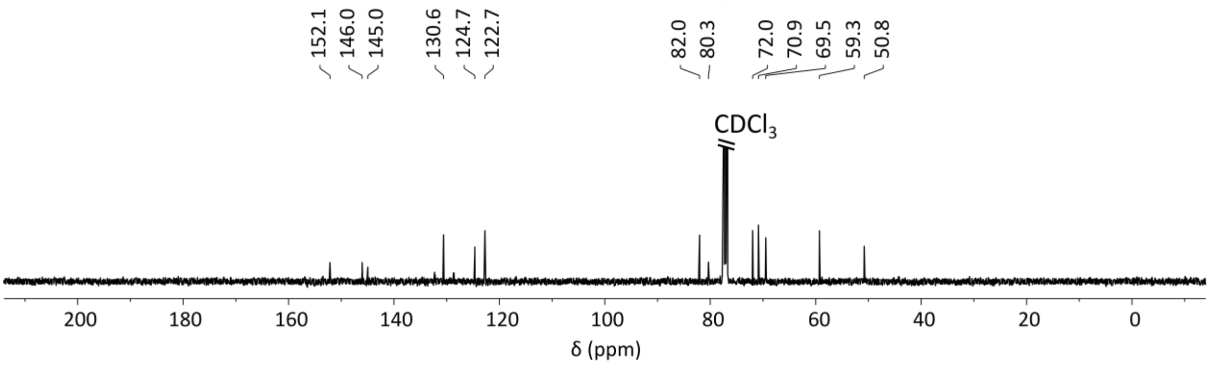

Figure S8 ^{13}C NMR spectrum (100 MHz, $CDCl_3$, 298 K) of **AA'-H**.

1.2.6. AD'-Br

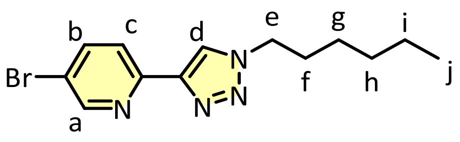

The combination of 1-bromohexane (1.30 g, 7.90 mmol) and NaN_3 (0.383 g, 5.93 mmol) in DMF (10 mL) was heated at 110 °C for 1.5 hours. This was added to 5-bromo-2-[2-(trimethylsilyl)ethynyl]pyridine[3] (1.00 g, 3.93 mmol), sodium carbonate (0.627 g, 5.93 mmol), sodium

ascorbate (0.783 g, 3.93 mmol) and $Cu(SO_4)_2 \cdot 5H_2O$ (0.493 g, 1.97 mmol) in DMF (10 mL) and the reaction was stirred overnight under nitrogen. After adding DCM (100 mL) and 0.1 M aqueous EDTA/NH_4OH (100 mL) and stirring vigorously for 30 minutes, the organic layer was removed under vacuum, the residue taken up again in DCM (100 mL), washed with water (100 mL), and removed under vacuum. Purification by column chromatography on silica (DCM to 1:19 acetone/DCM) gave the product as a white solid (0.927 g, 2.99 mmol, 76%).

^{1}H NMR (400 MHz, $CDCl_3$, 298 K) δ: 8.59 (1H, d, J = 2.1 Hz, H_a), 8.09 (1H, s, H_d), 8.05 (1H, d, J = 8.5 Hz, H_c), 8.59 (1H, dd, J = 8.5 Hz, 2.1 Hz, H_b), 4.38 (2H, t, J = 7.2 Hz, H_e), 1.92 (2H, tt, J = 7.2 Hz, H_f), 1.35-1.25 (6H, m, H_g, H_h, H_i), 0.85 (3H, t, J = 6.9 Hz, H_j).

^{13}C NMR (100 MHz, $CDCl_3$, 298 K) δ: 150.5, 149.0, 147.5, 139.5, 122.1, 121.4, 119.5, 50.7, 31.2, 30.3, 26.2, 22.5, 14.0.

HR ESI-MS (MeOH) m/z = 309.0718 $[MH]^+$ (calc. for $C_{13}H_{17}N_4Br$, 309.0715).

IR ν (cm^{-1}) 3133, 3120, 2953, 2926, 2855, 1592, 1537, 1456, 1431, 1359.

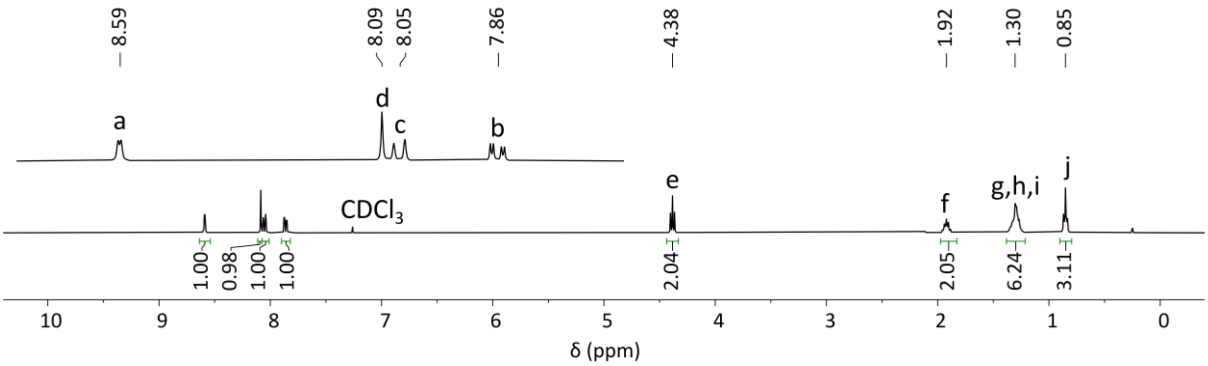

Figure S9 ^{1}H NMR spectrum (400 MHz, $CDCl_3$, 298 K) of **AD'-Br**.

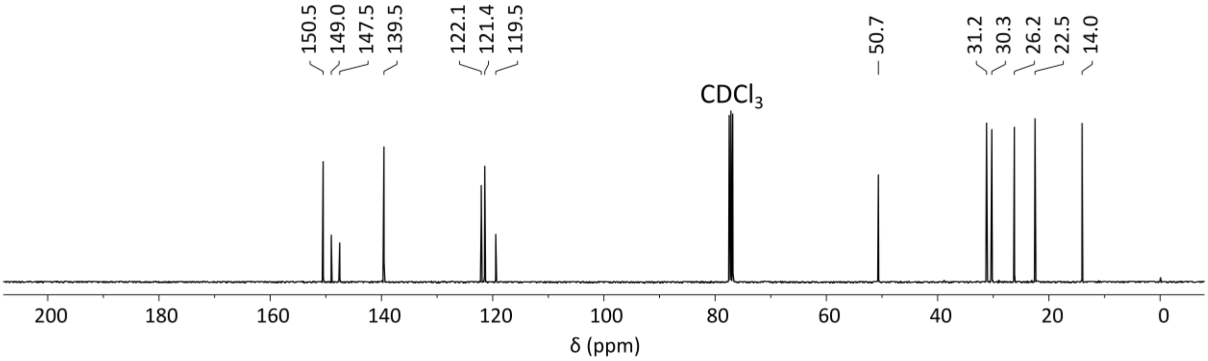

Figure S10 ^{13}C NMR spectrum (100 MHz, $CDCl_3$, 298 K) of **AD'-Br**.

1.3. AD ligands

1.3.1. AD--DA

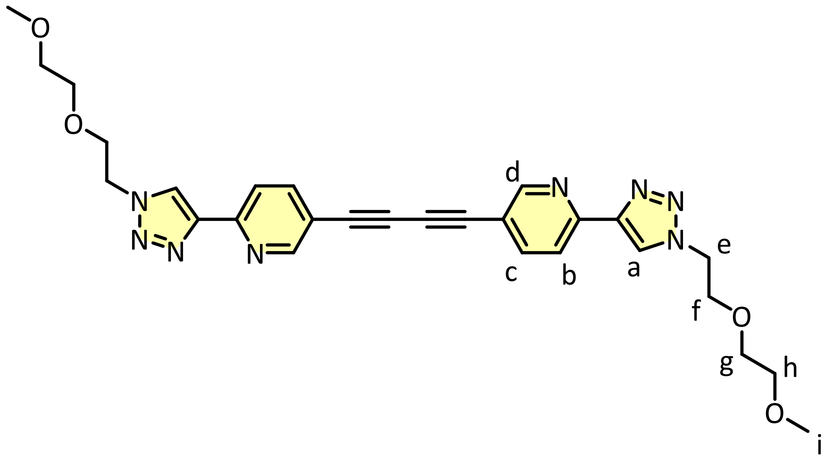

The ligand **AD--DA** was produced as the homocoupled byproduct in the formation of **AD-DA**.[6] It was able to be separated using the same column chromatography conditions reported above to be isolated as a white solid (56 mg, 0.010 mmol, 18%).

^{1}H NMR (400 MHz, [D$_6$]DMSO, 298 K) δ: *integration given per 'half' of the ligand* 8.86 (1H, dd, J = 2.1 Hz, 0.8 Hz, H_D), 8.67 (1H, s, H_A), 8.15 (1H, dd, J = 8.3 Hz, 2.2 Hz, H_C), 8.09 (1H, dd, J = 8.1 Hz, 1.0 Hz, H_B), 4.64 – 4.61 (2H, m, H_E), 3.89 – 3.87 (2H, m, H_F), 3.56 – 3.48 (2H, m, H_G), 3.41 – 3.37 (2H, m, H_H), 3.20 (3H, s, H_I).

D ($\times$ 10^{-10} m^2 s^{-1}, 400 MHz, [D$_6$]DMSO, 298 K) = 0.97.

^{13}C NMR (100 MHz, CDCl$_3$, 298 K) δ: 153.0, 150.1, 147.7, 140.2, 124.1, 119.5, 117.5, 79.9, 77.1, 71.8, 70.7, 69.5, 59.1, 50.6.

HR ESI-MS (DCM/methanol) m/z = 543.2457 [MH]$^+$ (calc. for $C_{28}H_{31}N_8O_4$, 543.2468).

IR v (cm^{-1}) 3125, 2956, 2920, 2890, 2870, 2815, 1730, 1592, 1466, 1441, 1361.

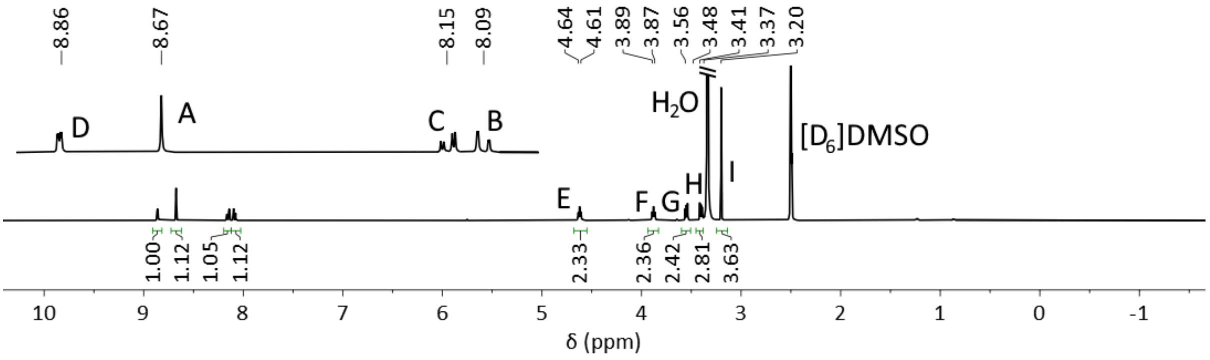

Figure S11 ^{1}H NMR spectrum (400 MHz, [D$_6$]DMSO, 298 K) of **AD--DA**.

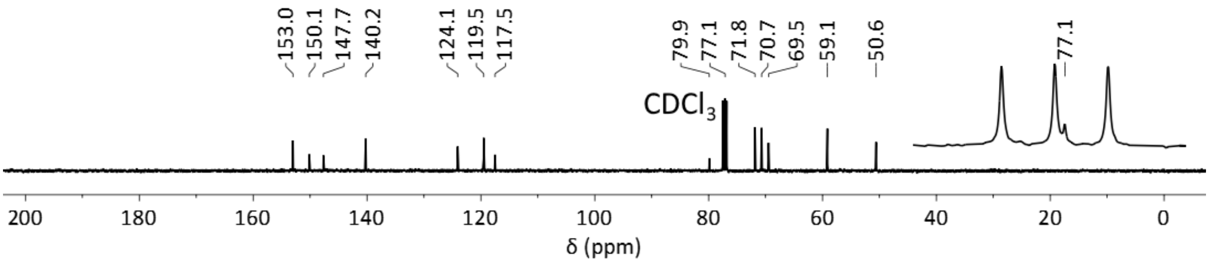

Figure S12 ^{13}C NMR spectrum (100 MHz, $CDCl_3$, 298 K) of **AD--DA**.

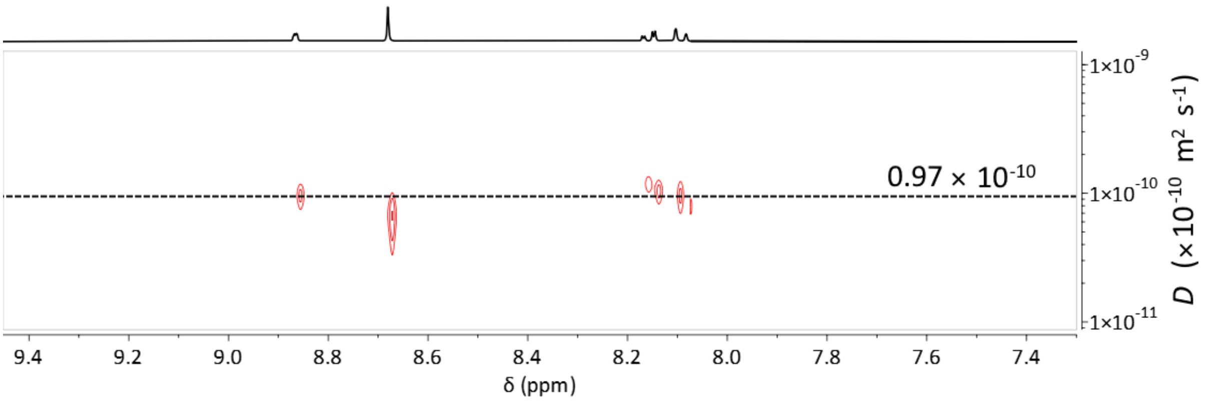

Figure S13 ^{1}H DOSY NMR spectrum (400 MHz, [D$_6$]DMSO, 298 K) of **AD--DA**.

1.3.2. AD-N-DA

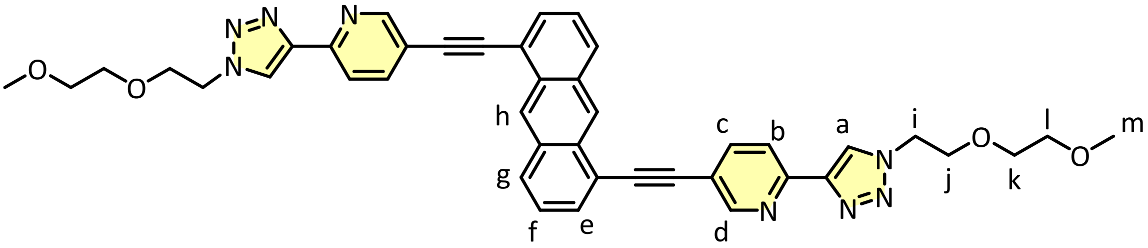

The combination of **AD-TMS** (300 mg, 0.868 mmol) and Na_2CO_3 (58 mg, 0.54 mmol) was stirred in methanol (50 mL) for 1 hour. After adding DCM (100 mL) and washing with water (100 mL), the organic layer was removed under vacuum. The residue was combined with 1,5-diiodoanthracene (156 mg, 0.362 mmol), [Pd_2(dba)$_3$] (8 mg, 0.009 mmol), triphenylphosphine (9 mg, 0.04 mmol) and CuI (7 mg, 0.04 mmol) in 1:1 THF/TEA (15 mL) under nitrogen and heated at 40 °C overnight. The reaction mixture was added to DCM (100 mL) and EDTA/NH_4OH (100 mL) and stirred vigorously for 30 minutes. The organic layer was washed with water (100 mL) and the solvent removed under vacuum. Purification by column chromatography on silica (DCM to 1:3 acetone/DCM to 1:1 acetone/DCM to 1:9 methanol/DCM) gave the product as a yellow solid (164 mg, 0.228 mmol, 63%).

^{1}H NMR (400 MHz, $CDCl_3$, 298 K) δ: *integration given per 'half' of the ligand* 9.01 (1H, s, H_h), 8.92 (1H, d, J = 2.1 Hz, H_d), 8.39 (1H, s, H_a), 8.26 (1H, d, J = 7.2 Hz, H_b), 8.15 (1H, d, J = 8.4 Hz, H_g), 8.07 (1H, dd, J = 8.2 Hz, 2,2 Hz, H_c), 7.86 (1H, d, J = 7.0 Hz, H_e), 7.54 (1H, dd, J = 8.5 Hz, 6.9 Hz, H_f), 4.67 – 4.62 (2H, m, H_i), 3.97 – 3.92 (2H, m, H_j), 3.66 – 3.62 (2H, m, H_k), 3.57 – 3.54 (2H, m, H_l), 3.40 (3H, s, H_m).

Poor solubility precluded the collection of a ^{1}H DOSY NMR spectrum.

^{13}C NMR (100 MHz, $CDCl_3$, 298 K) δ: 152.2, 149.6, 148.0, 139.5, 131.8, 131.3, 131.3, 130.2, 125.9, 125.5, 123.9, 120.5, 119.8, 119.4, 91.9, 91.3, 72.0, 70.8, 69.6, 59.2, 50.7.

HR ESI-MS (DCM/methanol) m/z = 719.3082 $[MH]^+$ (calc. for $C_{42}H_{39}N_8O_4$, 719.3094).

IR ν (cm^{-1}) 3137, 3067, 2919, 2878, 2823, 1769, 1724, 1674, 1595, 1523, 1484, 1460, 1442, 1394, 1366.

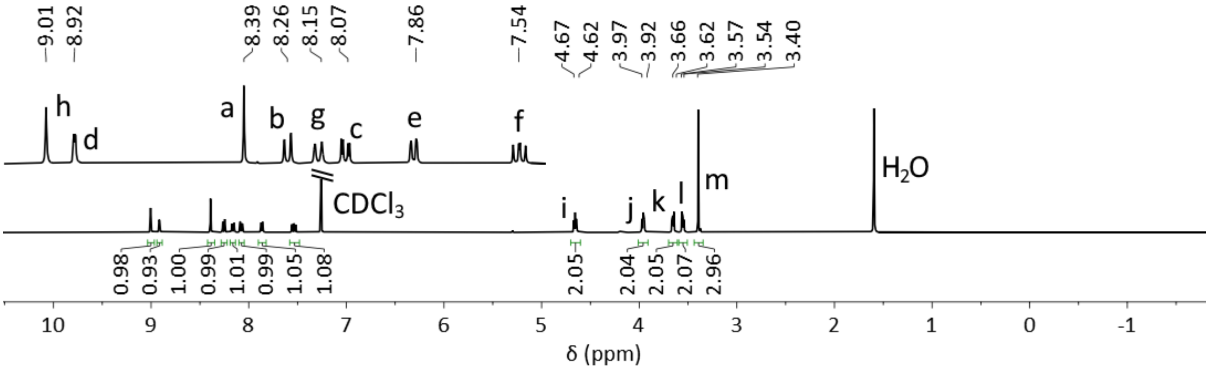

Figure S14 ^1H NMR spectrum (400 MHz, $[D_6]DMSO$, 298 K) of **AD-N-DA**.

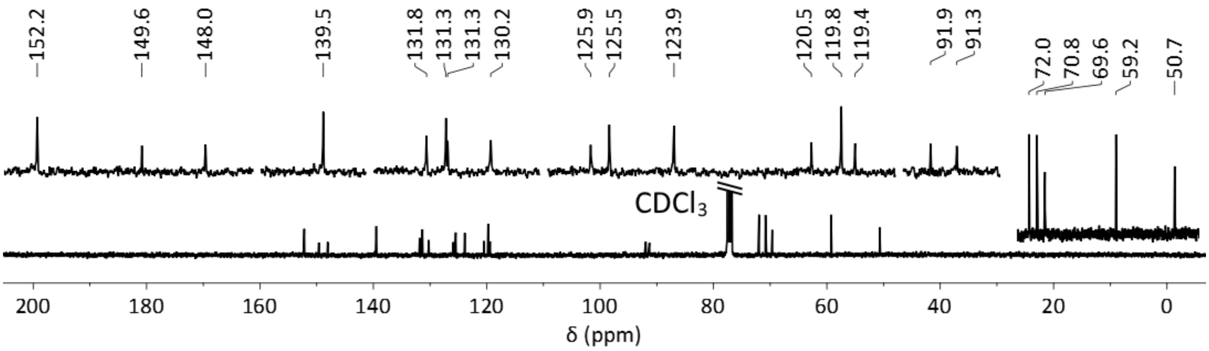

Figure S15 ^{13}C NMR spectrum (100 MHz, $CDCl_3$, 298 K) of **AD-N-DA**.

1.3.3. AD-NN-DA

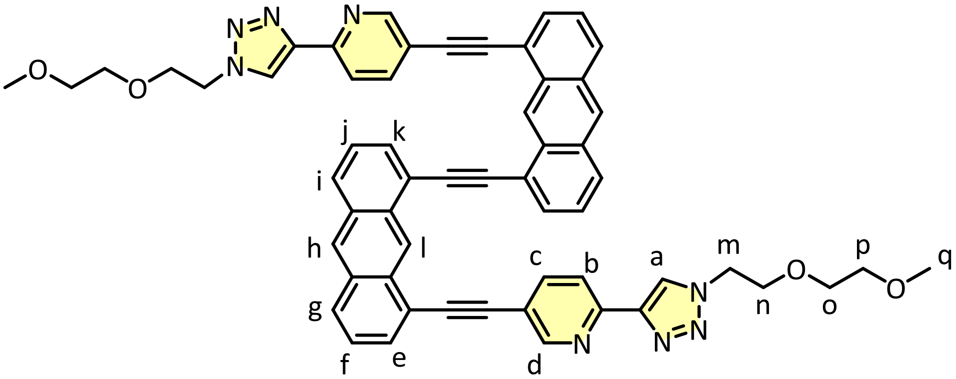

PART A

The combination of **AD-TMS** (175 mg, 0.508 mmol) and Na_2CO_3 (161 mg, 1.52 mmol) in methanol (40 mL) was stirred for 1 hour. After filtration, DCM (100 mL) was added and the organic phase was washed with water (100 mL). After removal of the solvent, the residue was combined with 1,8-diiodoanthracene (655 mg, 1.52 mmol), $[Pd_2(dba)_3]$ (12 mg, 0.013 mmol), triphenylphosphine (13 mg, 0.051 mmol) and CuI (10 mg, 0.051 mmol) in 1:1 THF/TEA (15 mL) under nitrogen, and heated at 40

°C overnight. The reaction mixture was added to DCM (100 mL) and EDTA/NH_4OH (100 mL) and stirred vigorously for 30 minutes. The organic layer was washed with water (100 mL) and the solvent removed under vacuum. Column chromatography on silica (DCM to remove unreacted 1,8-diiodoanthracne, then 1:3 acetone) gave a fraction which was predominantly 5-((8-iodoanthracen-1-yl)ethynyl)-2-(1-(2-(2-methoxyethoxy)ethyl)-1H-1,2,3-triazol-4-yl)pyridine (140 mg) which was used without additional purification in the next part.

PART B

Half of the residue from *PART A* (70 mg) was combined with [$Pd(PPh_3)_2Cl_2$] (4 mg, 0.006 mmol) and CuI (2 mg, 0.01 mmol) in 5:1 THF/TEA (25 mL) under nitrogen, and the sides washed down with ethynyl trimethysilane (48 mg, 0.49 mmol). The reaction was stirred at 40 °C under nitrogen overnight. The reaction mixture was added to DCM (100 mL) and EDTA/NH_4OH (100 mL) and stirred vigorously for 30 minutes. The organic layer was washed with water, and the solvent removed under vacuum. The residue was combined with the remaining residue from *PART A*, together with [$Pd_2(dba)_3$] (8 mg, 0.009 mmol) and triphenylphosphine (9 mg, 0.04 mmol), in 1.0 M [TBA]F (7 mL) under nitrogen. The reaction was stirred under nitrogen at 55 °C overnight. After adding DCM (50 mL) and washing with water (100 mL), the organic layer was removed under vacuum. The residue was sonicated in methanol with the solid collected through centrifugation. This solid was purified through column chromatography on silica (DCM to 9:1 DCM/methanol) to give the product as a yellow solid (9 mg, 0.010 mmol, 4%).

^{1}H NMR (400 MHz, $CDCl_3$, 298 K) *δ*: *integration given per 'half' of the ligand* 10.31 (1H, s, H_l), 4.15 (1H, s, H_d), 8.63 (1H, s, H_a), 8.48 (1H, s, H_h), 8.44 (1H, d, *J* = 7.1 Hz, H_k), 8.25 – 8.21 (2H, m, $H_{c,i}$), 8.17 (1H, d, *J* = 8.2 Hz, H_b), 8.00 (1H, d, *J* = 8.5 Hz, H_g), 7.75 (1H, d, *J* = 6 Hz, H_e), 7.55 (1H, t, *J* = 6.8 Hz, H_j), 7.46 (1H, t, *J* = 7.7 Hz, H_f), 4.63 – 4.60 (2H, m, H_m), 3.95 – 3.92 (2H, m, H_n), 3.64 – 3.62 (2H, m, H_o), 3.54 – 3.52 (2H, m, H_p), 3.35 (3H, s, H_q).

D ($\times 10^{-10}$ m^2 s^{-1}, 400 MHz, [D_6]DMSO, 298 K) = 1.69.

Poor solubility precluded the collection of a ^{13}C NMR spectrum.

HR ESI-MS (DCM/methanol) *m/z* = 919.3876 [MH]$^+$ (calc. for $C_{58}H_{47}N_8O_4$, 919.3720).

IR *v* (cm^{-1}) 3111, 3055, 2923, 2879, 1679, 1595, 1543, 1450, 1364, 1339.

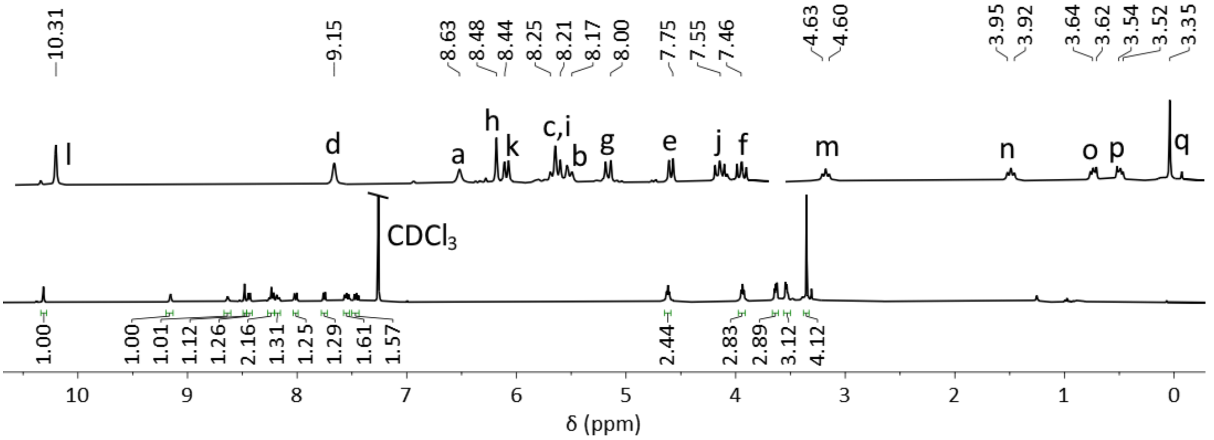

Figure S16 ^{1}H NMR spectrum (400 MHz, $CDCl_3$, 298 K) of **AD-NN-DA**.

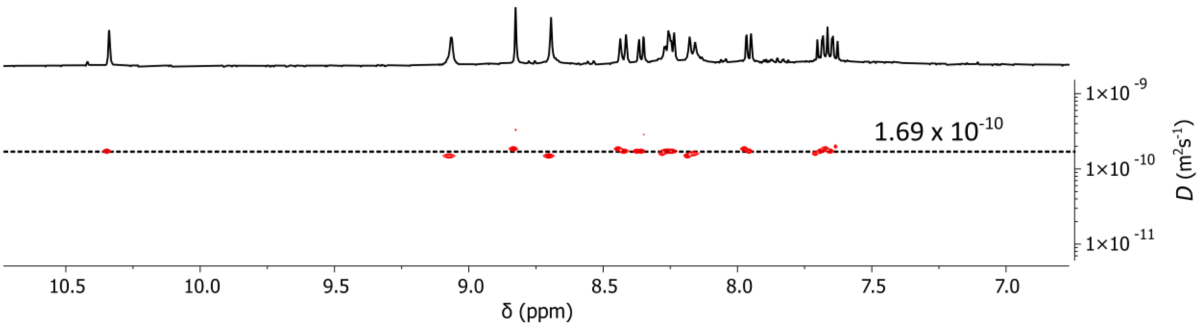

Figure S17 ^{1}H DOSY NMR spectrum (400 MHz, [D$_6$]DMSO, 298 K) of **AD-NN-DA**.

1.3.4. AD'-N'

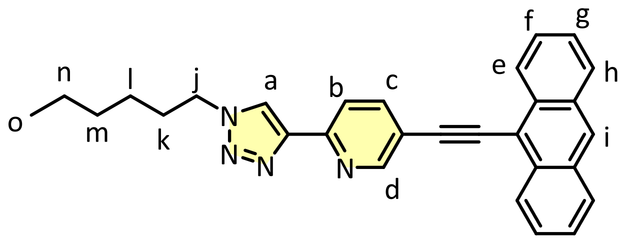

AD'-Br (101 mg, 0.653 mmol), dichlorobis(triphenylphospine)palladium(II) (11.5 mg, 0.0163 mmol), and copper iodide (6.20 mg, 0.0327 mmol) were added to a deoxygenated mixture of 9-ethynylanthracene[14] (132 mg, 0.653 mmol) in 3:1 dioxane/TEA (10 mL) against the flow of N_2. The mixture was then stirred at 80 °C for 17 hours under a nitrogen atmosphere. 0.1 M EDTA/NH_4OH (aq; 20 mL) and DCM (20 mL) were added, and the mixture was stirred vigorously for 30 min. The organic layer was then washed with distilled water (2 × 50 mL) and the volatiles removed under reduced pressure. The residue was purified via column chromatography on silica (9:1 PET/EtOAc to 1:1 PET/EtOAc) and the volatiles removed under reduced pressure to afford the product as a brown powder (50.0 mg, 0.111 mmol, 34%).

^{1}H NMR (400 MHz, $CDCl_3$, 298 K) δ: 8.94 (1H, br, H_d), 8.62 (2H, d, J = 8.8 Hz, H_e), 8.46 (1H, s, H_i), 8.27 (1H, d, J = 8.2 Hz, H_b), 8.20 (1H, s, H_a), 8.11 (1H, dd, J = 8.2 Hz, 2.1 Hz, H_c), 8.03 (2H, d, J = 8.4 Hz, H_h), 7.63 (2H, dd, J = 7.3 Hz, H_f), 7.53 (2H, dd, J = 7.3 Hz, H_g), 4.43 (2H, t, J = 7.3 Hz, H_j), 1.97 (2H, m, H_k), 1.35 (6H, m, H_l, H_m, H_n), 0.90 (3H, t, J = 6.8 Hz, H_o).

^{13}C NMR (100 MHz, $CDCl_3$, 298 K) δ: 151.9, 149.4, 148.0, 139.4, 132.8, 131.3, 128.9, 128.6, 127.1, 126.7, 125.9, 122.4, 119.8, 119.7, 116.5, 97.6, 90.2, 50.7, 31.3, 30.4, 26.3, 22.6, 14.1.

HR ESI-MS (MeOH) m/z = 431.2261 $[MH]^+$ (calc. for $C_{29}H_{26}N_4$, 431.2263)

IR ν (cm^{-1}) 3132, 3052, 2953, 2927, 2857, 2195, 1717, 1673, 1594, 1457.

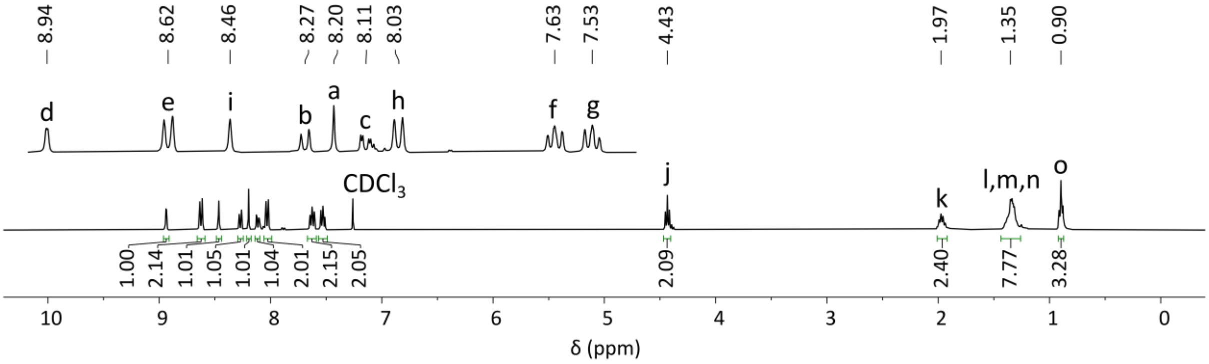

Figure S18 ^{1}H NMR spectrum (400 MHz, $CDCl_3$, 298 K) of **AD'-N'**.

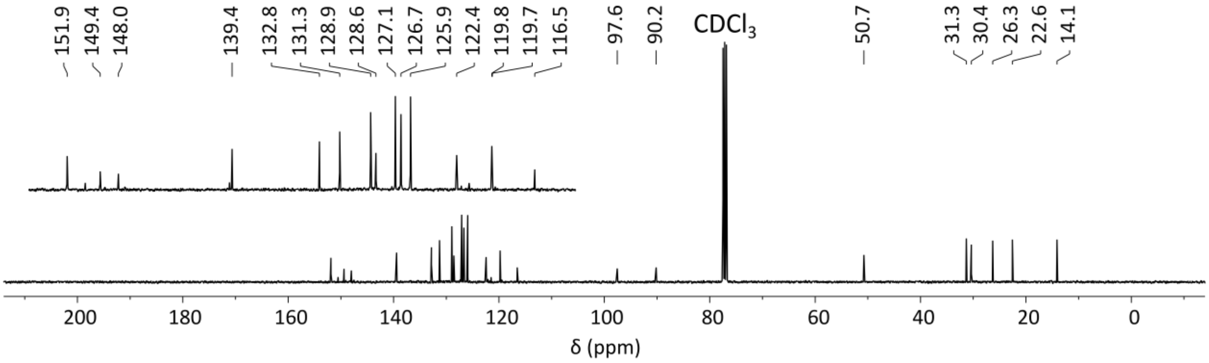

Figure S19 ^{13}C NMR spectrum (100 MHz, $CDCl_3$, 298 K) of **AD'-N'.**

1.3.5. AD--N'

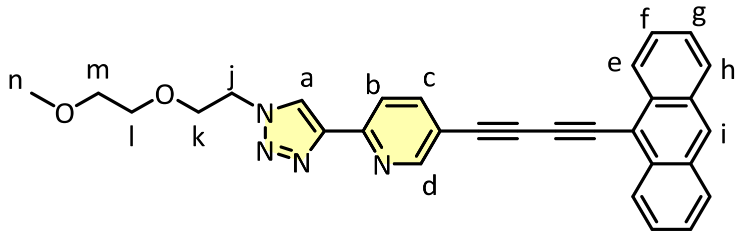

The combination of 2-(2-methoxyethoxy)ethyl p-toluenesulfonate[2] (175 mg, 0.638 mmol) and NaN_3 (50 mg, 0.77 mmol) in DMF (10 mL) was heated at 110 °C for 1.5 hours. This was added to **B** (310 mg, 0.766 mmol), sodium carbonate (122 mg, 1.15 mmol), sodium ascorbate (63 mg, 0.32 mmol) and $Cu(SO_4)_2{\cdot}5H_2O$ (80 mg, 0.32 mmol) in DMF (10 mL) and the reaction was stirred at room temperature overnight under nitrogen. After adding DCM (100 mL) and 0.1 M aqueous EDTA/NH_4OH (0.1 M, 100 mL) and stirring vigorously for 30 minutes, the organic layer was removed under vacuum, the residue taken up again in DCM (100 mL), washed with water (100 mL), and removed under vacuum. Purification by column chromatography on silica (DCM to 1:4 acetone/DCM) gave the product as a yellow solid (223 mg, 0.472 mmol, 74%).

^{1}H NMR (400 MHz, $CDCl_3$, 298 K) δ: 8.81 (1H, br, H_d), 8.57 (2H, d, J = 8.8 Hz, H_e), 8.48 (1H, s, H_i), 8.39 (1H, br, H_a), 8.20 (1H, d, J = 8.2 Hz, H_b), 8.03 (2H, d, J = 8.4 Hz, H_h), 7.97 (1H, dd, J = 8.2 Hz, 2.1 Hz, H_c), 7.65-7.61 (2H, m, H_f), 7.55-7.51 (2H, m, H_g), 4.64 (2H, t, J = 7.3 Hz, H_j), 3.94 (2H, t, J = 5.0 Hz, H_k), 3.65-3.63 (2H, m, H_l), 3.55-3.53 (2H, m, H_m), 3.38 (3H, s, H_n).

^{13}C NMR (100 MHz, $CDCl_3$, 298 K) δ: 152.3, 149.5, 147.2, 140.5, 134.2, 131.1, 129.3, 129.0, 127.4, 126.4, 126.0, 124.2, 119.7, 118.2, 115.1, 84.2, 81.0, 80.6, 78.2, 71.8, 70.7, 69.4, 59.1, 50.6.

HR ESI-MS (MeOH) m/z = 473.1982 [MH]$^+$ (calc. for $C_{30}H_{24}N_4O_2$, 473.1978); IR ν (cm^{-1}) 3142, 3066, 2981, 2971, 2930, 2890, 2200, 1723, 1670, 1594, 1460, 1380, 1363.

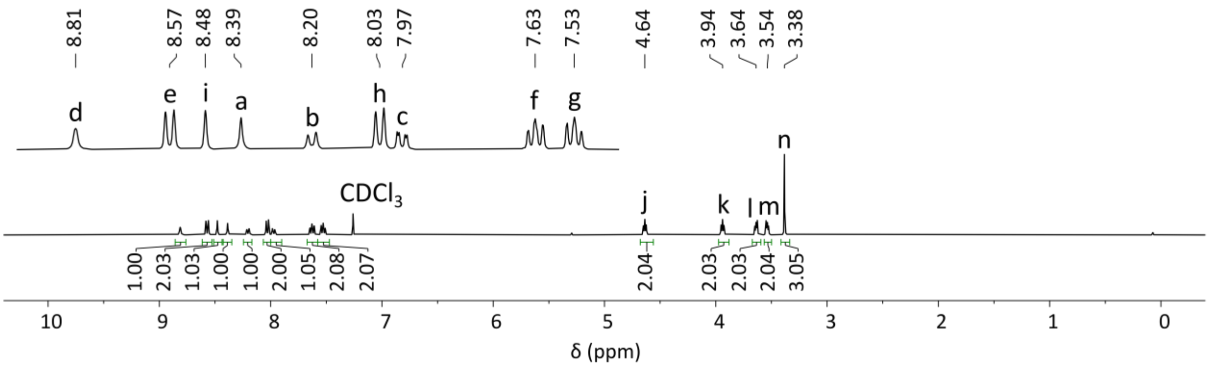

Figure S20 ^1H NMR spectrum (400 MHz, $CDCl_3$, 298 K) of **AD--N'**.

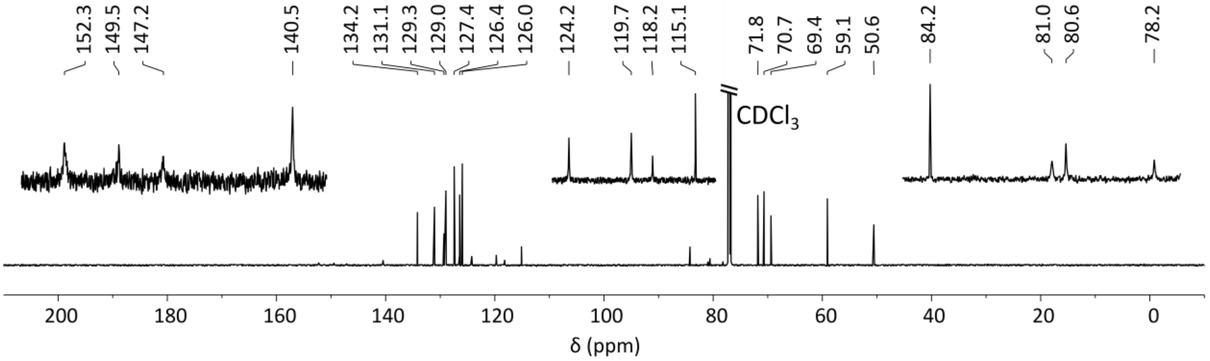

Figure S21 ^{13}C NMR spectrum (100 MHz, $CDCl_3$, 298 K) of **AD--N'**.

1.4. DA' ligands

1.4.1. DA'-N

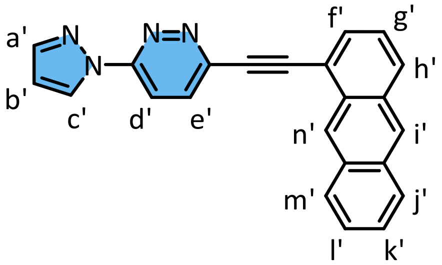

The combination of 4-(1-anthracenyl)-2-methyl-3-butyn-2-ol[1] (220 mg, 0.845 mmol) and finely ground KOH (142 mg, 2.54 mmol) in toluene (40 mL) was heated at 120 °C for 30 minutes. After filtration, the solvent was removed under vacuum. The residue was combined with **DA'-Br** (76 mg, 0.34 mmol), [$Pd(PPh_3)_2Cl_2$] (12 mg, 0.017 mmol) and CuI (6 mg, 0.04 mmol) in 1:1 dioxane/TEA (15 mL) under nitrogen, and heated at 80 °C overnight. The reaction mixture was added to DCM (100 mL) and EDTA/NH_4OH (100 mL) and stirred vigorously for 30 minutes. The organic layer was washed with water (100 mL) and the solvent removed under vacuum. Purification by column chromatography on silica (1:4 to 1:1 to 3:1 pet ether/DCM) gave the product as a yellow solid (102 mg, 0.295 mmol, 87%).

^{1}H NMR (400 MHz, $CDCl_3$, 298 K) δ: 9.03 (1H, s, $H_{n'}$), 8.87 (1H, d, J = 2.7 Hz, $H_{c'}$), 8.48 (1H, s, $H_{i'}$), 8.26 (1H, d, J = 9.1 Hz, $H_{d'}$), 8.16 – 8.13 (1H, m, $H_{m'}$), 8.10 (1H, d, J = 9.2 Hz, $H_{h'}$), 8.05 – 8.02 (1H, m, $H_{j'}$), 7.93 – 7.85 (2H, m, $H_{e',f'}$), 7.85 (1H, d, J = 1.7 Hz, $H_{a'}$), 7.56 – 7.46 (3H, m, $H_{g',k'l'}$), 6.59 (1H, dd, J = 2.7 Hz, 1.7 Hz, $H_{b'}$).

D ($\times 10^{-10}$ m^2 s^{-1}, 400 MHz, [D_6]DMSO, 298 K) = 2.2.

^{13}C NMR (100 MHz, $CDCl_3$, 298 K) δ: 152.5, 146.6, 143.5, 132.3, 132.2, 132.1, 131.8, 131.0, 130.7, 130.6, 128.6, 128.0, 127.8, 127.2, 126.2, 126.1, 124.9, 124.5, 119.2, 116.8, 109.2, 92.6, 90.6.

HR ESI-MS (DCM/methanol) m/z = 347.1294 $[MH]^+$ (calc. for $C_{23}H_{15}N_4$, 347.1297)

IR v (cm^{-1}) 3149, 3051, 2925, 2853, 2780, 2211, 1729, 1673, 1612, 1574, 1557, 1524, 1458, 1438, 1420, 1393.

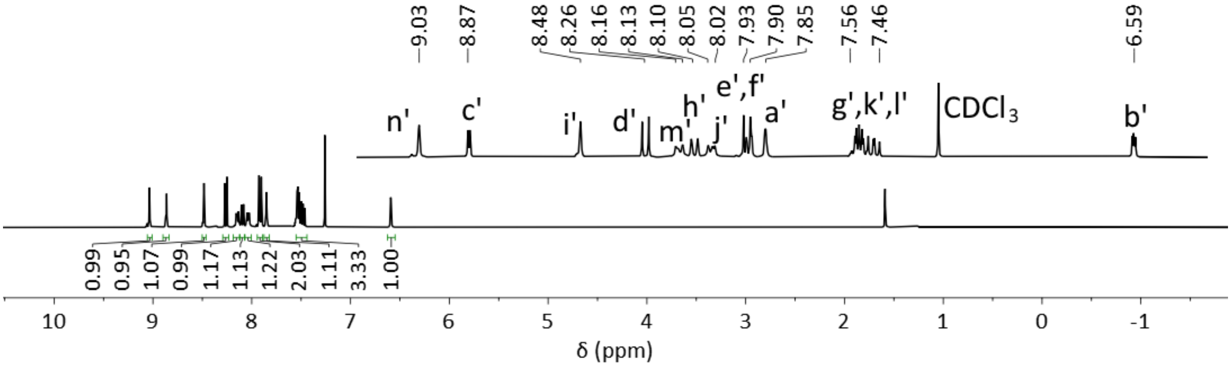

Figure S22 ^{1}H NMR spectrum (400 MHz, $CDCl_3$, 298 K) of **DA'-N**.

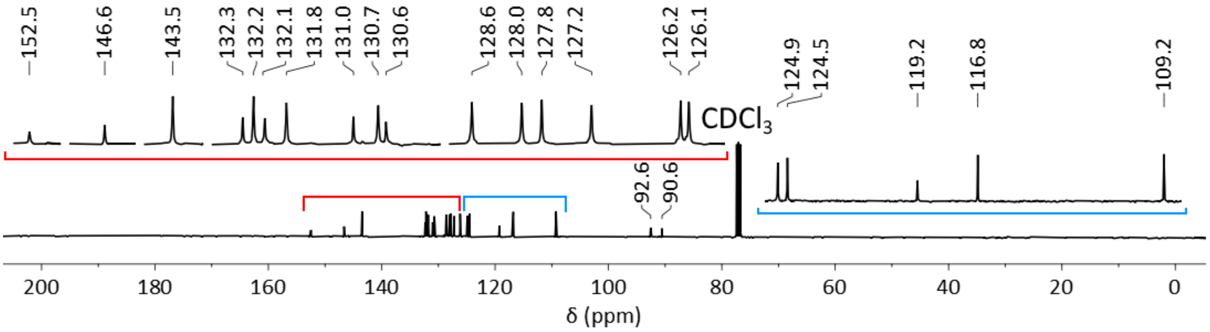

Figure S23 ^{13}C NMR spectrum (100 MHz, $CDCl_3$, 298 K) of **DA'-N**.

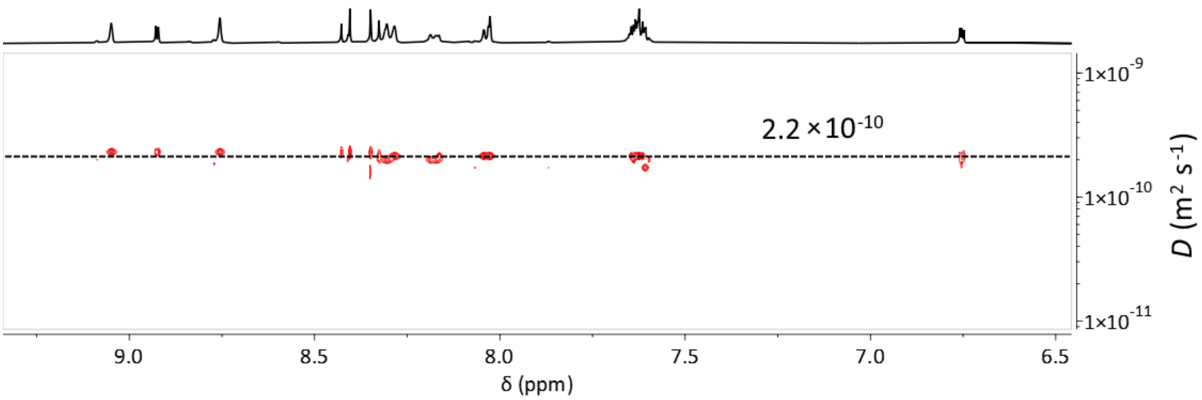

Figure S24 ^{1}H DOSY NMR spectrum (400 MHz, [D_6]DMSO, 298 K) of **DA'-N**.

1.4.2. DA'--N

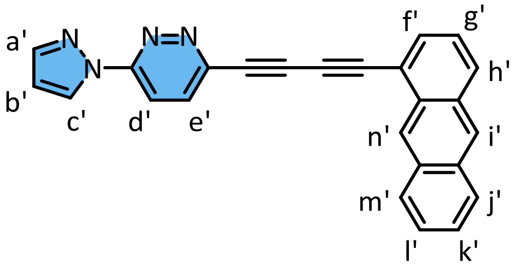

The combination of 1-ethynylanthracene[1] (290 mg, 1.43 mmol), 1,2-*cis*-dichloroethene (556 mg, 5.74 mmol), butylamine (315 mg, 4.30 mmol), CuI (54 mg, 0.29 mmol), $[Pd_2(dba)_3]$ (33 mg, 0.036 mmol) and triphenylphosphine (38 mg, 0.14 mmol) was stirred in toluene (10 mL) under a nitrogen atmosphere in the absence of light for 2 days. The mixture was washed with water (20 mL) and the organic solvent removed under vacuum. Purification by column chromatography on silica (pet ether to 1:1 pet ether/DCM) gave (Z)-1-(4-chlorobut-3-en-1-yn-1-yl)anthracene as a yellow solid with 85% purity (169 mg, 0.641 mmol, 45%). This was combined with tetrabutylammonium fluoride (505 mg, 1.93 mmol, administered as 1.93 mL from a 1.0 M solution) in THF (5 mL) was stirred in the absence of light overnight. After adding DCM (20 mL) and washing with water (30 mL), the organic solvent was removed under vacuum. The residue was combined with **DA'-I** (349 mg, 1.29 mmol), CuI (12 mg, 0.064 mmol), $[Pd_2(dba)_3]$ (15 mg, 0.016 mmol) and triphenylphosphine (17 mg, 0.064 mmol) in 1:1 THF/TEA (10 mL) and stirred in the absence of light under a nitrogen atmosphere for 2 days. After adding DCM (100 mL) and 0.1 M EDTA/NH_4OH (100 mL) and stirring vigorously for 30 minutes, the organic layer was washed with water. After removal of the solvent under vacuum, the residue was purified using column chromatography on silica (DCM to 1:99 acetone/DCM) to give the product at approximately 90% purity. The impure solid was sonicated repeatedly in diethyl ether, followed by centrifugation and discarding the filtrate to give the pure product (120 mg, 0.322 mmol, 50%).

^{1}H NMR (400 MHz, [D$_6$]DMSO, 298 K) δ: 8.91 – 8.91 (2H, m, $H_{c,n}$), 8.75 (1H, s, H_i), 8.32 – 8.24 (4H, m, $H_{d,e,h,m}$), 8.17 – 8.15 (1H, m, H_j), 8.07 (1H, d, J = 7.0 Hz, H_f), 8.02 (1H, d, J = 1.7 Hz, H_a), 7.63 – 7.57 (3H, m, $H_{g,k,l}$), 6.74 (1H, t, J = 2.7 Hz, H_b).

D ($\times 10^{-10}$ m^2 s^{-1}, 400 MHz, [D$_6$]DMSO, 298 K) = 2.01.

Poor solubility precluded the collection of a ^{13}C NMR spectrum.

HR ESI-MS (DCM/methanol) m/z = 371.1299 [MH]$^+$ (calc. for $C_{25}H_{15}N_4$, 371.1297)

IR ν (cm^{-1}) 3151, 3050, 2924, 2852, 2210, 1612, 1573, 1555, 1525, 1439, 1422, 1393, 1305.

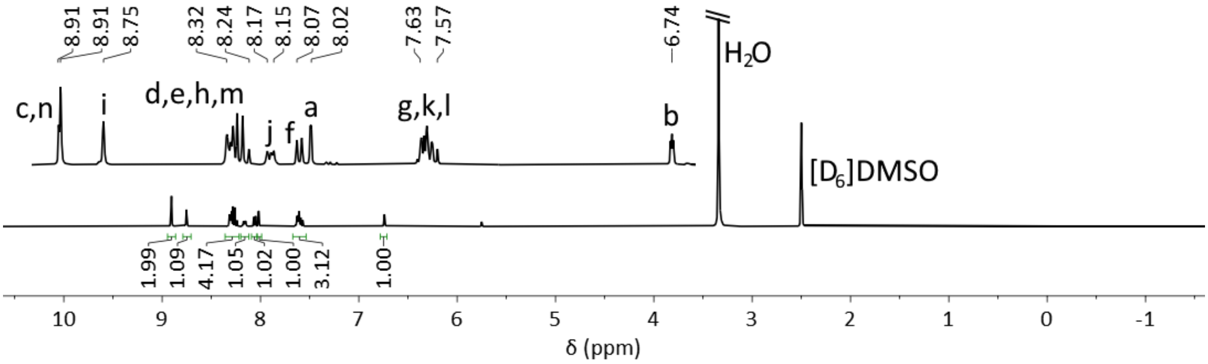

Figure S25 ^{1}H NMR spectrum (400 MHz, [D$_6$]DMSO, 298 K) of **DA'--N**.

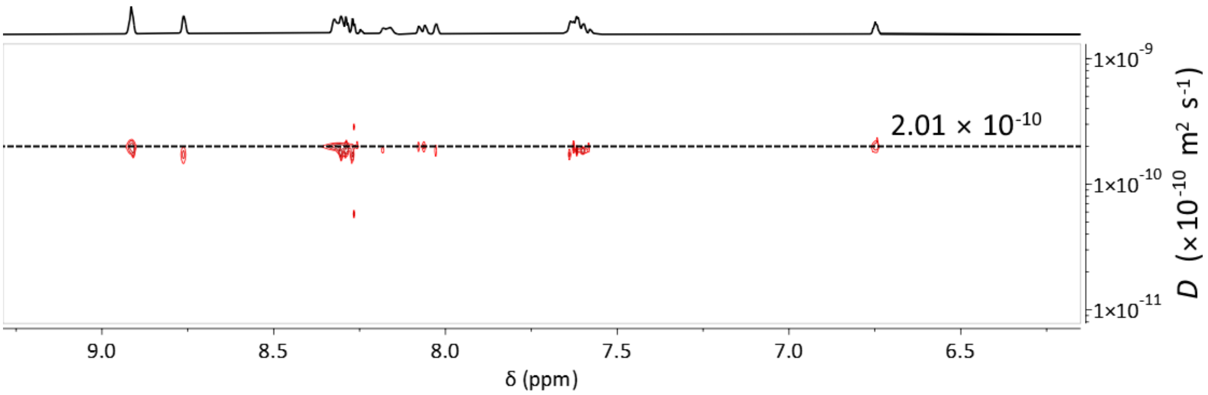

Figure S26 ^{1}H DOSY NMR spectrum (400 MHz, [D$_6$]DMSO, 298 K) of **DA'--N**.

1.4.3. DA'-N-AD'

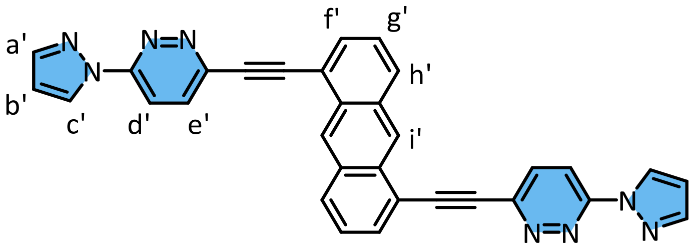

The combination of **DA'-TMS** (120 mg, 0.497 mmol) and Na_2CO_3 (33 mg, 0.31 mmol) was stirred in methanol (20 mL) for 1 hour. After adding DCM (100 mL) and washing with water (100 mL), the organic layer was removed under vacuum. The residue was combined with 1,5-diiodoanthracene (89 mg, 0.21 mmol), [Pd_2(dba)$_3$] (5 mg, 0.005 mmol), triphenylphosphine (5 mg, 0.02 mmol) and CuI (4 mg, 0.02 mmol) in 1:1 THF/TEA (15 mL) under nitrogen, and heated at 40 °C overnight. The reaction mixture was added to DCM (100 mL) and EDTA/NH_4OH (100 mL) and stirred vigorously for 30 minutes. The organic layer was washed with water (100 mL) and the solvent removed under vacuum. Purification by column chromatography on silica (DCM to 1:10 acetone/DCM to 1:3 acetone/DCM to 1:19 methanol/DCM) gave the product as a yellow solid (35 mg, 0.067 mmol, 33%).

^{1}H NMR (400 MHz, [D$_6$]DMSO, 298 K) δ: *integration given per 'half' of the ligand* 9.18 (1H, s, $H_{c'}$), 8.92 (1H, s, $H_{i'}$), 8.50 (1H, d, J = 8.9 Hz, $H_{h'}$), 8.44 (1H, d, J = 8.6 Hz, $H_{d'}$), 8.35 (1H, d, J = 8.6 Hz, $H_{e'}$), 8.11 (1H, d, J = 7.0 Hz, $H_{f'}$), 8.03 (1H, br, $H_{a'}$), 7.72 (1H, t, J = 7.3 Hz, $H_{g'}$), 6.75 (1H, br, $H_{b'}$).

D ($\times$ 10^{-10} m^2 s^{-1}, 400 MHz, [D$_6$]DMSO, 298 K) = 2.16.

Poor solubility precluded the collection of a ^{13}C NMR spectrum.

HR ESI-MS (DCM/methanol) *m/z* = 515.1725 [MH]$^+$ (calc. for $C_{32}H_{19}N_8$, 515.1733)

IR *v* (cm^{-1}) 3138, 3121, 3071, 2881, 2210, 1673, 1576, 1557, 1525, 1461, 1438, 1420, 1408, 1392.

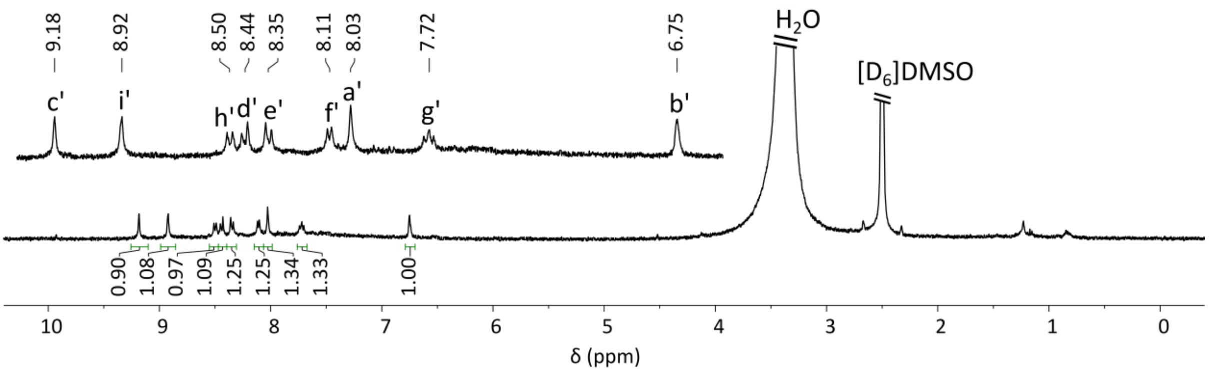

Figure S27 ^1H NMR spectrum (400 MHz, [D$_6$]DMSO, 298 K) of **DA'-N-AD'**.

1.4.4. DA'-NN-AD'

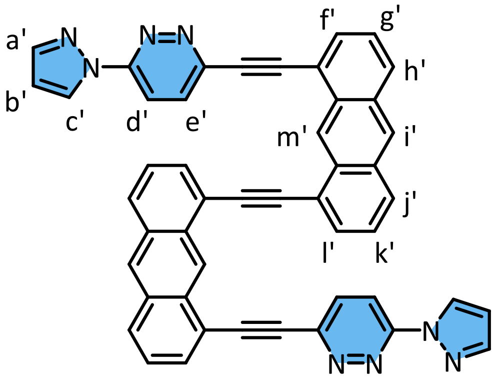

^1H NMR (400 MHz, [D$_6$]DMSO, 298 K) δ: *integration given per 'half' of the ligand* 9.59 (1H, s, $H_{m'}$), 8.50 (1H, d, J = 2.5 Hz, $H_{c'}$), 8.18 (1H, s, $H_{i'}$), 8.12-8.07 (3H, m, $H_{a',j',l'}$), 7.84 (1H, d, J = 6.8 Hz, $H_{f'}$), 7.72-7.68 (2H, m, $H_{h',k'}$), 7.45 (1H, t, J = 7.8 Hz, $H_{g'}$), 7.06 (1H, d, J = 9.0 Hz, $H_{e'}$), 6.99 (1H, d, J = 9.0 Hz, $H_{d'}$), 6.80 (1H, s, $H_{b'}$).

Poor solubility precluded the collection of a ^1H DOSY NMR spectrum.

Poor solubility precluded the collection of a ^{13}C NMR spectrum.

HR ESI-MS (DCM/methanol) *m/z* = 715.2361 [MH]$^+$ (calc. for $C_{48}H_{27}N_8$, 715.2358)

IR *v* (cm^{-1}) 3344, 3090, 3070, 2925, 2853, 1672, 1558, 1524, 1438, 1417, 1393, 1318.

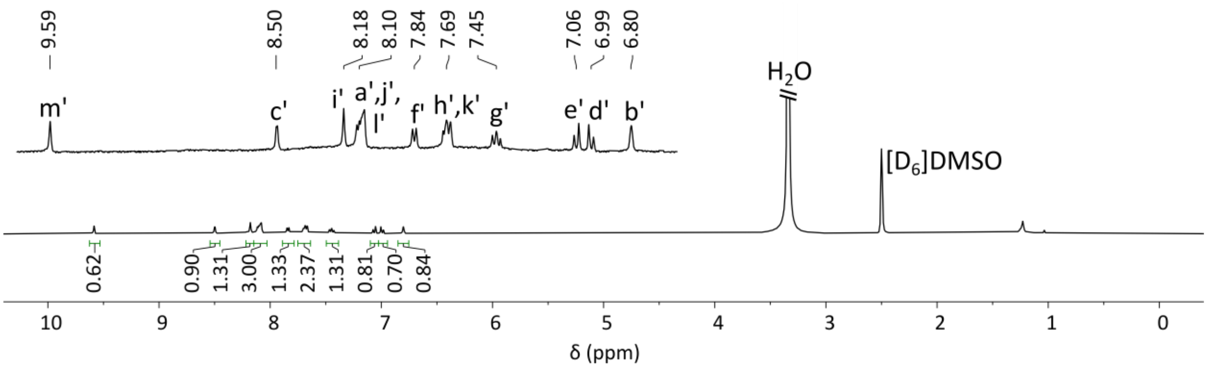

Figure S28 ^{1}H NMR spectrum (400 MHz, [D$_6$]DMSO, 298 K) of **DA'-NN-AD'**.

1.4.5. DA'-N'

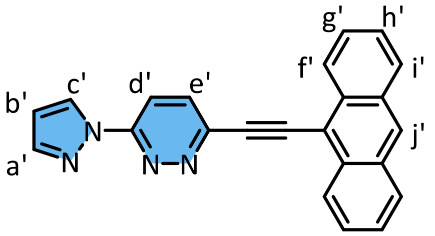

DA'-Br (416 mg, 1.85 mmol), dichlorobis(triphenylphospine)palladium(II) (114 mg, 0.162 mmol), and copper iodide (32.0 mg, 0.168 mmol) were added to a deoxygenated mixture of 9-ethynylanthracene[14] (340 mg, 1.68 mmol) in 3:1 dioxane/TEA (10 mL) against the flow of N_2. The mixture was then stirred at 80 °C for 17 hours under a nitrogen atmosphere. 0.1 M EDTA/NH_4OH (aq; 20 mL) and DCM (20 mL) were added, and the mixture was stirred vigorously for 30 min. The organic layer was then washed with distilled water (2 × 50 mL) and the volatiles removed under reduced pressure. The residue was purified via column chromatography on silica (1:1 PET/DCM to DCM) and the volatiles removed under reduced pressure to afford the product as a light-yellow powder (210 mg, 0.598 mmol, 36%).

^{1}H NMR (400 MHz, $CDCl_3$, 298 K) δ: 8.89 (1H, d, J = 2.6 Hz, $H_{c'}$), 8.74 (2H, d, J = 8.8 Hz, $H_{f'}$), 8.57 (1H, s, $H_{j'}$), 8.31 (1H, d, J = 9.0 Hz, $H_{d'}$), 8.08 (2H, d, J = 8.8 Hz, $H_{i'}$), 8.01 (1H, d, J = 9.0 Hz, $H_{e'}$), 7.88 (1H, br s, $H_{a'}$), 7.70-7.66 (2H, m, $H_{g'}$), 7.60-7.56 (2H, m, $H_{h'}$), 6.62 (1H, dd, J = 2.6 Hz, 1.7 Hz, $H_{b'}$);

^{13}C NMR (100 MHz, $CDCl_3$, 298 K) δ: 152.5, 146.9, 143.6, 133.3, 132.1, 131.2, 129.7, 129.0, 127.9, 127.5, 126.6, 126.1, 116.9, 115.2, 109.4, 96.7, 91.3.

HR ESI-MS (MeOH) m/z = 347.1299 [MH]$^+$ (calc. for $C_{23}H_{14}N_4$, 347.1297)

IR v (cm^{-1}) 3134, 3119, 3071, 3048, 3023, 2206, 1622, 1575, 1561, 1524, 1439.

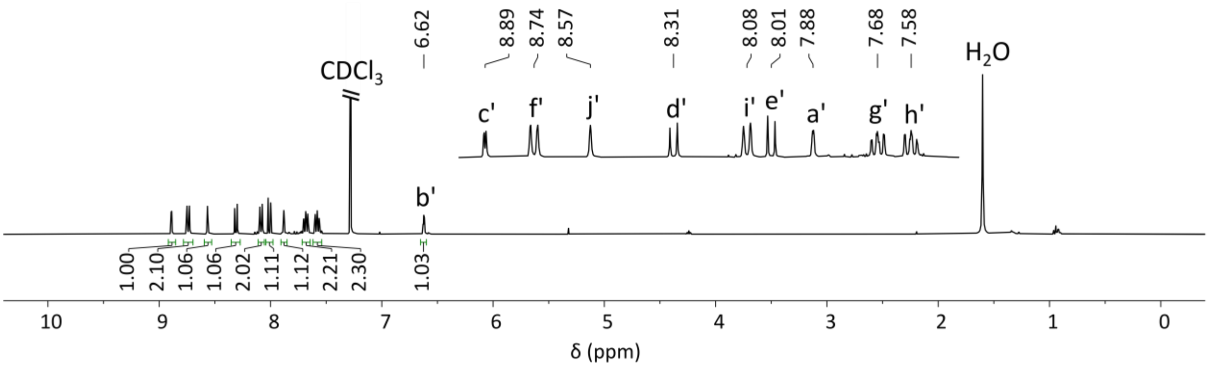

Figure S29 ^{1}H NMR spectrum (400 MHz, $CDCl_3$, 298 K) of **DA'-N'.**

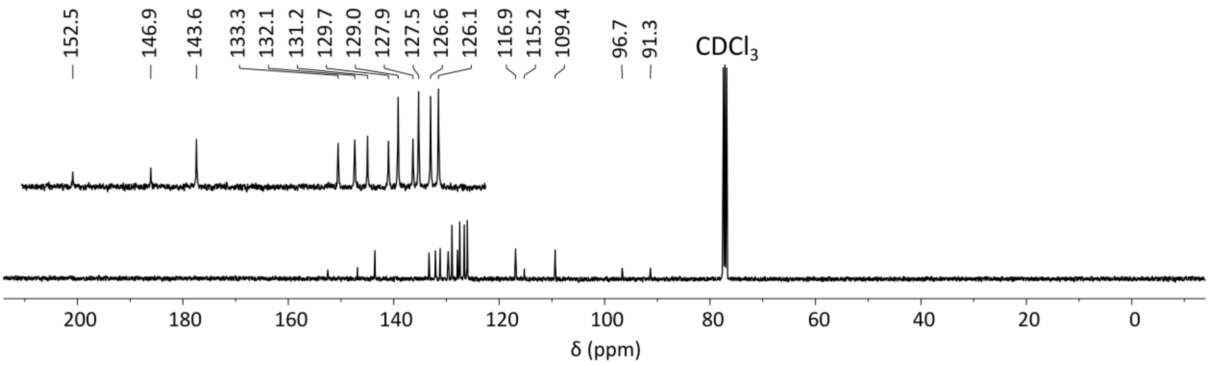

Figure S30 ^{13}C NMR spectrum (100 MHz, $CDCl_3$, 298 K) of **DA'-N'.**

1.4.6. DA'--N'

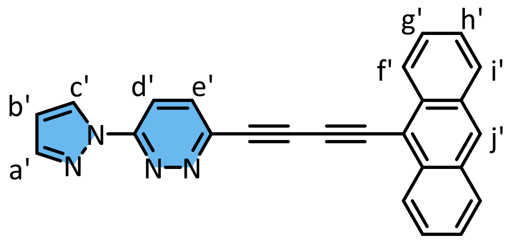

DA'-I (200 mg, 0.735 mmol), 9-(1,3-Butadiyn-1-yl)anthracene[5] (200 mg, 0.882 mmol), tris(dibenzylideneacetone)dipalladium(0) (34 mg, 0.037 mmol), triphenylphosphine (39 mg, 0.15 mmol), copper(I) iodide (14 mg, 0.074 mmol) were added to a deoxygenated solution of 3:1 dioxane/TEA (10 mL) against the flow of N_2. This was stirred at room temperature for 18 hours under a nitrogen atmosphere with the exclusion of light. After adding DCM (50 mL) and 0.1 M aqueous EDTA/NH_4OH (50 mL) and stirring vigorously for 30 minutes, the organic layer was removed under vacuum, the residue taken up again in DCM (100 mL), washed with water (100 mL), and removed under vacuum. Purification by column chromatography on silica (1:1 PET/DCM to DCM) gave the product as a yellow powder (130 mg, 0.35 mmol, 48%).

^{1}H NMR (400 MHz, $CDCl_3$, 298 K) δ: 8.83 (1H, d, J = 2.5 Hz, $H_{c'}$), 8.57 (2H, d, J = 8.8 Hz, $H_{f'}$), 8.51 (1H, s, $H_{j'}$), 8.22 (1H, d, J = 9.0 Hz, $H_{d'}$), 8.04 (2H, d, J = 8.6 Hz, $H_{i'}$), 7.84 (1H, d, J = 1.2 Hz, $H_{a'}$), 7.82 (1H, d, J = 9.0 Hz, $H_{e'}$), 7.65 (2H, t, J = 7.3 Hz, $H_{g'}$), 7.54 (2H, t, J = 7.3 Hz, $H_{h'}$), 6.58 (1H, dd, J = 2.5 Hz, 1.2 Hz, 1H, $H_{b'}$).

^{13}C NMR (100 MHz, $CDCl_3$, 298 K) δ: 145.9, 143.8, 134.5, 132.8, 131.2, 130.0, 129.2, 128.1, 127.8, 126.5, 126.2, 116.8, 116.2, 114.6, 109.6, 83.9, 82.2, 79.8, 79.1.

HR ESI-MS (MeOH) m/z = 371.1295 $[MH]^+$ (calc. for $C_{25}H_{14}N_4$, 371.1297).

IR ν (cm^{-1}) 3149, 3054, 2957, 2926, 2868, 2783, 2202, 1719, 1668, 1621, 1573, 1556, 1525, 1439, 1408, 1393.

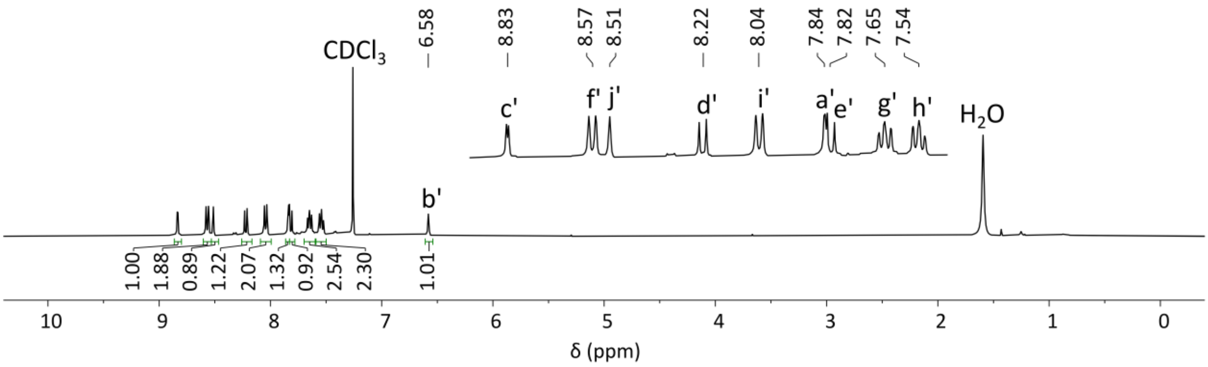

Figure S31 ^{1}H NMR spectrum (400 MHz, $CDCl_3$, 298 K) of **DA'--N'.**

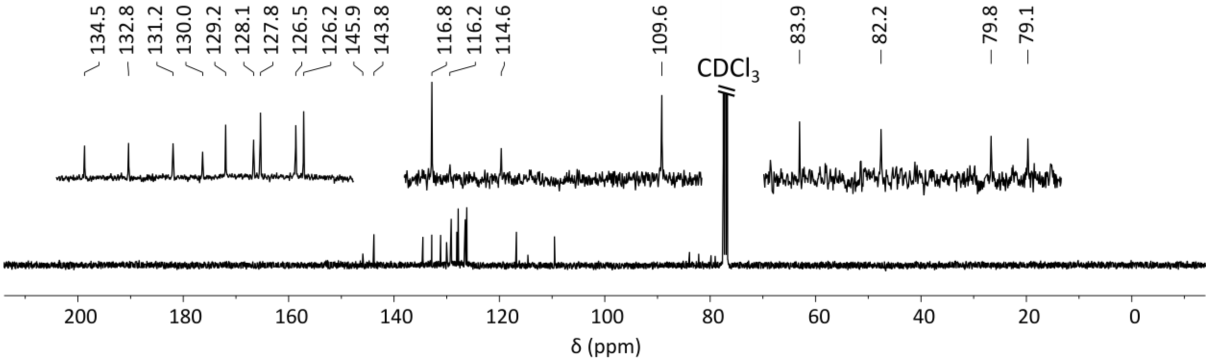

Figure S32 ^{13}C NMR spectrum (100 MHz, $CDCl_3$, 298 K) of **DA'--N'.**

1.5. DD ligands

1.5.1. DD-NN-DD

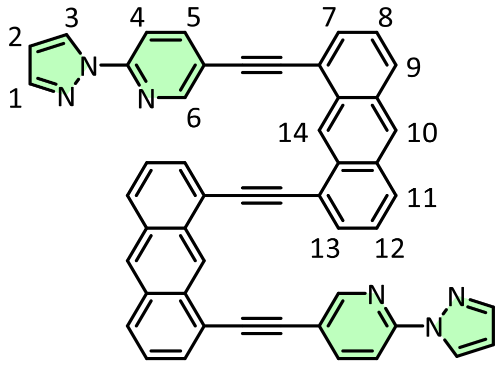

PART A

The combination of **DD-TMS** (120 mg, 0.497 mmol) and Na_2CO_3 (158 mg, 1.49 mmol) in methanol (40 mL) was stirred for 1 hour. After filtration, DCM (100 mL) was added and the organic phase was washed with water (100 mL). After removal of the solvent, the residue was combined with 1,8-diiodoanthracene (641 mg, 1.49 mmol), [Pd_2(dba)$_3$] (11 mg, 0.012 mmol), triphenylphosphine (13 mg, 0.050 mmol) and CuI (9 mg, 0.05 mmol) in 1:1 THF/TEA (15 mL) under nitrogen, and heated at 40°C overnight. The reaction mixture was added to DCM (100 mL) and EDTA/NH_4OH (100 mL) and stirred vigorously for 30 minutes. The organic layer was washed with water (100 mL) and the solvent removed under vacuum. Column chromatography on silica (DCM to remove unreacted 1,8-diiodoanthracne, then 1:3 acetone) gave a fraction which was predominantly 5-((8-iodoanthracen-1-yl)ethynyl)-2-(1-(2-(2-methoxyethoxy)ethyl)-1H-1,2,3-triazol-4-yl)pyridine (190 mg) which was used without additional purification in the next part.

PART B

Half of the residue from *PART A* (85 mg) was combined with [Pd(PPh_3)$_2$$Cl_2$] (6 mg, 0.009 mmol) and CuI (3 mg, 0.02 mmol) in 5:1 THF/TEA (25 mL) under nitrogen, and the sides washed down with ethynyl trimethysilane (71 mg, 0.72 mmol). The reaction was stirred at 40 °C under nitrogen overnight. The reaction mixture was added to DCM (100 mL) and EDTA/NH_4OH (100 mL) and stirred vigorously for 30 minutes. The organic layer was washed with water, and the solvent removed under vacuum. The residue was combined with the remaining residue from *PART A*, together with [Pd_2(dba)$_3$] (8 mg, 0.009 mmol) and triphenylphosphine (9 mg, 0.04 mmol), in 1.0 M [TBA]F (7 mL) under nitrogen. The reaction was stirred under nitrogen at 55 °C overnight. After adding DCM (50 mL) and washing with water (100 mL), the organic layer was removed under vacuum. The residue was sonicated in methanol with the solid collected through centrifugation. This solid was purified through column chromatography on silica (DCM to 9:1 DCM/methanol) to give the product as a yellow solid (54 mg, 0.076 mmol, 31%).

^{1}H NMR (400 MHz, $CDCl_3$, 298 K) δ: *integration given per 'half' of the ligand* 9.70 (1H, s, H_{14}), 8.31 (1H, d, J = 2.8 Hz, H_3), 7.99 – 7.96 (3H, m, $H_{10,11,13}$), 7.84 (1H, d, J = 2.1 Hz, H_1), 7.74 (1H, d, J = 2.1 Hz, H_6), 7.66 (1H, d, J = 6.8 Hz, H_7), 7.59 – 7.54 (2H, m, $H_{9,12}$), 6.96 (1H, dd, J = 8.4 Hz, 2.2 Hz, H_5), 6.79 (1H, d, J = 8.4 Hz, H_4), 6.56 (1H, t, J = 1.7 Hz, H_2).

D ($\times 10^{-10}$ m^2 s^{-1}, 400 MHz, [D_6]DMSO, 298 K) = 1.70.

Poor solubility precluded the collection of a ^{13}C NMR spectrum.

HR ESI-MS (DCM/methanol) *m/z* = 713.2451 $[MH]^+$ (calc. for $C_{50}H_{29}N_6$, 713.2454)

IR *v* (cm^{-1}) 3150, 3053, 2926, 1775, 1673, 1591, 1567, 1522, 1485, 1429, 1393, 1334.

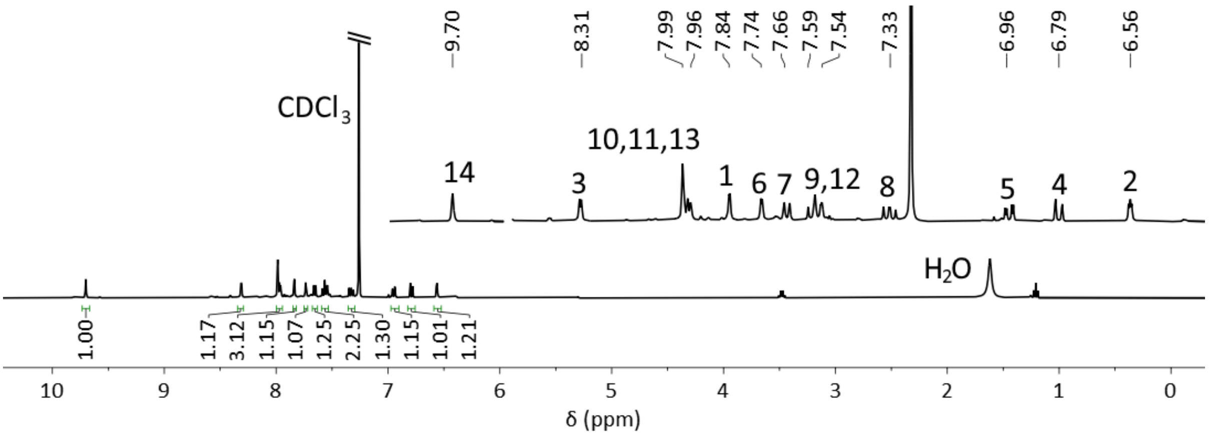

Figure S33 ^1H NMR spectrum (400 MHz, $CDCl_3$, 298 K) of **DD-NN-DD**.

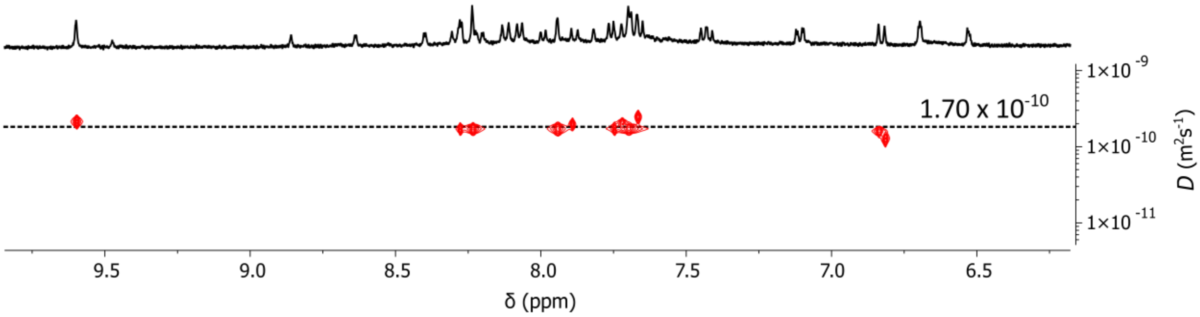

Figure S34 ^1H DOSY NMR spectrum (400 MHz, $[D_6]DMSO$, 298 K) of **DD-NN-DD**.

1.5.2. DD--N'

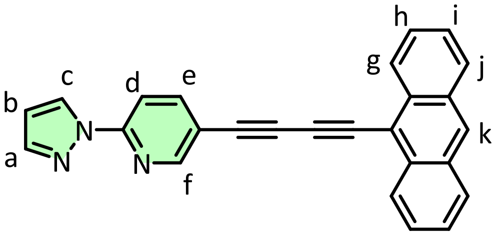

A solution of **DD-TMS** (110 mg, 0.456 mmol) and Na_2CO_3 (97.1 mg, 0.912 mmol) in MeOH was stirred at room temperature for one hour, filtered through cotton wool and volatiles removed under reduced pressure. The resulting residue was added in crude form to a mixture of **C** (150 mg, 0.456 mmol), copper(I) iodide (8.68 mg, 0.0456 mmol) and triethylamine (92.6 mg, 0.912 mmol) in deoxygenated acetonitrile (5 mL). This was stirred at room temperature for 21 hours under a nitrogen atmosphere with the exclusion of light. After adding DCM (50 mL) and 0.1 M aqueous EDTA/NH_4OH (50 mL) and stirring vigorously for 30 minutes, the organic layer was removed under vacuum, the residue taken up again in DCM (100 mL), washed with water (100 mL), and removed under vacuum. Purification by column chromatography on silica (1:1 PET/DCM to DCM) gave the product as a yellow solid (21 mg, 0.057 mmol, 21%).

^{1}H NMR (400 MHz, $CDCl_3$, 298 K) δ: 8.65 (1H, br, H_f), 8.59-8.57 (3H, m, H_c, H_g), 8.50 (1H, s, H_k), 8.03 (4H, m, H_d, H_e, H_j), 7.78 (1H, d, J = 1.3 Hz, H_a), 7.64 (2H, dd, J = 7.3 Hz, H_h), 7.54 (2H, dd, J = 7.3 Hz, H_i), 6.51 (1H, dd, J = 2.5 Hz, 1.3 Hz, H_b).

^{13}C NMR (100 MHz, $CDCl_3$, 298 K) δ: 151.9, 150.8, 142.9, 142.1, 134.3, 131.2, 129.4, 129.1, 127.6, 127.4, 126.6, 126.1, 116.8, 115.3, 112.1, 108.6, 84.4, 80.8, 80.4, 77.8.

HR ESI-MS (MeOH) m/z = 370.1346 $[MH]^+$ (calc. for $C_{26}H_{15}N_3$, 370.1344).

IR ν (cm^{-1}) 3135, 3120, 3072, 3048, 3022, 2207, 1622, 1575, 1563, 1524, 1440, 1411, 1393.

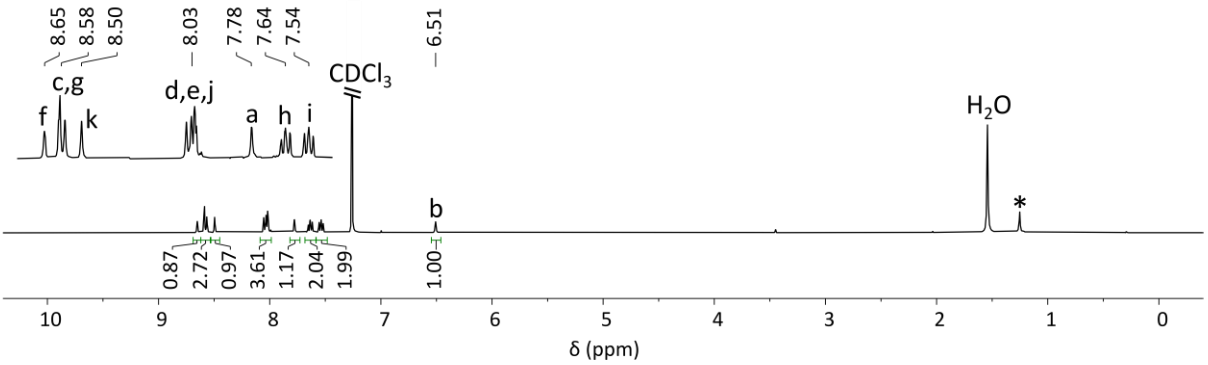

Figure S35 ^{1}H NMR spectrum (400 MHz, $CDCl_3$, 298 K) of **DD--N'.** *Small amount of H grease present.

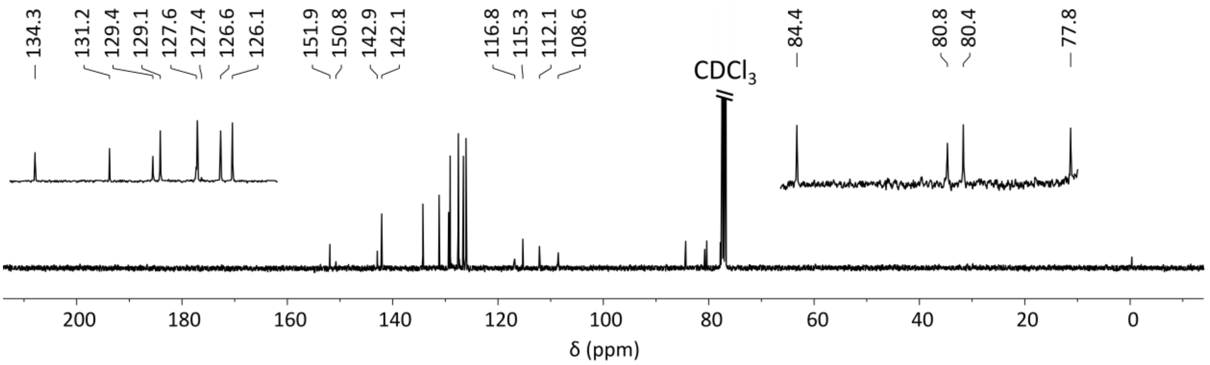

Figure S36 ^{13}C NMR spectrum (100 MHz, $CDCl_3$, 298 K) of **DD--N'.**

1.6. AA ligands

1.6.1. AA-N-AA

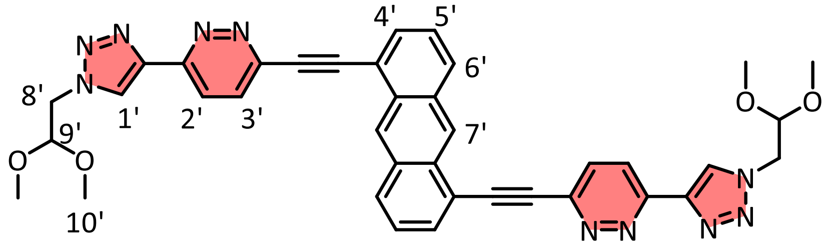

The combination of **AA-H** (300 mg, 1.16 mmol), 1,5-diiodoanthracene (207 mg, 0.482 mmol), $[Pd_2(dba)_3]$ (11 mg, 0.013 mmol), triphenylphosphine (13 mg, 0.048 mmol) and CuI (9 mg, 0.05 mmol) in 1:1 THF/TEA (15 mL) under nitrogen, and heated at 40 °C overnight. The reaction mixture was added

to DCM (100 mL) and EDTA/NH_4OH (100 mL) and stirred vigorously for 30 minutes. The organic layer was washed with water (100 mL) and the solvent removed under vacuum. Purification by column chromatography on silica (DCM to 1:10 acetone/DCM to 1:3 acetone/DCM to 1:10 methanol/DCM) gave the product as a yellow solid (220 mg, 0.317 mmol, 66%).

^1H NMR (400 MHz, $CDCl_3$, 298 K) *δ*: *integration given per 'half' of the ligand* 9.15 (1H, s, $H_{7'}$), 8.59 (1H, s, $H_{1'}$), 8.42 (1H, d, *J* = 8.7 Hz, $H_{2'}$), 8.28 (1H, d, *J* = 8.7 Hz, $H_{6'}$), 7.97 (1H, d, *J* = 7.7 Hz, $H_{4'}$), 7.92 (1H, d, *J* = 8.8 Hz, $H_{3'}$), 7.57 (1H, dd, *J* = 8.7 Hz, 7.2 Hz, $H_{5'}$), 4.75 (1H, t, *J* = 5.30 Hz, $H_{9'}$), 4.62 (2H, d, *J* = 5.12 Hz, $H_{8'}$), 3.48 (6H, s, $H_{10'}$).

Poor solubility precluded the collection of a ^1H DOSY NMR spectrum.

Poor solubility precluded the collection of a ^{13}C NMR spectrum.

HR ESI-MS (DCM/methanol) *m/z* = 693.2673 $[MH]^+$ (calc. for $C_{38}H_{33}N_{10}O_4$, 693.2686).

IR *v* (cm^{-1}) 3117, 3065, 2998, 2959, 2896, 2836, 2213, 1590, 1465, 1426.

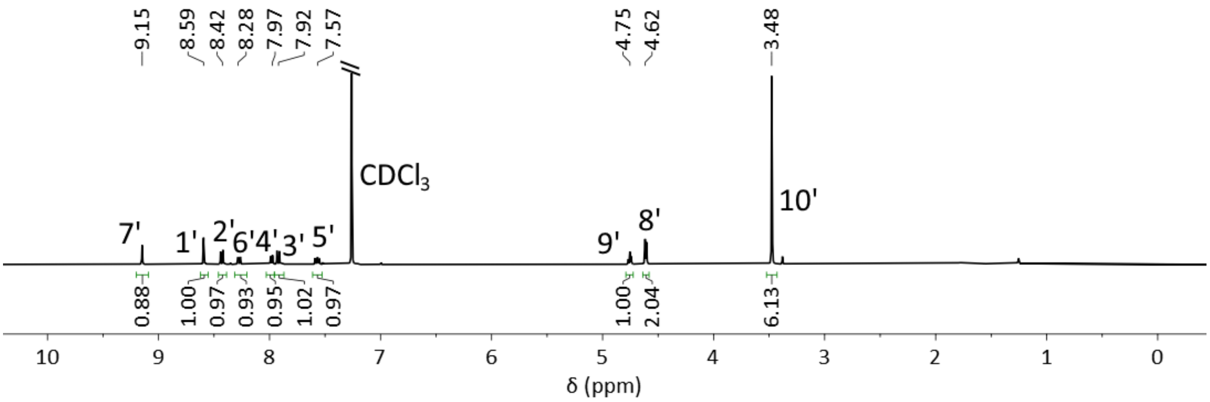

Figure S37 ^1H NMR spectrum (400 MHz, $CDCl_3$, 298 K) of **AA-N-AA**.

1.6.2. AA'--N'

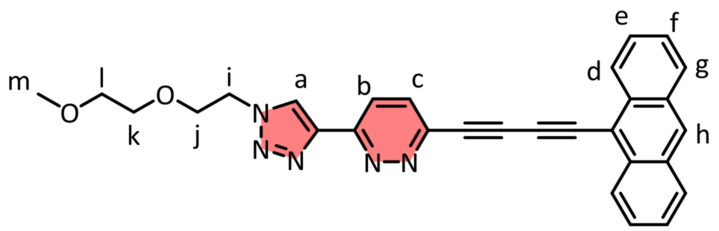

A mixture of **C** (300 mg, 0.864 mmol), **AA'-H** (236 mg, 0.864 mmol), copper(I) iodide (16.5 mg, 0.0173 mmol) and triethylamine (175 mg, 1.73 mmol) in deoxygenated acetonitrile (5 mL) was stirred at room temperature for 21 hours under a nitrogen atmosphere with the exclusion of light. After adding DCM (50 mL) and 0.1 M aqueous EDTA/NH_4OH (50 mL) and stirring vigorously for 30 minutes, the organic layer was removed under vacuum, the residue taken up again in DCM (100 mL), washed with water (100 mL), and removed under vacuum. Purification by column chromatography on silica (DCM to 1:4 acetone/DCM) gave the product as a yellow solid (48 mg, 0.101 mmol, 12%).

^1H NMR (400 MHz, $CDCl_3$, 298 K) *δ*: 8.60 (1H, s, H_a), 8.51 (2H, d, *J* = 8.8 Hz, H_d), 8.45 (1H, s, H_h), 8.28 (1H, d, *J* = 8.8 Hz, H_b), 7.98 (2H, d, *J* = 8.4 Hz, H_g), 7.72 (1H, dd, *J* = 8.8 Hz, H_c), 7.58 (2H, dd, *J* = 7.3 Hz,

H_e), 7.47 (2H, dd, J = 7.3 Hz, H_f), 4.61 (2H, t, J = 5.0 Hz, H_i), 3.89 (2H, t, J = 5.0 Hz, H_j), 3.59 (2H, m, H_k), 3.48 (2H, m, H_l), 3.33 (3H, s, H_m).

^{13}C NMR (100 MHz, $CDCl_3$, 298 K) δ: 151.6, 146.4, 144.9, 134.5, 131.1, 130.9, 129.9, 129.1, 127.8, 126.5, 126.1, 124.8, 122.6, 114.6, 84.0, 82.1, 80.5, 79.1, 71.9, 70.8, 69.4, 59.3, 50.8.

HR ESI-MS (MeOH) m/z = 474.1928 [MH]$^+$ (calc. for $C_{29}H_{23}N_5O_2$, 474.1930).

IR ν (cm^{-1}) 3124, 3054, 2923, 2877, 2825, 2203, 1722, 1672, 1590, 1519, 1457, 1432.

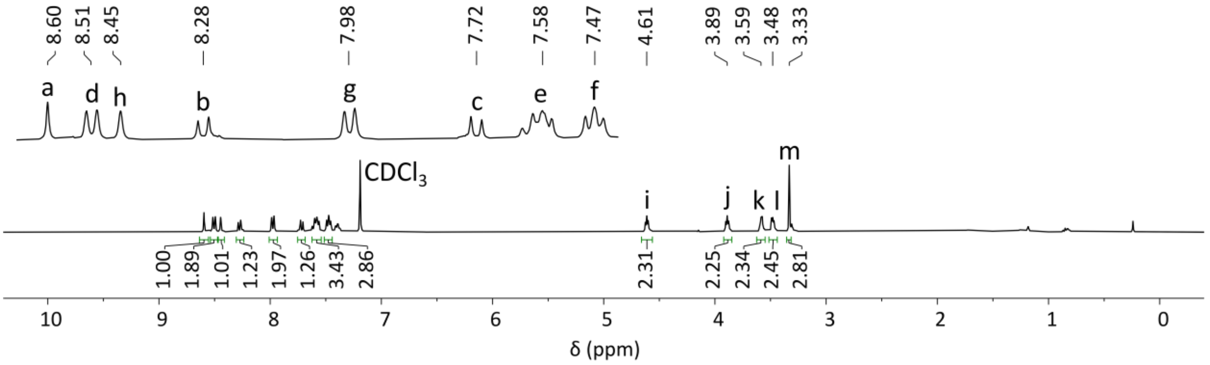

Figure S38 ^{1}H NMR spectrum (400 MHz, $CDCl_3$, 298 K) of **AA'--N'**.

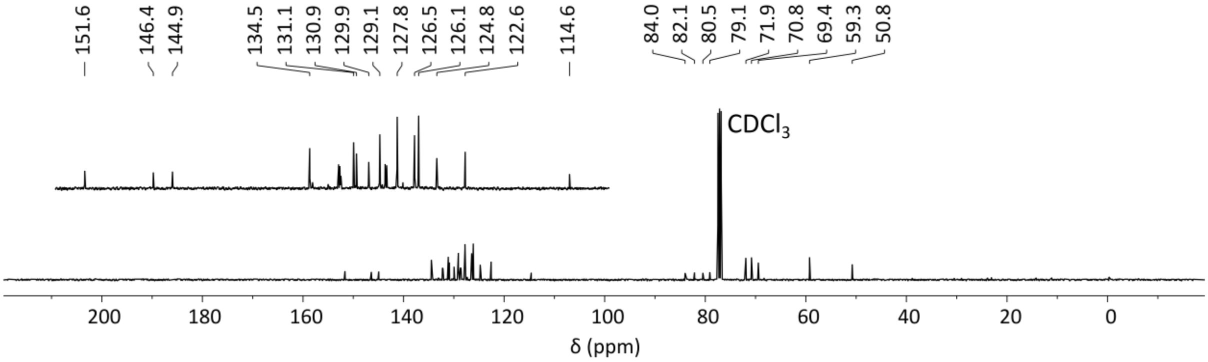

Figure S39 ^{13}C NMR spectrum (100 MHz, $CDCl_3$, 298 K) of **AA'--N'**.

1.7. Interlocked Architectures

1.7.1. [Pd(DA'-N')$_2$](BF$_4$)$_2$

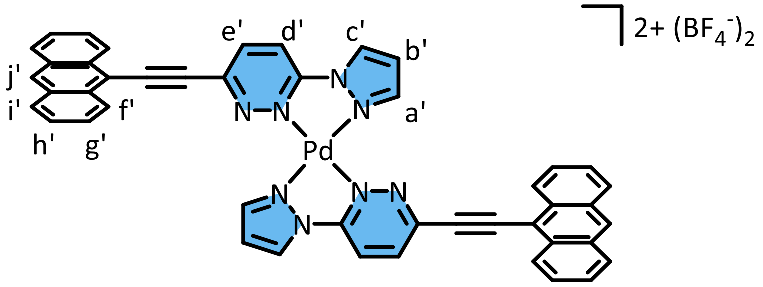

Using stock solutions, **DA'-N'** (3.11 mg, 8.98 µmol), and $[Pd(CH_3CN)_4](BF_4)_2$ (2.00 mg, 4.50 µmol) were combined in [D$_6$]DMSO (600 µL). Equilibration occurred in the time taken to run an NMR spectrum.

^{1}H NMR (400 MHz, [D$_6$]DMSO, 298 K) *δ*: 9.52 (1H, br s, H$_{c'}$), 9.21 (1H, d, *J* = 9.0 Hz, H$_{d'}$), 9.08 (1H, d, *J* = 9.0 Hz, H$_{e'}$), 8.94 (1H, br s, H$_{j'}$), 8.84 (1H, br s, H$_{a'}$), 8.70 (2H, d, *J* = 8.6 Hz, H$_{f'}$), 8.27 (2H, d, *J* = 8.6 Hz, H$_{i'}$), 7.90-7.88 (2H, m, H$_{g'}$), 7.73-7.74 (2H, m, H$_{h'}$), 7.53-7.51 (1H, m, H$_{b'}$).

l

D (× 10^{-10} m^2 s^{-1}, 400 MHz, [D$_6$]DMSO, 298 K) = 2.27.

HR ESI-MS (DMSO/acetonitrile) *m/z* = 399.0744 [**Pd(DA'-N')**$_2$]$^{2+}$ (calc. for $C_{46}H_{28}N_8Pd^{2+}$, 399.0744), *m/z* = 885.1537 [**Pd(DA'-N')**$_2$ + BF_4^-]$^+$ (calc. for $C_{46}H_{28}N_8PdBF_4^+$, 885.1523).

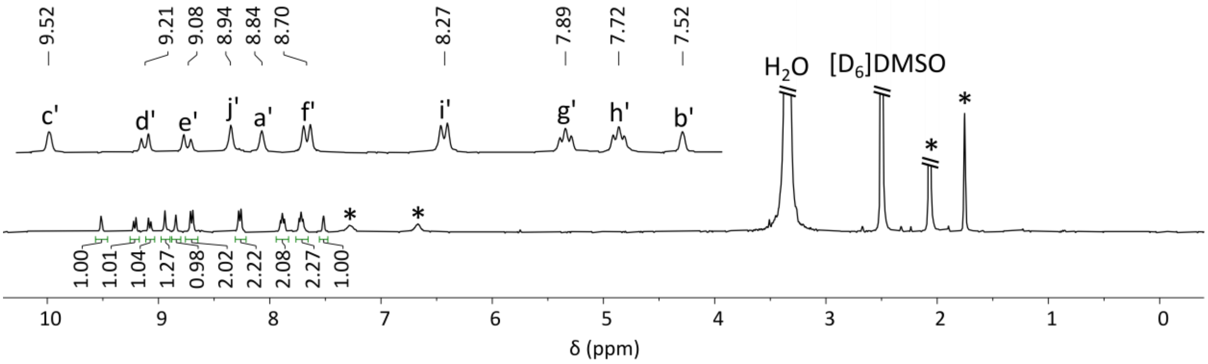

Figure S40 ^{1}H NMR spectrum (400 MHz, [D$_6$]DMSO, 298 K) of [Pd(**DA'-N'**)$_2$](BF$_4$)$_2$. Impurities from the Pd(II) source are indicated by *.

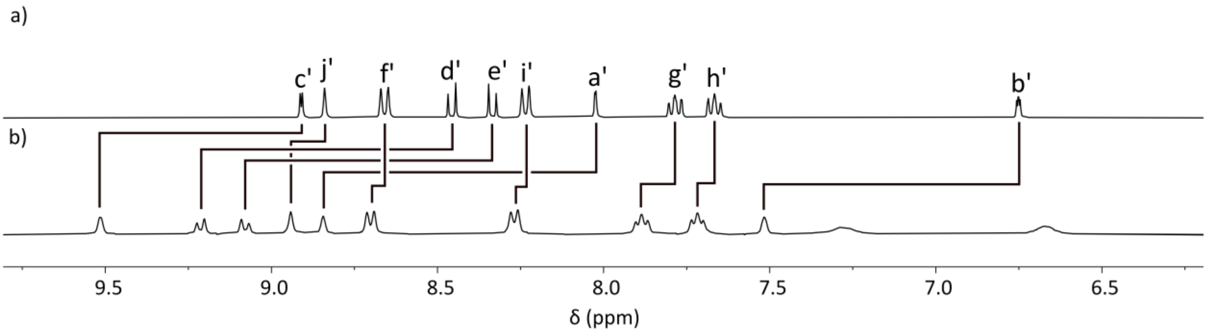

Figure S41 Partial ^{1}H NMR spectra (400 MHz, [D$_6$]DMSO, 298 K) of a) **DA'-N'** and b) [Pd(**DA'-N'**)$_2$](BF$_4$)$_2$.

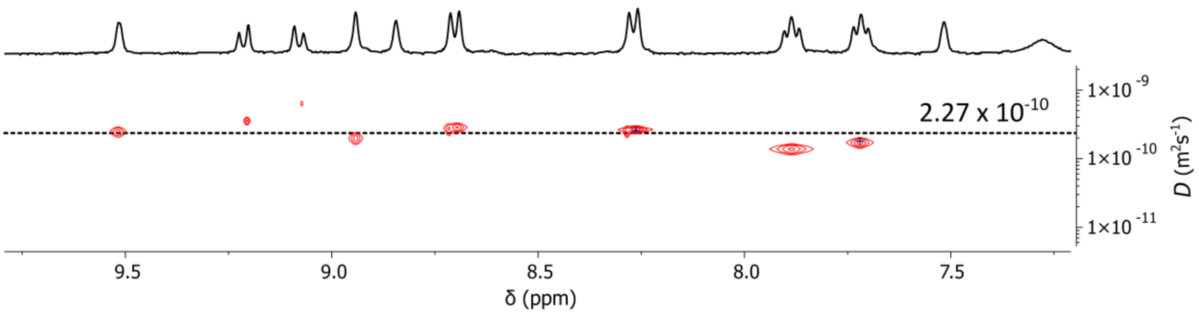

Figure S42 Partial ^{1}H DOSY NMR spectrum of [Pd(**DA'-N'**)$_2$](BF$_4$)$_2$ (400 MHz, [D$_6$]DMSO, 298 K).

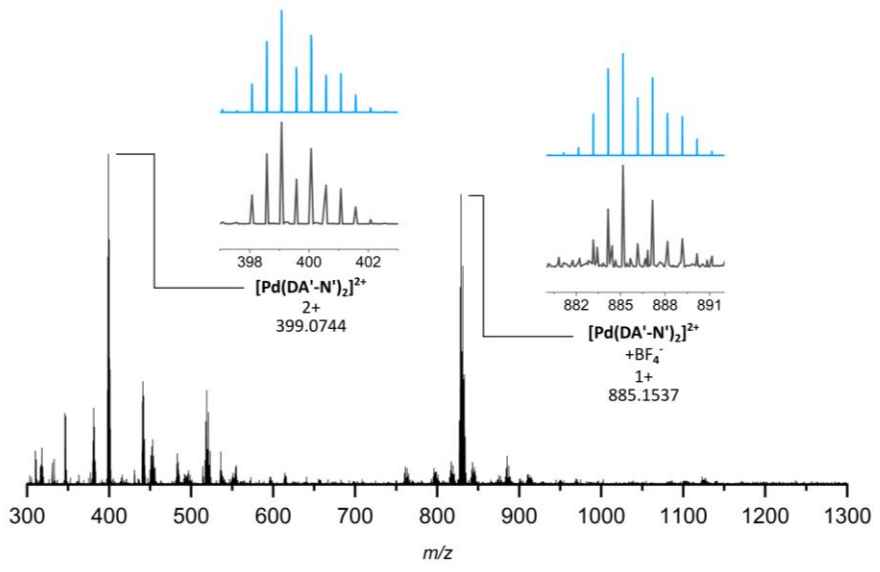

Figure S43 HR-ESI MS (DMSO/acetonitrile) of [Pd(**DA'-N'**)$_2$](BF_4)$_2$.

1.7.2. [Pd(AD'-N')$_2$](BF_4)$_2$

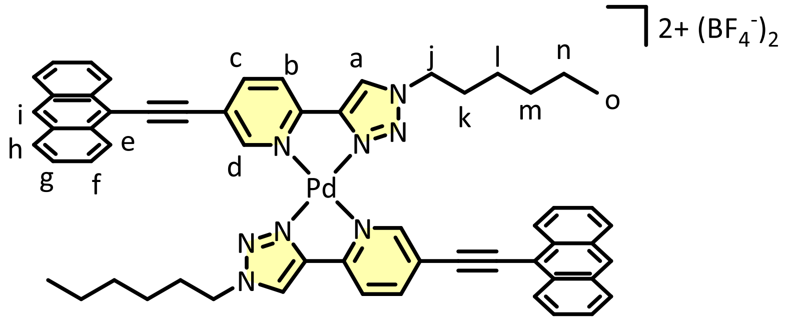

Using stock solutions, **AD'-N'** (3.87 mg, 9.00 µmol), and [Pd(CH_3CN)$_4$](BF_4)$_2$ (2.00 mg, 4.50 µmol) were combined in [D$_6$]DMSO (600 µL). Equilibration occurred in the time taken to run an NMR spectrum.

^{1}H NMR (400 MHz, [D$_6$]DMSO, 298 K) δ: 9.34 (1H, s, H_a), 9.16 (1H, br s, H_d), 9.04 (1H, d, J = 8.2 Hz, H_b), 8.74 (1H, s, H_i), 8.50 (2H, d, J = 8.5 Hz, H_e), 8.42 (1H, dd, J = 8.2 Hz, J = 2.1 Hz, H_c), 8.09 (2H, d, J = 8.4 Hz, H_h), 7.73 (2H, dd, J = 7.3 Hz, H_f), 7.58 (2H, dd, J = 7.3 Hz, H_g), 4.87-4.80 (2H, m, H_j), 2.15-2.08 (2H, m, H_k), 1.38-1.06 (6H, m, H_l, H_m, H_n), 0.64-0.58 (3H, t, J = 6.8 Hz, H_o).

D ($\times$ 10^{-10} m^2 s^{-1}, 400 MHz, [D$_6$]DMSO, 298 K) = 0.83.

HR ESI-MS (DMSO/acetonitrile) m/z = 483.1683 [**Pd(AD'-N')$_2$**]$^{2+}$ (calc. for $C_{58}H_{52}N_8Pd^{2+}$, 483.1685).

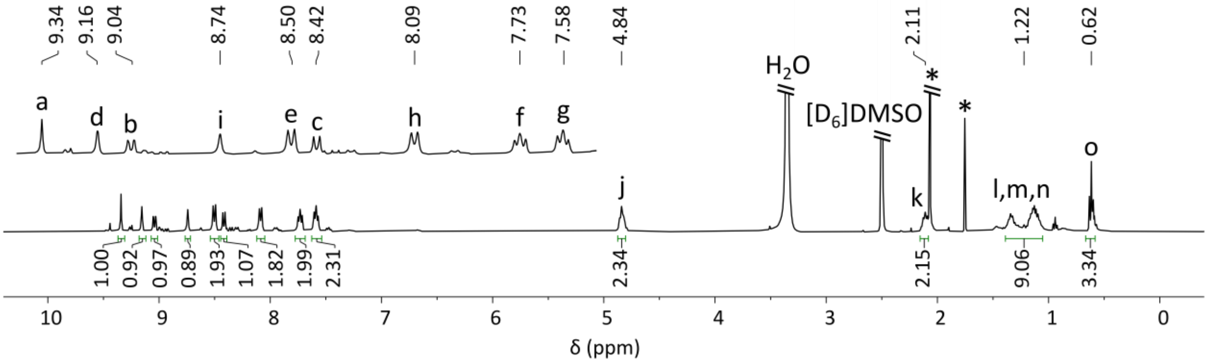

Figure S44 ^{1}H NMR spectrum (400 MHz, $[D_6]DMSO$, 298 K) of Pd(**AD'-N'**)$_2$](BF$_4$)$_2$. Impurities from the Pd(II) source are indicated by *.

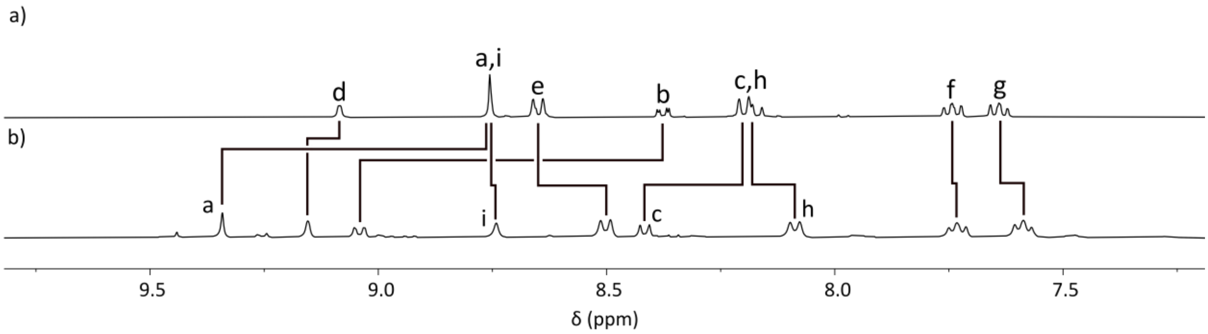

Figure S45 Partial ^{1}H NMR spectra (400 MHz, $[D_6]DMSO$, 298 K) of a) **AD'-N'** and b) [Pd(**AD'-N'**)$_2$](BF$_4$)$_2$.

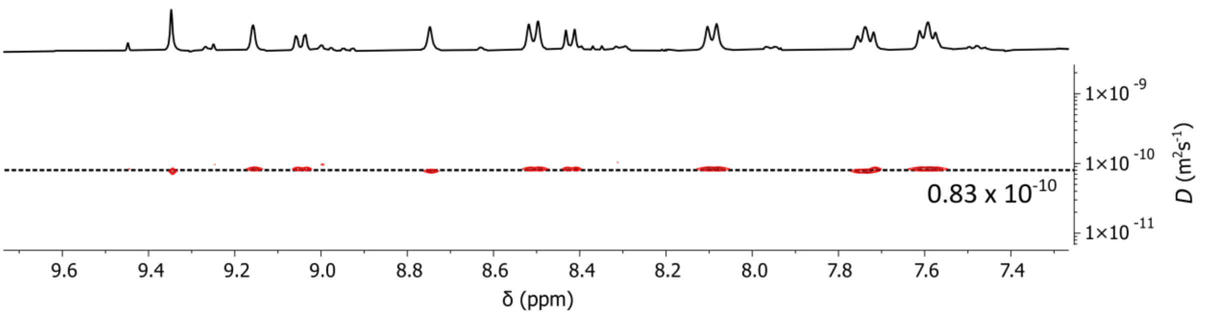

Figure S46 Partial ^{1}H DOSY NMR spectrum of Pd(**AD'-N'**)$_2$](BF$_4$)$_2$ (400 MHz, $[D_6]DMSO$, 298 K).

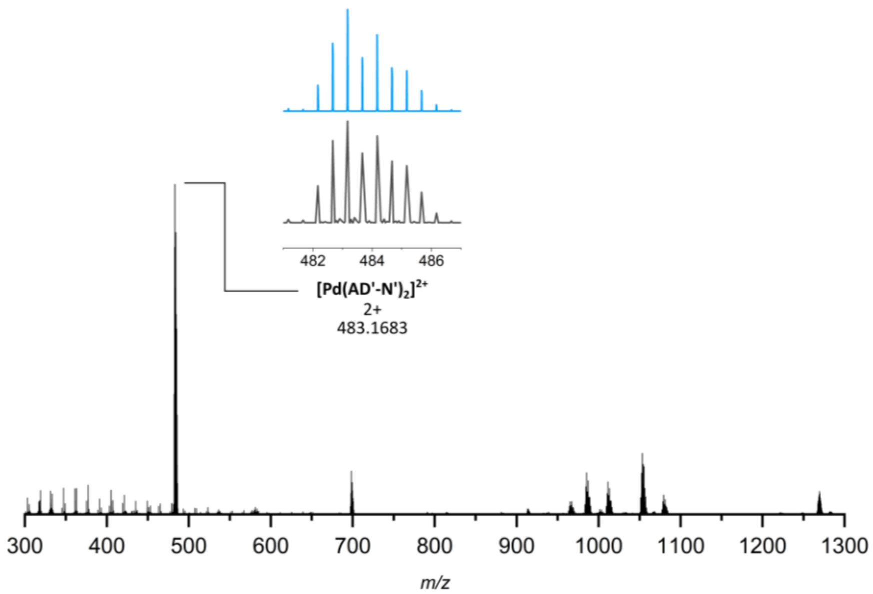

Figure S47 HR-ESI MS (DMSO/acetonitrile) of [Pd(**AD'-N'**)$_2$](BF_4)$_2$.

1.7.3. [Pd(DA'--N')$_2$](BF$_4$)$_2$

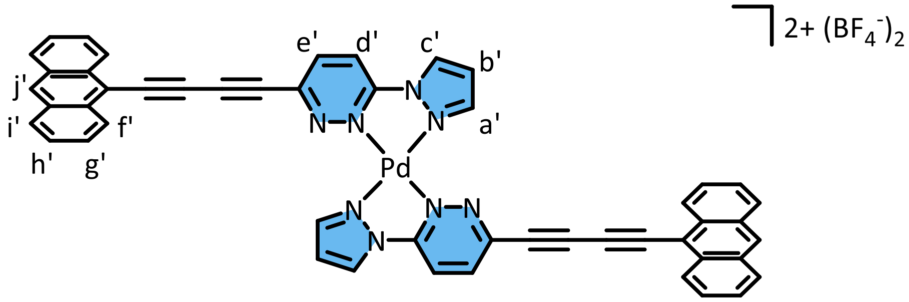

Using stock solutions, **DA'--N'** (2.59 mg, 6.99 µmol), and [Pd(CH_3CN)$_4$](BF_4)$_2$ (1.55 mg, 3.49 µmol) were combined in [D$_6$]DMSO (600 µL). Equilibration occurred in the time taken to run an NMR spectrum.

^{1}H NMR (400 MHz, [D$_6$]DMSO, 298 K) δ: 9.38 (1H, d, J = 3.0 Hz, H$_{c'}$), 9.05 (1H, d, J = 9.2 Hz, H$_{d'}$), 9.00 (1H, d, J = 9.2 Hz, H$_{e'}$), 8.88 (1H, br s, H$_{j'}$), 8.77 (1H, d, J = 1.6 Hz, H$_{a'}$), 8.40 (2H, d, J = 8.7 Hz, H$_{f'}$), 8.22 (2H, d, J = 8.6 Hz, H$_{i'}$), 7.78 (2H, m, H$_{g'}$), 7.65 (2H, m, H$_{h'}$), 7.38 (1H, br s, H$_{b'}$).

D (× 10^{-10} m^2 s^{-1}, 400 MHz, [D$_6$]DMSO, 298 K) = 1.61.

HR ESI-MS (DMSO/acetonitrile) m/z = 423.0748 [**Pd(DA'--N')$_2$**]$^{2+}$ (calc. for $C_{50}H_{28}N_8Pd^{2+}$, 423.0744).

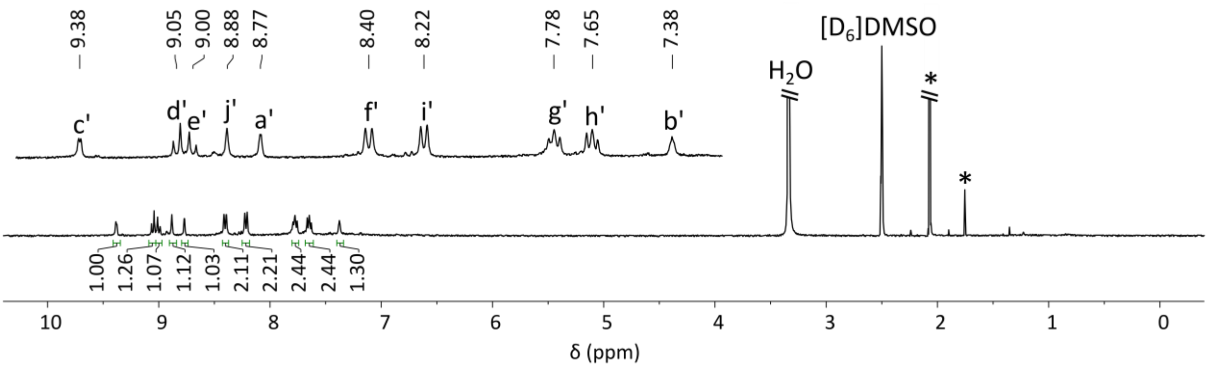

Figure S48 ^{1}H NMR spectrum (400 MHz, [D$_6$]DMSO, 298 K) of [Pd(**DA'--N'**)$_2$](BF$_4$)$_2$. Impurities from the Pd(II) source are indicated by *.

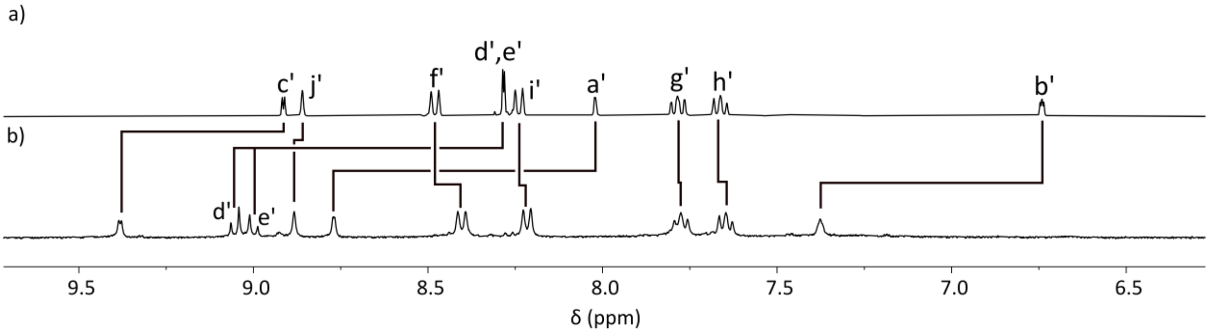

Figure S49 Partial ^{1}H NMR spectra (400 MHz, [D$_6$]DMSO, 298 K) of a) **DA'--N'** and b) [Pd(**DA'--N'**)$_2$](BF$_4$)$_2$.

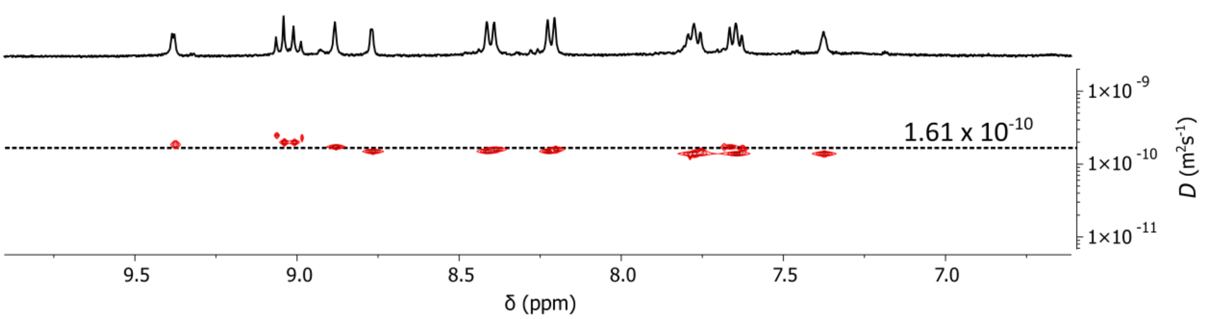

Figure S50 Partial ^{1}H DOSY NMR spectrum of [Pd(**DA'--N'**)$_2$](BF$_4$)$_2$ (400 MHz, [D$_6$]DMSO, 298 K).

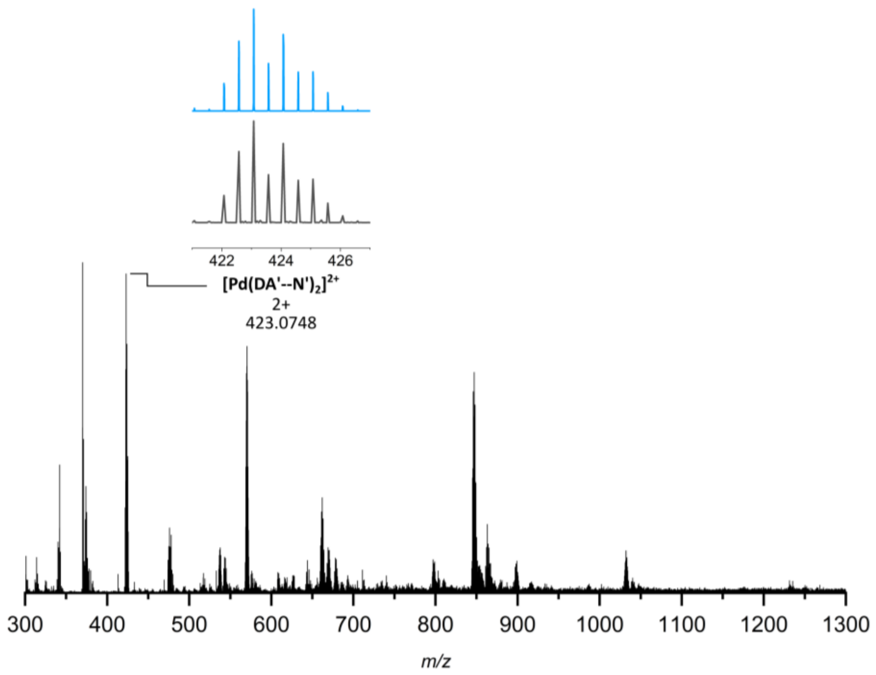

Figure S51 HR-ESI MS (DMSO/acetonitrile) of [Pd(**DA'--N'**)$_2$](BF$_4$)$_2$.

1.7.4. [Pd(AD--N')$_2$](BF$_4$)$_2$

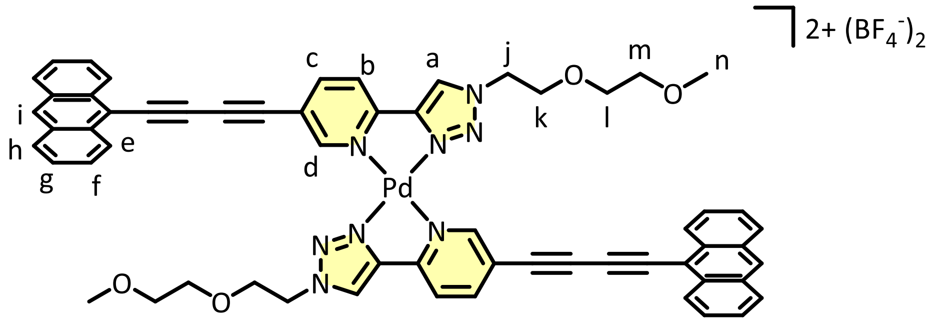

Using stock solutions, **AD--N'** (3.30 mg, 6.98 µmol), and [Pd(CH$_3$CN)$_4$](BF$_4$)$_2$ (1.55 mg, 3.49 µmol) were combined in [D$_6$]DMSO (600 µL). Equilibration occurred in the time taken to run an NMR spectrum.

^{1}H NMR (400 MHz, [D$_6$]DMSO, 323 K) δ: 9.39 (s, 1H, H$_a$), 9.34 (1H, br s, H$_d$), 8.83 (1H, d, J = 8.2 Hz, H$_b$), 8.76 (1H, s, H$_i$), 8.41 (1H, d, J = 8.2 Hz, H$_c$), 8.33 (2H, d, J = 8.5 Hz, H$_e$), 8.15 (2H, d, J = 8.4 Hz, H$_h$), 7.74-7.68 (2H, m, H$_f$), 7.62-7.58 (2H, m, H$_g$), 5.12-5.09 (2H, m, H$_j$), 4.24-4.21 (2H, m, H$_k$), 3.83-3.79 (2H, m, H$_l$), 3.59-3.55 (2H, m, H$_m$), 3.23 (3H, m, H$_n$, obscured by H$_2$O peak).

D ($\times$ 10^{-10} m^2 s^{-1}, 400 MHz, [D$_6$]DMSO, 298 K) = 0.70.

HR ESI-MS (DMSO/acetonitrile) m/z = 525.1490 [**Pd(AD--N')$_2$**]$^{2+}$ (calc. for C$_{60}$H$_{48}$N$_8$O$_4$Pd^{2+}, 525.1426), m/z = 1137.2867 [**Pd(AD--N')$_2$** + BF$_4^-$]$^+$ (calc. for C$_{60}$H$_{48}$N$_8$O$_4$PdBF$_4^+$, 1137.2889).

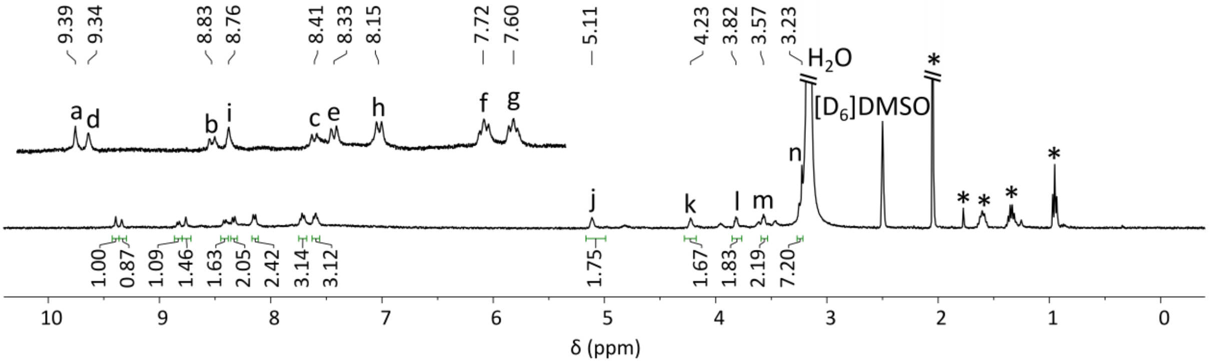

Figure S52 ^{1}H NMR spectrum (400 MHz, [D_6]DMSO, 338 K) of [Pd(**AD--N'**)$_2$](BF_4)$_2$. Impurities from the Pd(II) source are indicated by *.

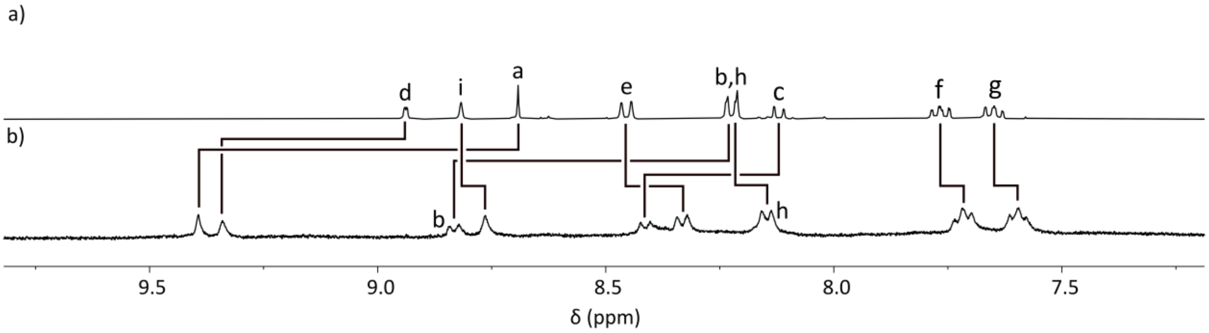

Figure S53 Partial ^{1}H NMR spectra (400 MHz, [D_6]DMSO) of a) **AD--N'** and b) [Pd(**AD--N'**)$_2$](BF_4)$_2$.

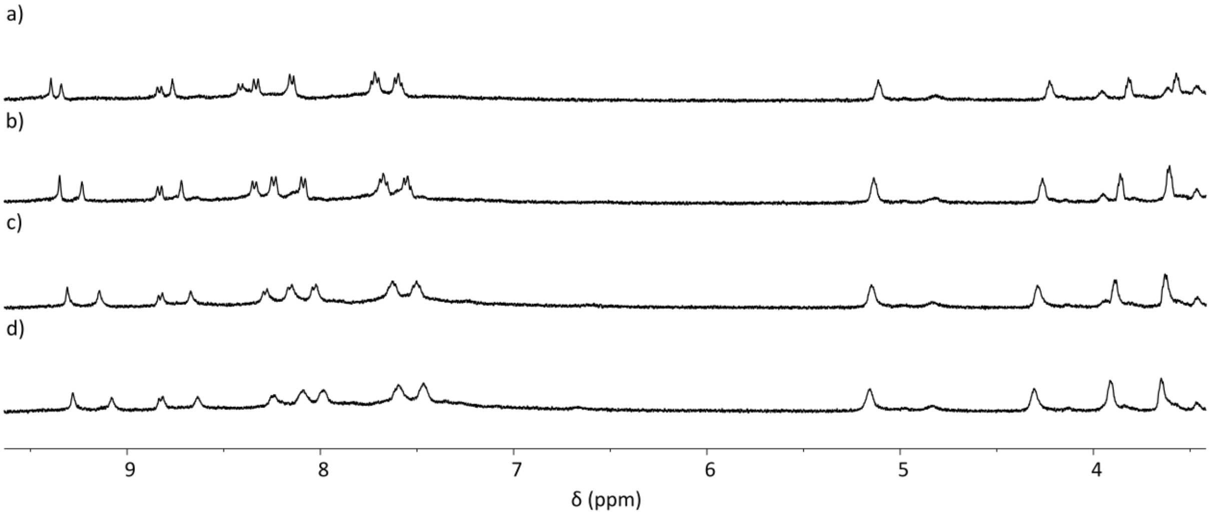

Figure S54 Partial variable temperature ^{1}H NMR spectra (400 MHz, [D_6]DMSO) of [Pd(**AD--N'**)$_2$](BF_4)$_2$ at a) 338 K, b) 323 K, c) 308 K and d) 298 K.

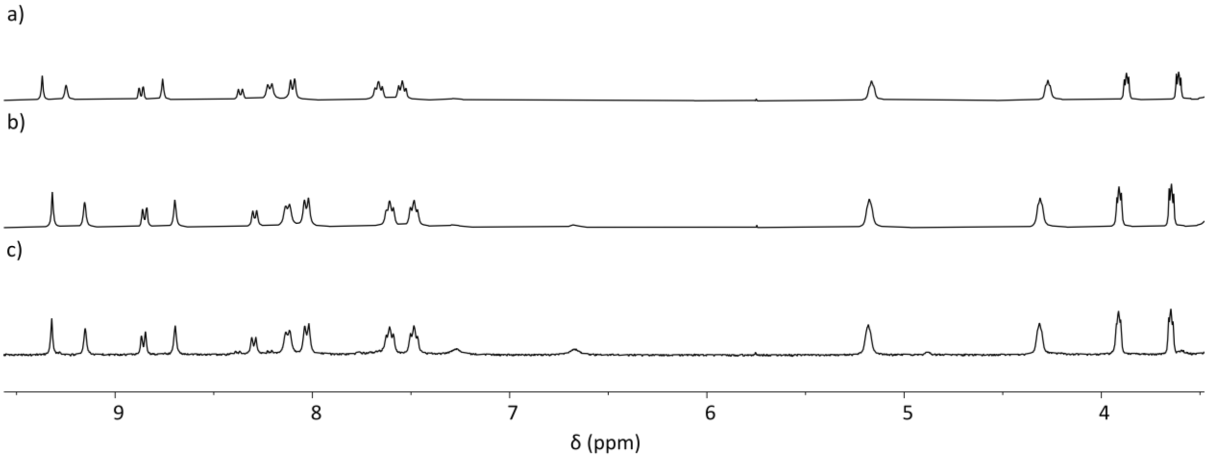

Figure S55 Partial ^{1}H NMR spectra (400 MHz, [D$_6$]DMSO, 298K) of [Pd(**AD--N'**)$_2$](BF$_4$)$_2$ at a) 8.18 mM, b) 4.09 mM, c) 2.05 mM.

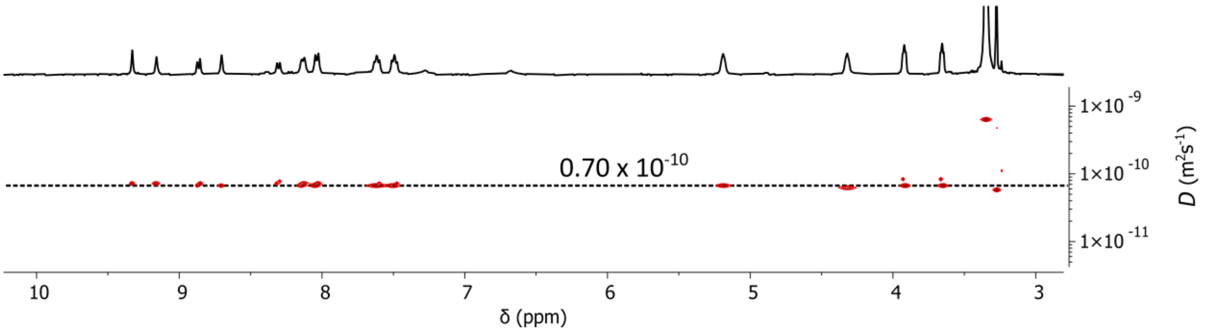

Figure S56 Partial ^{1}H DOSY NMR spectrum of Pd(**AD--N'**)$_2$](BF$_4$)$_2$ (400 MHz, [D$_6$]DMSO, 298 K).

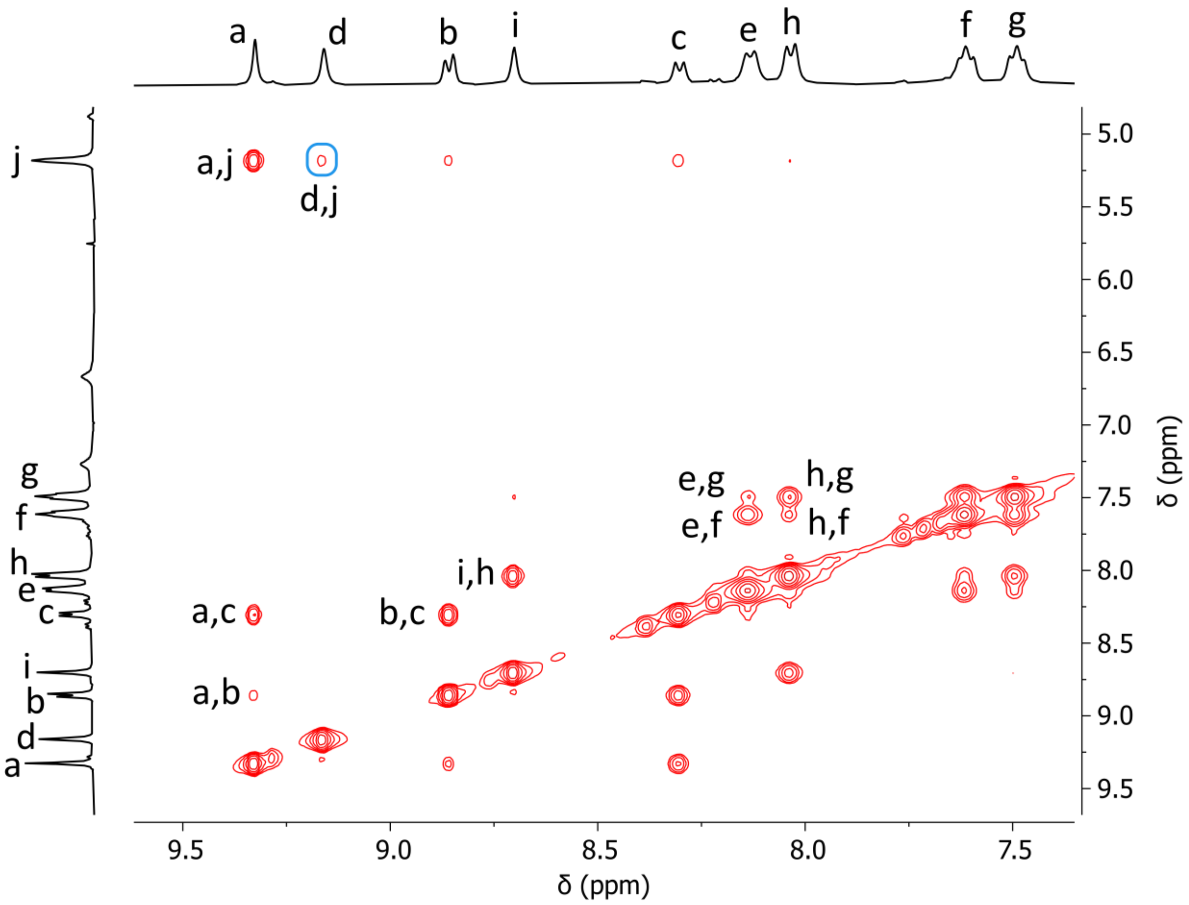

Figure S57 ^1H-^1H NOESY spectrum (400 MHz, [D$_6$]DMSO, 298 K) of [Pd(**AD--N'**)$_2$](BF$_4$)$_2$. Major through-space NOE correlations have been labelled, with the H$_d$, H$_j$ correlation highlighted by a blue box. This correlation and lack of other correlations suggest the presence of a monomeric [Pd(**AD--N'**)$_2$](BF$_4$)$_2$ complex in which ligands adopt a head-to-tail orientation.

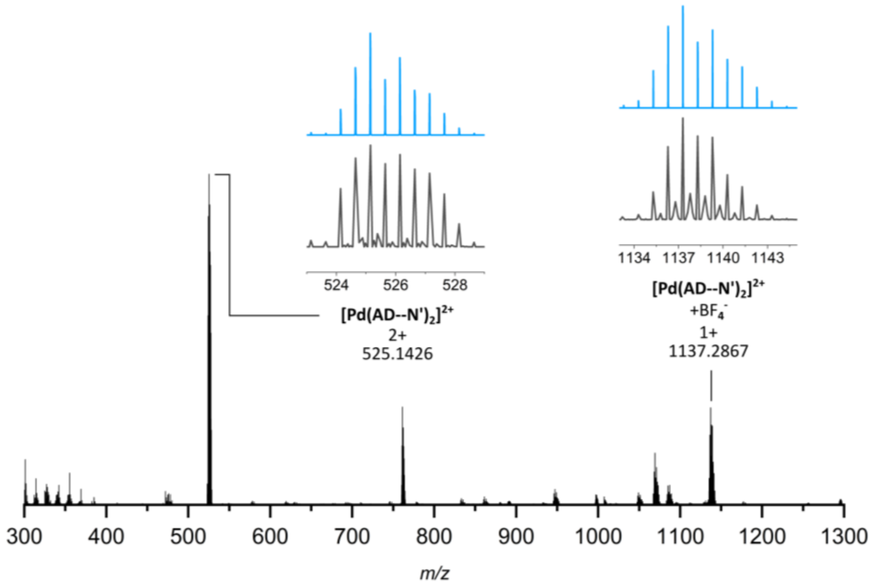

Figure S58 HR-ESI MS (DMSO/acetonitrile) of [Pd(**AD--N'**)$_2$](BF$_4$)$_2$. Note: despite the appearance of a +2 species overlapping with **[Pd(AD--N')$_2$ + BF$_4^-$]**$^+$, the VT ^1H NMR, serial dilution and ^1H-^1H NOESY NMR spectra provided above suggest that only [Pd(**AD--N'**)$_2$](BF$_4$)$_2$ is present on an NMR scale. It is possible in MS under extremely dilute conditions that we observe a dimerised adduct.

1.7.5. [Pd(DA'--N')(AD--N')]$_2$(BF$_4$)$_4$

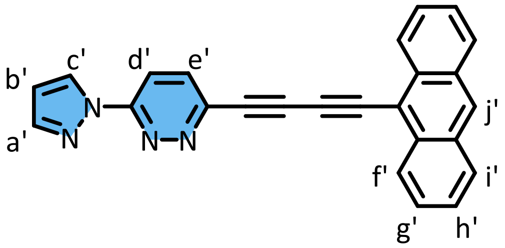

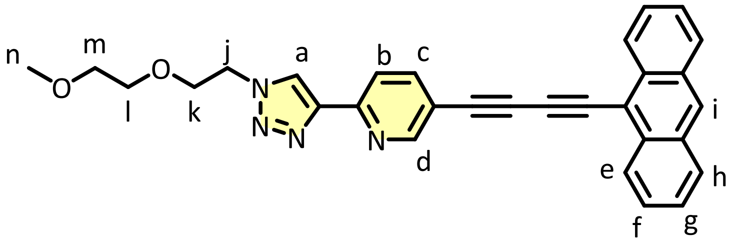

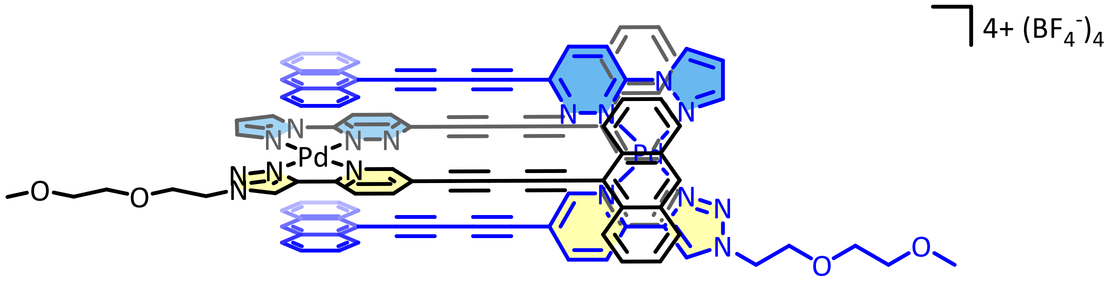

Using stock solutions, **DA'--N'** (1.73 mg, 4.66 µmol), **AD--N'** (2.20 mg, 4.66 µmol), and [Pd(CH$_3$CN)$_4$](BF$_4$)$_2$ (2.07 mg, 4.66 µmol) were combined in [D$_6$]DMSO (600 µL). Equilibration occurred in the time taken to run an NMR spectrum.

^{1}H NMR (400 MHz, [D$_6$]DMSO, 298 K) δ: 9.36 (2H, br s, H$_d$), 9.10 (2H, d, J = 8.8 Hz, H$_{d'}$), 8.95 (2H, s, H$_a$), 8.88-8.84 (4H, m, H$_{c'}$, H$_c$), 8.59 (2H, d, J = 8.8 Hz, H$_{e'}$), 8.26 (4H, m, H$_{j'}$, H$_i$), 8.04 (2H, d, J = 2.3 Hz, H$_{a'}$), 7.99 (2H, d, J = 8.5 Hz, H$_b$), 7.60-7.54 (8H, m, H$_{i'}$, H$_h$), 7.48-7.37 (8H, m, H$_{f'}$, H$_e$), 7.27 (2H, t, J = 2.3 Hz, H$_{b'}$), 7.22-7.12 (16H, m, H$_{g'}$, H$_{h'}$, H$_f$, H$_g$), 5.04 (4H, t, J = 5.0 Hz, H$_j$), 4.22 (4H, t, J = 5.0 Hz, H$_k$), 3.95-3.91 (4H, m, H$_l$), 3.74-3.70 (4H, m, H$_m$). **Note: H_n is obscured by the H_2O peak.*

^{1}H DOSY NMR (400 MHz, [D$_6$]DMSO, 298 K) D (× 10^{-10} m^2/s): 0.86.

Nanospray HR MS (DMSO) m/z = 474.8598 [[Pd(**DA'--N'**)(**AD--N'**)]$_2$]$^{4+}$ (calc. for $C_{110}H_{76}N_{16}O_4Pd_2^{4+}$, 474.8580), m/z = 948.7203 [[Pd(**DA'--N'**)(**AD--N'**)]$_2$ – 2H$^+$]$^{2+}$ (calc. for $C_{110}H_{74}N_{16}O_4Pd_2^{2+}$, 948.7088).

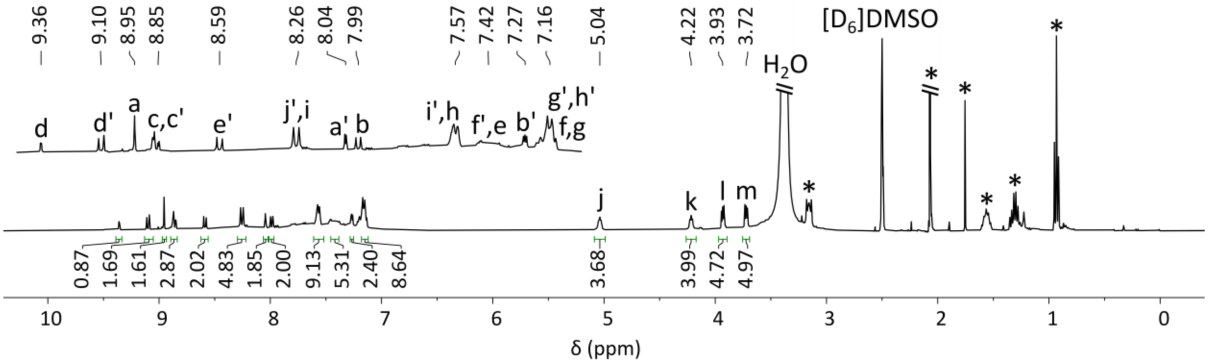

Figure S59 ^{1}H NMR spectrum (400 MHz, $[D_6]DMSO$, 298 K) of $[Pd($**DA'--N'**$)($**AD--N'**$)]_2(BF_4)_4$. Impurities from the Pd(II) source and ligand are indicated by *.

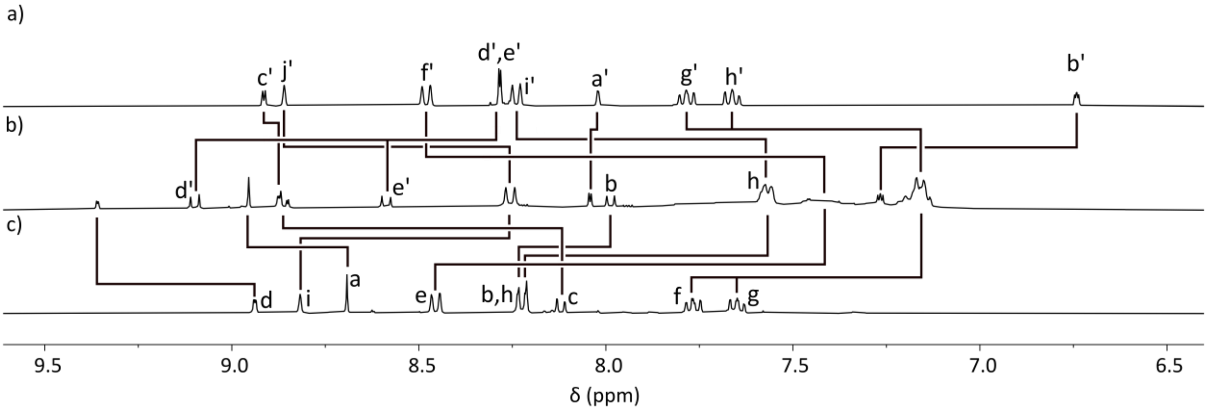

Figure S60 Partial ^{1}H NMR spectra (400 MHz, $[D_6]DMSO$, 298 K) of a) **DA'--N'**, b) $[Pd($**DA'--N'**$)($**AD--N'**$)]_2(BF_4)_4$ and c) **AD--N'**.

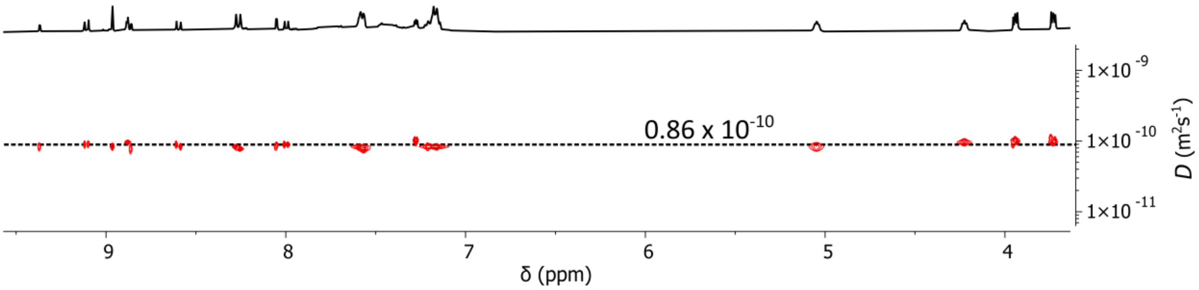

Figure S61 Partial ^{1}H DOSY NMR spectrum of $[Pd($**DA'--N'**$)($**AD--N'**$)]_2(BF_4)_4$ (400 MHz, $[D_6]DMSO$, 298 K).

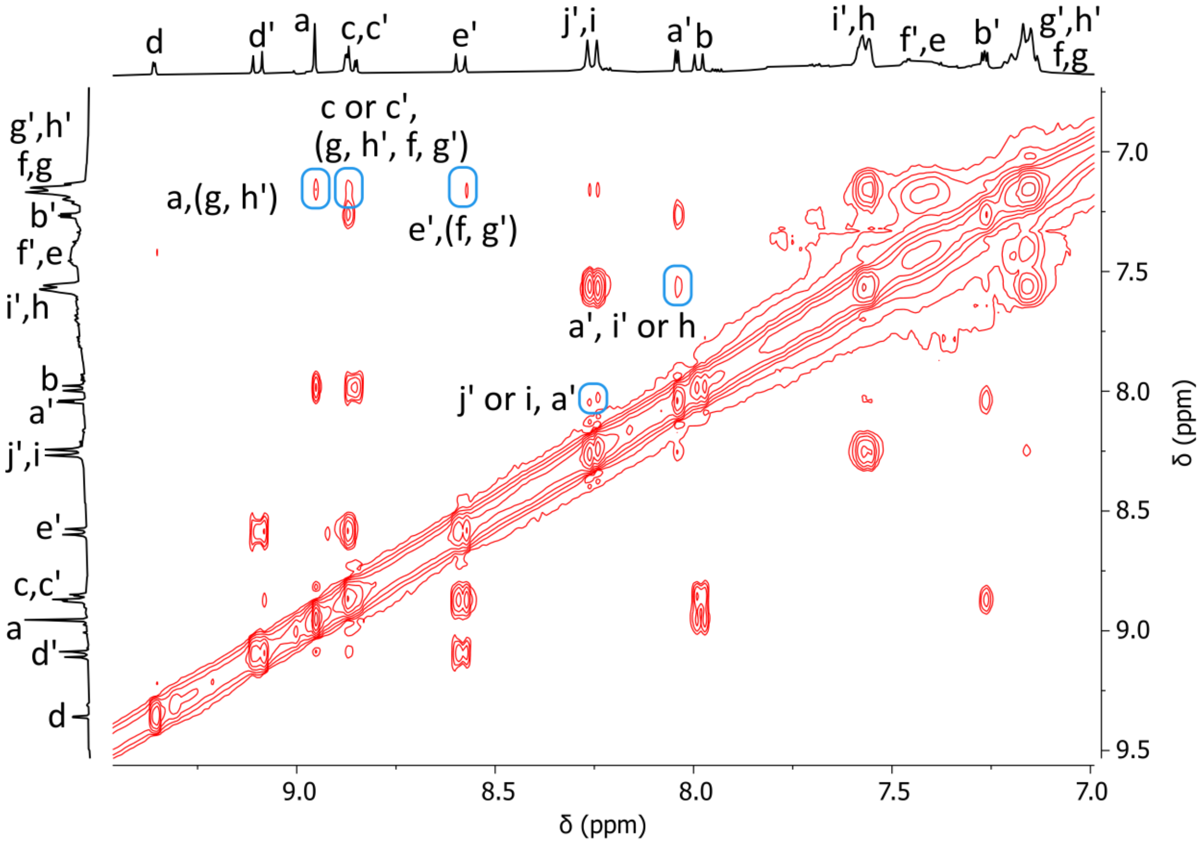

Figure S62 ^1H-^1H NOESY spectrum (400 MHz, [D$_6$]DMSO, 298 K) of [Pd(**DA'--N'**)(**AD--N'**)]$_2$(BF$_4$)$_4$. Only the correlations demonstrating dimerisation have been highlighted by blue squares and labelled. In most cases, analogous environments of the anthracene panels on each ligand overlap and so the exact environments producing each correlation cannot be precisely determined and instead denoted as 'proton x or proton y'. It is likely a through-space NOE correlation to both 'proton x' and 'proton y' could exist but irrespective of their exact identity, they can only be explained through the existence of a clippane.

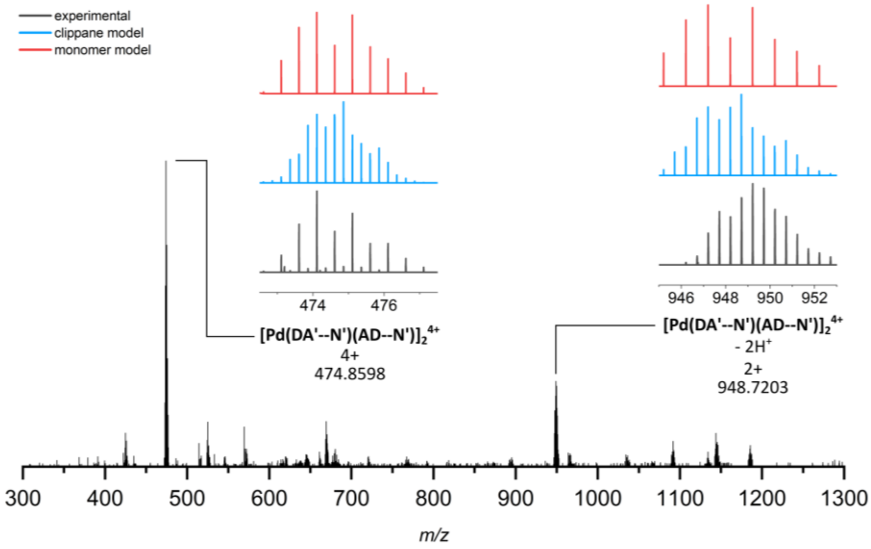

Figure S63 Nanospray HR MS (DMSO) of [Pd(**DA'--N'**)(**AD--N'**)]$_2$(BF$_4$)$_4$. Note the observed *m/z* peaks corresponding to the clippane overlap with that of the monomer at half the respective charge. Models of the monomer (red) have been included for reference.

1.7.6. [Pd(DA'--N)(AD--N')]$_2$(BF$_4$)$_4$

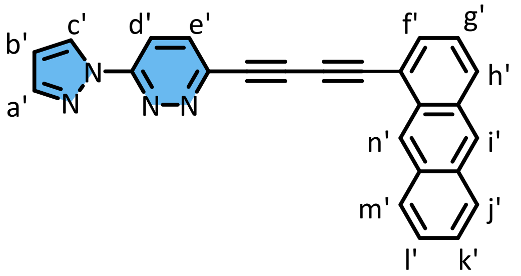

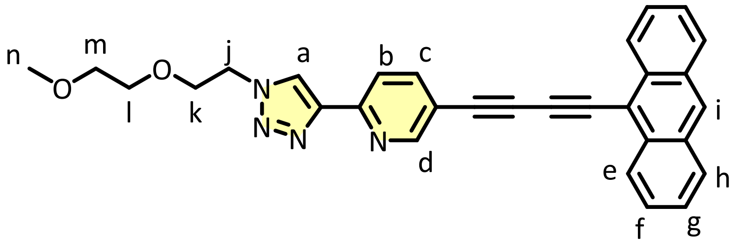

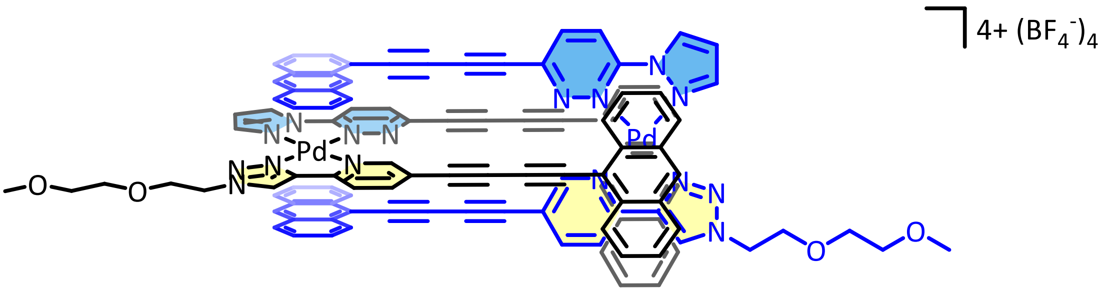

Using stock solutions, **DA'--N** (1.66 mg, 4.50 µmol), **AD--N'** (2.13 mg, 4.50 µmol), and [Pd(CH$_3$CN)$_4$](BF$_4$)$_2$ (2.00 mg, 4.50 µmol) were combined in [D$_6$]DMSO (550 µL). Equilibration occurred in the time taken to run an NMR spectrum.

^{1}H NMR (400 MHz, [D$_6$]DMSO, 298 K) δ: 9.28 (2H, d, J = 1.7 Hz, H_d), 9.10 (2H, d, J = 8.8 Hz, $H_{e'}$), 8.88 (2H, dd, J = 8.1, 1.7 Hz, H_c), 8.85-8.81 (4H, m, $H_{c'}$, H_a), 8.56 (2H, d, J = 9.2 Hz, $H_{d'}$), 8.23 (2H, br s, H_i), 8.13 (2H, br s, $H_{i'}$), 8.10 (2H, d, J = 2.3 Hz, $H_{a'}$), 7.92-7.87 (4H, m, $H_{n'}$, H_b), 7.67 (2H, d, J = 8.5 Hz, $H_{j'}$), 7.58 (2H, d, J = 8.8 Hz, $H_{h'}$), 7.56-7.50 (6H, m, $H_{m'}$, H_h), 7.48-7.37 (8H, m, $H_{k'}$, $H_{l'}$, H_e), 7.24 (2H, t, J = 2.5 Hz, $H_{b'}$), 7.18-7.10 (10H, m, $H_{f'}$, H_f, H_g), 6.79 (2H, dd, J = 8.5, 7.0 Hz, $H_{g'}$), 5.07-4.94 (4H, m, H_j), 4.23-4.18 (4H, m, H_k), 3.93-3.90 (4H, m, H_l), 3.72-3.69 (4H, m, H_m), 3.36 (6H, s, H_n).

^{1}H DOSY NMR (400 MHz, [D$_6$]DMSO, 298 K) D (× 10^{-10} m^2/s): 0.87.

Nanospray HR MS (DMSO) m/z = 1036.7216 [[Pd(**DA'--N**)(**AD--N'**)]$_2$ + 2BF$_4^-$]$^{2+}$ (calc. for $C_{110}H_{76}N_{16}O_4Pd_2B_2F_8^{2+}$, 1036.7195).

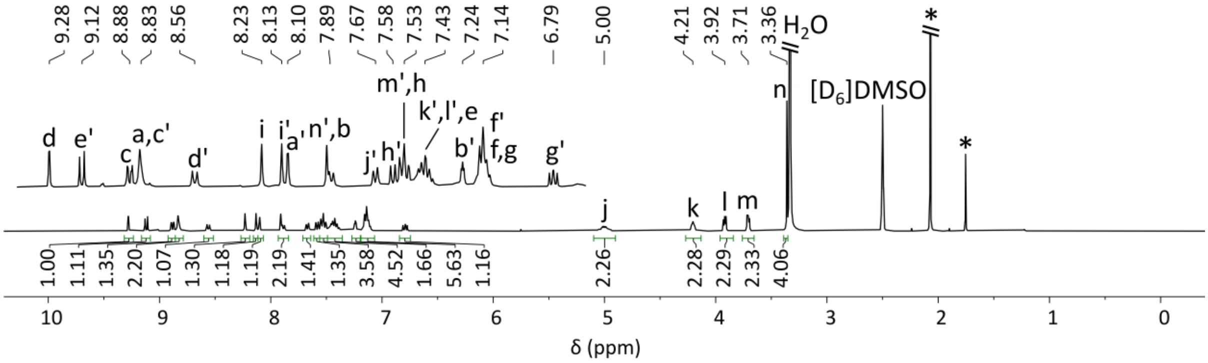

Figure S64 ^{1}H NMR spectrum (400 MHz, [D$_6$]DMSO, 298 K) of [Pd(**DA'--N**)(**AD--N'**)]$_2$(BF$_4$)$_4$. Impurities from the Pd(II) source and ligand are indicated by *.

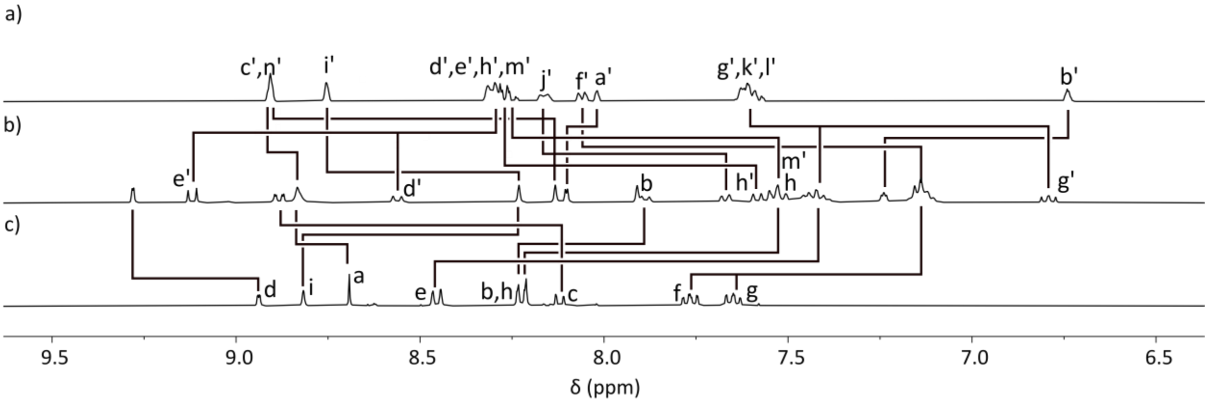

Figure S65 Partial ^{1}H NMR spectra (400 MHz, [D$_6$]DMSO, 298 K) of a) **DA'--N**, b) [Pd(**DA'--N**)(**AD--N'**)]$_2$(BF$_4$)$_4$ and c) **AD--N'**.

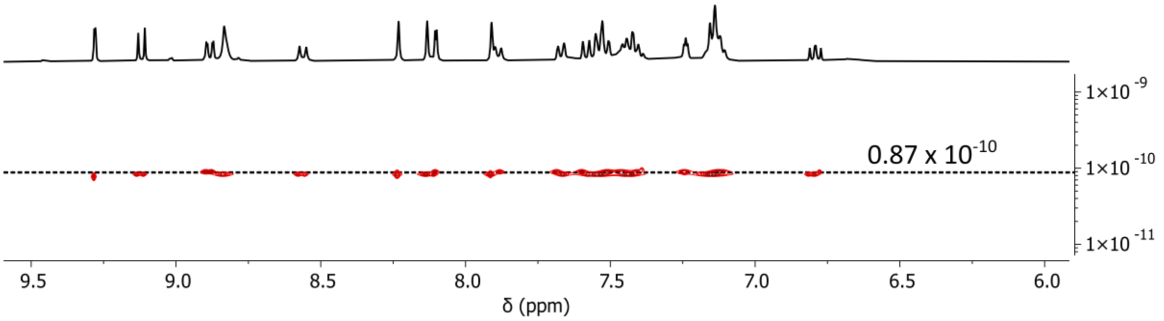

Figure S66 Partial ^{1}H DOSY NMR spectrum of [Pd(**DA'--N**)(**AD--N'**)]$_2$(BF$_4$)$_4$ (400 MHz, [D$_6$]DMSO, 298 K).

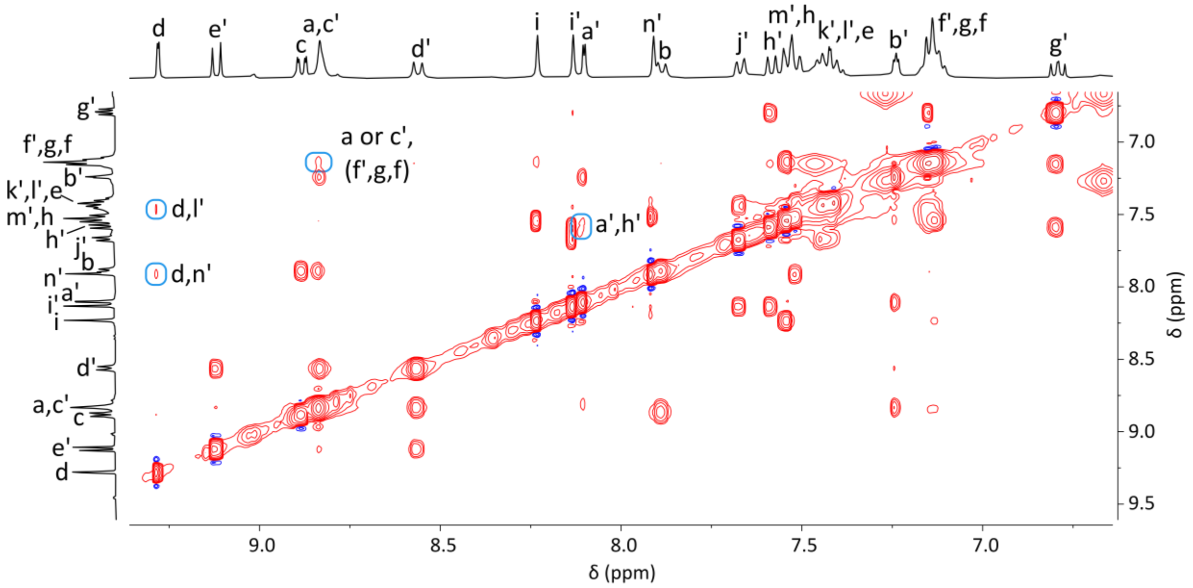

Figure S67 ^{1}H-^{1}H NOESY spectrum (400 MHz, [D_6]DMSO, 298 K) of [Pd(**DA'--N**)(**AD--N'**)]$_2$(BF_4)$_4$. Only the correlations demonstrating dimerisation have been highlighted by blue squares and labelled. In most cases, analogous environments of the anthracene panels on each ligand overlap and so the exact protons producing each correlation cannot be precisely determined. Irrespective of their exact identity, the described through-space NOE correlations can only be explained through the existence of a clippane.

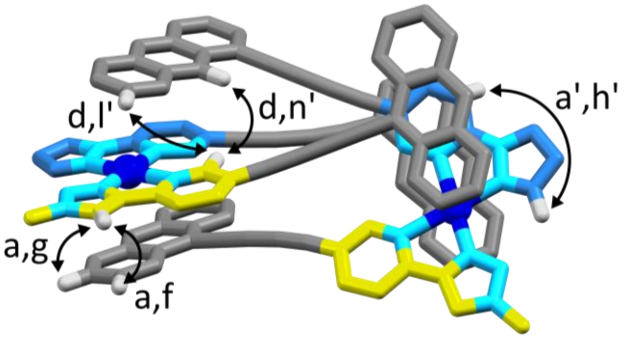

Figure S68 Model of [Pd(**DA'--N**)(**AD--N'**)]$_2$(BF_4)$_4$ depicting the through-space NOE correlations observed in the ^{1}H-^{1}H NOESY spectrum above. The correlations, particularly between H_d and $H_{n'}$ as well as H_d and $H_{l'}$ suggest the 1-substituted anthracenyl group of **DA'--N** prefers to stack with the more electron deficient pyridyl ring of **AD--N'** rather than the pyridazinyl ring of **DA'--N**.

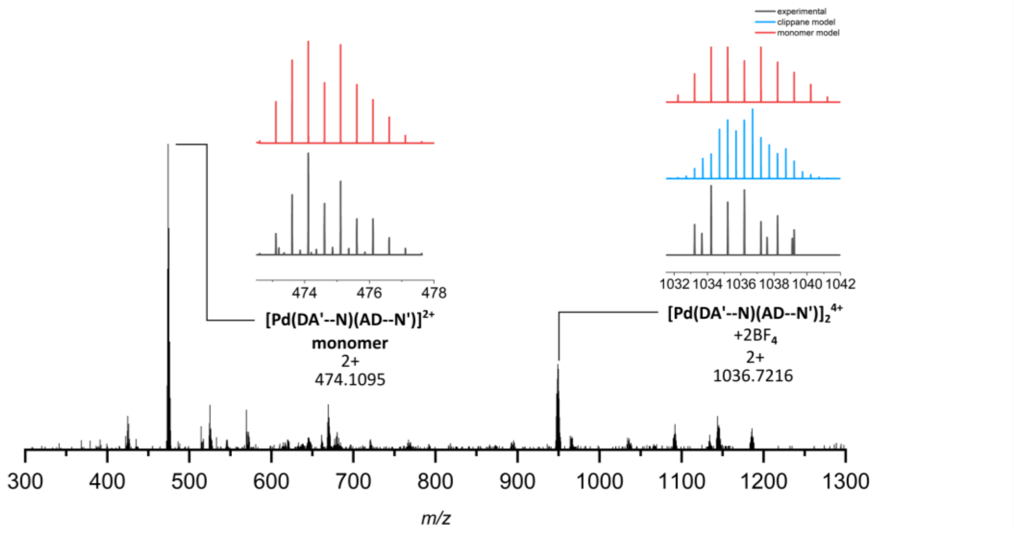

Figure S69 Nanospray HR MS (DMSO) of [Pd(**DA'--N**)(**AD--N'**)]$_2$(BF$_4$)$_4$. Note the observed *m/z* peaks corresponding to the clippane overlap with that of the monomer at half the respective charge. Models of the monomer (red) have been included for reference.

1.7.7. [Pd(DD--N')(AA--N')]$_2$(BF$_4$)$_4$

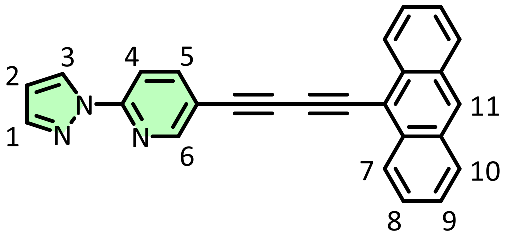

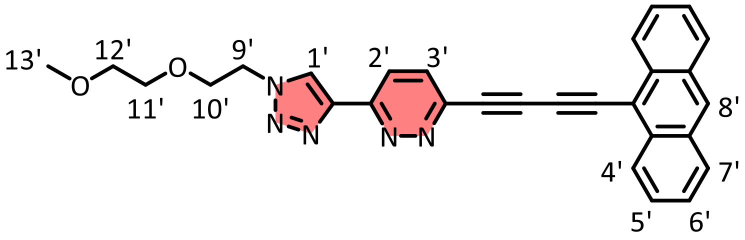

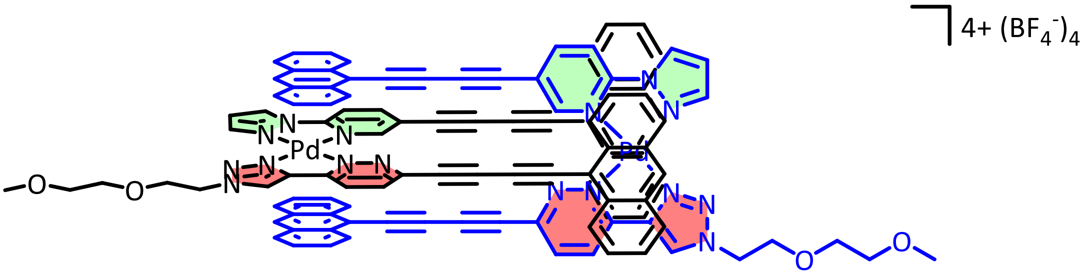

Stock solutions of pre-formed [Pd(**DD--N'**)$_2$](BF$_4$)$_2$ and [Pd(**AA'--N'**)$_2$](BF$_4$)$_2$ were combined in equimolar amounts to give a 1:1:1 ratio of **DD--N'** (2.27 mg, 6.15 mmol), **AA'--N'** (2.91 mg, 6.15 mmol), and [Pd(CH$_3$CN)$_4$](BF$_4$)$_2$ (2.73 mg, 6.15 mmol) in 800 μL [D$_6$]DMSO. Equilibration occurred in the time taken to run an NMR spectrum.

^{1}H NMR (400 MHz, [D$_6$]DMSO, 298 K) δ: 9.30 (2H, br s, H$_6$), 9.15 (2H, s, H$_{1'}$), 8.99-8.94 (4H, m, H$_5$, H$_{3'}$), 8.81 (2H, d, J = 3.0 Hz, H$_3$), 8.53 (1H, d, J = 8.8 Hz, H$_{2'}$), 8.25 (4H, m, H$_{11}$, H$_{8'}$), 8.02 (2H, d, J = 8.8 Hz, H$_4$), 7.94 (1H, d, J = 1.3 Hz, H$_1$), 7.58-7.52 (8H, m, H$_{10}$, H$_{7'}$), 7.49-7.38 (8H, m, H$_7$, H$_{4'}$), 7.20-7.10 (17H, m, H$_2$, H$_8$, H$_9$, H$_{5'}$, H$_{6'}$), 5.12 (4H, t, J = 5.0 Hz, H$_{9'}$), 4.24 (2H, t, J = 5.0 Hz, H$_{10'}$), 3.95-3.91 (4H, m, H$_{11'}$), 3.74-3.70 (4H, m, H$_{12'}$). **Note: H$_{13'}$ is obscured by the H$_2$O peak.*

^{1}H DOSY NMR (400 MHz, [D$_6$]DMSO, 298 K) D ($\times 10^{-10}$ m^2/s): 0.70.

Nanospray HR MS (DMSO) m/z = 948.7209 [[Pd(**DD--N'**)(**AA--N'**)]$_2$ – 2H$^+$]$^{2+}$ (calc. for $C_{110}H_{74}N_{16}O_4Pd_2^{2+}$, 948.7088), m/z = 1036.7255 [[Pd(**DD--N'**)(**AA--N'**)]$_2$+ 2BF$_4^-$]$^{2+}$ (calc. for $C_{110}H_{76}N_{16}O_4Pd_2B_2F_8^{2+}$, 1036.7195).

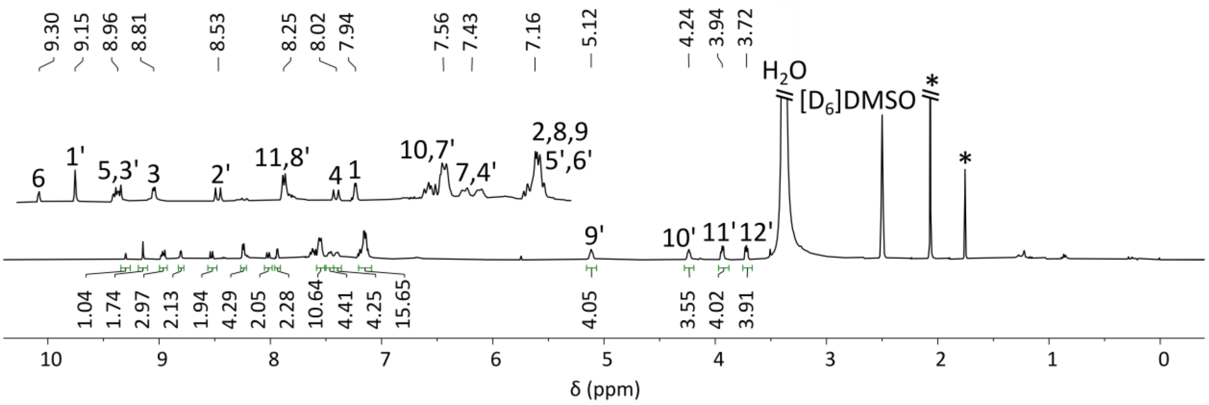

Figure S70 ^{1}H NMR spectrum (400 MHz, [D$_6$]DMSO, 298 K) of [Pd(**DD--N'**)(**AA'--N'**)]$_2$(BF$_4$)$_4$. Impurities from the Pd(II) source and ligand are indicated by *.

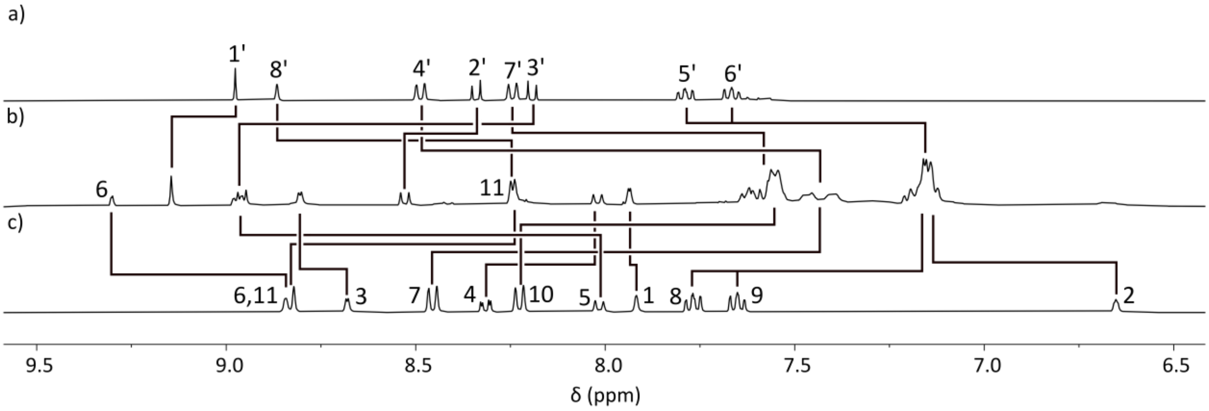

Figure S71 Partial ^{1}H NMR spectra (400 MHz, [D$_6$]DMSO, 298 K) of a) **AA--N'**, b) [Pd(**AA--N'**)(**DD--N'**)]$_2$(BF$_4$)$_4$ and c) **DD--N'**.

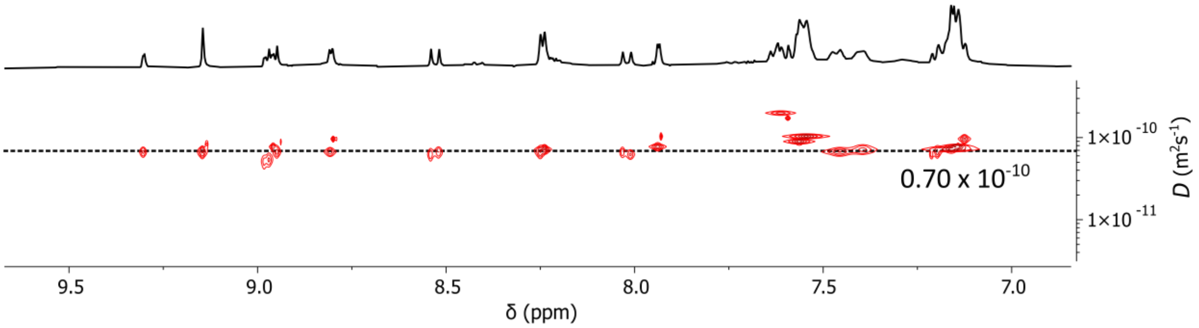

Figure S72 Partial ^{1}H DOSY NMR spectrum of [Pd(**DD--N'**)(**AA'--N'**)]$_2$(BF$_4$)$_4$ (400 MHz, [D$_6$]DMSO, 298 K).

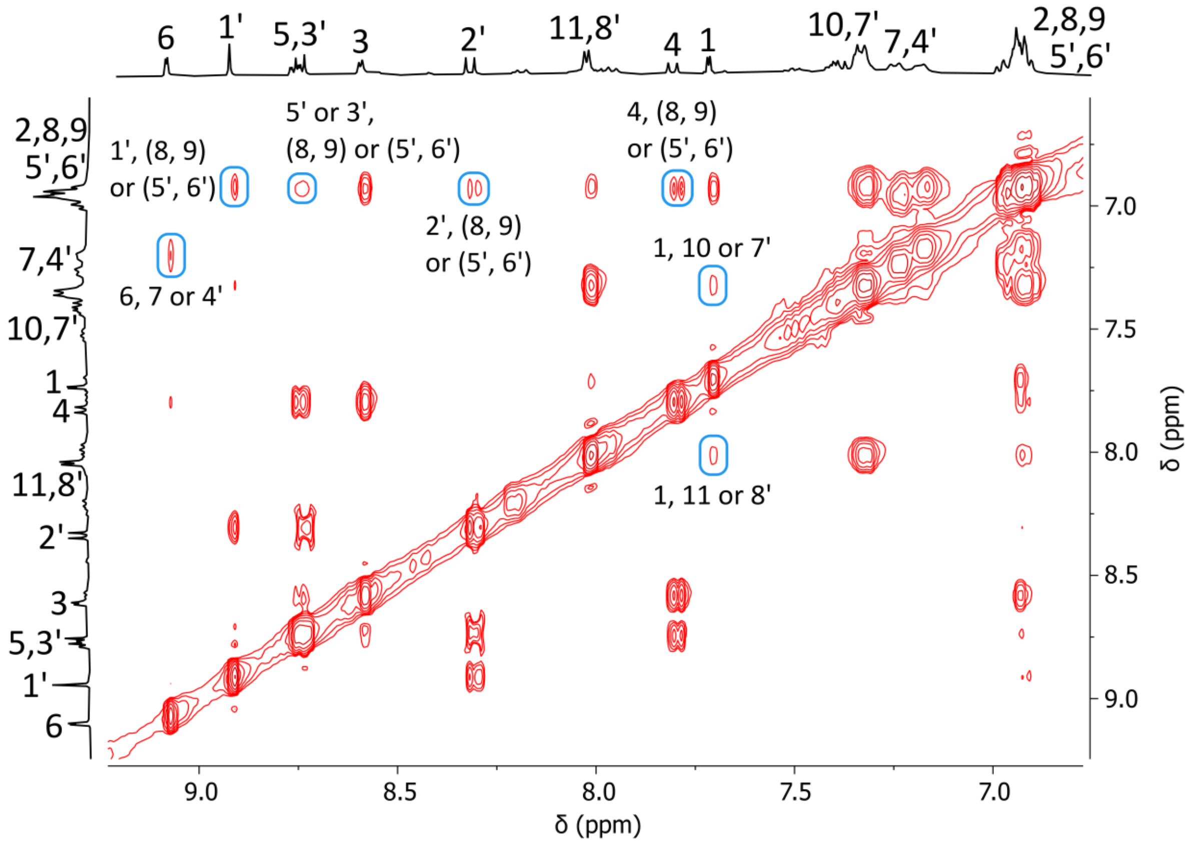

Figure S73 ^1H-^1H NOESY spectrum (400 MHz, [D_6]DMSO, 298 K) of [Pd(**DD--N'**)(**AA'--N'**)]$_2$(BF_4)$_4$. Only the correlations demonstrating dimerisation have been highlighted by blue squares and labelled. In most cases, analogous environments of the anthracene panels on each ligand overlap and so the exact environments producing each correlation cannot be precisely determined and instead denoted as 'proton x or proton y'. It is likely a through-space NOE correlation to both 'proton x' and 'proton y' could exist but irrespective of their exact identity, they can only be explained through the existence of a clippane.

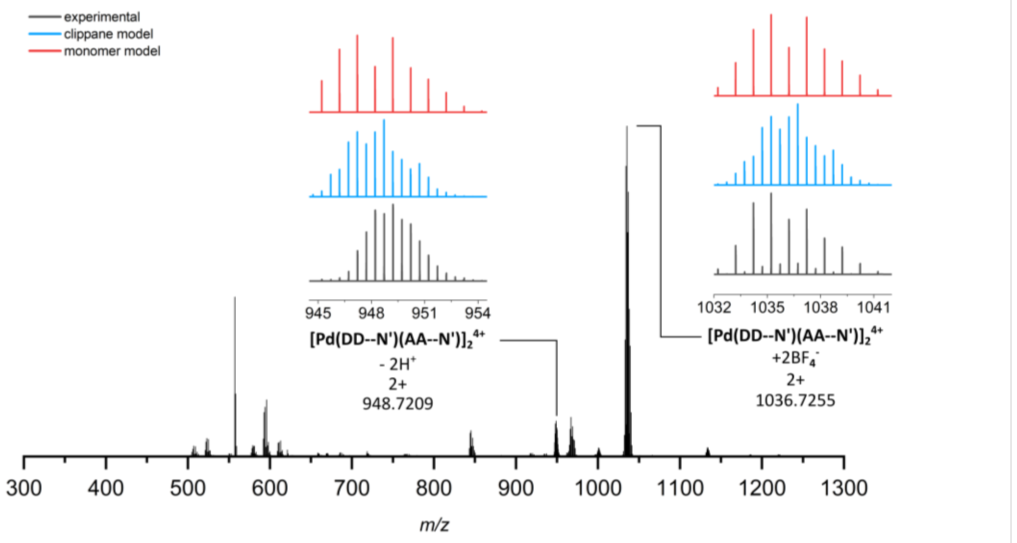

Figure S74 Nanospray HR MS (DMSO) of [Pd(**DD--N'**)(**AA--N'**)]$_2$(BF_4)$_4$. Note the observed *m/z* peaks corresponding to the clippane overlap with that of the monomer at half the respective charge. Models of the monomer (red) have been included for reference.

1.8. Non cyclic foldamers

1.8.1. N-AD'•AD-DA•DA'-N

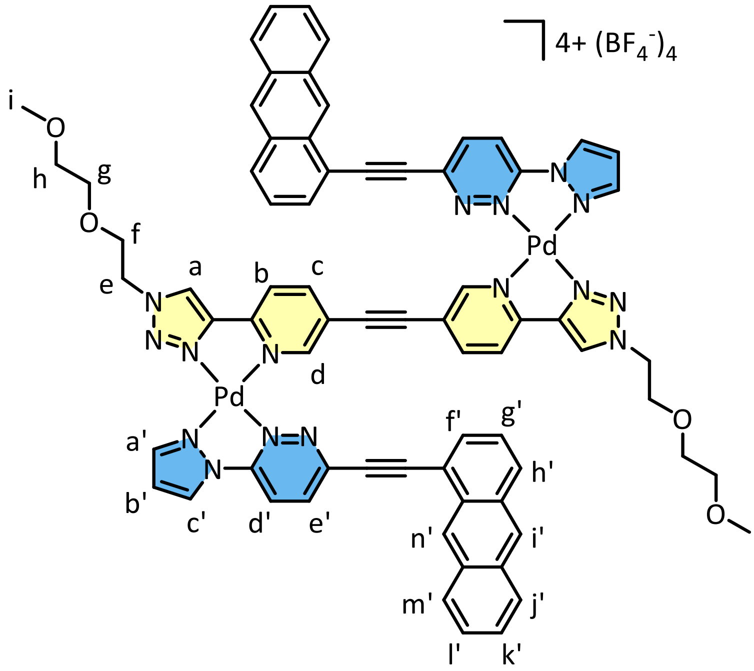

Using stock solutions, **DA'-N** (4.02 mg, 11.6 µmol), **AD-DA** (3.01 mg, 5.80 µmol) and $[Pd(CH_3CN)_4](BF_4)_2$ (5.16 mg, 11.6 µmol) were combined in $[D_6]$DMSO (550 µL). Equilibration occurred at room temperature overnight.

^{1}H NMR (400 MHz, $[D_6]$DMSO, 298 K) δ: *integration given per 'half' of the central ligand* 9.47 (1H, s, H_a), 9.21 (1H, br, $H_{c'}$), 9.07 (1H, d, J = 9.2 Hz, $H_{e'}$), 9.02 – 8.99 (2H, m, $H_{d',d}$), 8.66 (1H, br, $H_{a'}$), 8.47 (1H, s, $H_{n'}$), 8.33 (1H, d, J = 7.7 Hz, H_c), 8.13 (1H, s, $H_{i'}$), 8.01 (1H, d, J = 5.7 Hz, H_b), 7.85 (1H, d, J = 8.2 Hz, $H_{m'}$), 7.79 (1H, d, J = 8.8 Hz, $H_{h'}$), 7.70 (1H, d, J = 8.0 Hz, $H_{j'}$), 7.53 – 7.47 (3H, m, $H_{f',k',l'}$), 7.25 (1H, br, $H_{b'}$), 6.88 (1H, t, J = 6.9 Hz, $H_{g'}$), 5.12 – 510 (2H, m, H_e), 4.22 – 4.19 (2H, m, H_f), 3.81 – 3.79 (2H, m, H_g), 3.60 – 3.56 (2H, m, H_h, 3.26 (3H, s, H_i).

D ($\times 10^{-10}$ m^2 s^{-1}, 400 MHz, $[D_6]$DMSO, 298 K) = 0.92.

HR ESI-MS (DMSO/acetonitrile) m/z = 799.1472 [**N-AD'•AD-DA•DA'-N** + $2BF_4]^{2+}$ (calc. for $C_{72}H_{58}N_{16}O_4Pd_2B_2F_8^{2+}$, 799.1502).

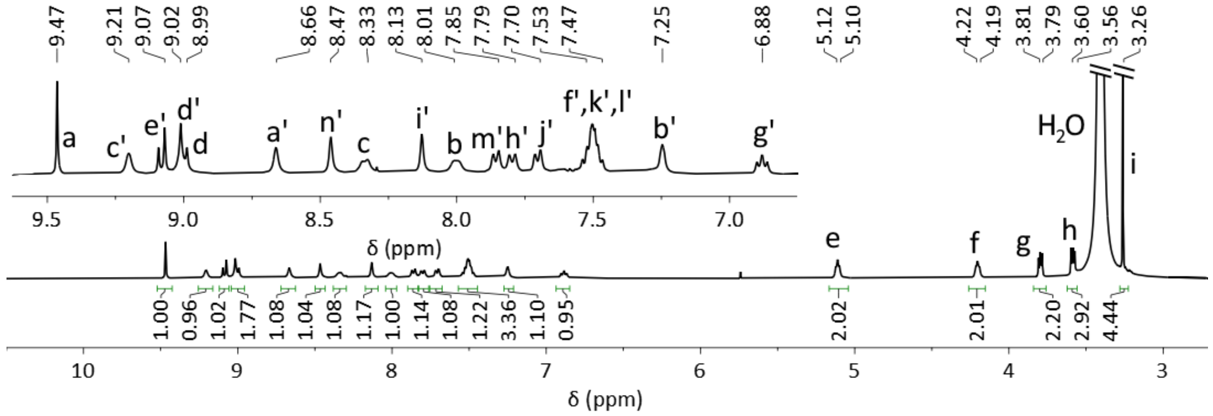

Figure S75 ^{1}H NMR spectrum (400 MHz, [D$_6$]DMSO, 298 K) of **N-AD'●AD-DA●DA'-N**.

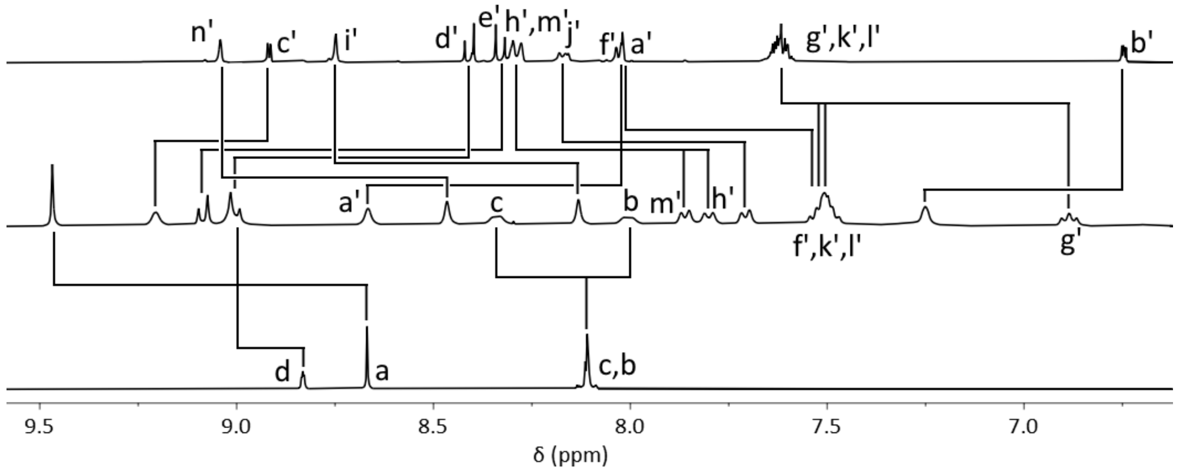

Figure S76 Partial ^{1}H NMR spectra (400 MHz, [D$_6$]DMSO, 298 K) of a) **DA'-N**, b) **N-AD'●AD-DA●DA'-N**, c) **AD-DA**.

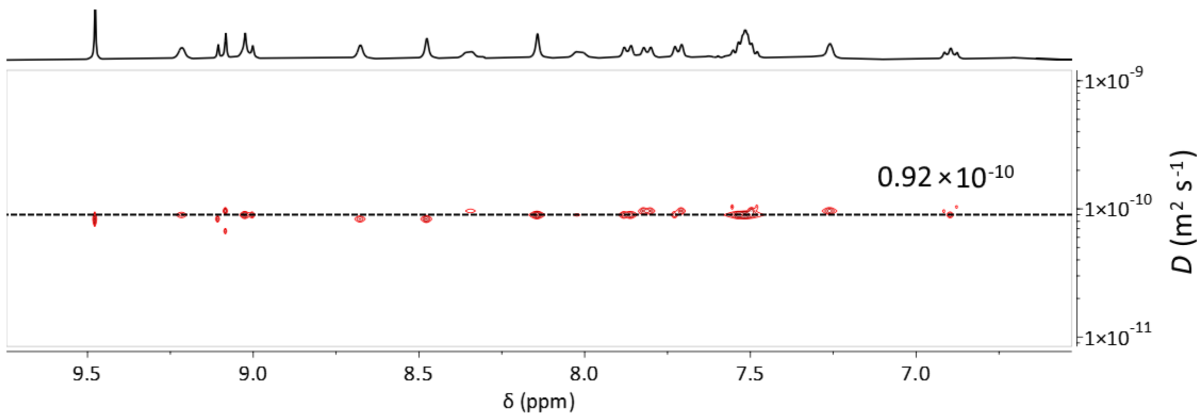

Figure S77 ^{1}H DOSY NMR spectrum (400 MHz, [D$_6$]DMSO, 298 K) of **N-AD'●AD-DA●DA'-N**.

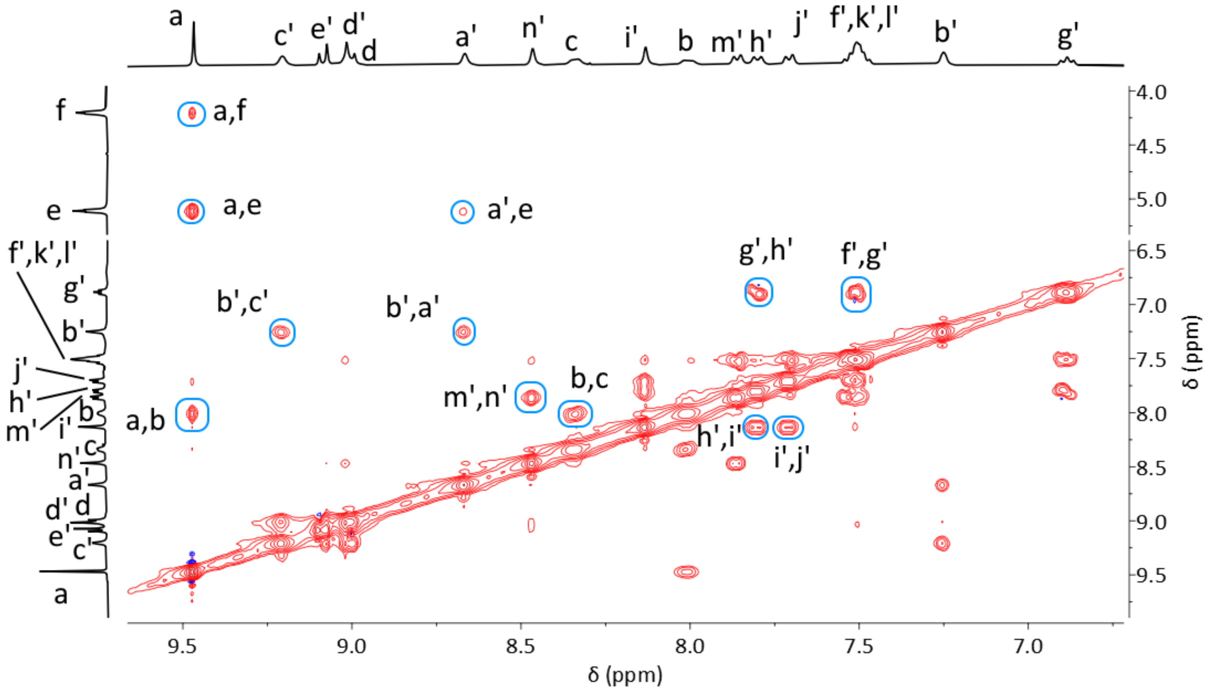

Figure S78 ^{1}H 2D NOESY NMR (400 MHz, $[D_6]$DMSO, 298 K, 200 ms) of **N-AD'●AD-DA●DA'-N**.

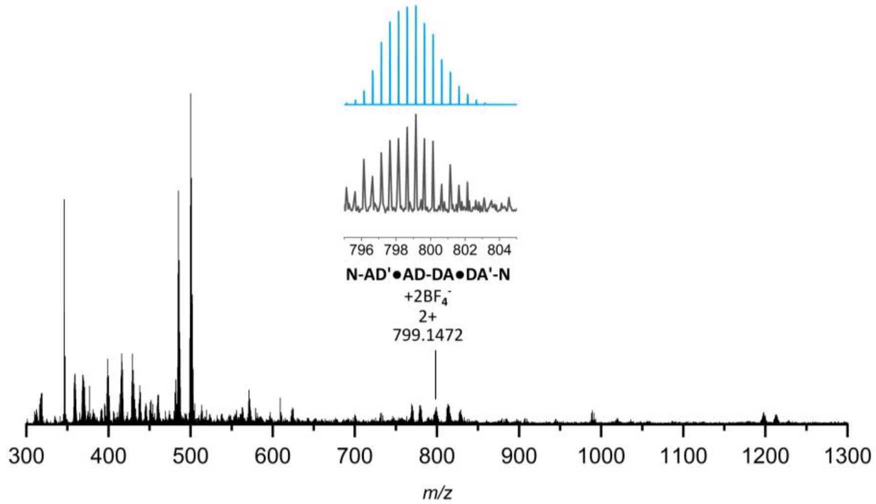

Figure S79 HR-ESI MS (DMSO/acetonitrile) of **N-AD'●AD-DA●DA'-N**.

1.8.2. N--AD'●AD--DA●DA'--N

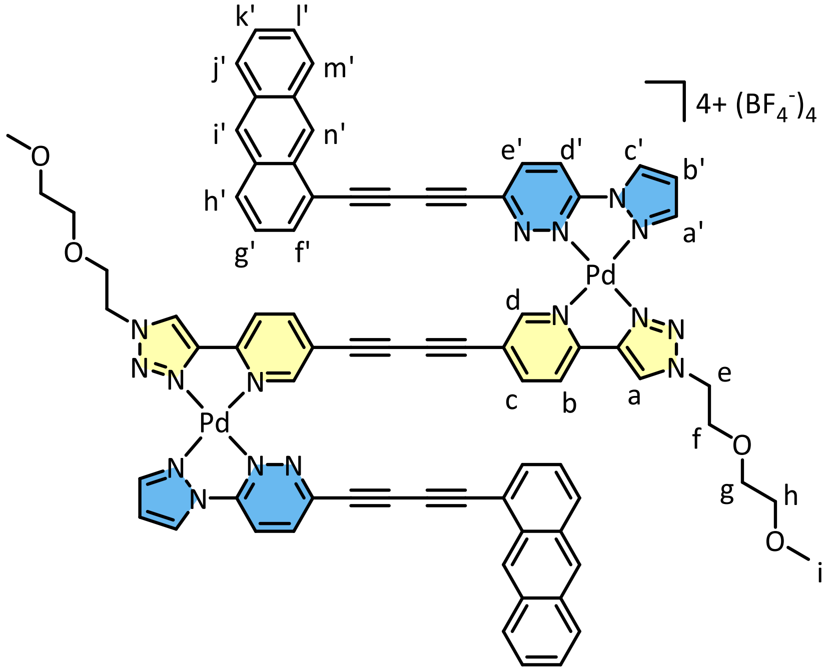

Using stock solutions, **DA'--N** (2.40 mg, 6.48 µmol), **AD--DA** (1.76 mg, 3.24 µmol) and $[Pd(CH_3CN)_4](BF_4)_2$ (2.88 mg, 6.48 µmol) were combined in $[D_6]$DMSO (550 µL). Equilibration occurred at room temperature overnight.

^{1}H NMR (400 MHz, $[D_6]$DMSO, 298 K) *integration given per 'half' of the central ligand δ*: 9.43 (1H, s, H_a), 9.31 (1H, d, J = 3.2 Hz, $H_{c'}$), 9.08 (1H, d, J = 9.1 Hz, $H_{e'}$), 8.93 – 8.90 (2H, m, $H_{d',d}$), 8.71 (1H, d, J = 2.4 Hz, $H_{a'}$), 8.44 – 8.41 (2H, m, $H_{c,n'}$), 8.23 (1H, s, $H_{i'}$), 8.00 (1H, d, J = 8.2 Hz, H_b), 7.81 – 7.78 (2H, m, $H_{h',m'}$), 7.70 – 7.68 (1H, m, $H_{j'}$), 7.59 (1H, d, J = 8.0 Hz, $H_{f'}$), 7.41 – 7.38 (2H, m, $H_{k',l'}$), 7.35 (1H, t, J = 2.3 Hz, $H_{b'}$), 7.04 (1H, dd, J = 8.6 Hz, 7.0 Hz, $H_{g'}$), 5.10 – 5.09 (2H, m, H_e), 4.21 – 4.18 (2H, m, H_f), 3.80 – 3.78 (2H, m, H_g), 3.58 – 3.56 (2H, m, H_h), 3.25 (3H, s, H_i).

D ($\times 10^{-10}$ m^2 s^{-1}, 400 MHz, $[D_6]$DMSO, 298 K) = 0.84.

HR ESI-MS (DMSO/acetonitrile) m/z = 498.4322 [**N--AD'●AD--DA●DA'--N** – H]$^{3+}$ (calc. for $C_{78}H_{57}N_{16}O_4Pd_2^{3+}$, 498.4284), m/z = 505.0948 [**N--AD'●AD--DA●DA'--N** + F]$^{3+}$ (calc. for $C_{78}H_{58}FN_{16}O_4Pd_2^{3+}$, 505.0972), m/z = 527.4368 [**N--AD'●AD--DA●DA'--N** + BF_4]$^{3+}$ (calc. for $C_{78}H_{58}BF_4N_{16}O_4Pd_2^{3+}$, 527.7656), m/z = 834.6525 [**N--AD'●AD--DA●DA'--N** + $2BF_4$]$^{2+}$ (calc. for $C_{78}H_{58}B_2F_8N_{16}O_4Pd_2^{2+}$, 834.6505).

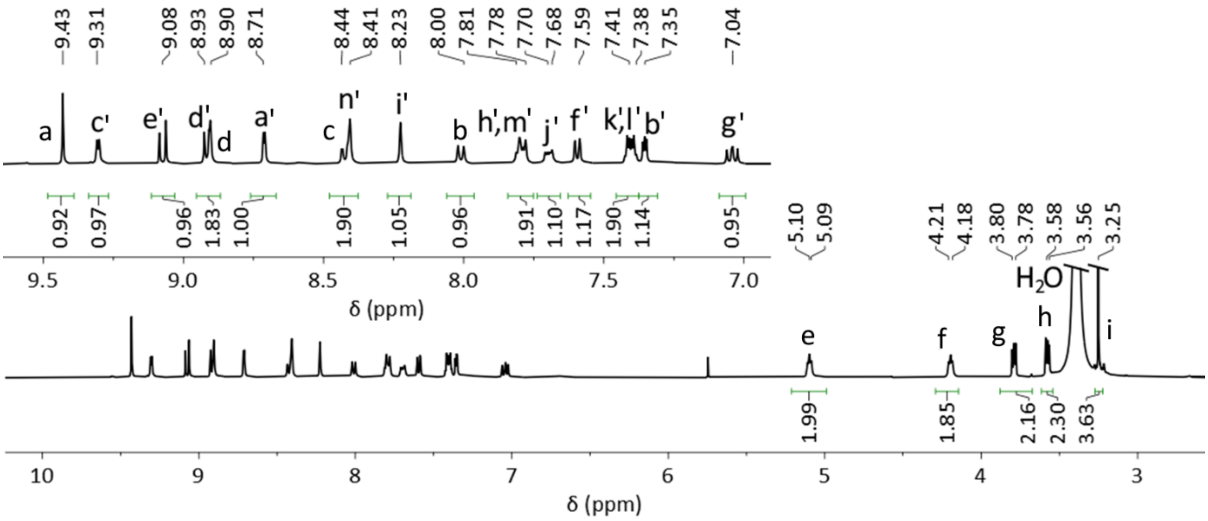

Figure S80 ^{1}H NMR spectrum (400 MHz, [D$_6$]DMSO, 298 K) of **N--AD'●AD--DA●DA'--N**.

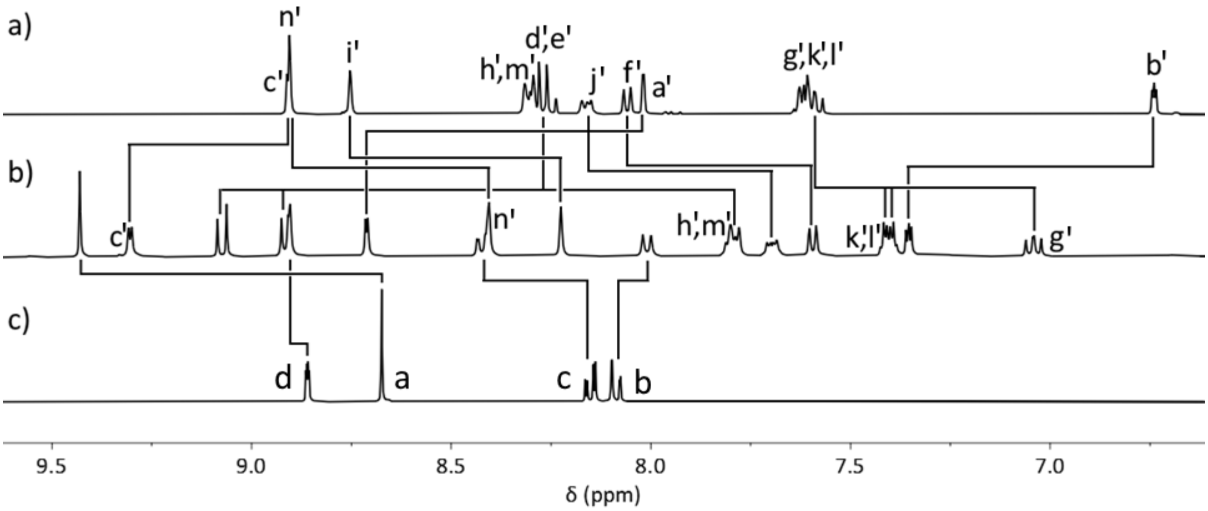

Figure S81 Partial ^{1}H NMR spectra (400 MHz, [D$_6$]DMSO, 298 K) of a) **DA'--N**, b) **N--AD'●AD--DA●DA'--N**, c) **AD--DA**.

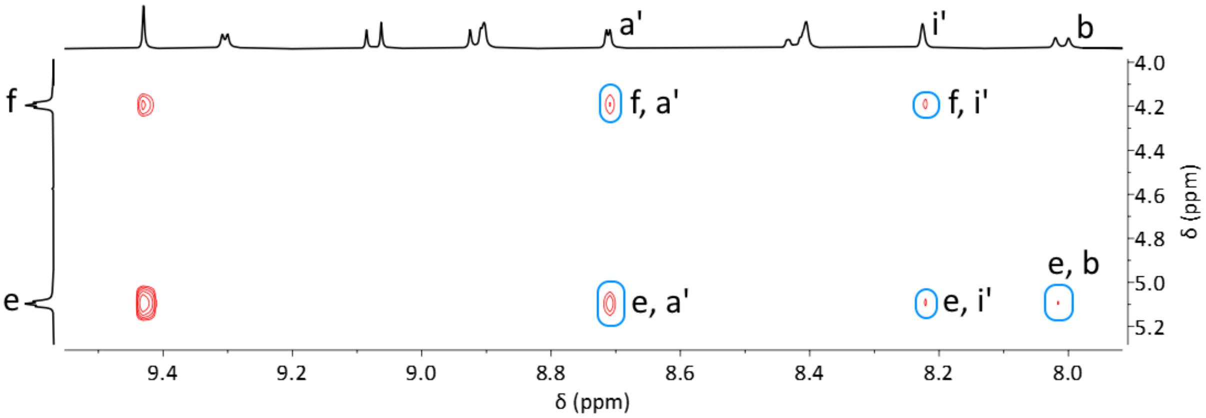

Figure S82 ^{1}H 2D NOESY NMR (400 MHz, [D$_6$]DMSO, 298 K, 200 ms) of **N--AD'●AD--DA●DA'--N**.

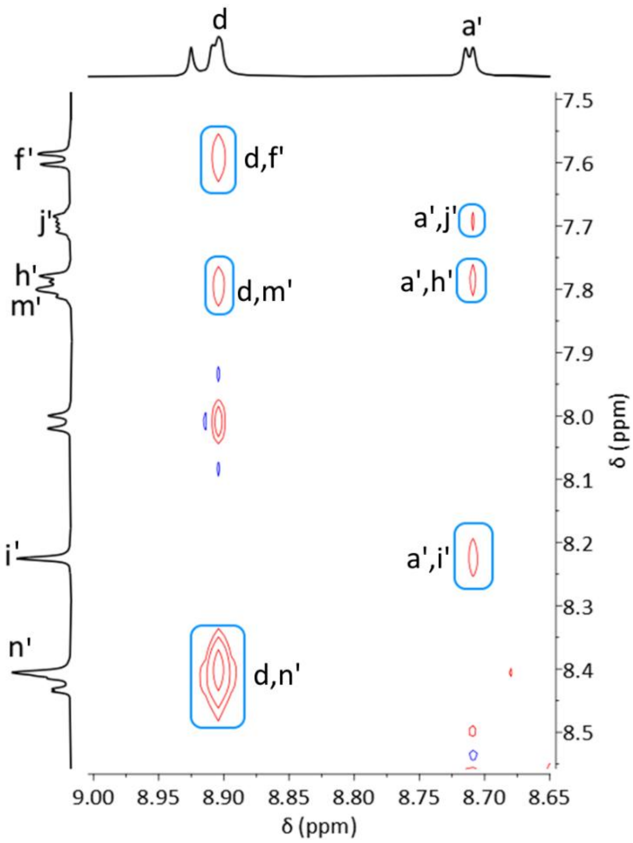

Figure S83 ^{1}H 2D NOESY NMR (400 MHz, [D_6]DMSO, 298 K, 200 ms) of **N--AD'●AD--DA●DA'--N**.

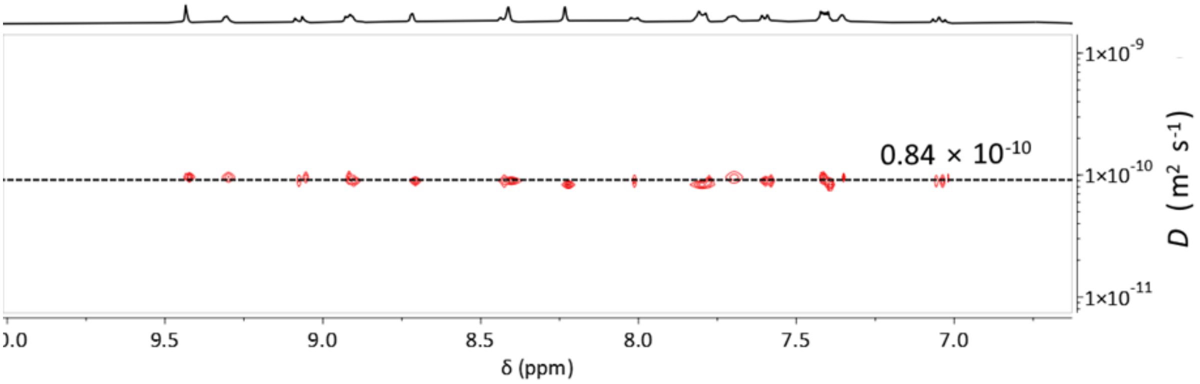

Figure S84 ^{1}H DOSY NMR spectrum (400 MHz, [D_6]DMSO, 298 K) of **N--AD'●AD--DA●DA'--N**.

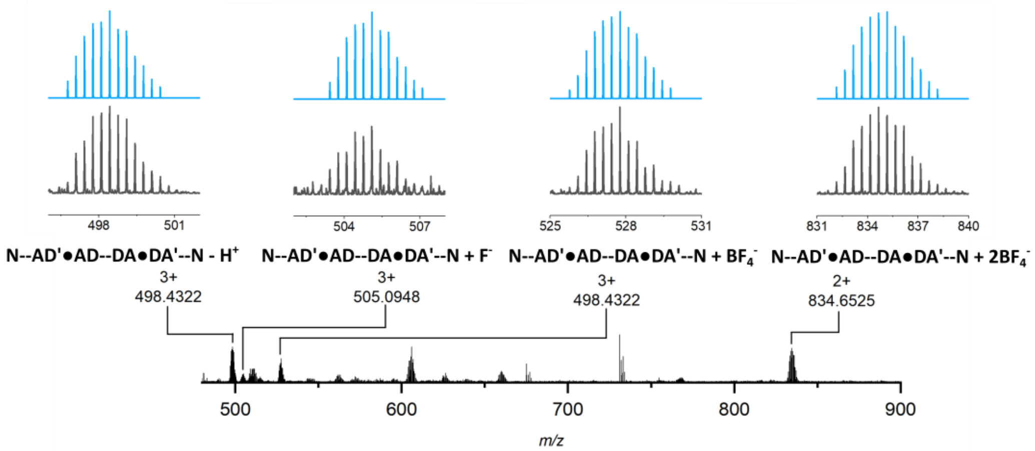

Figure S85 HR-ESI MS (DMSO/acetonitrile) of **N--AD'●AD--DA●DA'--N**.

1.8.3. N-AD'●AD--DA●DA'-N

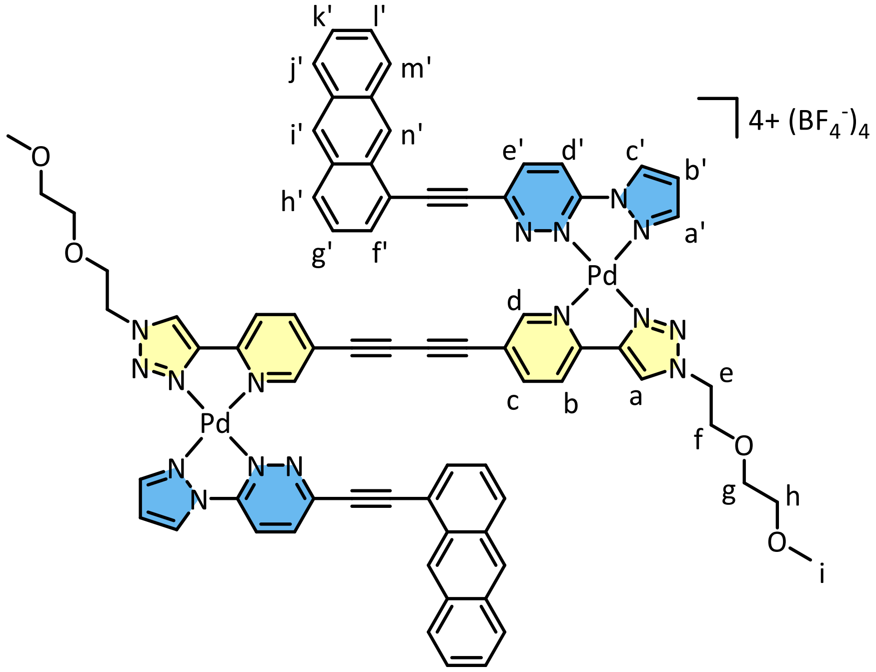

Using stock solutions, **DA'-N** (2.87 mg, 7.75 µmol), **AD--DA** (2.10 mg, 3.87 µmol) and $[Pd(CH_3CN)_4](BF_4)_2$ (3.44 mg, 7.75 µmol) were combined in $[D_6]$DMSO (500 µL). Equilibration occurred at room temperature overnight.

^{1}H NMR (400 MHz, $[D_6]$DMSO, 298 K) *integration given per 'half' of the central ligand δ*: 9.50 (1H, s, H_a), 9.27 (1H, br, $H_{c'}$), 8.99 (1H, d, *J* = 8.6 Hz, $H_{e'}$), 8.83 (1H, d, *J* = 8.6 Hz, $H_{d'}$), 8.60 (1H, d, *J* = 8.0 Hz, H_c), 8.50 – 8.42 (3H, m, $H_{b,a',n'}$), 8.28 - 8.18 (2H, m, $H_{i',d}$), 8.00 (1H, d, *J* = 8.2 Hz, H_b), 7.92 (1H, d, *J* = 6.8 Hz, $H_{h'}$), 7.85 (1H, br, $H_{m'}$), 7.57 – 7.43 (2H, m, $H_{f',j'}$), 7.39 – 7.25 (3H, m, $H_{b',k',l'}$), 7.07 (1H, t, *J* = 7.5 Hz, $H_{g'}$), 5.07 (2H, t, *J* = 4.5 Hz, H_e), 4.15 (2H, t, *J* = 4.6 Hz, H_f), 3.78– 3.75 (2H, m, H_g), 3.59 – 3.56 (2H, m, H_h), 3.30 (3H, s, H_i).

D (× 10^{-10} m^2 s^{-1}, 400 MHz, $[D_6]$DMSO, 298 K) = 0.88.

HR ESI-MS (DMSO/acetonitrile) *m/z* = 482.4315 [**N-AD'●AD--DA●DA'-N** – H]$^{3+}$ (calc. for $C_{74}H_{57}N_{16}O_4Pd_2^{3+}$, 482.4284), *m/z* = 511.7688 [**N-AD'●AD--DA●DA'-N** + BF_4]$^{3+}$ (calc. for $C_{74}H_{58}BF_4N_{16}O_4Pd_2^{3+}$, 511.7656), *m/z* = 811.1541 [**N-AD'●AD--DA●DA'-N** + $2BF_4$]$^{2+}$ (calc. for $C_{74}H_{58}B_2F_8N_{16}O_4Pd_2^{2+}$, 811.1502).

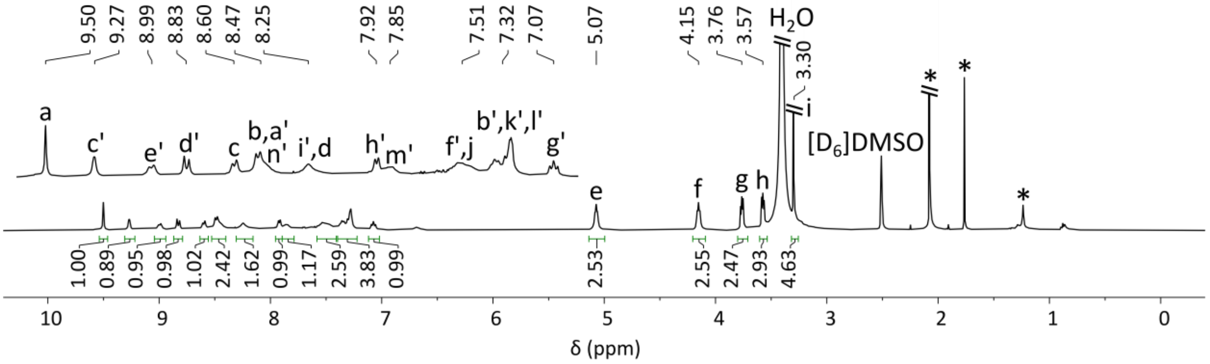

Figure S86 ^1H NMR spectrum (400 MHz, [D$_6$]DMSO, 298 K) of **N-AD'●AD--DA●DA'-N**. Impurities from the Pd(II) source are indicated by *.

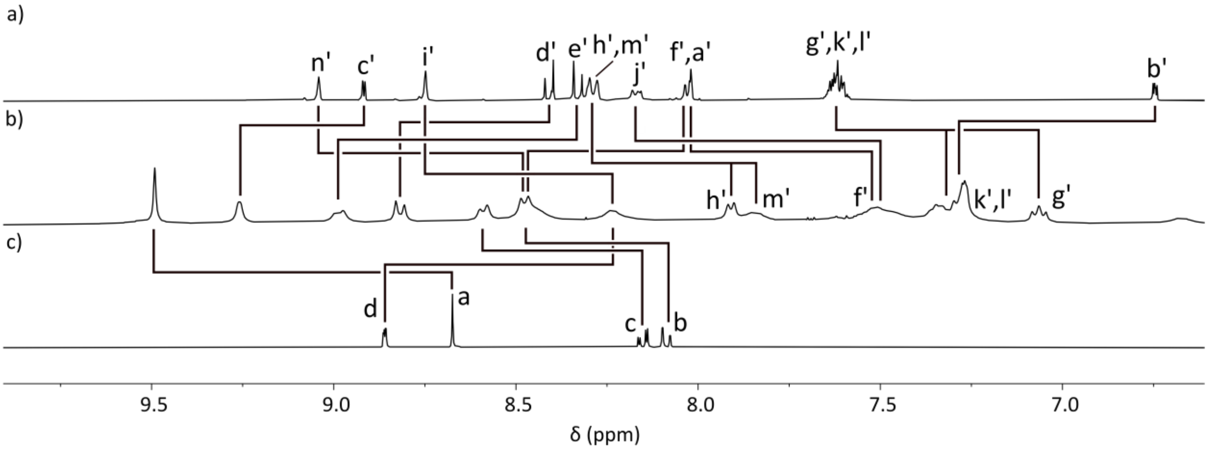

Figure S87 Partial ^1H NMR spectra (400 MHz, [D$_6$]DMSO, 298 K) of a) **DA'-N**, b) **N-AD'●AD--DA●DA'-N**, c) **AD--DA**.

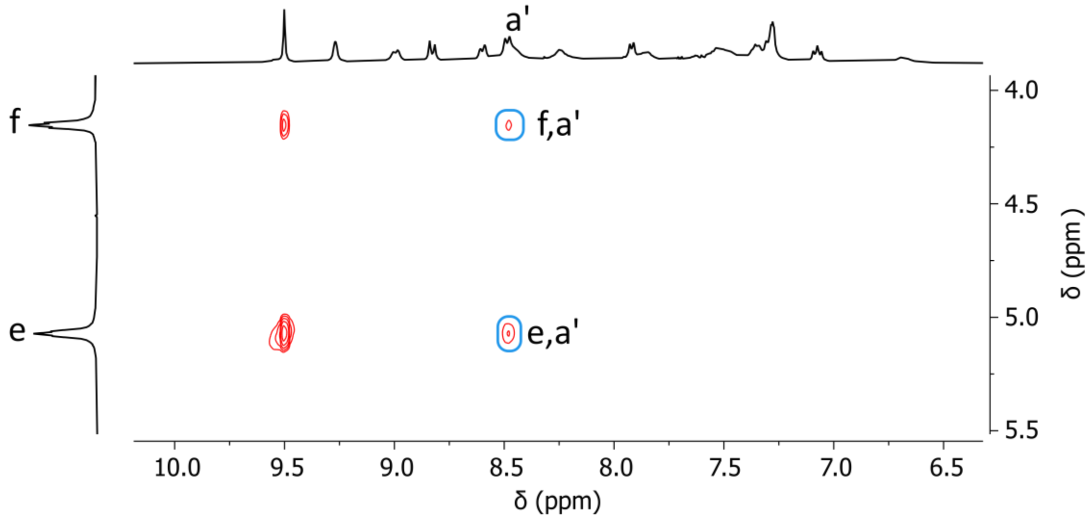

Figure S88 ^1H 2D NOESY NMR (400 MHz, [D$_6$]DMSO, 298 K, 200 ms) of **N-AD'●AD--DA●DA'-N**.

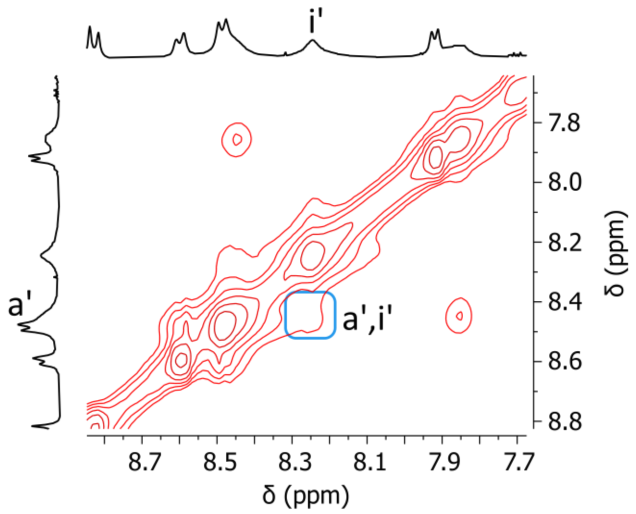

Figure S89 ^{1}H 2D NOESY NMR (400 MHz, [D$_6$]DMSO, 298 K, 200 ms) of **N-AD'●AD--DA●DA'-N**.

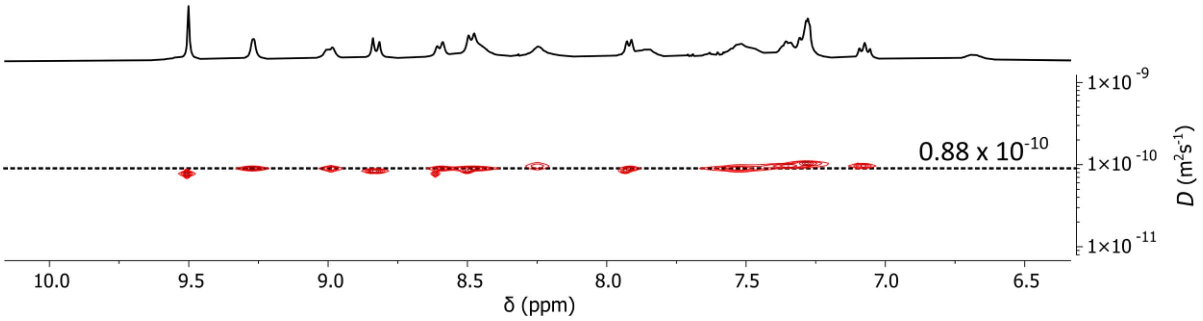

Figure S90 ^{1}H DOSY NMR spectrum (400 MHz, [D$_6$]DMSO, 298 K) of **N-AD'●AD--DA●DA'-N**.

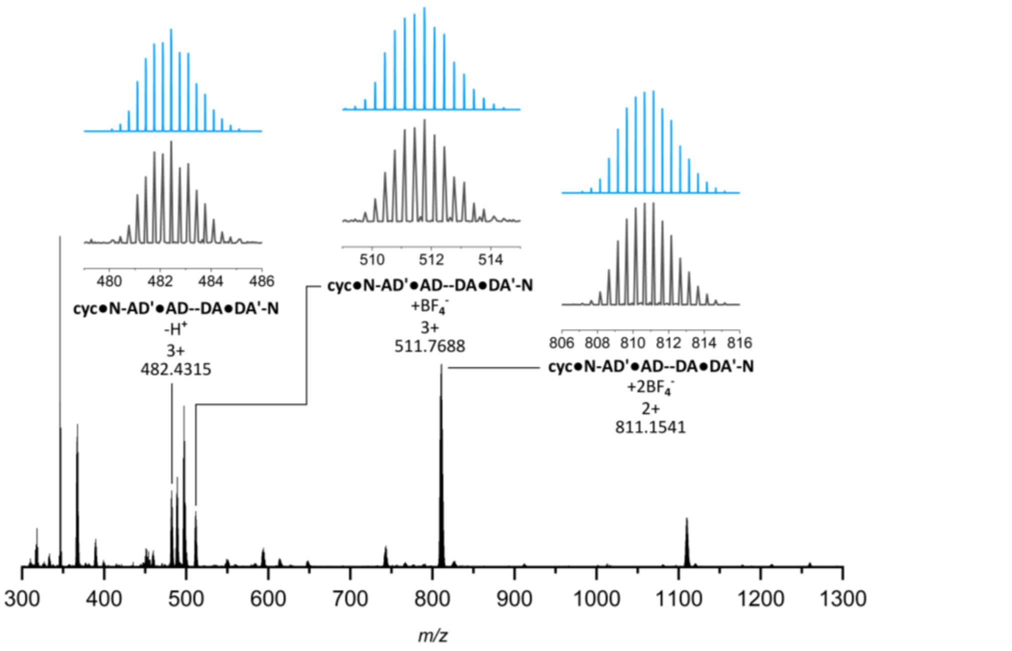

Figure S91 HR-ESI MS (DMSO/acetonitrile) of **cyc●N-AD'●AD--DA●DA'-N**.

1.8.4. EP-DD●AA-N-AA●DD-EP

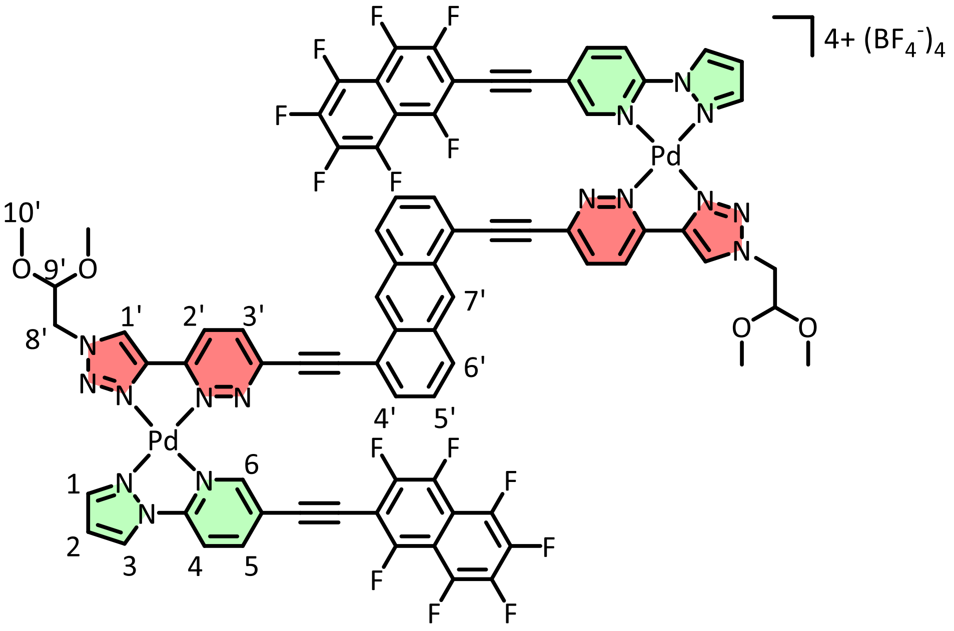

Using stock solutions, **DD-EP** (1.90 mg, 4.50 µmol), **AA-N-AA** (1.56 mg, 2.25 µmol) and $[Pd(CH_3CN)_4](BF_4)_2$ (2.00 mg, 4.50 µmol) were combined in $[D_6]$DMSO (550 µL). Equilibration occurred in the time taken to run an NMR spectrum.

^{1}H NMR (400 MHz, $[D_6]$DMSO, 298 K) *integration given per 'half' of the central ligand δ*: 9.93 (1H, br, H_6), 9.63 (1H, s, $H_{1'}$), 9.57 (1H, d, *J* = 3.0 Hz, H_3), 9.26 (1H, d, *J* = 8.7 Hz, $H_{2'}$), 9.00 (1H, d, *J* = 8.7 Hz, $H_{3'}$), 8.95 (1H, d, *J* = 8.8 Hz, H_5), 8.88 (1H, d, *J* = 2.7 Hz, H_1), 8.68 (1H, d, *J* = 8.8 Hz, H_4), 8.37 (1H, s, $H_{7'}$), 8.09 (1H, d, *J* = 6.9 Hz, $H_{4'}$), 8.03 (1H, d, *J* = 8.8 Hz, $H_{6'}$), 7.57 (1H, t, *J* = 7.9 Hz, $H_{5'}$), 7.37 (1H, t, *J* = 2.7 Hz, H_2), 5.15 (2H, d, *J* = 4.2 Hz, $H_{8'}$), 5.04 (1H, t, *J* = 4.2 Hz, $H_{9'}$), 3.48 (6H, s, $H_{10'}$).

D ($\times 10^{-10}$ m^2 s^{-1}, 400 MHz, $[D_6]$DMSO, 298 K) = 0.85.

HR ESI-MS (DMSO/acetonitrile) *m/z* = 437.0457 $[$**EP-DD●AA-N-AA●DD-EP**$]^{4+}$ (calc. for $C_{78}H_{44}N_{16}O_4F_{14}Pd_2^{4+}$, 437.0403), *m/z* = 589.0577 $[$**EP-DD●AA-N-AA●DD-EP** + $F^-]^{3+}$ (calc. for $C_{78}H_{44}N_{16}O_4F_{15}Pd_2^{3+}$, 589.0532), *m/z* = 960.5901 $[$**EP-DD●AA-N-AA●DD-EP** + $2BF_4^-]^{2+}$ (calc. for $C_{78}H_{44}N_{16}O_4B_2F_{22}Pd_2^{3+}$, 960.5845).

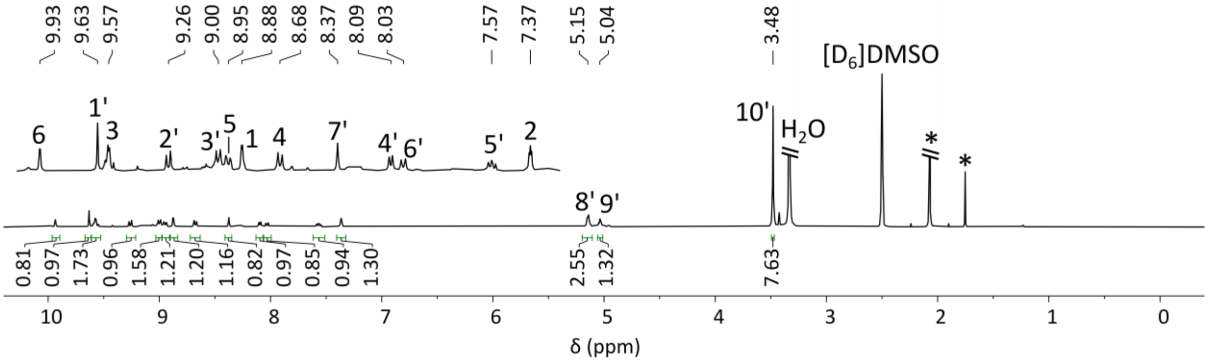

Figure S92 ^{1}H NMR spectrum (400 MHz, $[D_6]$DMSO, 298 K) of **EP-DD●AA-N-AA●DD-EP**. Impurities from the Pd(II) source are indicated by *.

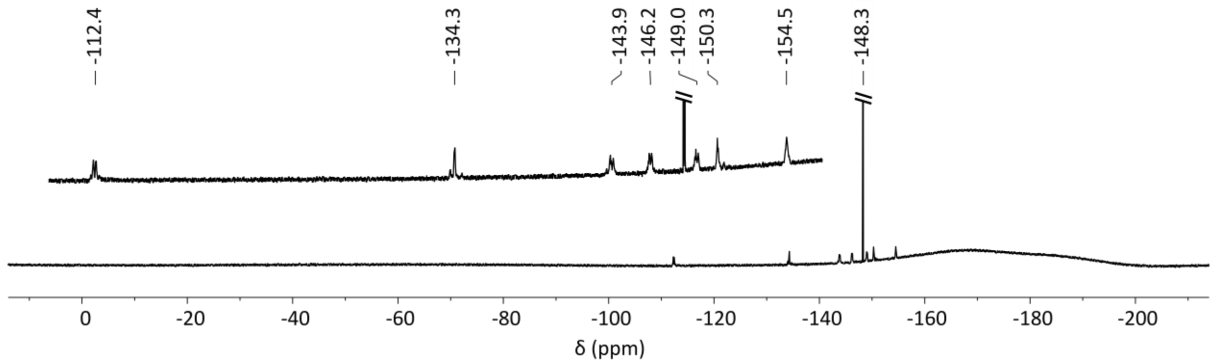

Figure S93 ^{19}F NMR spectrum (376 MHz, [D$_6$]DMSO, 298 K) of **EP-DD●AA-N-AA●DD-EP**.

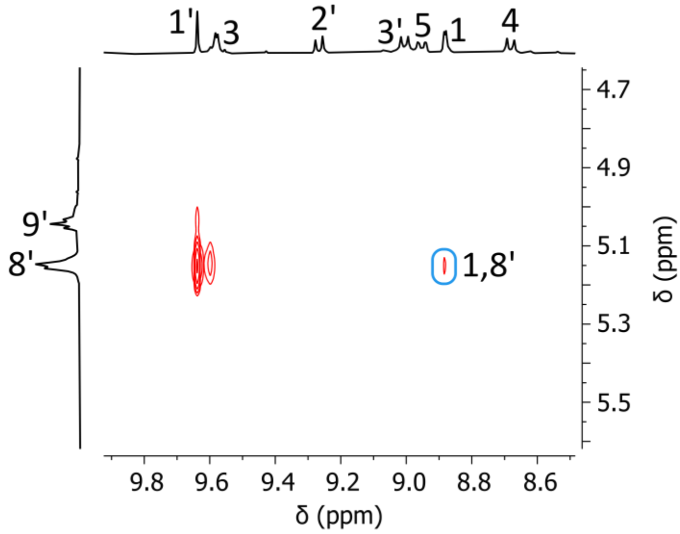

Figure S94 ^{1}H 2D NOESY NMR (400 MHz, [D$_6$]DMSO, 298 K, 200 ms) of **EP-DD●AA-N-AA●DD-EP**.

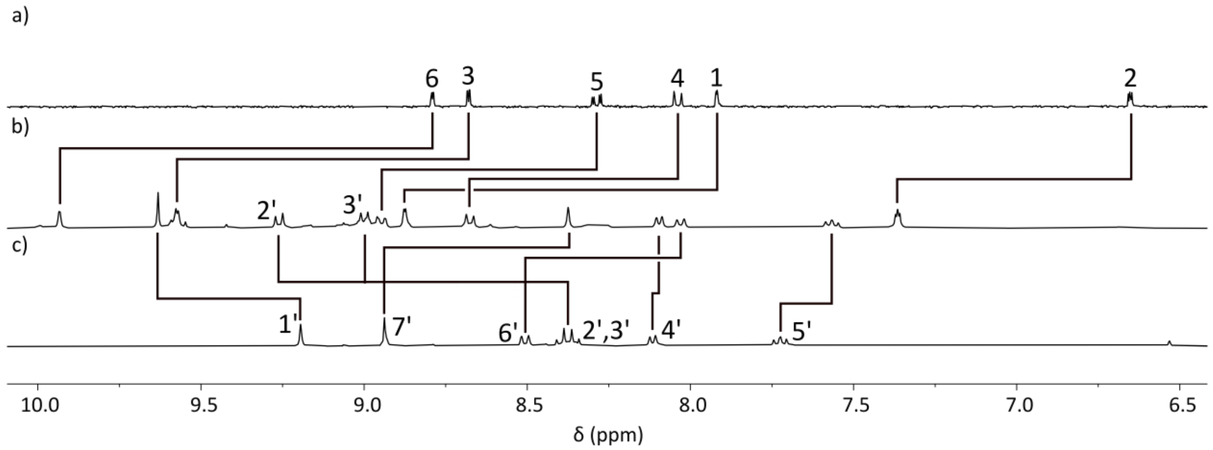

Figure S95 Partial ^{1}H NMR spectra (400 MHz, [D$_6$]DMSO, 298 K) of a) **DD-EP**, b) **EP-DD●AA-N-AA●DD-EP**, c) **AA-N-AA**.

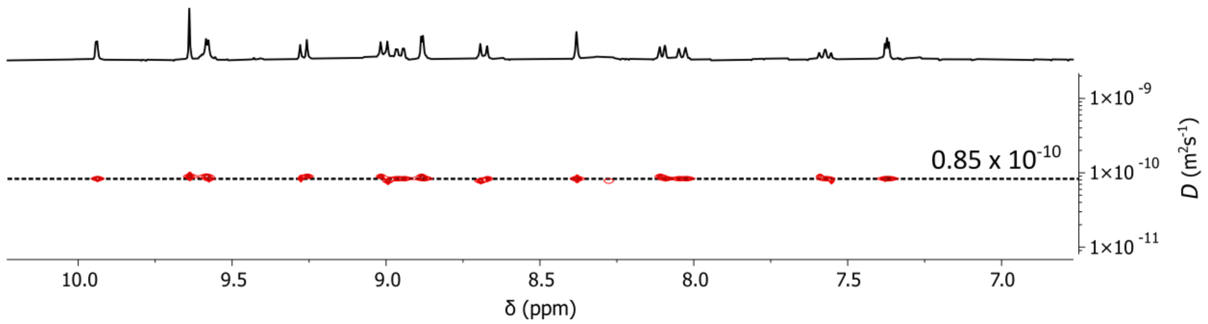

Figure S96 ^{1}H DOSY NMR spectrum (400 MHz, [D_6]DMSO, 298 K) of **EP-DD●AA-N-AA●DD-EP**.

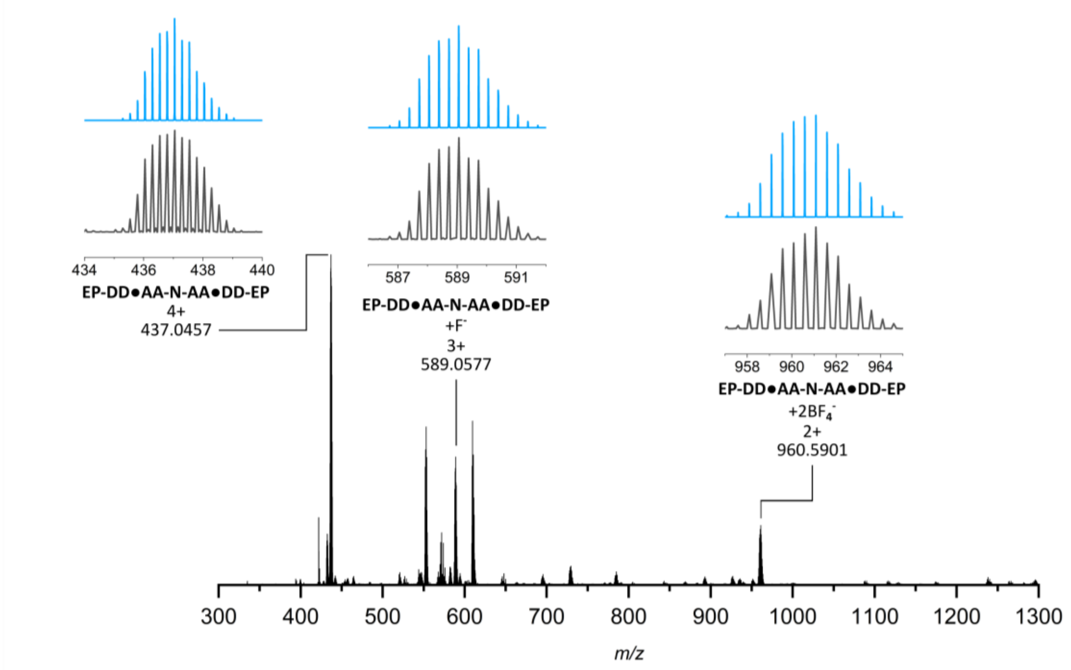

Figure S97 HR-ESI MS (DMSO/acetonitrile) of **EP-DD●AA-N-AA●DD-EP**.

1.8.5. EP-AA●DD-NN-DD●AA-EP

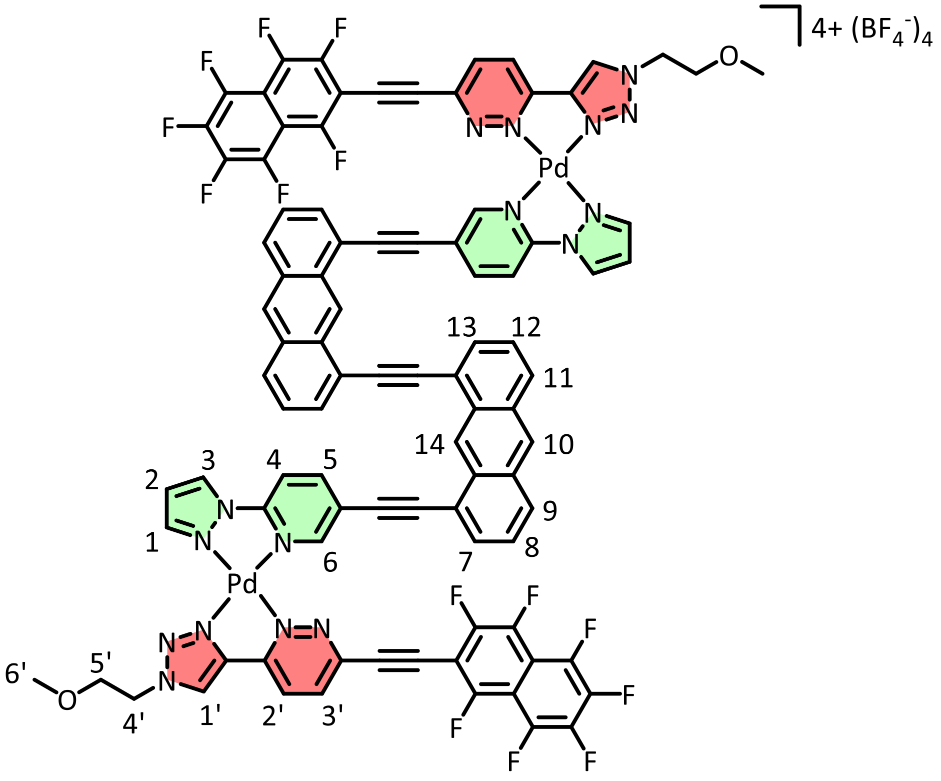

Using stock solutions, **AA-EP** (2.17 mg, 4.50 μmol), **DD-NN-DD** (1.61 mg, 2.25 μmol) and $[Pd(CH_3CN)_4](BF_4)_2$ (2.00 mg, 4.50 μmol) were combined in $[D_6]$DMSO (550 μL). Equilibration occurred in the time taken to run an NMR spectrum.

^{1}H NMR (400 MHz, $[D_6]$DMSO, 298 K) *integration given per 'half' of the central ligand δ*: 9.63 (1H, s, $H_{1'}$), 9.25 (1H, d, *J* = 3.0 Hz, H_3), 9.14 (1H, s, H_6), 8.94 (1H, d, *J* = 8.8 Hz, $H_{2'}$), 8.78 (1H, dd, *J* = 8.7 Hz, 2.0 Hz, H_5), 8.74-8.71 (2H, m, $H_{3',1}$), 8.67 (1H, s, H_{14}), 8.00-7.96 (2H, m, $H_{4,10}$), 7.73 (1H, dd, *J* = 5.5 Hz, 2.4 Hz, H_{13}), 7.51 (1H, d, *J* = 6.9 Hz, H_7), 7.39 (1H, d, *J* = 8.6 Hz, H_9), 7.36 (1H, t, *J* = 2.7 Hz, H_2), 7.30-7.25 (2H, m, $H_{11,12}$), 7.11 (1H, dd, *J* = 8.4 Hz, 7.1 Hz, H_8), 5.18 (2H, t, *J* = 4.7 Hz, $H_{4'}$), 4.13-4.09 (2H, m, $H_{5'}$), 3.48 (3H, s, $H_{6'}$).

D ($\times 10^{-10}$ m^2 s^{-1}, 400 MHz, $[D_6]$DMSO, 298 K) = 0.90.

HR ESI-MS (DMSO/acetonitrile) *m/z* = 472.0539 [**EP-AA●DD-NN-DD●AA-EP**]$^{4+}$ (calc. for $C_{92}H_{48}N_{16}O_2F_{14}Pd_2^{4+}$, 472.0509), *m/z* = 658.4019 [**EP-AA●DD-NN-DD●AA-EP** + BF_4^-]$^{3+}$ (calc. for $C_{92}H_{48}N_{16}O_2BF_{18}Pd_2^{3+}$, 658.4023), *m/z* = 1031.1014 [**EP-AA●DD-NN-DD●AA-EP** + $2BF_4^-$]$^{2+}$ (calc. for $C_{92}H_{48}N_{16}O_2B_2F_{22}Pd_2^{2+}$, 1031.1053).

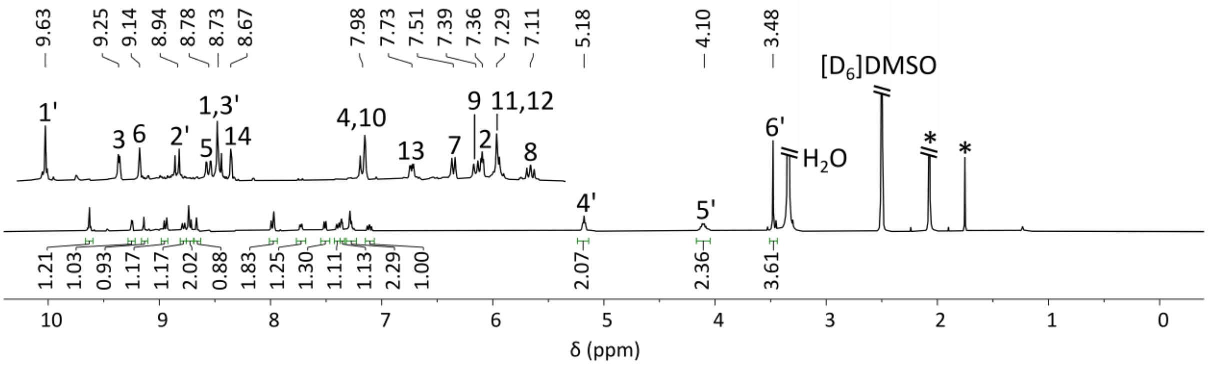

Figure S98 ^{1}H NMR spectrum (400 MHz, [D_6]DMSO, 298 K) of **EP-AA●DD-NN-DD●AA-EP**. Impurities from the Pd(II) source are indicated by *.

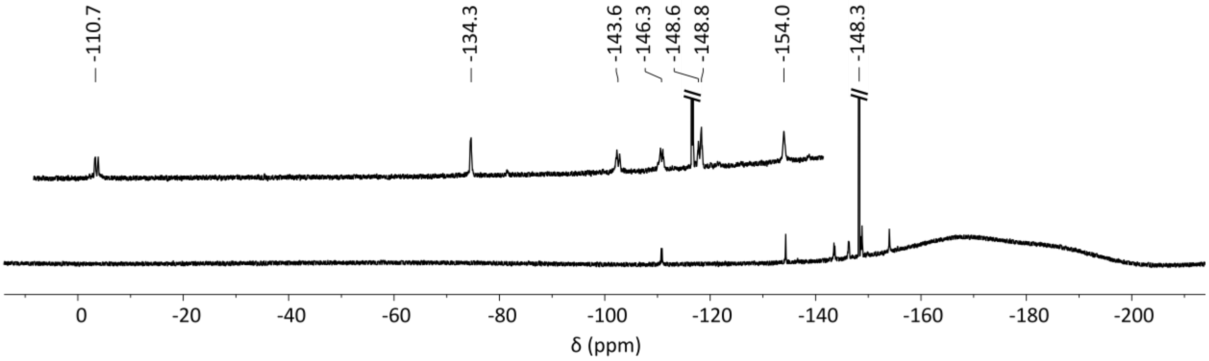

Figure S99 ^{19}F NMR spectrum (376 MHz, [D_6]DMSO, 298 K) of **EP-AA●DD-NN-DD●AA-EP**.

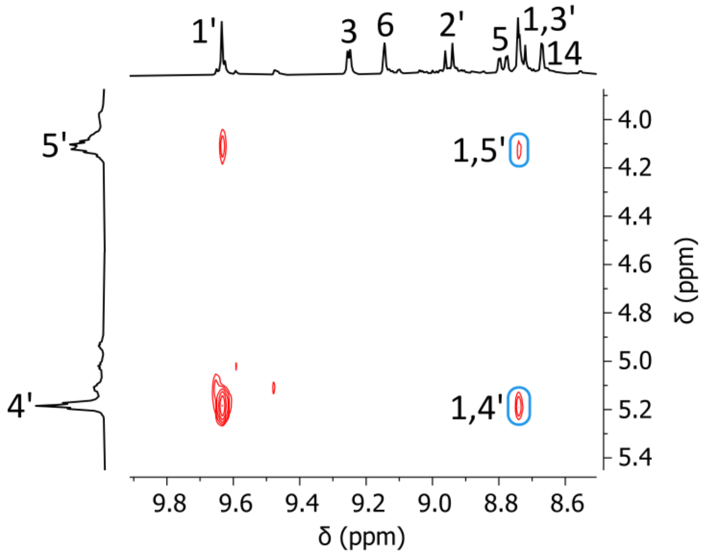

Figure S100 ^{1}H 2D NOESY NMR (400 MHz, [D_6]DMSO, 298 K, 200 ms) of **EP-AA●DD-NN-DD●AA-EP**.

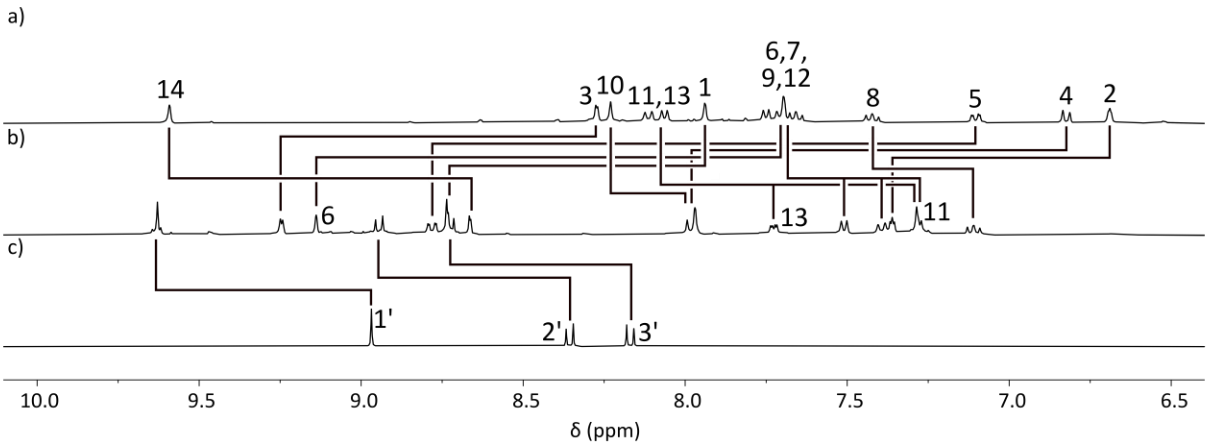

Figure S101 Partial ^{1}H NMR spectra (400 MHz, [D_6]DMSO, 298 K) of a) **DD-NN-DD**, b) **EP-AA●DD-NN-DD●AA-EP**, c) **AA-EPs**.

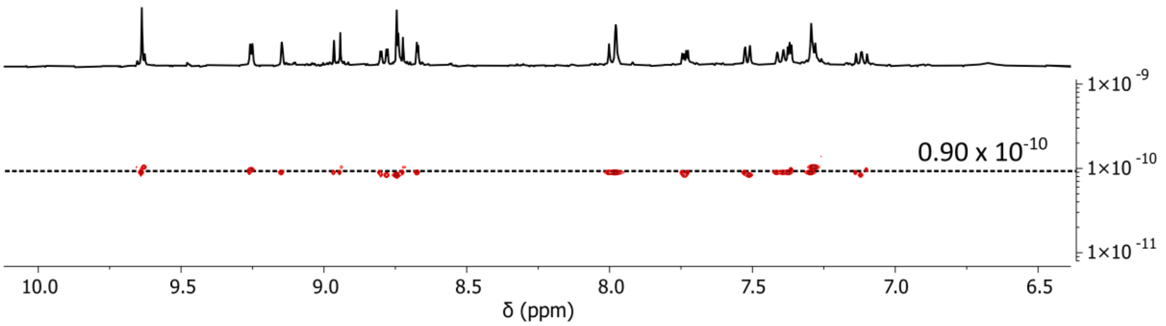

Figure S102 ^{1}H DOSY NMR spectrum (400 MHz, [D_6]DMSO, 298 K) of **EP-AA●DD-NN-DD●AA-EP**.

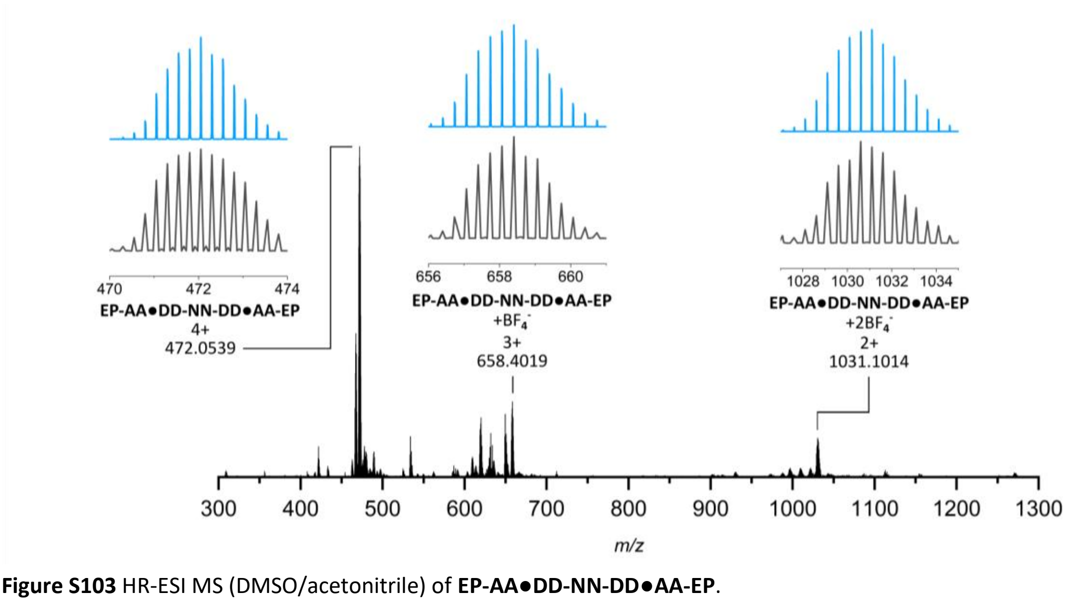

Figure S103 HR-ESI MS (DMSO/acetonitrile) of **EP-AA●DD-NN-DD●AA-EP**.

1.9. Macrocycles

1.9.1. *cyc*●AD--DA●DA'-N-AD'

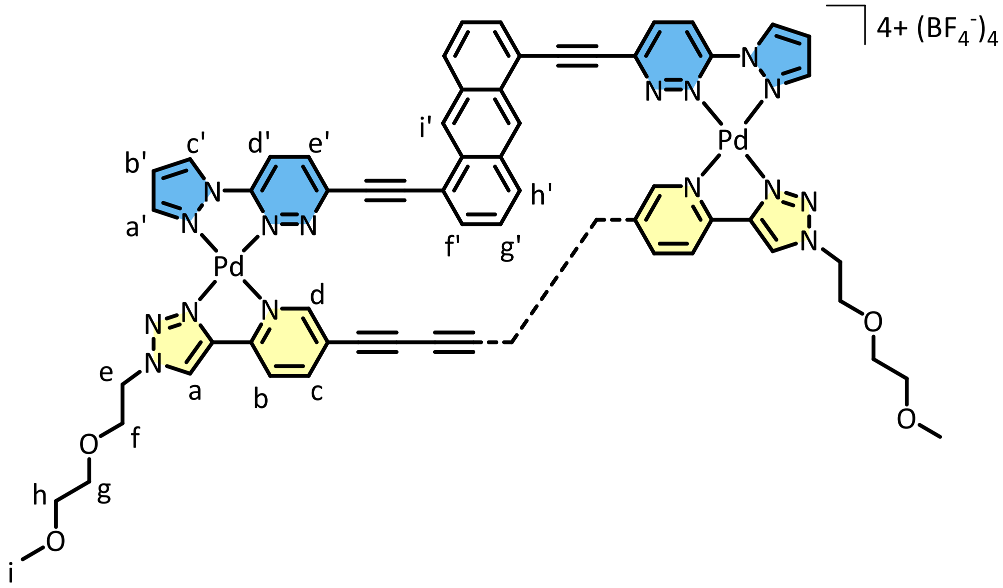

Using stock solutions, **DA'-N-AD'** (1.14 mg, 1.93 µmol), **AD--DA** (1.20 mg, 2.21 µmol) and $[Pd(CH_3CN)_4](BF_4)_2$ (1.96 mg, 4.42 µmol) were combined in $[D_6]$DMSO (550 µL). Equilibration occurred in the time taken to run an NMR spectrum.

^{1}H NMR (400 MHz, $[D_6]$DMSO, 298 K) *integration given per 'half' of the central ligand δ*: 9.65 (1H, br, H_d), 9.57 (1H, d, J = 3.2 Hz, $H_{c'}$), 9.50 (1H, s, H_a), 9.17-9.16 (1H, m, $H_{i'}$), 9.12 (1H, d, J = 4.7 Hz, $H_{d'}$), 9.03 (1H, d, J = 9.2 Hz, $H_{e'}$), 8.95 (1H, d, J = 2.2 Hz, $H_{a'}$), 8.51 (1H, d, J = 8.4 Hz, H_b), 8.37 (1H, dd, J = 8.2 Hz, 1.7 Hz, H_c), 8.15 (1H, d, J = 7.0 Hz, $H_{f'}$), 7.93 (1H, d, J = 8.8 Hz, $H_{h'}$), 7.46 (1H, dd, J = 8.5 Hz, 7.1 Hz, $H_{g'}$), 7.40-7.38 (1H, m, $H_{b'}$), 5.04-5.01 (2H, m, H_e), 4.07-4.05 (2H, m, H_f), 3.66-3.64 (2H, m, H_g), 3.48-3.45 (2H, m, H_h), 3.23 (3H, s, H_i).

D ($\times 10^{-10}$ m^2 s^{-1}, 400 MHz, $[D_6]$DMSO, 298 K) = 0.91.

HR ESI-MS (DMSO/acetonitrile) m/z = 423.0875 [**cyc●AD--DA●DA'-N-AD'** – H^+]$^{3+}$ (calc. for $C_{60}H_{47}N_{16}O_4Pd_2$, 423.0688).

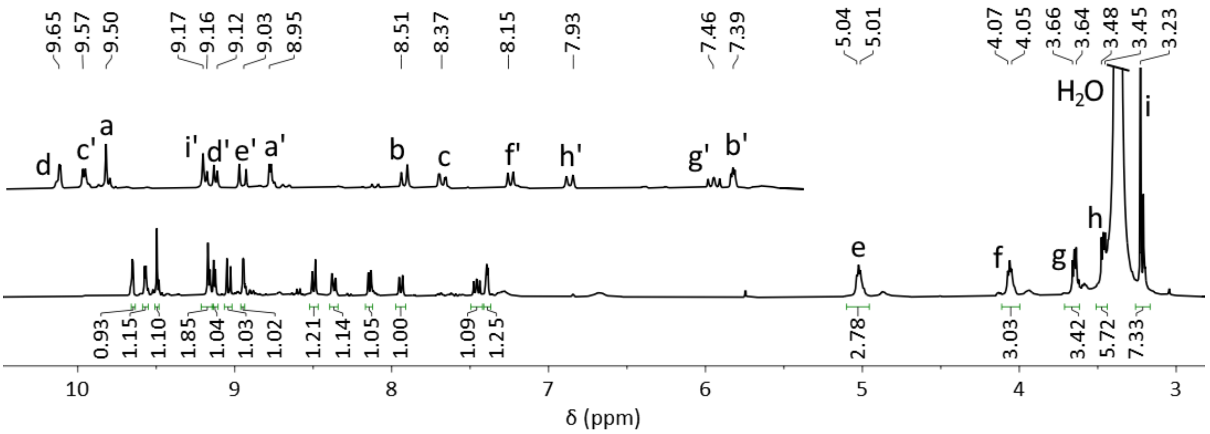

Figure S104 ^{1}H NMR spectrum (400 MHz, $[D_6]$DMSO, 298 K) of **cyc●AD--DA●DA'-N-AD'**.

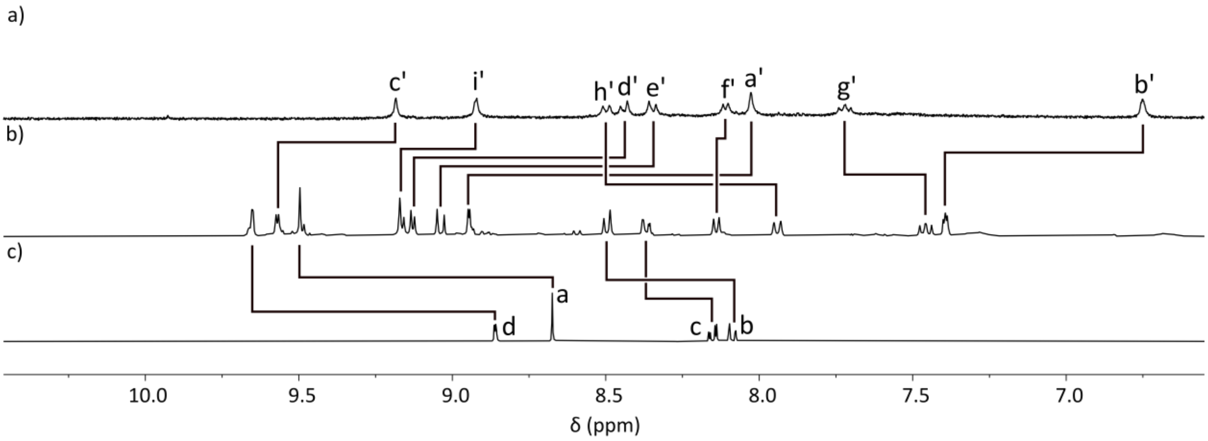

Figure S105 Partial ^{1}H NMR spectra (400 MHz, [D$_6$]DMSO, 298 K) of a) **DA'-N-AD'**, b) **cyc●AD--DA●DA'-N-AD'**, c) **AD--DA**.

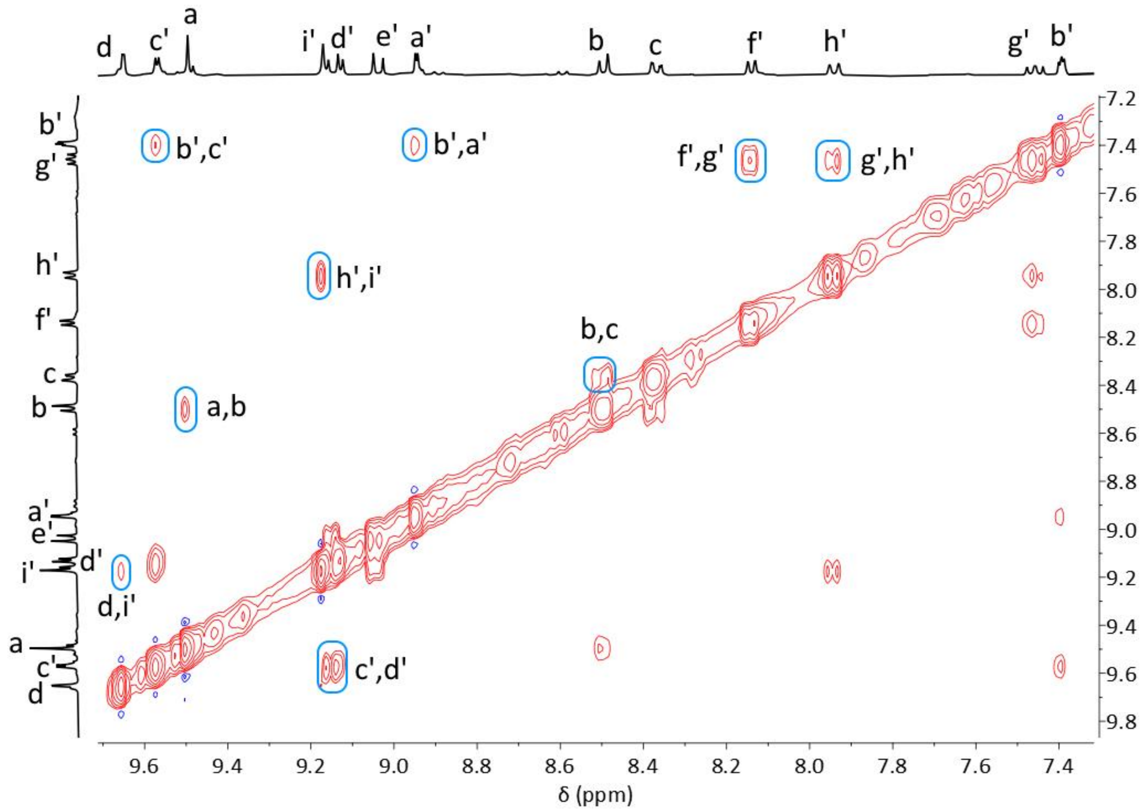

Figure S106 ^{1}H 2D NOESY NMR (400 MHz, [D$_6$]DMSO, 298 K, 200 ms) of **cyc●AD--DA●DA'-N-AD'**.

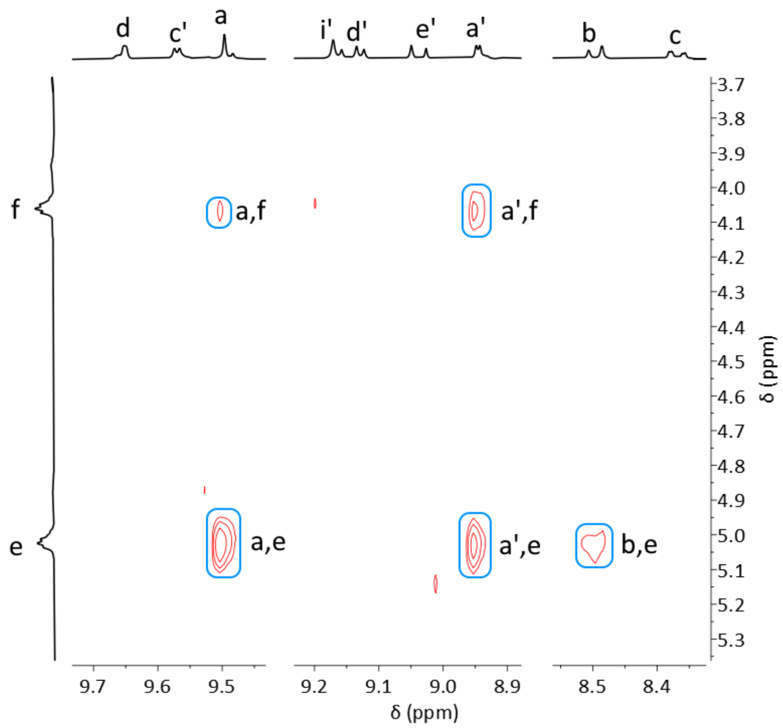

Figure S107 ^{1}H 2D NOESY NMR inset (400 MHz, $[D_6]$DMSO, 298 K, 200 ms) of **cyc●AD--DA●DA'-N-AD'**.

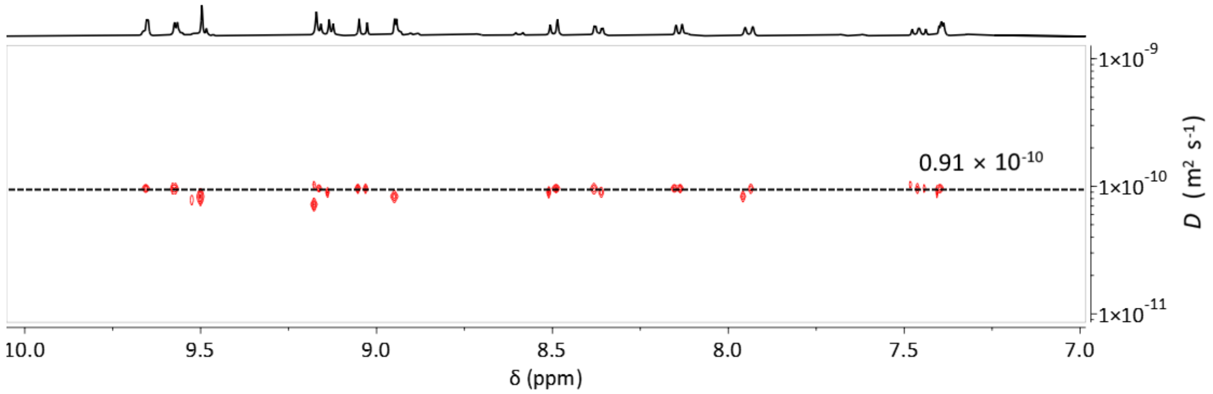

Figure S108 ^{1}H DOSY NMR spectrum (400 MHz, $[D_6]$DMSO, 298 K) of **cyc●AD--DA●DA'-N-AD'**.

1.9.2. *cyc*●AD-DA●DA'-N-AD'●AD-DA

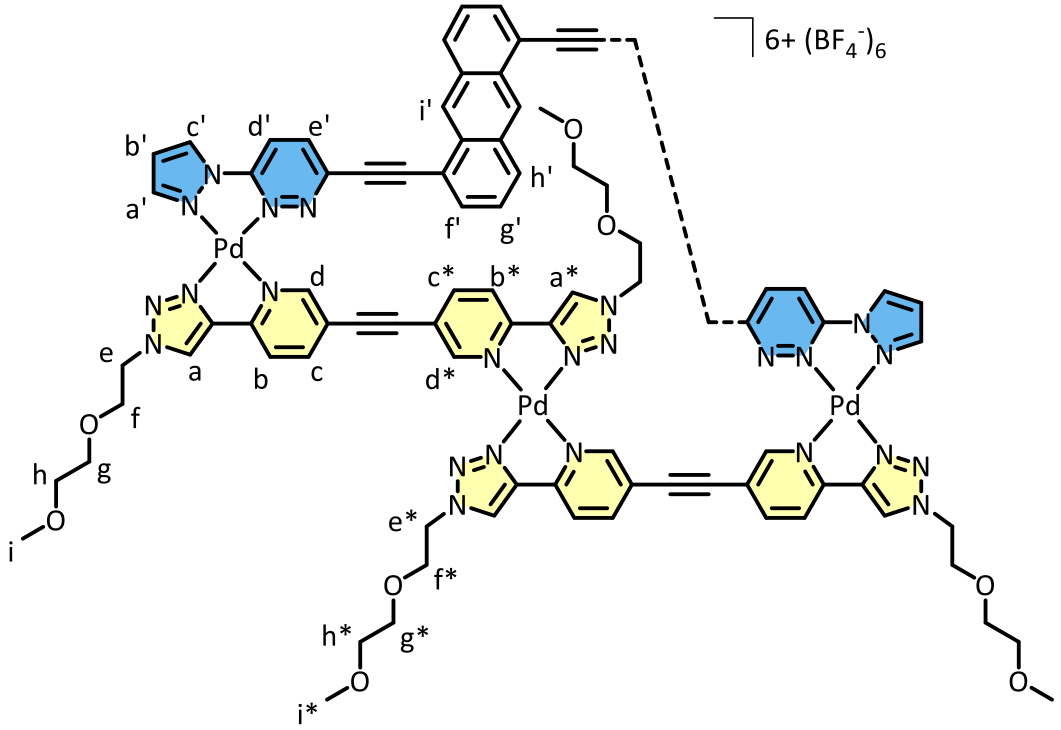

Using stock solutions, **DA'-N-AD'** (0.99 mg, 1.93 µmol), **AD-DA** (2.00 mg, 3.86 µmol) and $[Pd(CH_3CN)_4](BF_4)_2$ (2.57 mg, 5.79 µmol) were combined in $[D_6]$DMSO (550 µL). Equilibration occurred in the time taken to run an NMR spectrum.

^{1}H NMR (400 MHz, $[D_6]$DMSO, 298 K) *integration given per 'half' of the central ligand δ*: 10.18 (1H, br, H_d), 9.61 (1H, d, *J* = 3.2 Hz, $H_{c'}$), 9.54 (1H, s, H_a), 9.50 (1H, s, H_{a*}), 9.24 (1H, d, *J* = 9.2 Hz, $H_{d'}$), 9.03 (1H, d, *J* = 9.2 Hz, $H_{e'}$), 8.97 (1H, d, *J* = 2.2 Hz, $H_{a'}$), 8.87 (1H, d, *J* = 1.2 Hz, H_{d*}), 8.85 (1H, dd, *J* = 8.2 Hz, 1.5 Hz, H_c), 8.72 (1H, dd, *J* = 8.2 Hz, 1.5 Hz, H_{c*}), 8.67 (1H, s, $H_{i'}$), 8.64 (1H, d, *J* = 8.0 Hz, H_b), 8.42 (1H, d, *J* = 8.2 Hz, H_{b*}), 7.86-7.82 (2H, m, $H_{f'}$, $H_{h'}$), 7.45 (1H, t, *J* = 2.7 Hz, $H_{b'}$), 7.13 (1H, t, *J* = 2.7 Hz, $H_{g'}$), 5.06-5.01 (2H, m, H_e), 4.79-4.65 (2H, m, H_{e*}), 4.10-4.07 (2H, m, H_f), 3.94-3.88 (2H, m, H_{f*}), 3.68-3.65 (2H, m, H_f), 3.58-3.50 (4H, m, H_g, H_{g*}), 3.49-3.47 (2H, m, H_h), 3.24 (3H, s, H_i), 3.10 (3H, s, H_{i*}). **Note: H_{h*} is obscured by the H_2O peak.*

D ($\times 10^{-10}$ m^2 s^{-1}, 400 MHz, $[D_6]$DMSO, 298 K) = 0.82.

HR ESI-MS (DMSO/acetonitrile) *m/z* = 378.0719 [**cyc●AD-DA●DA'-N-AD'●AD-DA** + $F^-]^{5+}$ (calc. for $C_{84}H_{78}N_{24}O_8Pd_3F^{5+}$, 378.0682), *m/z* = 510.8406 [**cyc●AD-DA●DA'-N-AD'●AD-DA** + $2BF_4^-]^{4+}$ (calc. for $C_{84}H_{78}N_{24}O_8Pd_3B_2F_8^{4+}$, 510.8418), *m/z* = 710.4587 [**cyc●AD-DA●DA'-N-AD'●AD-DA** + $3BF_4^-]^{3+}$ (calc. for $C_{84}H_{78}N_{24}O_8Pd_3B_3F_{12}^{3+}$, 710.4598), *m/z* = 1109.1791 [**cyc●AD-DA●DA'-N-AD'●AD-DA** + $4BF_4^-]^{2+}$ (calc. for $C_{84}H_{78}N_{24}O_8Pd_3B_4F_{16}^{2+}$, 1109.1879).

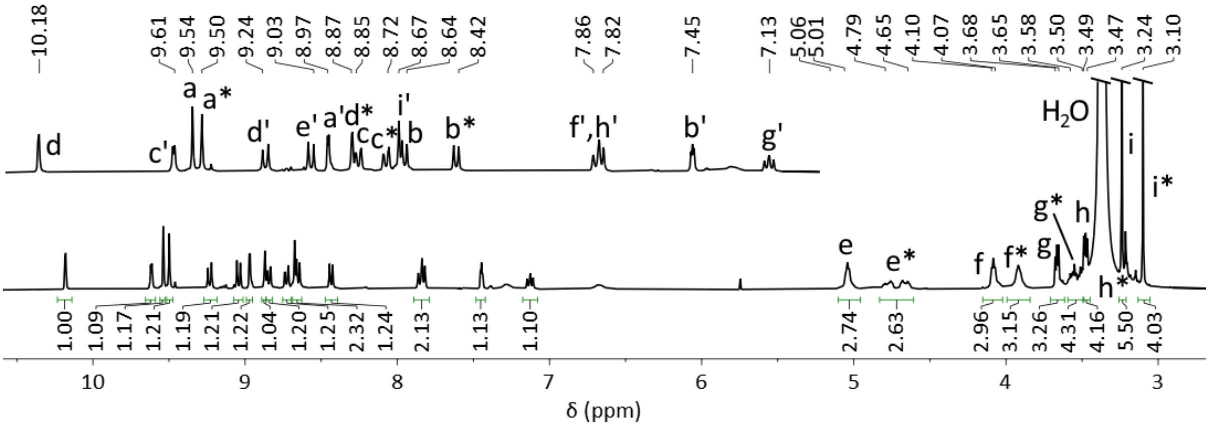

Figure S109 ^{1}H NMR spectrum (400 MHz, [D$_6$]DMSO, 298 K) of **AD-DA●DA'-N-AD'●AD-DA**.

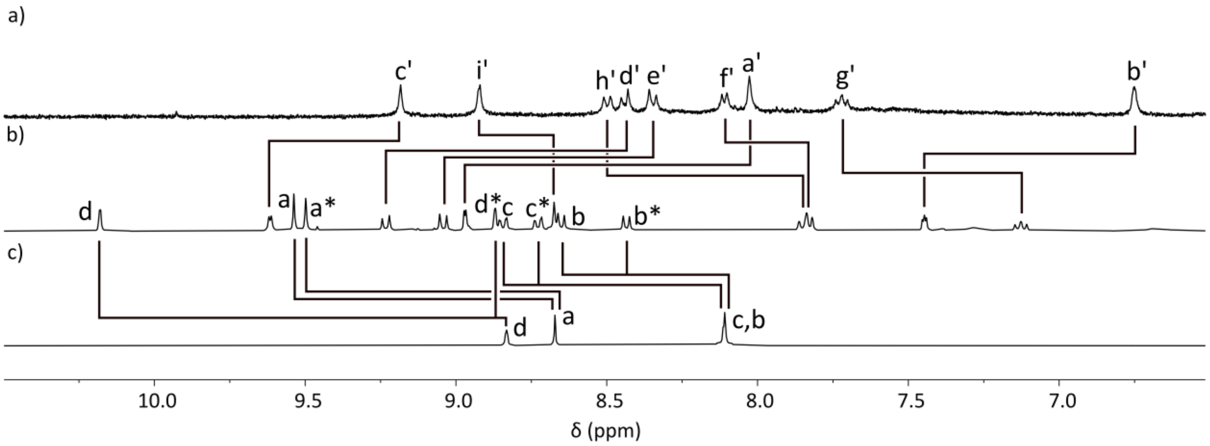

Figure S110 Partial ^{1}H NMR spectra (400 MHz, [D$_6$]DMSO, 298 K) of a) **DA'-N-AD'**, b) **AD-DA●DA'-N-AD'●AD-DA**, c) **AD-DA**.

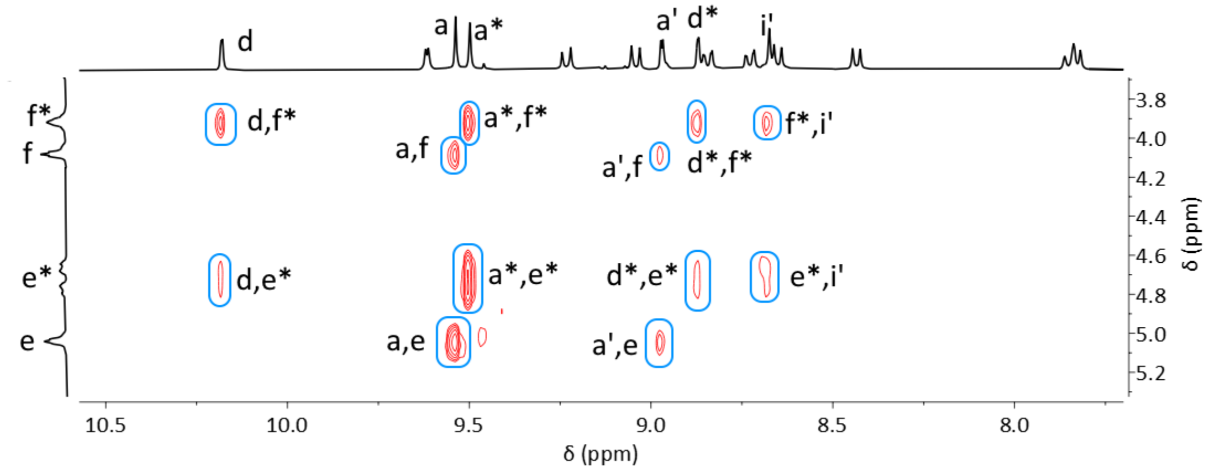

Figure S111 ^{1}H 2D NOESY NMR inset (400 MHz, [D$_6$]DMSO, 298 K, 200 ms) of **AD-DA●DA'-N-AD'●AD-DA**.

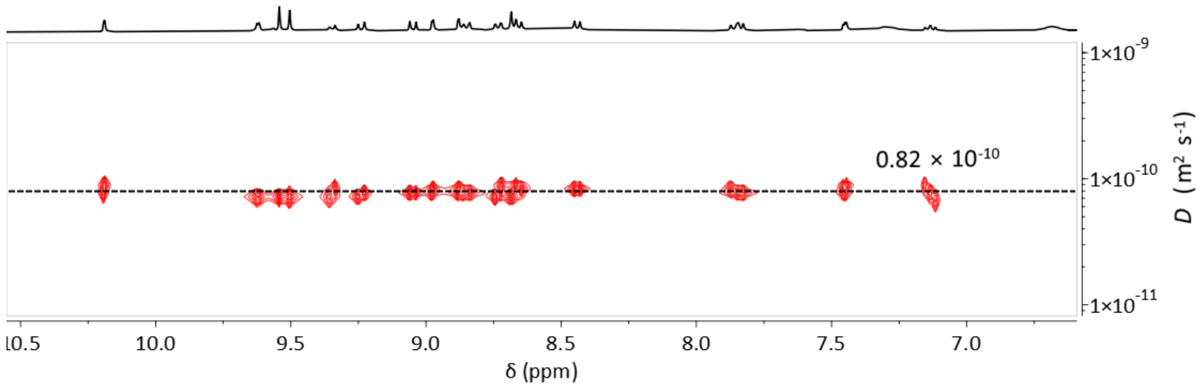

Figure S112 ^{1}H DOSY NMR spectrum (400 MHz, $[D_6]$DMSO, 298 K) of **AD-DA●DA'-N-AD'●AD-DA**.

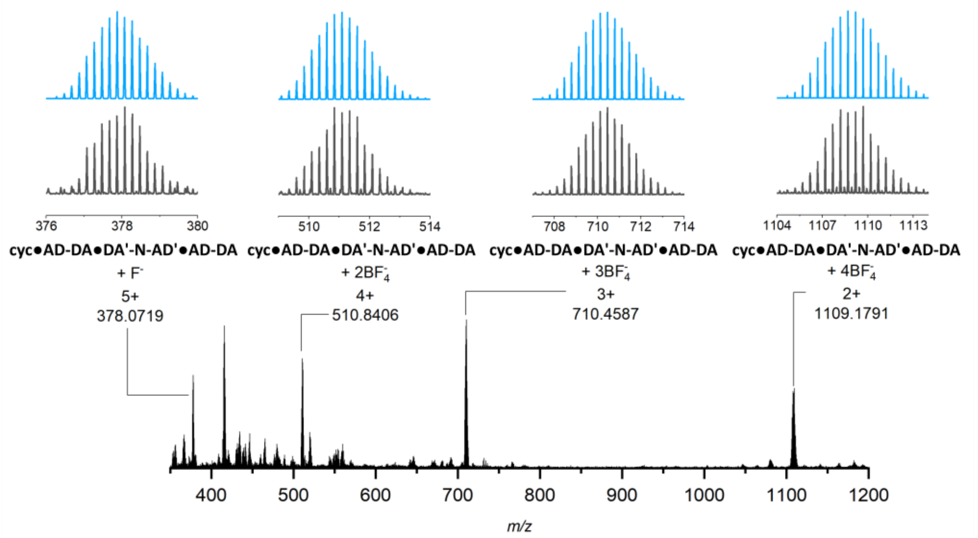

Figure S113 HR-ESI MS (DMSO/acetonitrile) of **AD-DA●DA'-N-AD'●AD-DA**.

1.9.3. *cyc*●DD-DA●AA-N-AA●AD-DD

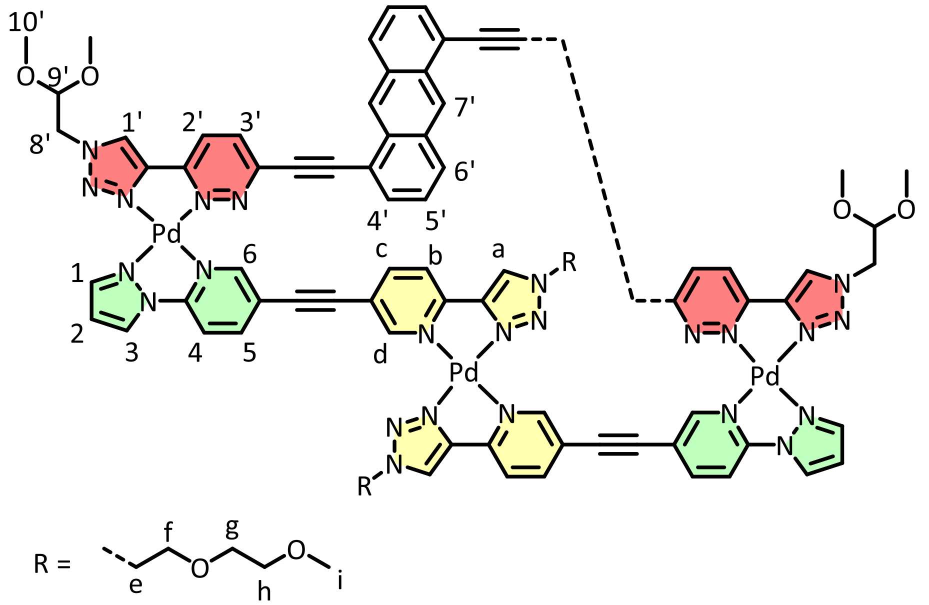

Using stock solutions, **AA-N-AA** (1.56 mg, 2.25 µmol), **AD-DD** (1.87 mg, 4.50 µmol) and $[Pd(CH_3CN)_4](BF_4)_2$ (3.00 mg, 6.75 µmol) were combined in $[D_6]$DMSO (550 µL). Equilibration occurred in the time taken to run an NMR spectrum.

^{1}H NMR (400 MHz, $[D_6]$DMSO, 298 K) *integration given per 'half' of the central ligand δ*: 10.10 (1H, s, H_6), 9.61 (1H, s, $H_{1'}$), 9.57 (1H, d, J = 3.2 Hz, H_3), 9.50 (1H, s, H_a), 9.24 (1H, d, J = 8.7 Hz, $H_{2'}$), 8.99-8.93 (2H, m, $H_{3'}$, H_4), 8.87-8.85 (2H, m, H_d, H_1), 8.73-8.65 (3H, m, $H_{7'}$, H_c, H_5), 8.44 (1H, d, J = 8.1 Hz, H_b), 7.86 (2H, m, $H_{4'}$, $H_{6'}$), 7.37 (1H, br, H_2), 7.12 (1H, t, J = 6.8 Hz, $H_{5'}$), 5.16 (2H, br, $H_{8'}$), 5.11-5.00 (1H, m, $H_{9'}$), 4.80-4.62 (1H, m, H_e), 3.94-3.88 (2H, m, H_f), 3.50-3.48 (6H, m, H_{10}), 3.10 (3H, s, H_i). **Note: H_g and H_h are obscured by the H_2O peak.*

D (× 10^{-10} m^2 s^{-1}, 400 MHz, $[D_6]$DMSO, 298 K) = 0.66.

HR ESI-MS (DMSO/acetonitrile) m/z = 504.0829 [**cyc●DD-DA●AA-N-AA●AD-DD** + $2BF_4^-]^{4+}$ (calc. for $C_{82}H_{74}N_{24}O_8Pd_3B_2F_8^{4+}$, 504.0836), m/z = 701.1203 [**cyc●DD-DA●AA-N-AA●AD-DD** + $3BF_4^-]^{3+}$ (calc. for $C_{82}H_{74}N_{24}O_8Pd_3B_3F_{12}^{3+}$, 701.1127), m/z = 1094.6696 [**cyc●DD-DA●AA-N-AA●AD-DD** + $4BF_4^-]^{2+}$ (calc. for $C_{82}H_{74}N_{24}O_8Pd_3B_4F_{16}^{2+}$, 1094.6709).

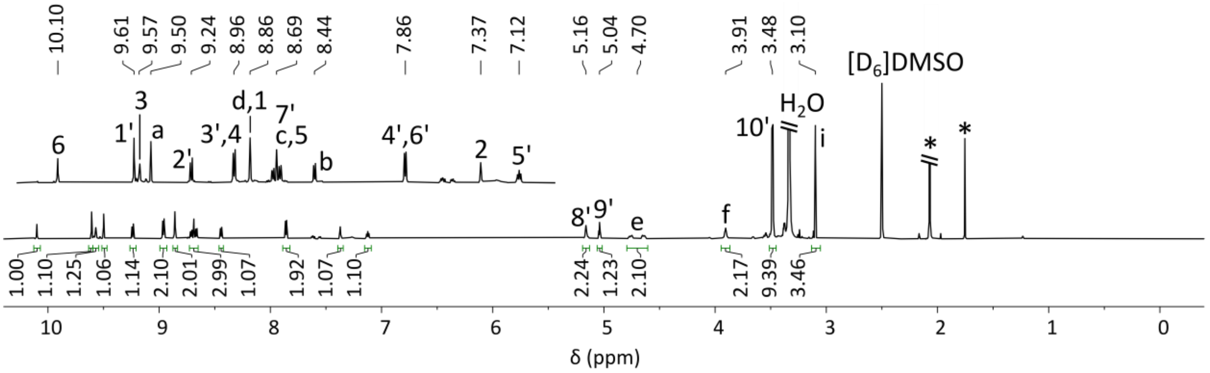

Figure S114 ^{1}H NMR spectrum (400 MHz, $[D_6]$DMSO, 298 K) of **cyc●DD-DA●AA-N-AA●AD-DD**. Impurities from the Pd(II) source are indicated by *.

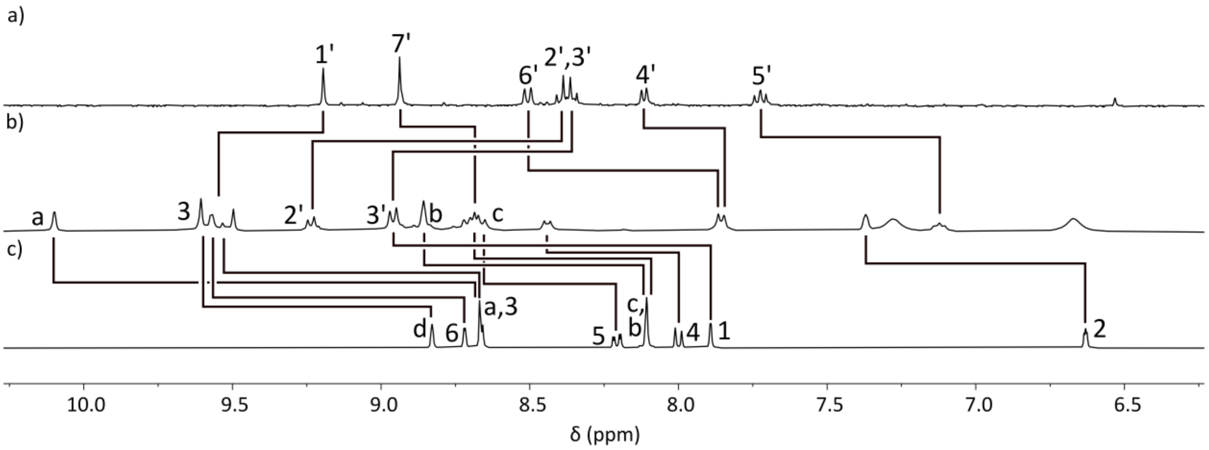

Figure S115 Partial ^{1}H NMR spectra (400 MHz, [D_6]DMSO, 298 K) of a) **AA-N-AA**, b) **cyc●DD-DA●AA-N-AA●AD-DD**, c) **AD-DD**.

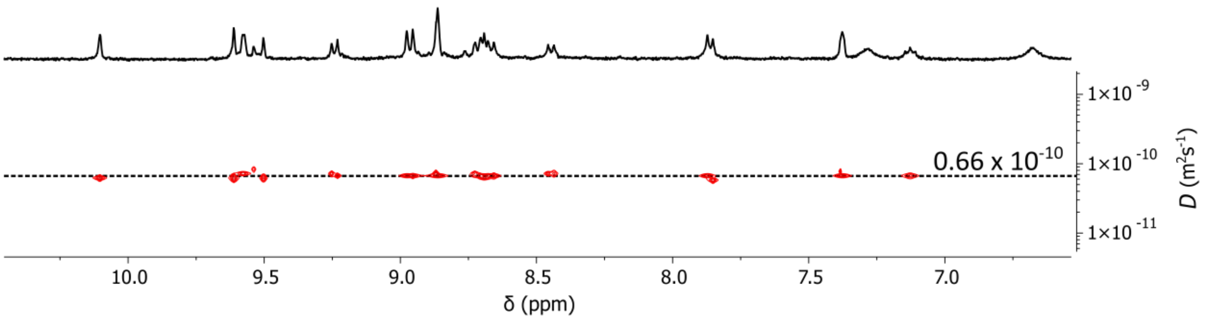

Figure S116 ^{1}H DOSY NMR spectrum (400 MHz, [D_6]DMSO, 298 K) of **cyc●DD-DA●AA-N-AA●AD-DD**.

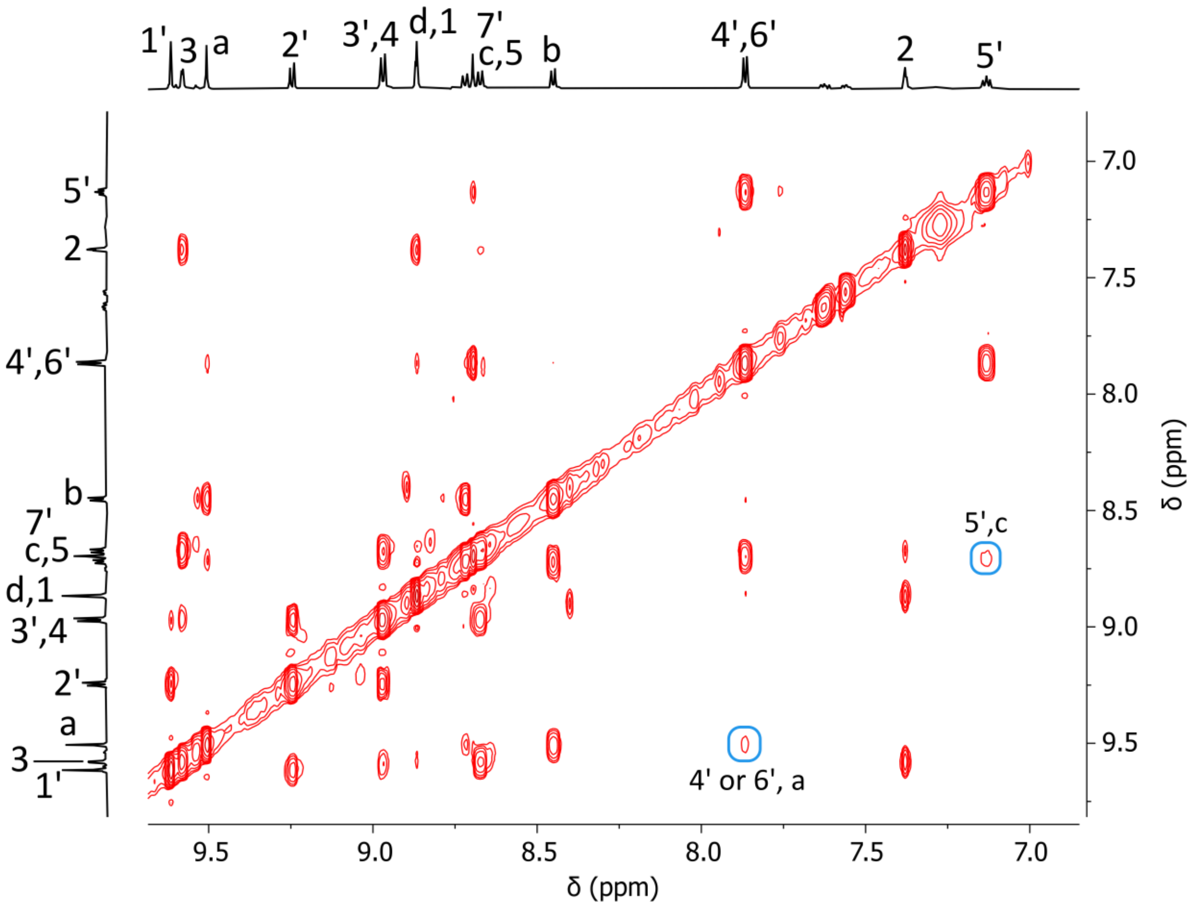

Figure S117 ^{1}H 2D NOESY NMR (400 MHz, [D$_6$]DMSO, 298 K, 200 ms) of **cyc●DD-DA●AA-N-AA●AD-DD**.

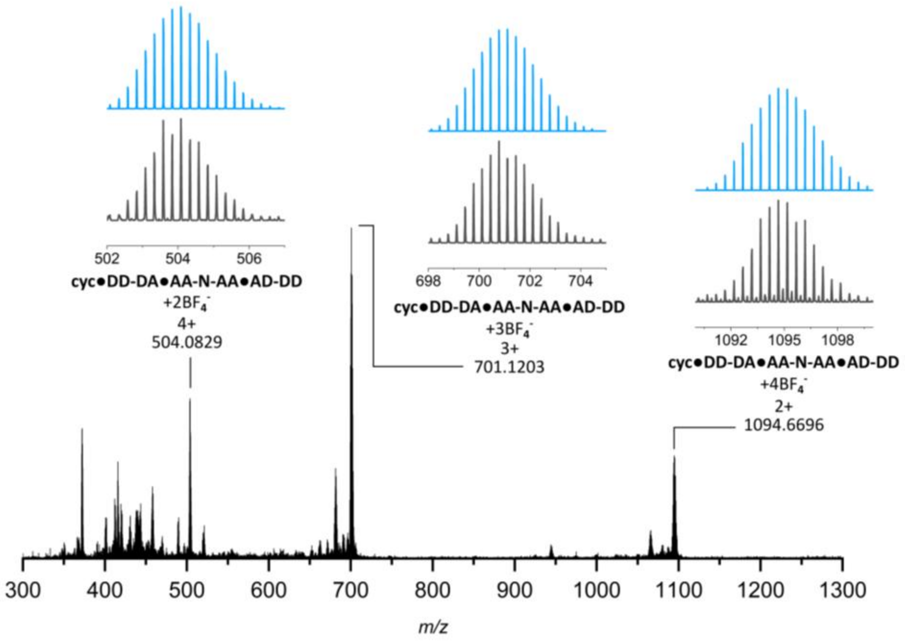

Figure S118 HR-ESI MS (DMSO/acetonitrile) of **cyc●DD-DA●AA-N-AA●AD-DD**.

1.9.4. *cyc*●AD-DA●AA-N-AA●AD-DA

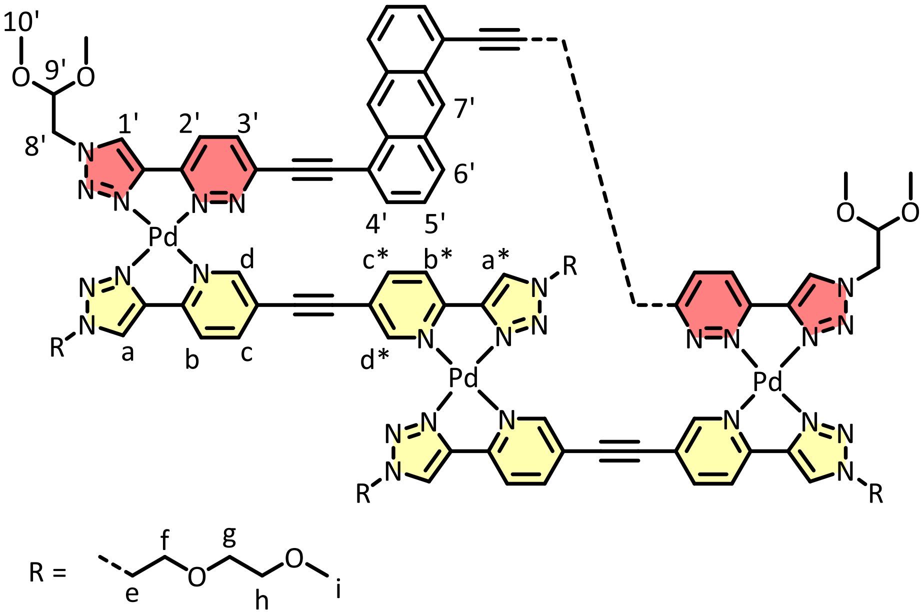

Using stock solutions, **AA-N-AA** (1.30 mg, 1.88 µmol), **AD-DA** (1.95 mg, 3.76 µmol) and $[Pd(CH_3CN)_4](BF_4)_2$ (2.50 mg, 5.63 µmol) were combined in [D$_6$]DMSO (550 µL). Equilibration occurred in the time taken to run an NMR spectrum.

^{1}H NMR (400 MHz, [D$_6$]DMSO, 298 K) *integration given per 'half' of the central ligand δ*: 10.25 (1H, s, H_d), 9.59 (1H, s, $H_{1'}$), 9.54 (1H, s, H_a), 9.50 (1H, s, H_{a*}), 9.23 (1H, d, *J* = 8.7 Hz, $H_{2'}$), 8.97 (1H, d, *J* = 8.7 Hz, $H_{3'}$), 8.87-8.83 (2H, m, H_{d*}, H_c), 8.73 (1H, dd, *J* = 8.3 Hz, 1.3 Hz, H_{c*}), 8.70 (1H, s, $H_{7'}$), 8.64 (1H, d, *J* = 8.0 Hz, H_b), 8.45 (1H, d, *J* = 8.1 Hz, H_{b*}), 7.88-7.82 (2H, m, $H_{4'}$, $H_{6'}$), 7.13 (1H, t, *J* = 7.7 Hz, $H_{5'}$), 5.10 (2H, br, $H_{8'}$), 5.01-4.94 (3H, m, $H_{9'}$, H_e), 4.80-4.62 (2H, m, H_{e*}), 4.08-4.02 (2H, m, H_f), 3.92-3.88 (2H, m, H_{f*}), 3.68-3.64 (2H, m, H_g), 3.56-3.54 (2H, m, H_{g*}), 3.49-3.47 (6H, m, H_{10}), 3.24 (3H, s, H_i), 3.10 (3H, s, H_{i*}).

D ($\times 10^{-10}$ m^2 s^{-1}, 400 MHz, [D$_6$]DMSO, 298 K) = 0.69.

HR ESI-MS (DMSO/acetonitrile) *m/z* = 555.6232 [**cyc●AD-DA●AA-N-AA●AD-DA** + $2BF_4^-]^{4+}$ (calc. for $C_{90}H_{92}N_{26}O_{12}Pd_3B_2F_8^{4+}$, 555.6135), *m/z* = 769.8300 [**cyc●AD-DA●AA-N-AA●AD-DA** + $3BF_4^-]^{3+}$ (calc. for $C_{90}H_{92}N_{26}O_{12}Pd_3B_3F_{12}^{3+}$, 769.8217), *m/z* = 1198.2493 [**cyc●AD-DA●AA-N-AA●AD-DA** + $4BF_4^-]^{2+}$ (calc. for $C_{90}H_{92}N_{26}O_{12}Pd_3B_4F_{16}^{2+}$, 1198.2344).

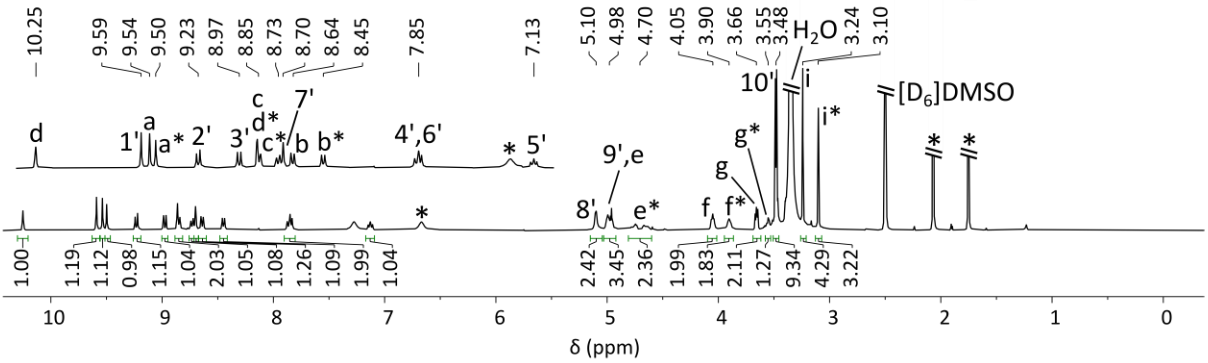

Figure S119 ^{1}H NMR spectrum (400 MHz, [D$_6$]DMSO, 298 K) of **cyc●AD-DA●AA-NN-AA●AD-DA**. Impurities from the Pd(II) source are indicated by *.

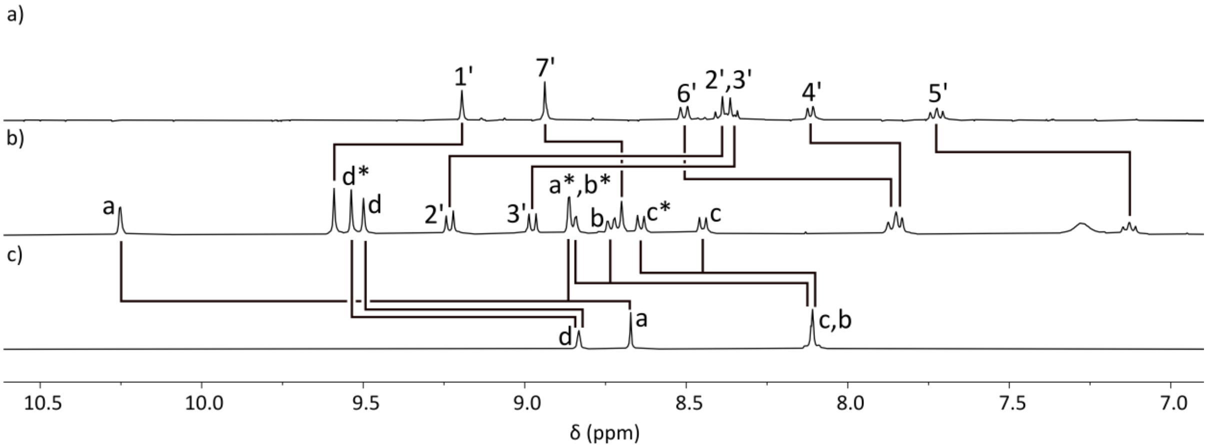

Figure S120 Partial ^{1}H NMR spectra (400 MHz, [D$_6$]DMSO, 298 K) of a) **AA-N-AA**, b) **cyc●AD-DA●AA-N-AA●AD-DA**, c) **AD-DA**.

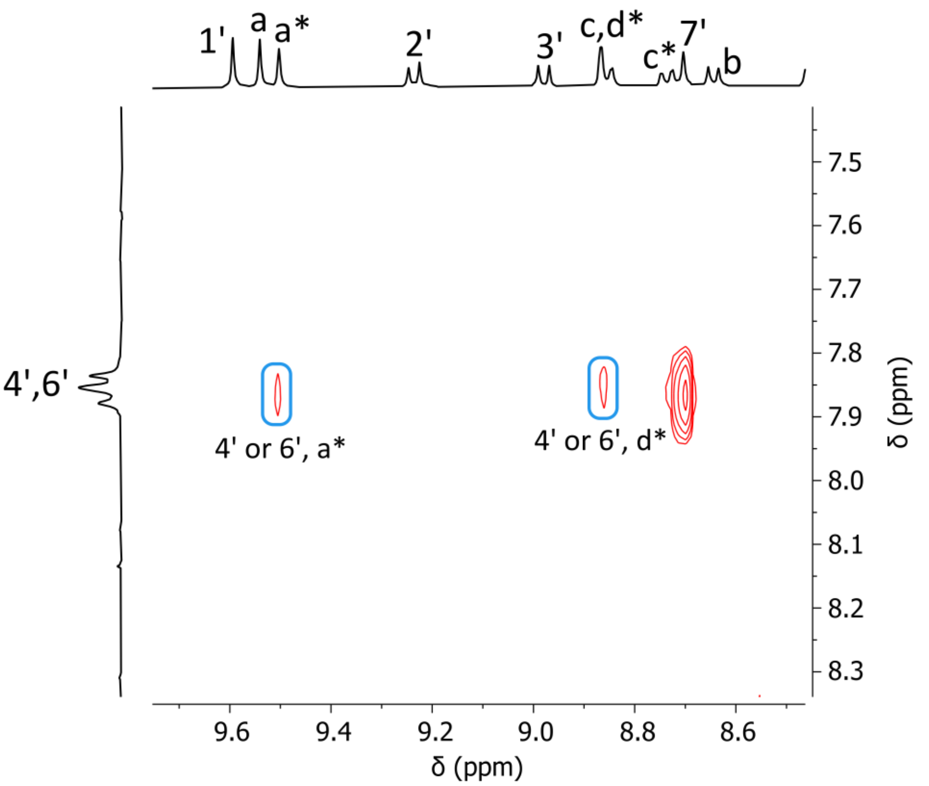

Figure S121 ^{1}H 2D NOESY NMR (400 MHz, [D$_6$]DMSO, 298 K, 200 ms) of **cyc●AD-DA●AA-N-AA●AD-DA**.

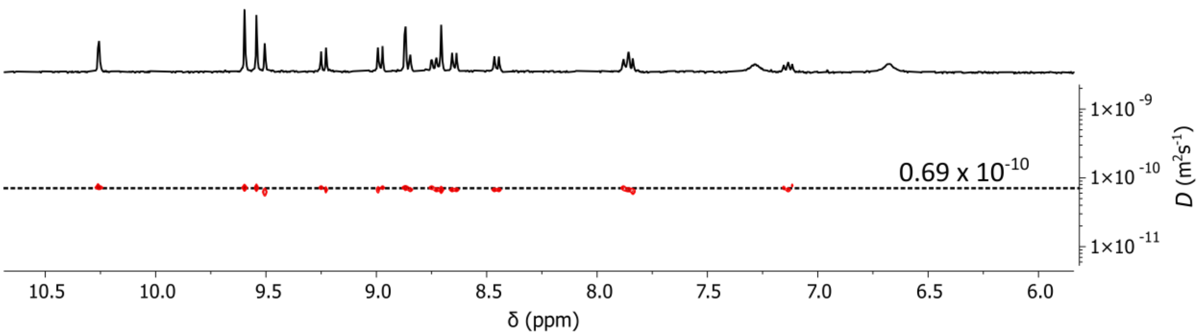

Figure S122 ^{1}H DOSY NMR spectrum (400 MHz, [D$_6$]DMSO, 298 K) of **cyc●AD-DA●AA-NN-AA●AD-DA**.

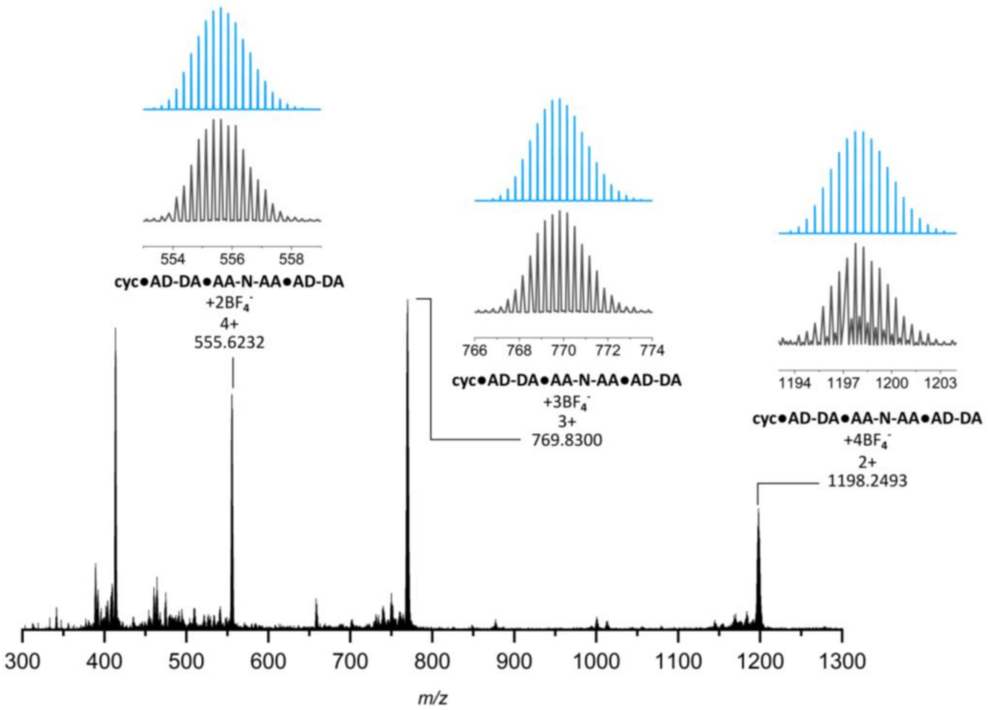

Figure S123 HR-ESI MS (DMSO/acetonitrile) of **cyc●AD-DA●AA-N-AA●AD-DA**.

1.9.5. *cyc*●AD-DA●DA'-NN-AD'

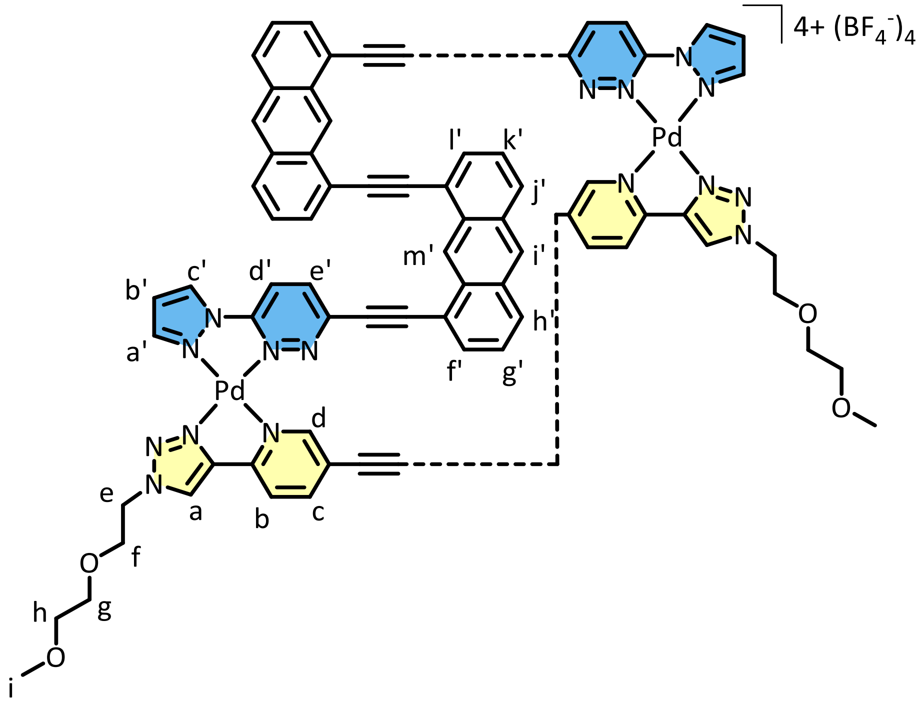

Using stock solutions, **DA'-NN-AD'** (2.05 mg, 2.87 µmol), **AD-DA** (1.24 mg, 2.87 µmol) and $[Pd(CH_3CN)_4](BF_4)_2$ (2.55 mg, 5.74 µmol) were combined in $[D_6]$DMSO (550 µL). Equilibration occurred in the time taken to run an NMR spectrum.

^{1}H NMR (400 MHz, $[D_6]$DMSO, 298 K) *integration given per 'half' of the central ligand δ*: 9.63 (1H, d, J = 3.2 Hz, $H_{c'}$), 9.51 (1H, s, H_a), 9.47 (1H, s, $H_{m'}$), 9.10-9.05 (3H, m, $H_{a'}$, $H_{d'}$, $H_{e'}$), 9.02 (1H, d, J = 1.5 Hz, H_d), 8.29 (1H, d, J = 8.8 Hz, $H_{h'}$), 8.26 (1H, d, J = 7.0 Hz, $H_{f'}$), 8.23 (1H, s, $H_{i'}$), 7.96 (1H, dd, J = 8.4 Hz, 6.8 Hz, $H_{k'}$), 7.91-7.82 (3H, m, $H_{g'}$, $H_{j'}$, $H_{l'}$), 7.51 (1H, dd, J = 8.1 Hz, 1.6 Hz, H_c), 7.47 (1H, t, J = 2.7 Hz, $H_{b'}$), 7.09 (1H, d, J = 8.1 Hz, H_b), 5.16–5.12 (2H, m, H_e), 4.22–4.19 (2H, m, H_f), 3.79 – 3.76 (2H, m, H_g), 3.57 – 3.55 (2H, m, H_h), 3.23 (3H, s, H_i).

D (× 10^{-10} m^2 s^{-1}, 400 MHz, $[D_6]$DMSO, 298 K) = 0.91.

HR ESI-MS (DMSO/acetonitrile) m/z = 361.5722 [**cyc●AD-DA●DA'-NN-AD'**]$^{4+}$ (calc. for $C_{74}H_{56}N_{16}O_4Pd_2^{4+}$, 361.5696), m/z = 447.7334 [**cyc●AD-DA●DA'-NN-AD'** – $CH_3OCH_2CH_2OCH_2CH_2^+$]$^{3+}$ (calc. for $C_{69}H_{45}N_{16}O_2Pd_2^{3+}$, 447.7336), m/z = 481.7575 [**cyc●AD-DA●DA'-NN-AD'** – H^+]$^{3+}$ (calc. for $C_{74}H_{55}N_{16}O_4Pd_2^{3+}$, 481.7542), m/z = 492.4354 [**cyc●AD-DA●DA'-NN-AD'** + CH_3O^-]$^{3+}$ (calc. for $C_{74}H_{56}N_{16}O_4Pd_2CH_3O^{3+}$, 481.7542).

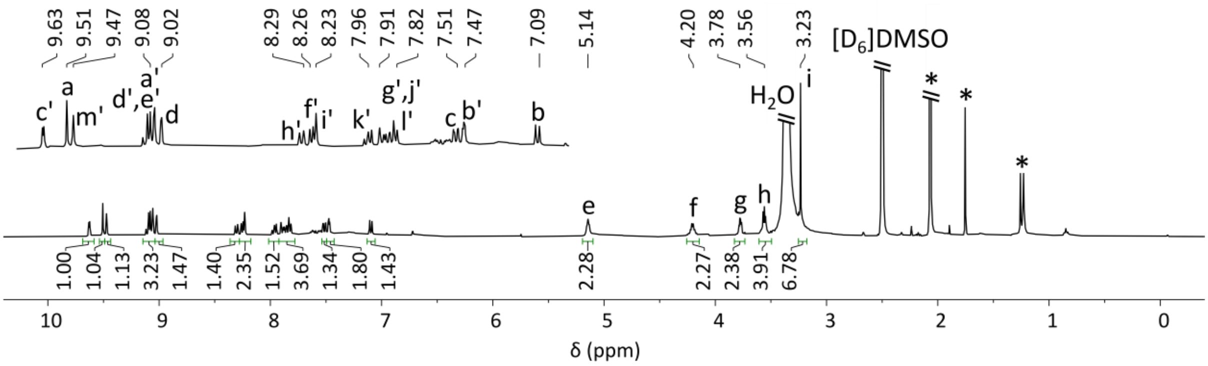

Figure S124 ^{1}H NMR spectrum (400 MHz, [D$_6$]DMSO, 298 K) of **AD-DA●DA'-NN-AD'**. Impurities from the Pd(II) source are indicated by *.

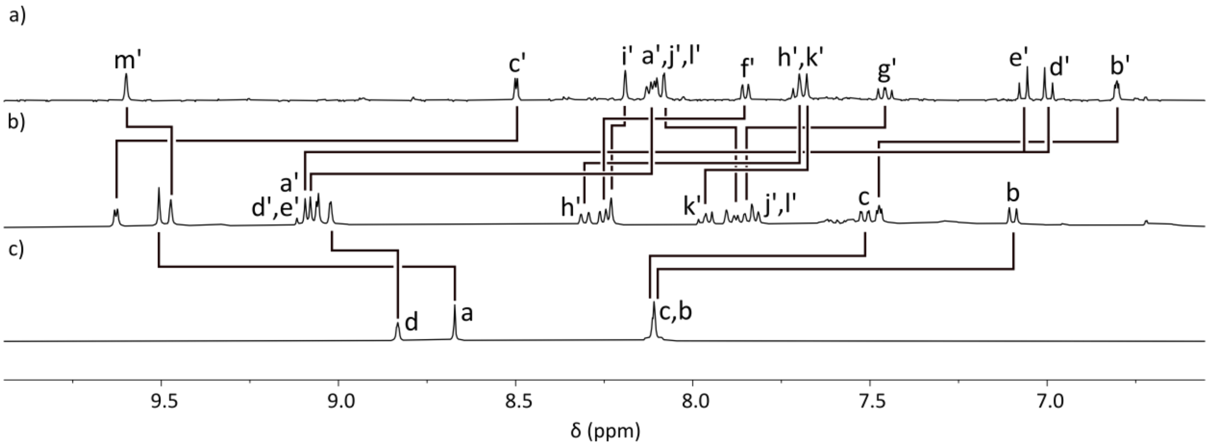

Figure S125 Partial ^{1}H NMR spectra (400 MHz, [D$_6$]DMSO, 298 K) of a) **DA'-NN-AD'**, b) **AD-DA●DA'-NN-AD'**, c) **AD-DA**.

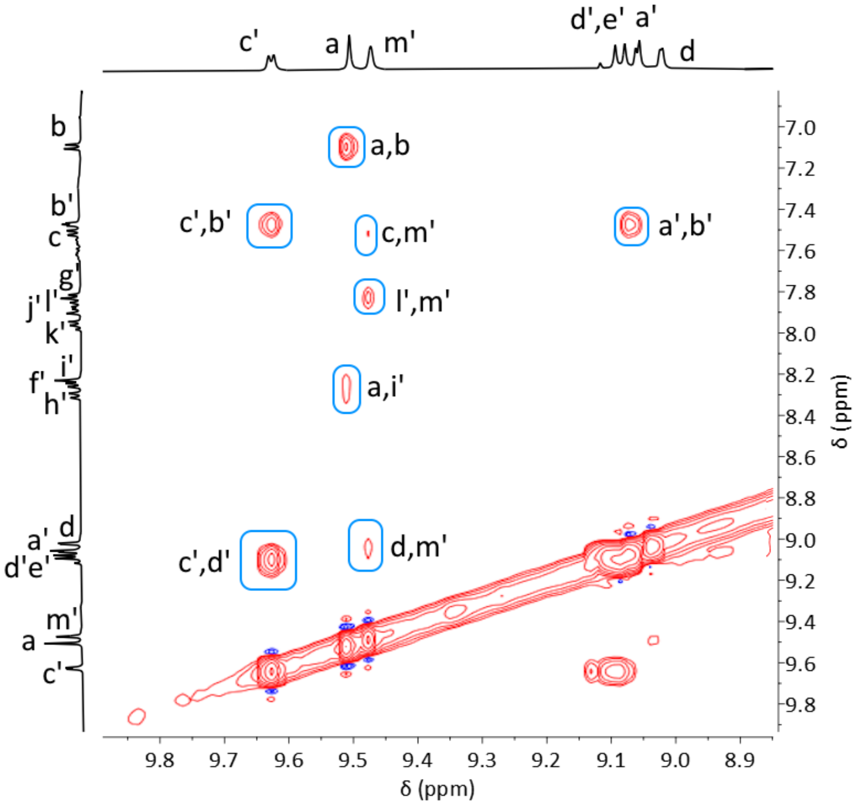

Figure S126 ^{1}H 2D NOESY NMR inset (400 MHz, [D$_6$]DMSO, 298 K, 200 ms) of **AD-DA●DA'-NN-AD'**.

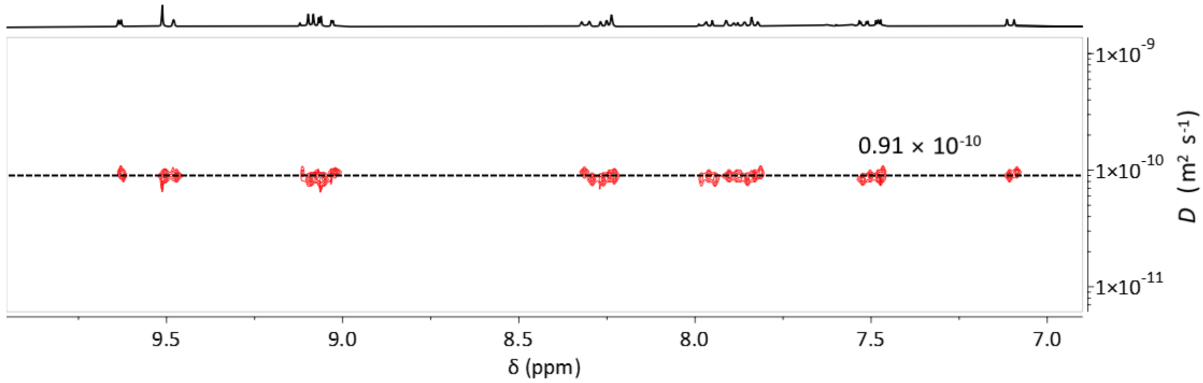

Figure S127 ^{1}H DOSY NMR spectrum (400 MHz, [D$_6$]DMSO, 298 K) of **AD-DA●DA'-NN-AD'**.

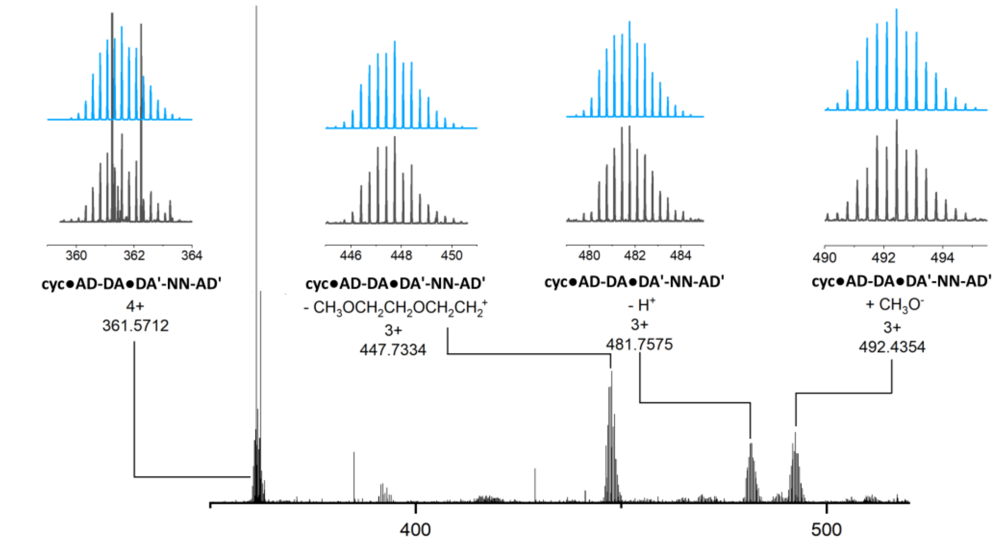

Figure S128 HR-ESI MS (DMSO/acetonitrile) of **AD-DA●DA'-NN-AD'**.

1.9.6. *cyc*●AD--DA●DA'-NN-AD'

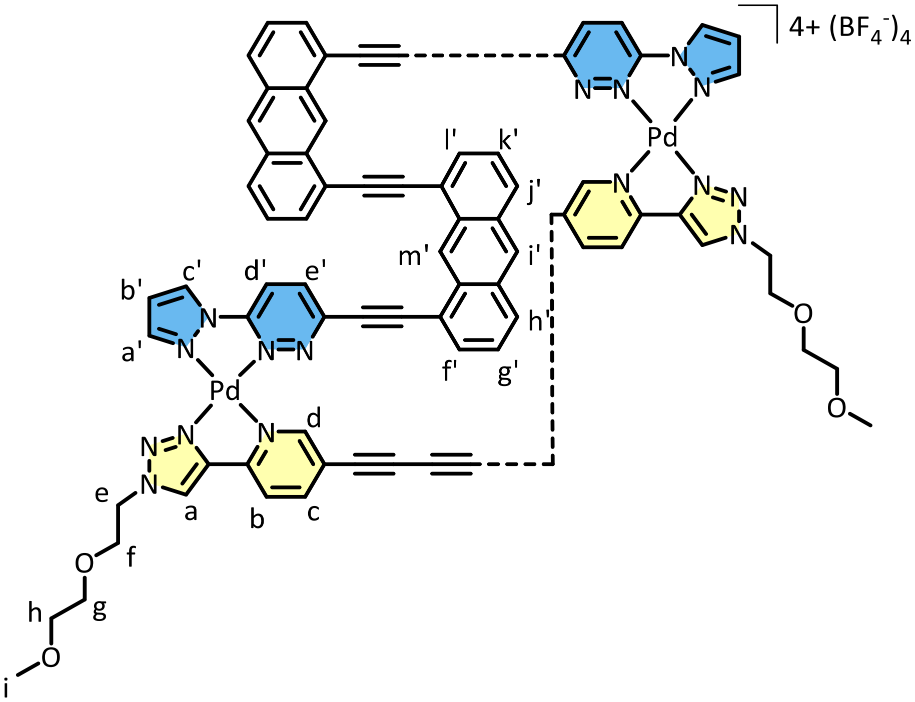

Using stock solutions, **DA'-NN-AD'** (1.57 mg, 2.20 µmol), **AD--DA** (1.00 mg, 2.20 µmol) and $[Pd(CH_3CN)_4](BF_4)_2$ (1.95 mg, 4.40 µmol) were combined in $[D_6]$DMSO (550 µL). Equilibration occurred in the time taken to run an NMR spectrum.

^{1}H NMR (400 MHz, $[D_6]$DMSO, 298 K) *integration given per 'half' of the central ligand δ*: 9.84 (1H, s, $H_{m'}$), 9.58 (1H, s, H_a), 9.52 (1H, d, *J* = 3.2 Hz, $H_{c'}$), 9.09 (1H, d, *J* = 2.3 Hz, $H_{a'}$), 8.86 (1H, dd, *J* = 8.5 Hz, 1.6 Hz, H_c), 8.82 (1H, d, *J* = 1.5 Hz, H_d), 8.68-8.66 (2H, m, H_b, $H_{i'}$), 8.32 (1H, d, *J* = 8.8 Hz, $H_{h'}$), 8.16-8.13 (2H, m, $H_{e'}$, $H_{l'}$), 7.94 (1H, d, *J* = 8.8 Hz, $H_{d'}$), 7.91 (1H, d, *J* = 7.4 Hz, $H_{f'}$), 7.78 (1H, d, *J* = 8.8 Hz, $H_{j'}$), 7.74 (1H, dd, *J* = 8.8 Hz, 2.0 Hz, $H_{k'}$), 7.53 (1H, t, *J* = 2.7 Hz, $H_{b'}$), 7.03 (1H, t, *J* = 7.7 Hz, $H_{g'}$), 5.12–5.08 (2H, m, H_e), 4.13–4.09 (2H, m, H_f), 3.74 – 3.70 (2H, m, H_g), 3.53 – 3.49 (2H, m, H_h), 3.28 (3H, s, H_i).

D ($\times 10^{-10}$ m^2 s^{-1}, 400 MHz, $[D_6]$DMSO, 298 K) = 0.85.

HR ESI-MS (DMSO/acetonitrile) *m/z* = 367.5667 **[cyc●AD--DA●DA'-NN-AD']**$^{4+}$ (calc. for $C_{76}H_{56}N_{16}O_4Pd_2^{4+}$, 367.5696), *m/z* = 455.7333 **[cyc●AD--DA●DA'-NN-AD'** – $CH_3OCH_2CH_2OCH_2CH_2^+]^{3+}$ (calc. for $C_{71}H_{45}N_{16}O_2Pd_2^{3+}$, 455.7336), *m/z* = 489.7549 **[cyc●AD--DA●DA'-NN-AD'** – $H^+]^{3+}$ (calc. for $C_{76}H_{55}N_{16}O_4Pd_2^{3+}$, 489.7542), *m/z* = 500.4327 **[cyc●AD--DA●DA'-NN-AD'** + $CH_3O^-]^{3+}$ (calc. for $C_{76}H_{56}N_{16}O_4Pd_2CH_3O^{3+}$, 500.4297), *m/z* = 519.0948 **[cyc●AD--DA●DA'-NN-AD'** + $BF_4^-]^{3+}$ (calc. for $C_{76}H_{56}N_{16}O_4Pd_2BF_4^{3+}$, 519.0940).

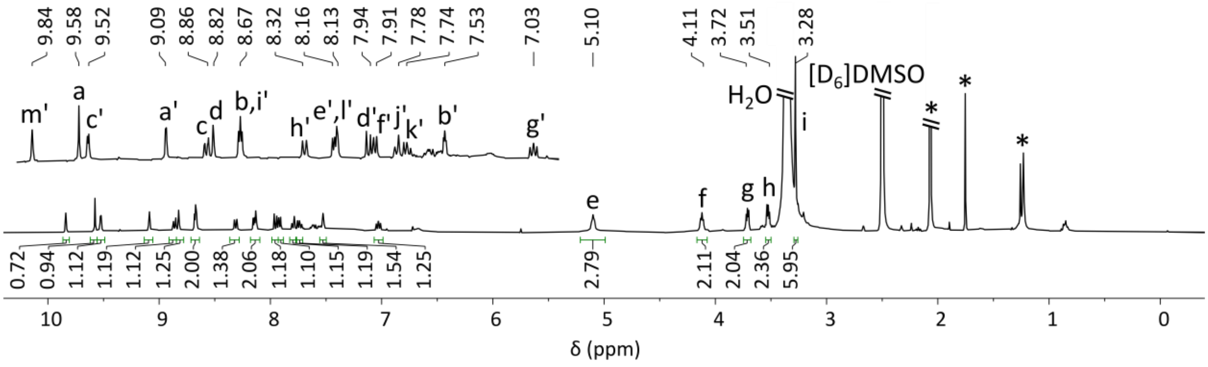

Figure S129 ^{1}H NMR spectrum (400 MHz, [D$_6$]DMSO, 298 K) of **AD--DA●DA'-NN-AD'**. Impurities from the Pd(II) source are indicated by *.

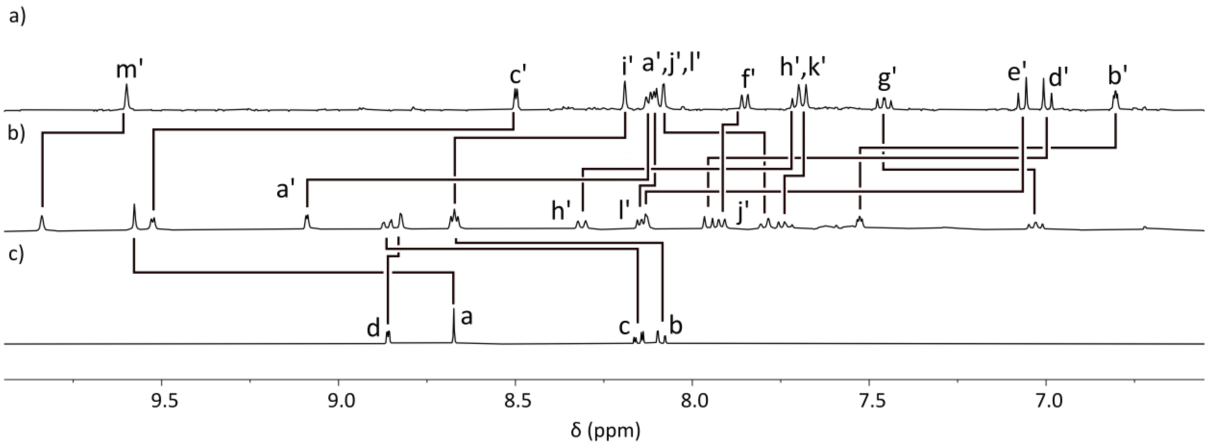

Figure S130 Partial ^{1}H NMR spectra (400 MHz, [D$_6$]DMSO, 298 K) of a) **DA'-NN-AD'**, b) **AD--DA●DA'-NN-AD'**, c) **AD--DA**.

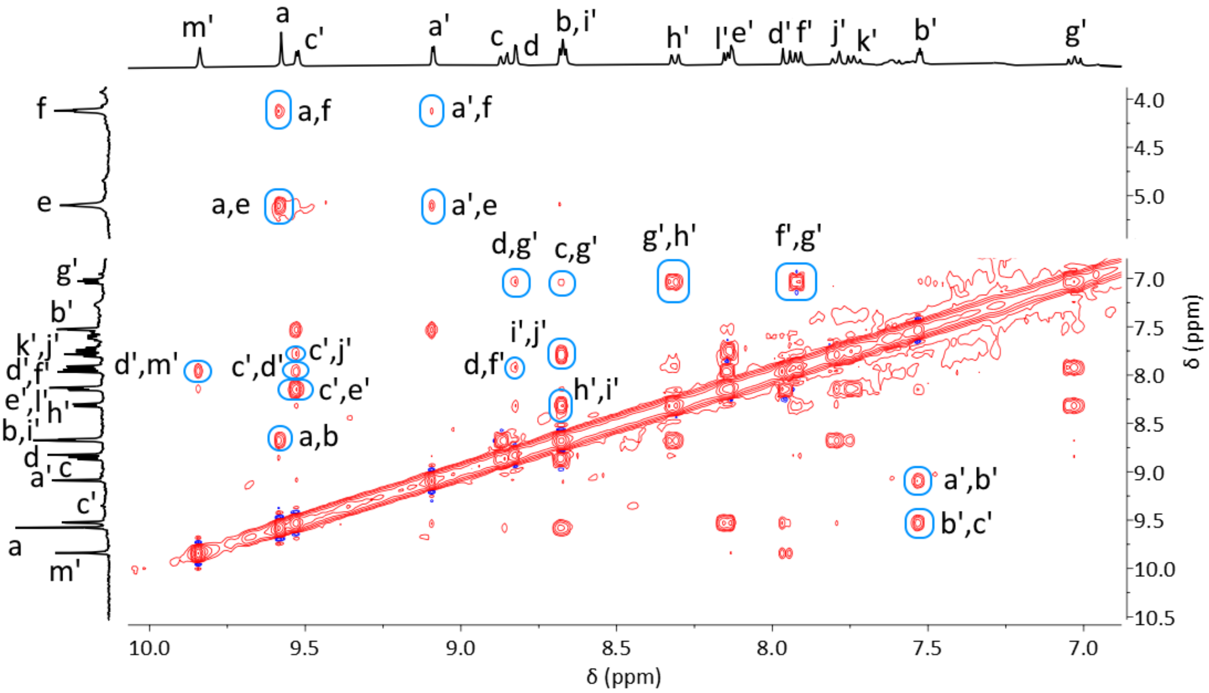

Figure S131 ^{1}H 2D NOESY NMR (400 MHz, [D$_6$]DMSO, 298 K, 200 ms) of **AD--DA●DA'-NN-AD'**.

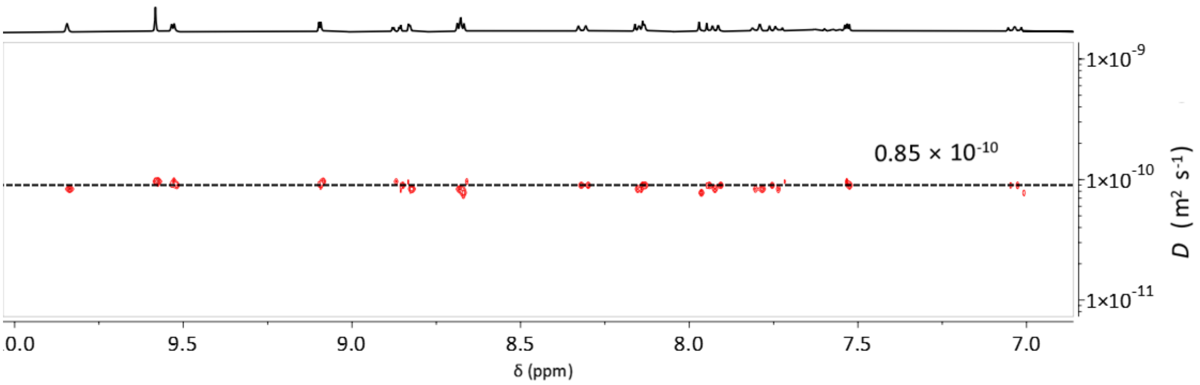

Figure S132 ^{1}H DOSY NMR spectrum (400 MHz, [D$_6$]DMSO, 298 K) of **AD--DA●DA'-NN-AD'**.

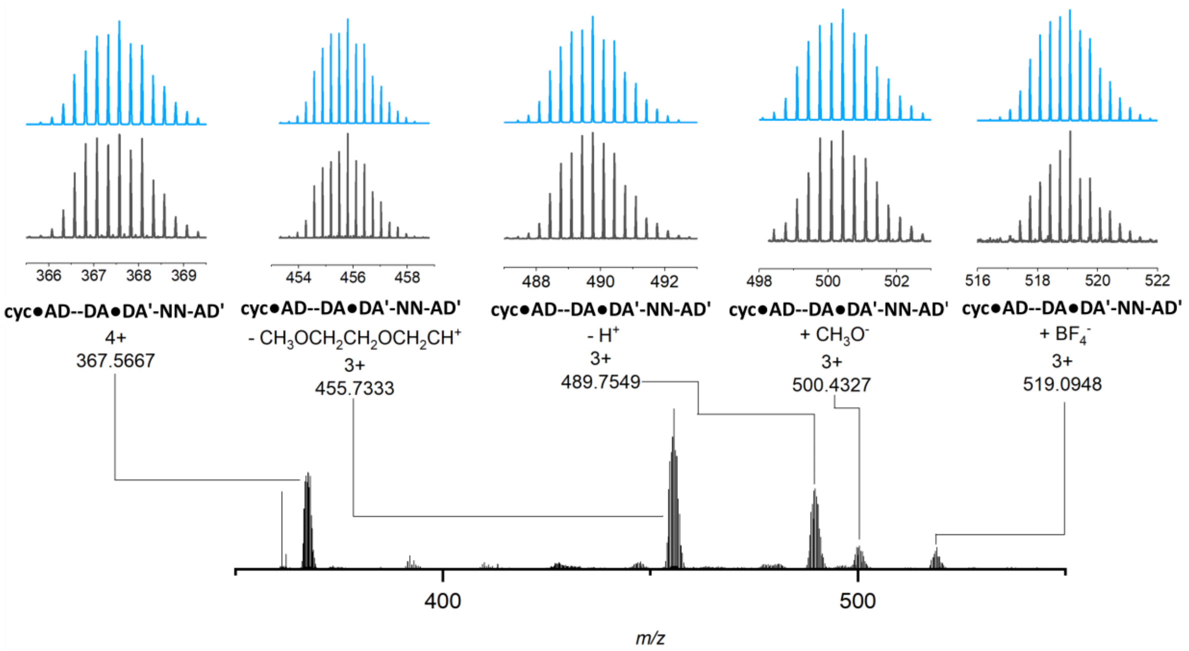

Figure S133 HR-ESI MS (DMSO/acetonitrile) of **AD--DA●DA'-NN-AD'**.

1.9.7. *cyc*●DD-DD●AA-N-AA

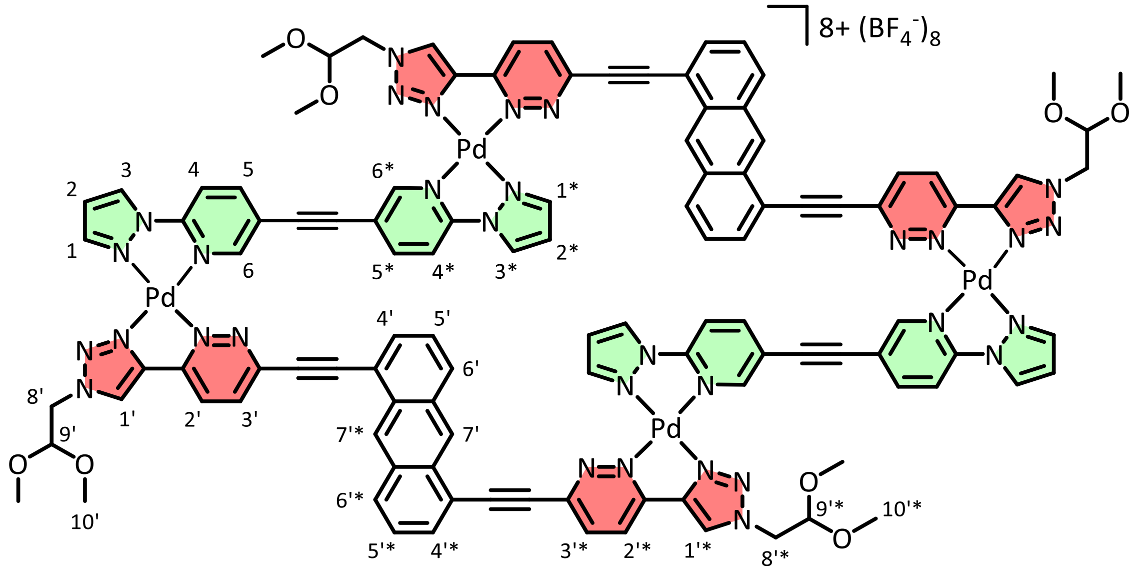

Using stock solutions, **AA-N-AA** (3.12 mg, 4.50 μmol), **DD-DD** (1.41 mg, 4.50 μmol) and $[Pd(CH_3CN)_4](BF_4)_2$ (4.00 mg, 9.00 μmol) were combined in $[D_6]$DMSO (550 μL). Equilibration occurred overnight at room temperature.

^{1}H NMR (400 MHz, $[D_6]$DMSO, 298 K) *integration given per ligand δ*: 10.10 (1H, s, H_6), 9.61 (1H, s, $H_{1'}$), 9.58-9.55 (2H, m, $H_{3,1'*}$), 9.48 (1H, br, H_{3*}), 9.21 (1H, d, J = 8.0 Hz, $H_{2'}$), 9.16 (1H, d, J = 8.0 Hz, $H_{3'*}$), 9.08 (1H, d, J = 8.0 Hz, $H_{2'*}$), 9.03 (1H, d, J = 8.0 Hz, $H_{3'}$), 8.95 (1H, d, J = 8.2 Hz, H_4), 8.92 (1H, br, $H_{7'*}$), 8.87 (1H, s, H_1), 8.82-8.78 (2H, m, $H_{1*,N}$), 8.67 (1H, d, 8.2 Hz, H_5), 8.44-8.40 (2H, m, $H_{6',N}$), 8.37 (1H, d, J = 8.1 Hz, H_{6*}), 8.28-8.24 (2H, m, $H_{5*,N}$), 8.19 (1H, d, J = 5.7 Hz, H_N), 7.88 (1H, br, H_{4*}), 7.73 (1H, br, H_N), 7.39-7.32 (3H, m, $H_{2,2*,N}$), 5.28-5.10 (4H, m, $H_{8',8'*}$), 5.06-4.95 (2H, m, $H_{9',9'*}$), 3.52-3.48 (12H, m, $H_{10',10'*}$). Note not all anthracene environments could be confidently assigned, instead $H_{4', 5', 7', 4'*, 5'*, 6'*}$ have been denoted as 'N'.

D ($\times$ 10^{-10} m^2 s^{-1}, 400 MHz, $[D_6]$DMSO, 298 K) = 0.84.

HR ESI-MS (DMSO/acetonitrile) *m/z* = 539.2742 [**cyc●DD-DD●AA-N-AA** + $3BF_4^-]^{5+}$ (calc. for $C_{112}H_{88}N_{32}O_8Pd_4B_3F_{12}^{5+}$, 539.2755), *m/z* = 695.5930 [**cyc●DD-DD●AA-N-AA** + $4BF_4^-]^{4+}$ (calc. for $C_{112}H_{88}N_{32}O_8Pd_4B_4F_{16}^{4+}$, 695.5933), *m/z* = 956.4621 [**cyc●DD-DD●AA-N-AA** + $5BF_4^-]^{3+}$ (calc. for $C_{112}H_{88}N_{32}O_8Pd_4B_5F_{20}^{3+}$, 956.4616), *m/z* = 1478.1973 [**cyc●DD-DD●AA-N-AA** + $6BF_4^-]^{2+}$ (calc. for $C_{112}H_{88}N_{32}O_8Pd_4B_6F_{24}^{2+}$, 1478.1942).

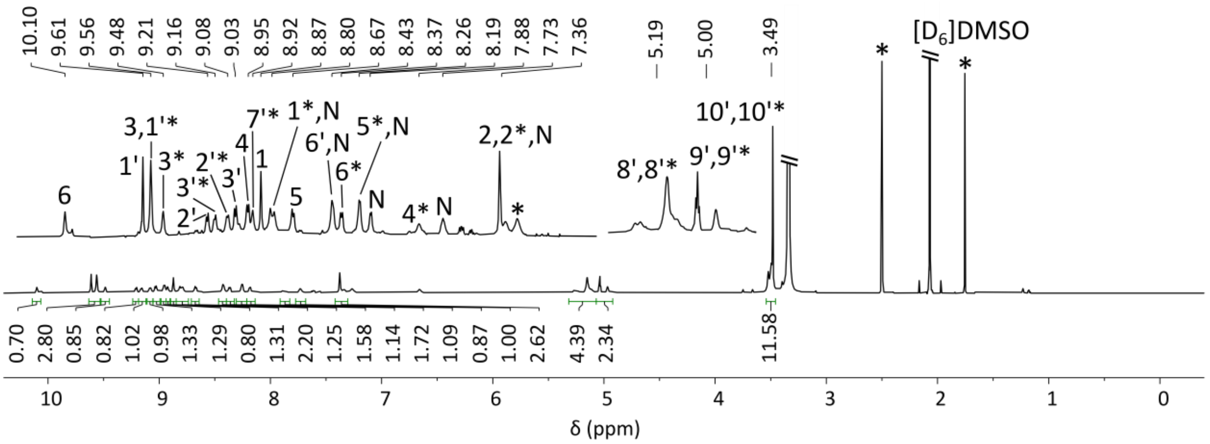

Figure S134 ^{1}H NMR spectrum (400 MHz, [D$_6$]DMSO, 298 K) of **cyc●DD-DD●AA-N-AA**. Note not all anthracene environments could be confidently assigned, instead $H_{4', 5', 7', 4'*, 5'*, 6'*}$ have been denoted as 'N'. Impurities from the Pd(II) source are indicated by *.

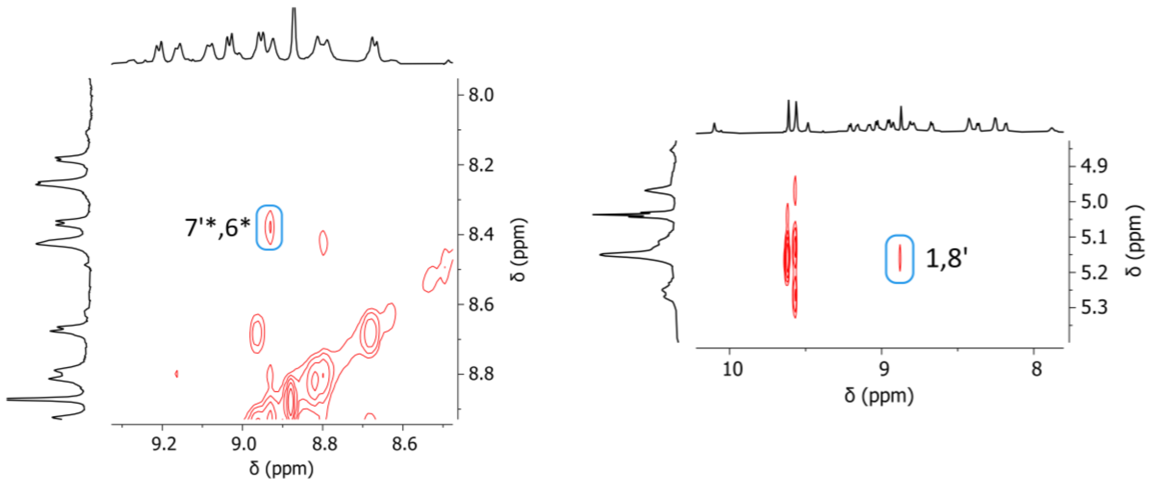

Figure S135 ^{1}H 2D NOESY NMR insets (400 MHz, [D$_6$]DMSO, 298 K, 200 ms) of **cyc●DD-DD●AA-N-AA.**

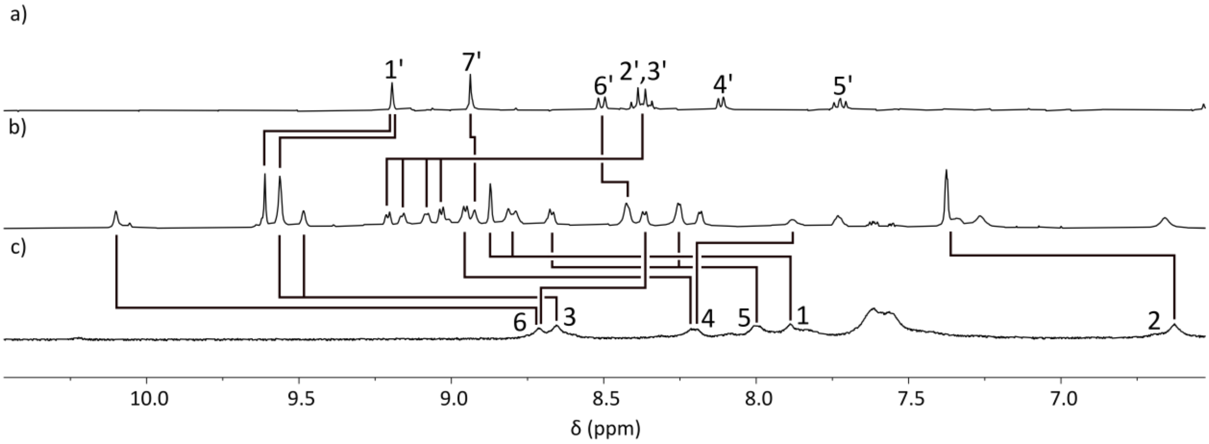

Figure S136 Partial ^{1}H NMR spectra (400 MHz, [D$_6$]DMSO, 298 K) of a) **AA-N-AA**, b) **cyc●DD-DD●AA-N-AA**, c) **DD-DD**.

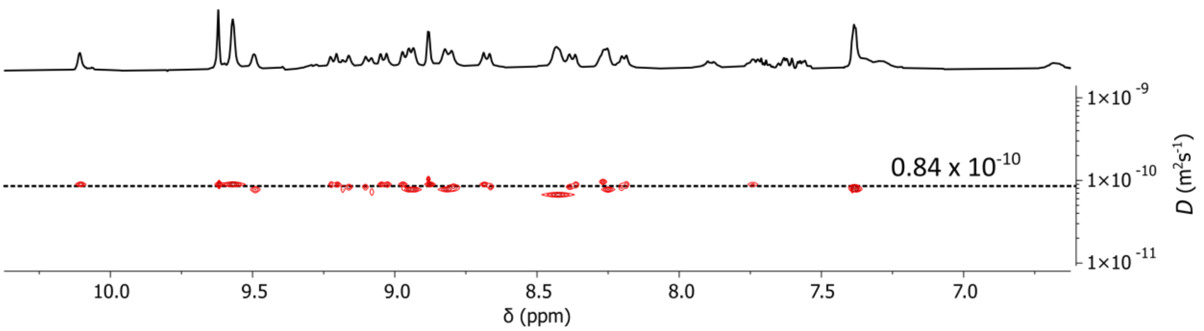

Figure S137 ^{1}H DOSY NMR spectrum (400 MHz, [D$_6$]DMSO, 298 K) of **cyc●DD-DD●AA-N-AA**.

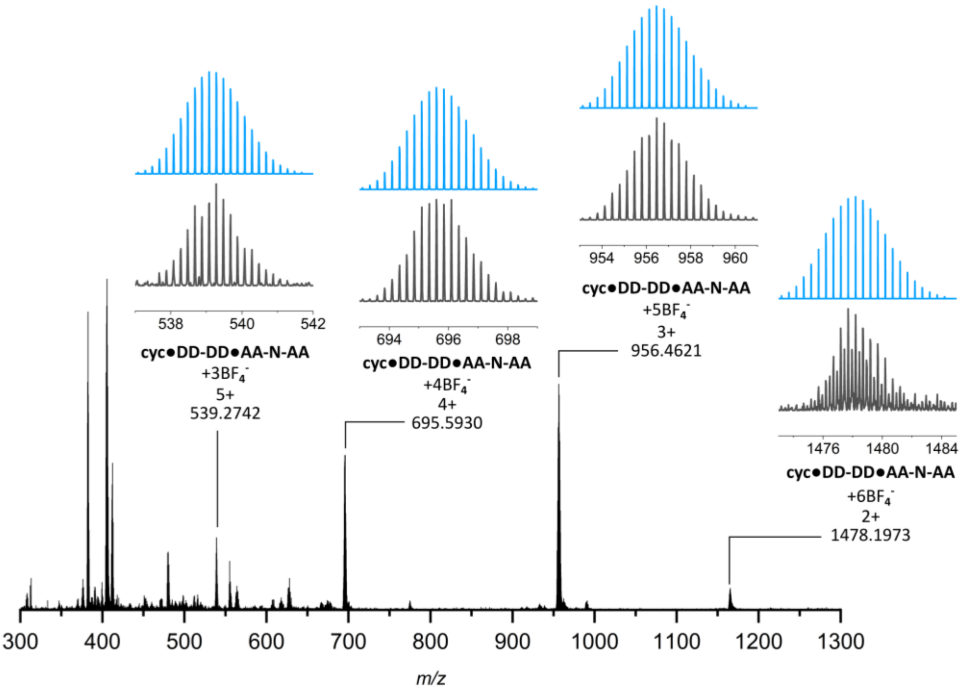

Figure S138 HR-ESI MS (DMSO/acetonitrile) of **cyc●DD-DD●AA-N-AA**.

2. Unsuccessful self-assemblies

Characterisation data for combinations of ligands with Pd(II) which did not give single identifiable products are detailed below. The ^{1}H NMR spectrum is included for each attempted complexation, in addition to MS data. Most ^{1}H NMR spectra are extremely complicated with significant overlap between environments and/or broadening of peaks, making solution-phase characterisation quite challenging. The MS data provided further insight into what may be present in each case, but it is important to note that we cannot be sure whether the species identified are simply fragments or rearrangements that occur in the MS process. Nevertheless, all available data is reported here for reference.

2.1.1. [Pd(DA'-N')(AD-N')]$_2$(BF$_4$)$_4$

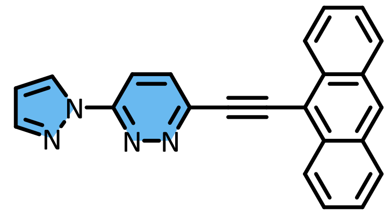

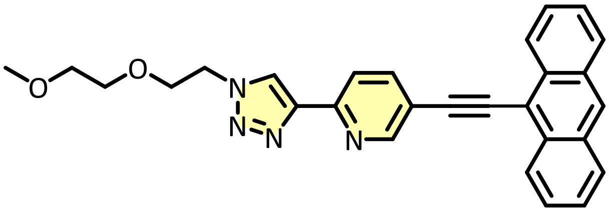

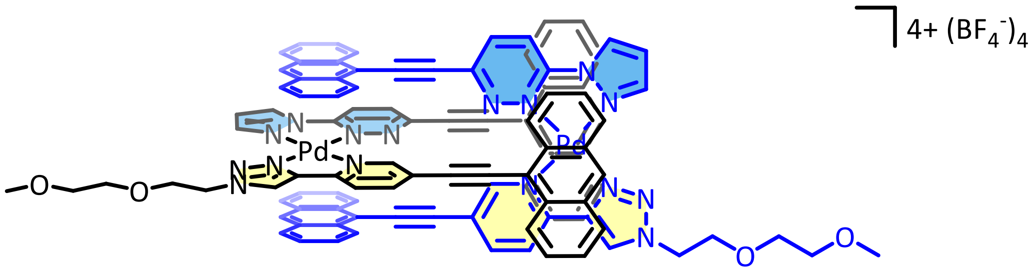

Using stock solutions, **DA'-N'** (2.00 mg, 5.77 µmol), **AD-N'** (2.57 mg, 5.77 µmol) and [Pd(CH_3CN)$_4$](BF_4)$_2$ (2.57 mg, 5.77 µmol) were combined in [D$_6$]DMSO (600 µL). The combination did not result in the self-assembly of a single species.

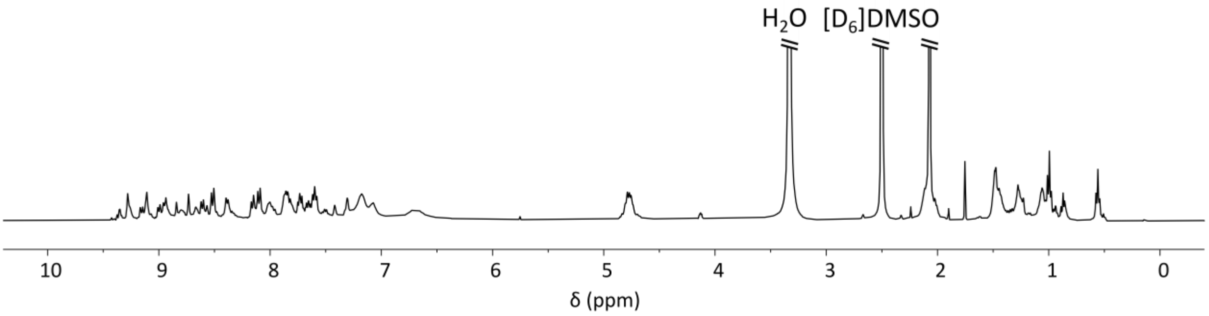

Figure S139 ^{1}H NMR spectrum (400 MHz, [D$_6$]DMSO, 298 K) of the attempted synthesis of **[Pd(DA'-N')(AD-N')]$_2$(BF$_4$)$_4$**.

2.1.2. N'-AD'●AD-DA●DA'-N'

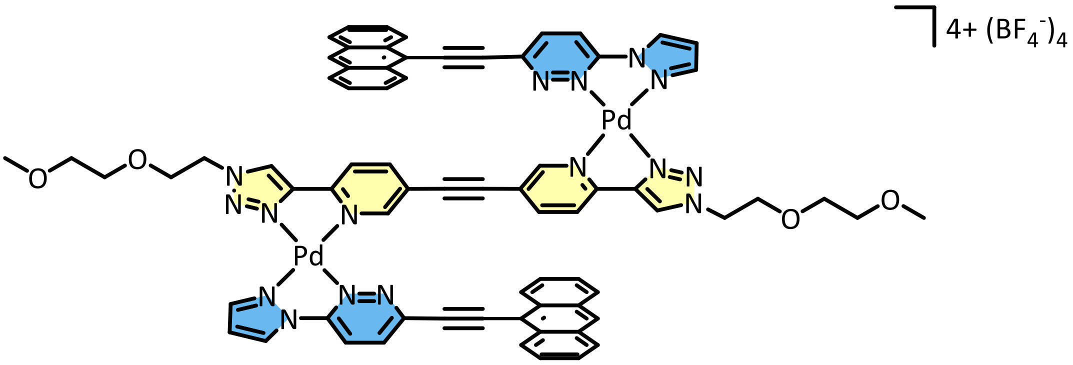

Using stock solutions, **AD-DA** (1.17 mg, 2.25 µmol), **DA'-N'** (1.56 mg, 4.50 µmol) and $[Pd(CH_3CN)_4](BF_4)_2$ (2.00 mg, 4.50 µmol) were combined in [D$_6$]DMSO (550 µL). The combination did not result in the self-assembly of a single species. The ^{1}H NMR and MS data suggests **N'-AD'●AD-DA●DA'-N'** does form and accounts for roughly 69% of the mixture (based on ^{1}H NMR integration), in addition to other complexes. The MS suggests the presence of [Pd(**DA'-N'**)$_2$]$^{2+}$. It is possible polymeric **AD-DA** species may be present, but identification of these larger species was not possible.

Nanospray MS (DMSO) *m/z* = 346.1217 [**DA'-N'**]$^{+\bullet}$ (calc. for $C_{23}H_{14}N_4^{+\bullet}$, 346.1213), 356.0731 [**N'-AD'●AD-DA●DA'-N'**]$^{4+}$ (calc. for $C_{72}H_{58}N_{16}O_4Pd_2^{4+}$, 356.0720), 400.0736 [Pd(**DA'-N'**)$_2$]$^{2+}$ (calc. for $C_{46}H_{28}N_8Pd^{2+}$, 400.0732), 485.1327 [Pd(**DA'-N'**)(**AD-DA**)]$^{2+}$ (calc. for $C_{49}H_{44}N_{12}O_4Pd^{2+}$, 485.1316), 503.7649 [**N'-AD'●AD-DA●DA'-N'** + BF_4^-]$^{3+}$ (calc. for $C_{72}H_{58}N_{16}O_4Pd_2BF_4^{3+}$, 503.7638), 799.1498 [**N'-AD'●AD-DA●DA'-N'** + $2BF_4^-$]$^{2+}$ (calc. for $C_{72}H_{58}N_{16}O_4Pd_2B_2F_8^{2+}$, 799.1474).

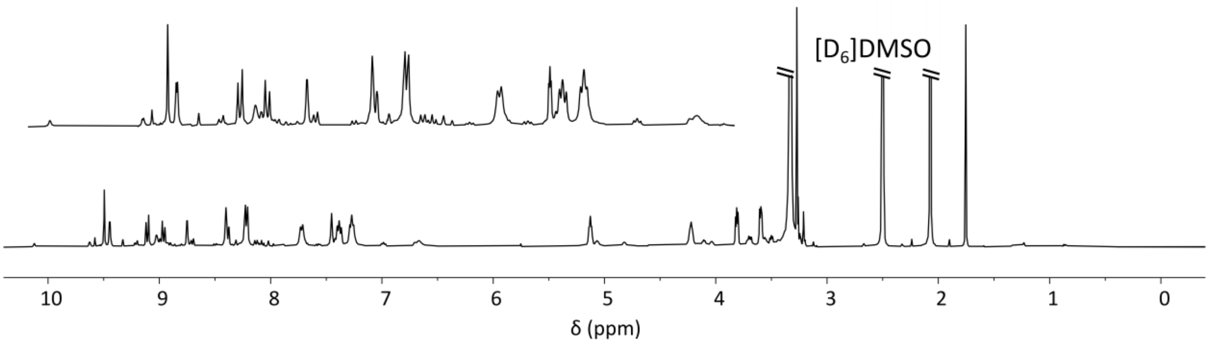

Figure S140 ^{1}H NMR spectrum (400 MHz, [D$_6$]DMSO, 298 K) of the attempted synthesis of **N'-AD'●AD-DA●DA'-N'**.

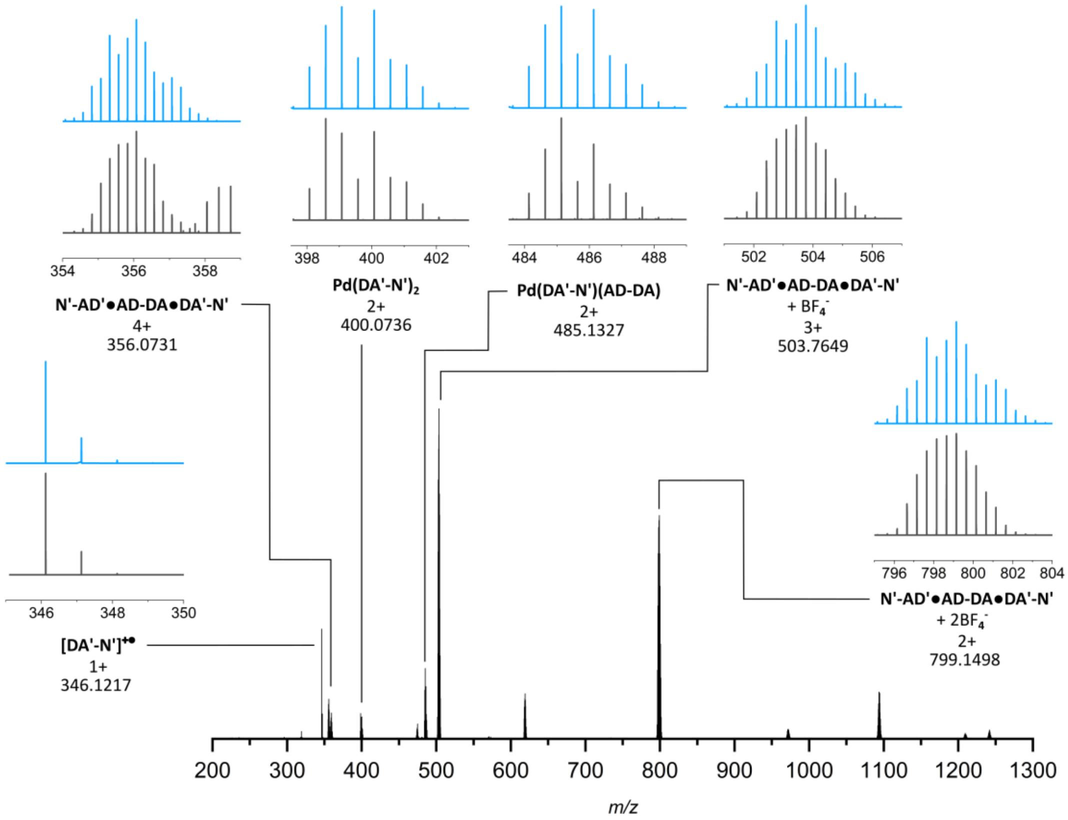

Figure S141 Nanospray MS (DMSO) of the attempted synthesis of **N'-AD'•AD-DA•DA'-N'**.

2.1.3. EP-AA'•DD-DD•AA'-EP

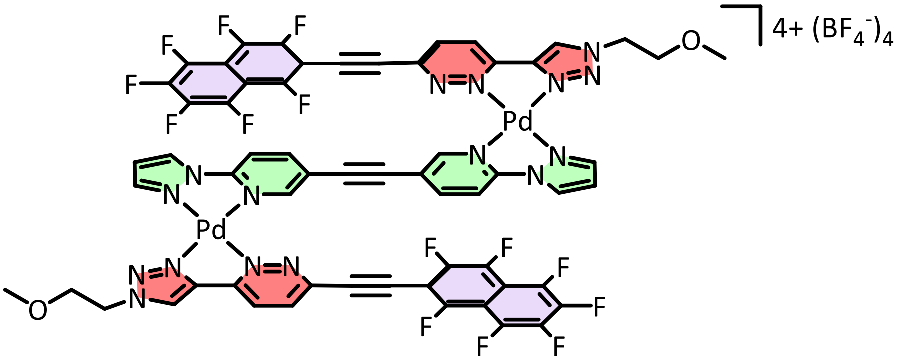

Using stock solutions, **DD-DD** (0.70 mg, 2.3 µmol), **AA'-EP** (2.17 mg, 4.50 µmol) and $[Pd(CH_3CN)_4](BF_4)_2$ (2.00 mg, 4.50 µmol) were combined in $[D_6]$DMSO (550 µL). The combination did not result in the self-assembly of a single species. The ^{1}H NMR and MS data suggests **EP-AA'•DD-DD•AA'-EP** does form but in addition to several other complexes. It is possible that both HH and HT isomers of **EP-AA'•DD-DD•AA'-EP** are present. We estimate based on ^{1}H NMR integration that the expected HH **EP-AA'•DD-DD•AA'-EP** foldamer may account for roughly 50% of the mixture. The MS also suggests the presence of $[Pd($**AA'-EP**$)_2]^{2+}$.

Nanospray MS (DMSO) *m/z* = 372.0202 [**EP-AA'•DD-DD•AA'-EP**]$^{4+}$ (calc. for $C_{60}H_{32}N_{16}O_2Pd_2F_{14}{}^{4+}$, 372.0181), 449.5508 [Pd(**DD-DD**)(**AA'-EP**)]$^{2+}$ (calc. for $C_{39}H_{22}N_{11}OPdF_7{}^{2+}$, 449.5463), 525.0291 [**EP-AA'•DD-DD•AA'-EP** + BF_4^-]$^{3+}$ (calc. for $C_{60}H_{32}N_{16}O_2Pd_2BF_4{}^{3+}$, 525.0252), 534.0319 $[Pd($**AA'-EP**$)_2]^{2+}$ (calc.

for $C_{42}H_{20}N_{10}O_2PdF_{14}^{2+}$, 534.0286), 986.1039 [Pd(**DD-DD**)(**AA'-EP**) + BF_4^-]$^{1+}$ (calc. for $C_{39}H_{22}N_{11}OPdBF_{11}^{+}$, 986.0956).

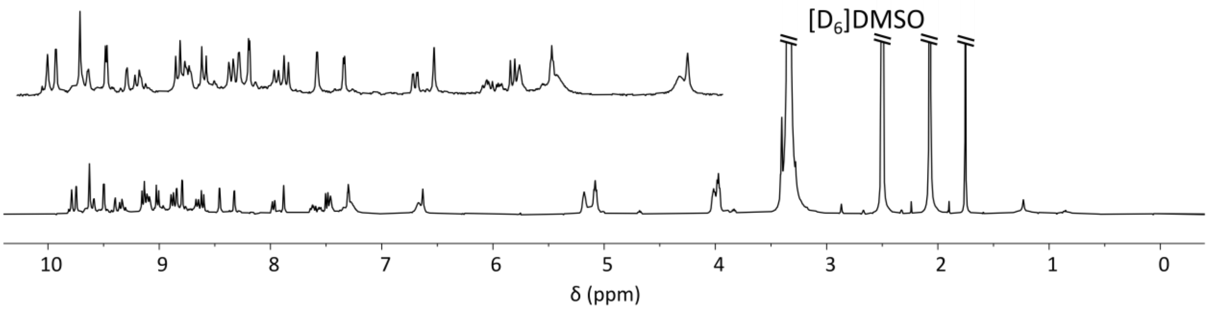

Figure S142 ^{1}H NMR spectrum (400 MHz, [D$_6$]DMSO, 298 K) of the attempted synthesis of **EP-AA'●DD-DD●AA'-EP**.

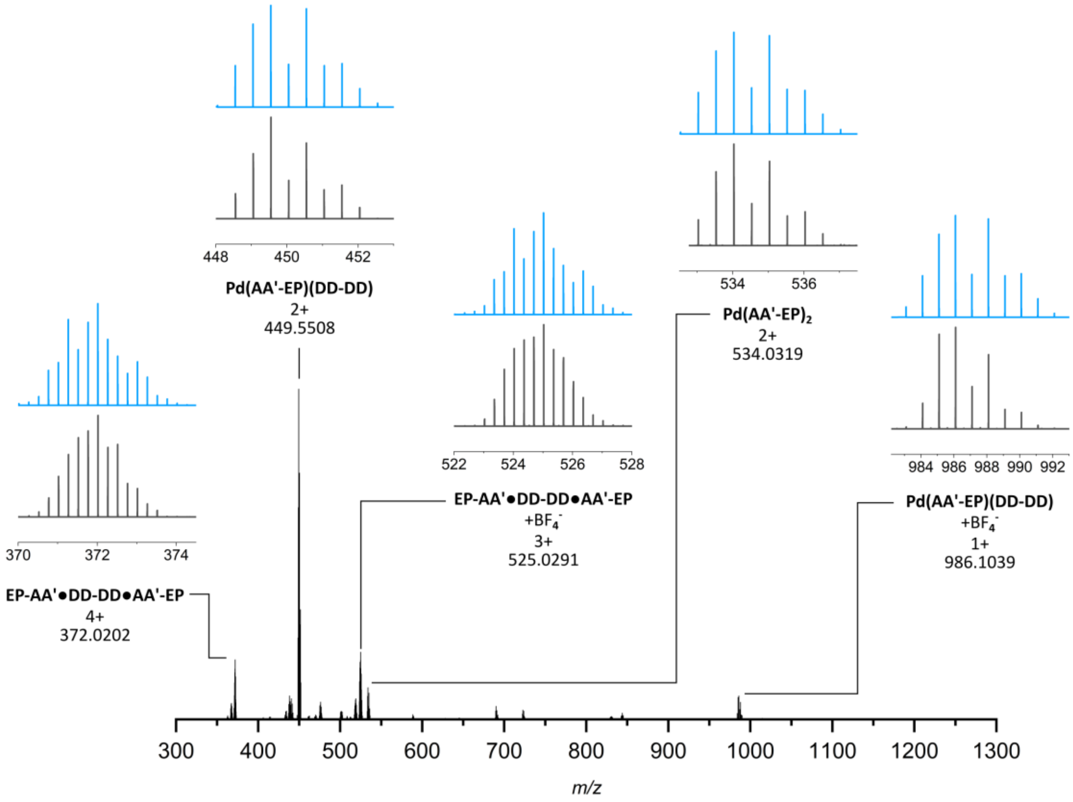

Figure S143 Nanospray MS (DMSO) of the attempted synthesis of **EP-AA'●DD-DD●AA'-EP**.

2.1.4. *cyc*●DD-DD●DD-N-DD

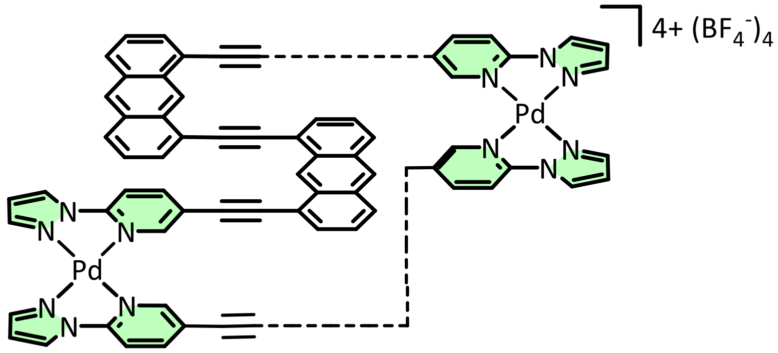

Using stock solutions, **DD-DD** (0.70 mg, 2.3 µmol), **DD-NN-DD** (1.60 mg, 2.30 µmol) and $[Pd(CH_3CN)_4](BF_4)_2$ (2.00 mg, 4.50 µmol) were combined in [D$_6$]DMSO (550 µL). The combination did not result in the self-assembly of a single species. The intended **cyc●DD-DD●DD-N-DD** complex could not be identified via ^{1}H NMR or even MS. Both ^{1}H NMR and MS suggest the formation of [Pd$_2$(**DD-DD**)$_2$]$^{4+}$, as we have observed previously.[6] Note DD-NN-DD has extremely poor solubility, and solid residue remained in the mixture following the addition of $[Pd(CH_3CN)_4](BF_4)_2$.

Nanospray MS (DMSO) *m/z* = 209.5082 [Pd$_2$(**DD-DD**)$_2$]$^{4+}$ (calc. for $C_{36}H_{24}N_{12}Pd_2^{4+}$, 209.5090), 279.0937 [Pd$_2$(**DD-DD**)$_2$ - H$^+$]$^{3+}$ (calc. for $C_{36}H_{23}N_{12}Pd_2^{3+}$, 279.0075), 312.1123 [**DD-DD**]$^{+\bullet}$ (calc. for $C_{18}H_{12}N_6^{+\bullet}$, 312.1118), 409.0712 [Pd(**DD-NN-DD**)]$^{2+}$ (calc. for $C_{50}H_{28}N_6Pd^{2+}$, 409.0700), 438.0154 [Pd$_2$(**DD-DD**)$_2$ + 2F$^-$]$^{2+}$ (calc. for $C_{36}H_{24}N_{12}Pd_2F_2^{2+}$, 438.0139), 539.0364 [Pd(**DD-DD**) + CH_3OH + BF_4^-]$^{1+}$ (calc. for $C_{19}H_{16}N_6OPdBF_4^{1+}$, 539.0448).

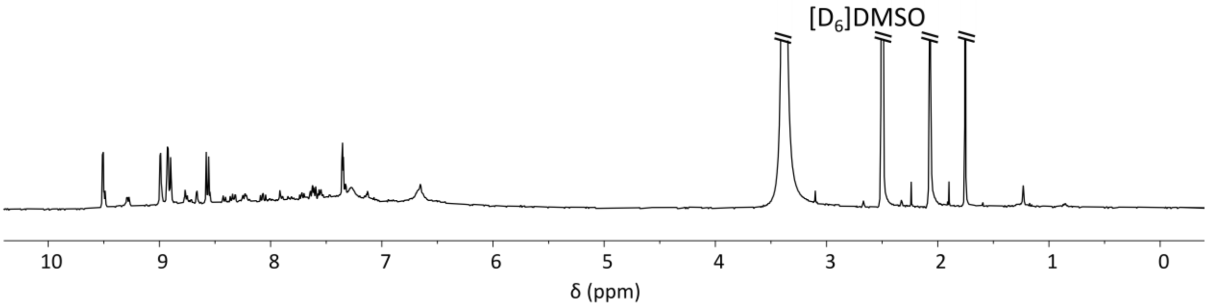

Figure S144 ^{1}H NMR spectrum (400 MHz, [D$_6$]DMSO, 298 K) of the attempted synthesis of **cyc●DD-DD●DD-N-DD**.

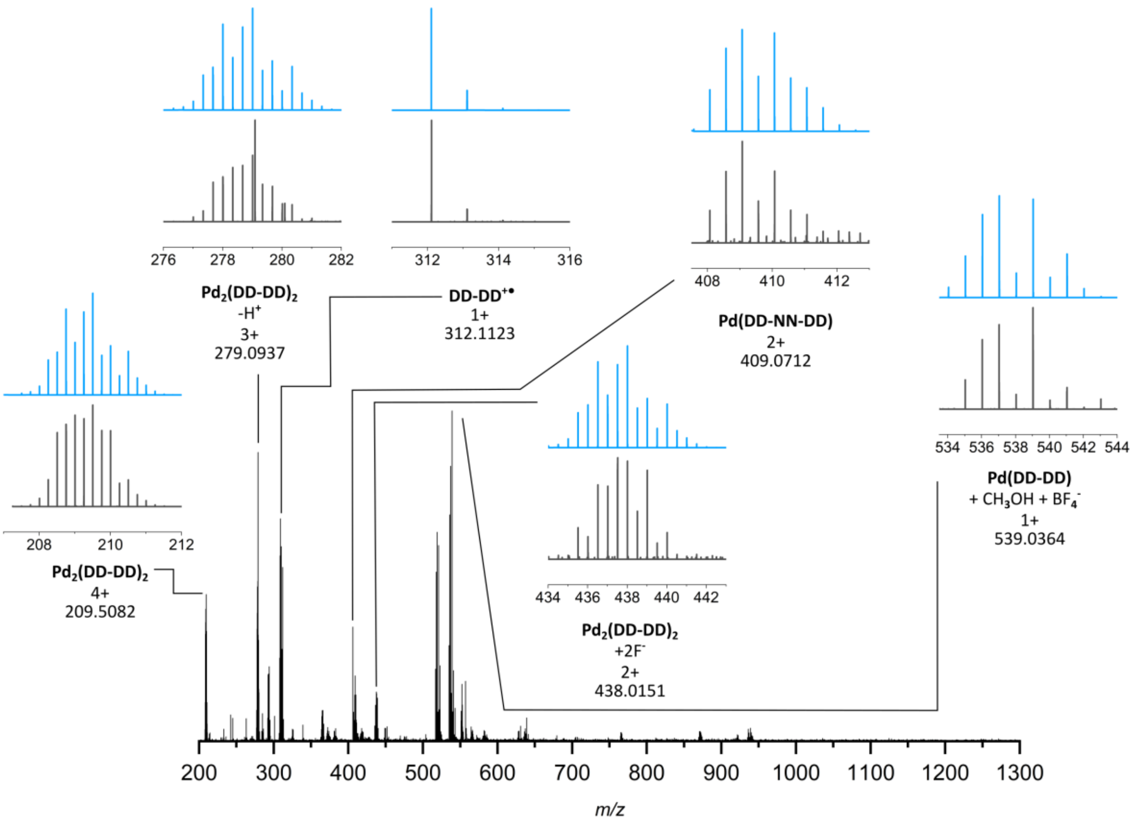

Figure S145 Nanospray MS (DMSO) of the attempted synthesis of **cyc●DD-DD●DD-N-DD**.

2.1.5. *cyc*●AD-DA●DD-N-DD

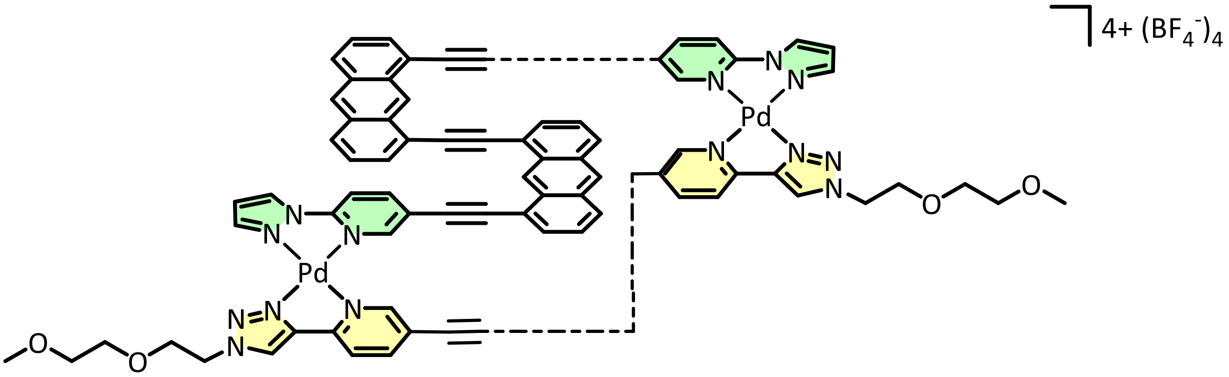

Using stock solutions, **AD-DA** (0.59 mg, 1.1 µmol), **DD-NN-DD** (0.80 mg, 1.1 µmol) and $[Pd(CH_3CN)_4](BF_4)_2$ (1.00 mg, 2.23 µmol) were combined in $[D_6]$DMSO (550 µL). The combination did not result in the self-assembly of a single species. The ^{1}H NMR and MS data suggests **cyc●AD-DA●DD-N-DD** does form but in addition to several other species. Due to the complexity of the spectrum, it is not possible to estimate the proportion of the mixture which is the intended foldamer. The MS suggests the presence of [Pd(**AD-DA**)$_2$]$^{2+}$. It is possible polymeric **AD-DA** species may be present, but identification of these larger species was not possible.

Nanospray MS (DMSO) *m/z* = 361.0714 [***cyc*●AD-DA●DD-N-DD**]$^{4+}$ (calc. for $C_{76}H_{58}N_{14}O_4Pd_2^{4+}$, 361.0704), 409.0706 [Pd(**DD-NN-DD**)]$^{2+}$ (calc. for $C_{50}H_{28}N_6Pd^{2+}$, 409.0700), 480.7598 [***cyc*●AD-DA●DD-N-DD** – H$^+$]$^{3+}$ (calc. for $C_{76}H_{57}N_{14}O_4Pd_2^{3+}$, 480.7587), 510.0967 [***cyc*●AD-DA●DD-N-DD** + BF_4^-]$^{3+}$ (calc. for $C_{76}H_{58}N_{14}O_4Pd_2BF_4^{3+}$, 510.0956), 571.1913 [Pd(**AD-DA**)$_2$]$^{2+}$ (calc. for $C_{52}H_{60}N_{16}O_8Pd^{2+}$, 571.1902).

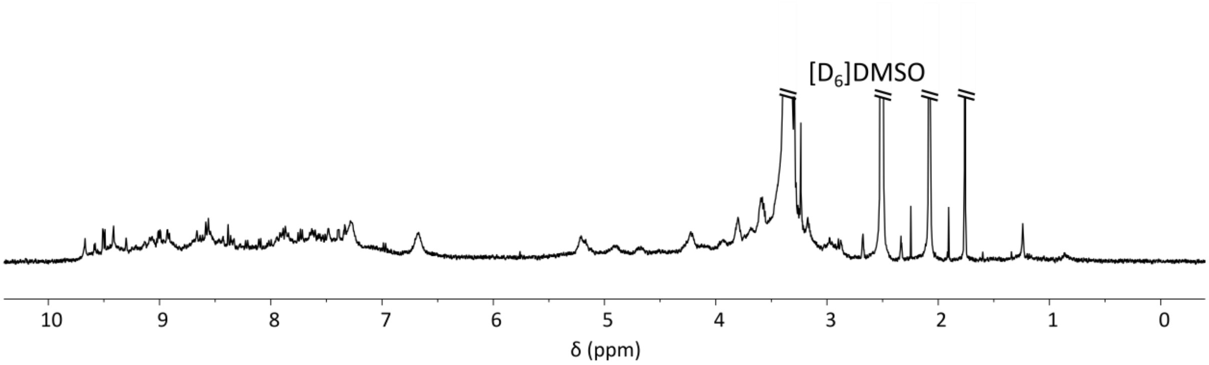

Figure S146 ^{1}H NMR spectrum (400 MHz, [D$_6$]DMSO, 298 K) of the attempted synthesis of **cyc●AD-DA●DD-N-DD**.

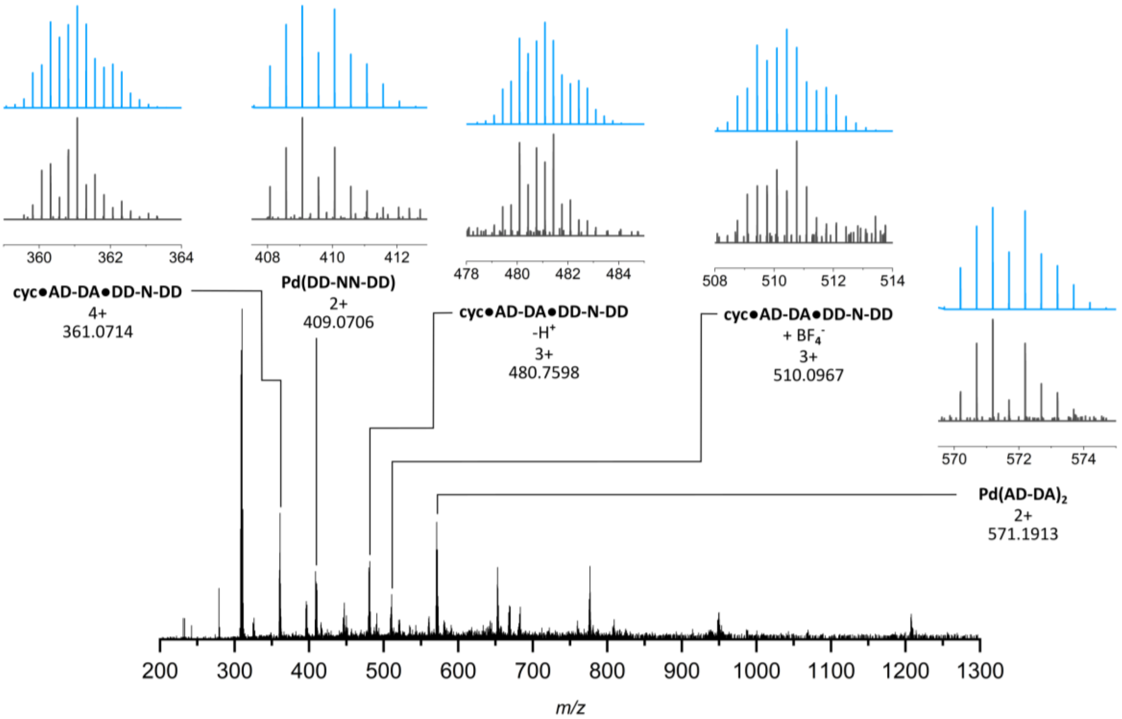

Figure S147 Nanospray MS (DMSO) of the attempted synthesis of **cyc●AD-DA●DD-N-DD**.

2.1.6. N-DA'●AD-N-DA●DA'-N

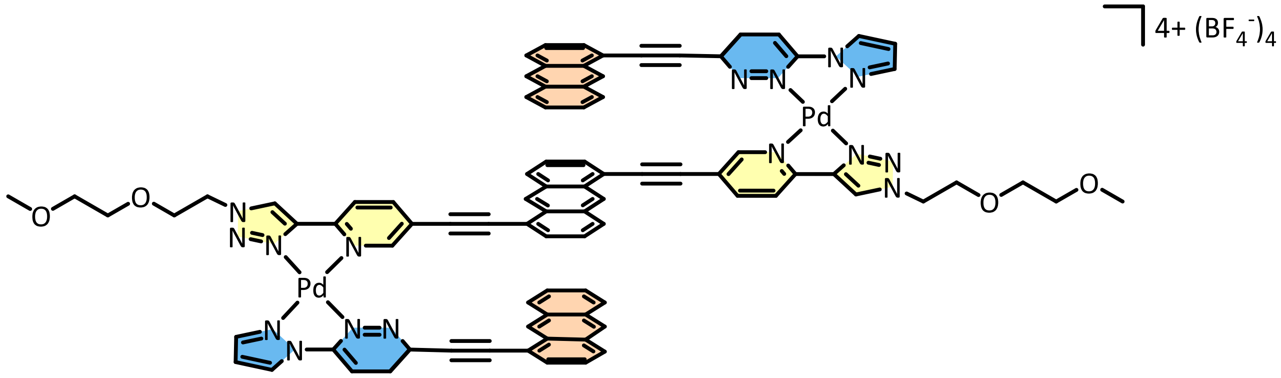

Using stock solutions, **AD-N-DA** (1.62 mg, 2.25 µmol), **DA'-N** (1.56 mg, 4.50 µmol) and $[Pd(CH_3CN)_4](BF_4)_2$ (2.00 mg, 4.50 µmol) were combined in [D$_6$]DMSO (550 µL). The combination did not result in the self-assembly of a single species. The ^{1}H NMR and MS data suggests **N-DA'●AD-N-DA●DA'-N** does form but in addition to several other species. Due to the complexity of the spectrum, it is not possible to estimate the proportion of the mixture which is the intended foldamer. The MS suggests the presence of [Pd(**DA'-N**)$_2$]$^{2+}$ and free **DA'-N**. It is possible polymeric **AD-N-DA** species may be present, but identification of these larger species was not possible.

Nanospray MS (DMSO) *m/z* = 346.1219 [**DA'-N**]$^{+\bullet}$ (calc. for $C_{23}H_{14}N_4^{+\bullet}$, 346.1213), 399.0749 [Pd(**DA'-N**)$_2$]$^{2+}$ (calc. for $C_{46}H_{28}N_8Pd^{2+}$, 399.0730), 570.1198 [**N-AD'●AD-N-DA●DA'-N** + BF_4^-]$^{3+}$ (calc. for $C_{88}H_{66}N_{16}O_4Pd_2BF_4^{3+}$, 570.1221), 885.1521 [Pd(**DA'-N**)$_2$ + BF_4^-]$^{1+}$ (calc. for $C_{46}H_{28}N_8PdBF_4^{1+}$, 885.1496).

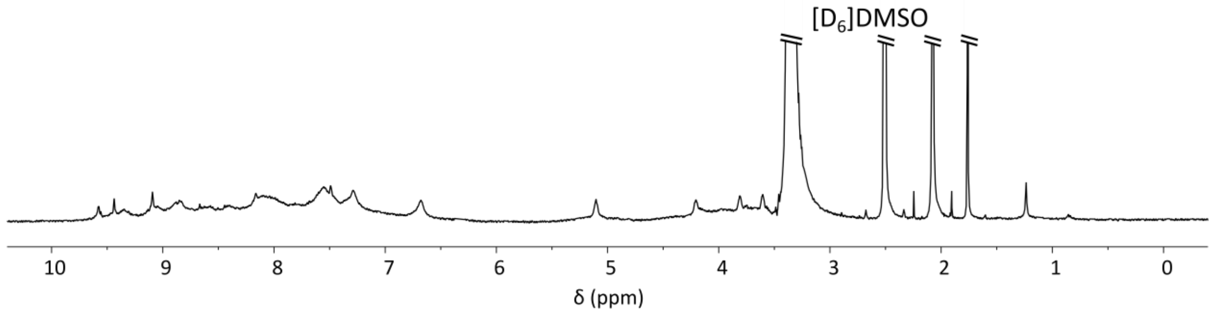

Figure S148 ^{1}H NMR spectrum (400 MHz, [D$_6$]DMSO, 298 K) of the attempted synthesis of **N-DA'●AA-N-AA●DA'-N**.

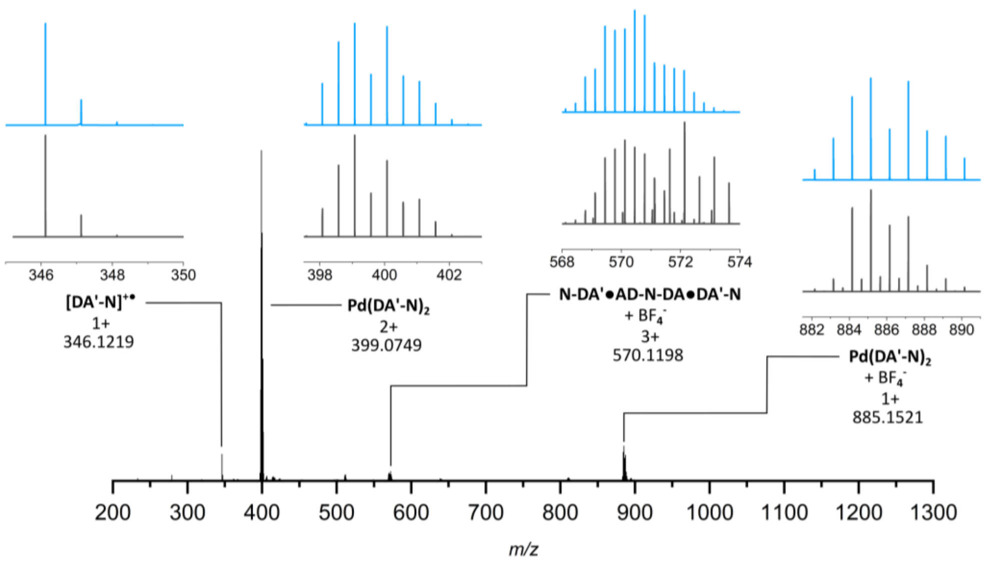

Figure S149 Nanospray MS (DMSO) of the attempted synthesis of **N-DA'●AD-NN-DA●DA'-N.**

2.1.7. N-DA'●AD-NN-DA●DA'-N

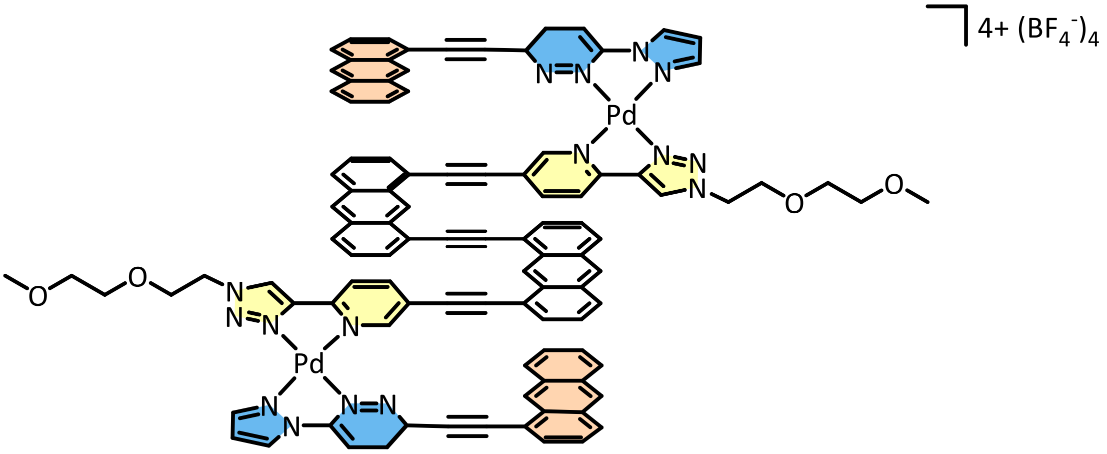

Using stock solutions, **AD-NN-DA** (1.30 mg, 1.41 μmol), **DA'-N** (0.98 mg, 2.8 μmol) and $[Pd(CH_3CN)_4](BF_4)_2$ (1.26 mg, 2.82 μmol) were combined in $[D_6]$DMSO (550 μL). The combination did not result in the self-assembly of a single species. The ^{1}H NMR and MS data suggests **N-DA'●AD-NN-DA●DA'-N** does form but in addition to several other species. Due to the complexity of the spectrum, it is not possible to estimate the proportion of the mixture which is the intended foldamer. The MS suggests the presence of [Pd(**DA'-N**)$_2$]$^{2+}$ and free **DA'-N**. It is possible polymeric **AD-NN-DA** species may be present, but identification of these larger species was not possible.

HR ESI-MS (DMSO/acetonitrile) *m/z* = 346.1235 [**DA'-N**]$^{+\bullet}$ (calc. for $C_{23}H_{14}N_4^{+\bullet}$, 346.1213), 399.0761 [Pd(**DA'-N**)$_2$]$^{2+}$ (calc. for $C_{46}H_{28}N_8Pd^{2+}$, 399.0730), 456.1060 [**N-AD'●AD-NN-DA●DA'-N**]$^{4+}$ (calc. for $C_{104}H_{74}N_{16}O_4Pd_2^{4+}$, 456.1048), 492.7668 [**N-AD'●AD-N-DA●DA'-N** - H$^+$]$^{3+}$ (calc. for $C_{104}H_{73}N_{16}O_4Pd_2^{3+}$, 492.7632), 512.1365 [Pd(**AD-NN-DA**)]$^{2+}$ (calc. for $C_{58}H_{46}N_8O_4Pd^{2+}$, 512.1349), 637.1426 [**N-AD'●AD-N-DA●DA'-N** + BF$_4^-$]$^{3+}$ (calc. for $C_{104}H_{74}N_{16}O_4Pd_2BF_4^{3+}$, 637.1410), 685.1979 [Pd(**DA'-N**)(**AD-NN-DA**)]$^{2+}$

(calc. for $C_{81}H_{60}N_{12}O_4Pd^{2+}$, 685.1958), 999.2137 [**N-AD'●AD-N-DA●DA'-N** + $2BF_4^-$]$^{2+}$ (calc. for $C_{104}H_{74}N_{16}O_4Pd_2B_2F_8^{2+}$, 999.2138).

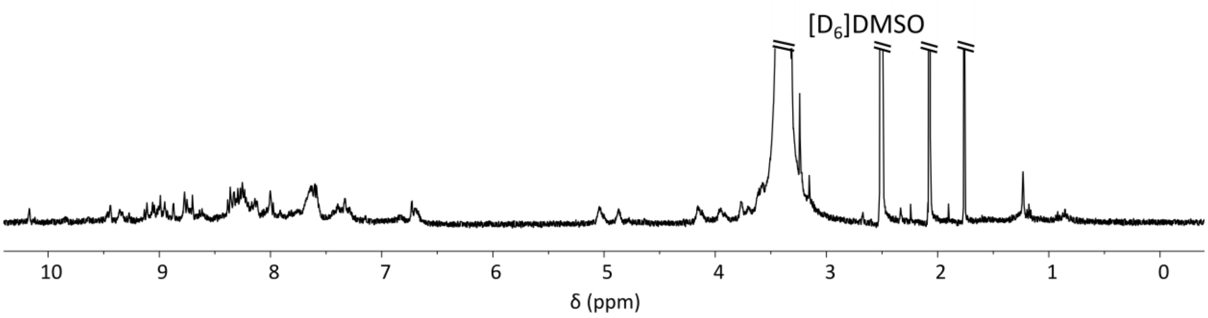

Figure S150 ^{1}H NMR spectrum (400 MHz, [D$_6$]DMSO, 298 K) of the attempted synthesis of **N-DA'●AD-NN-DA●DA'-N**.

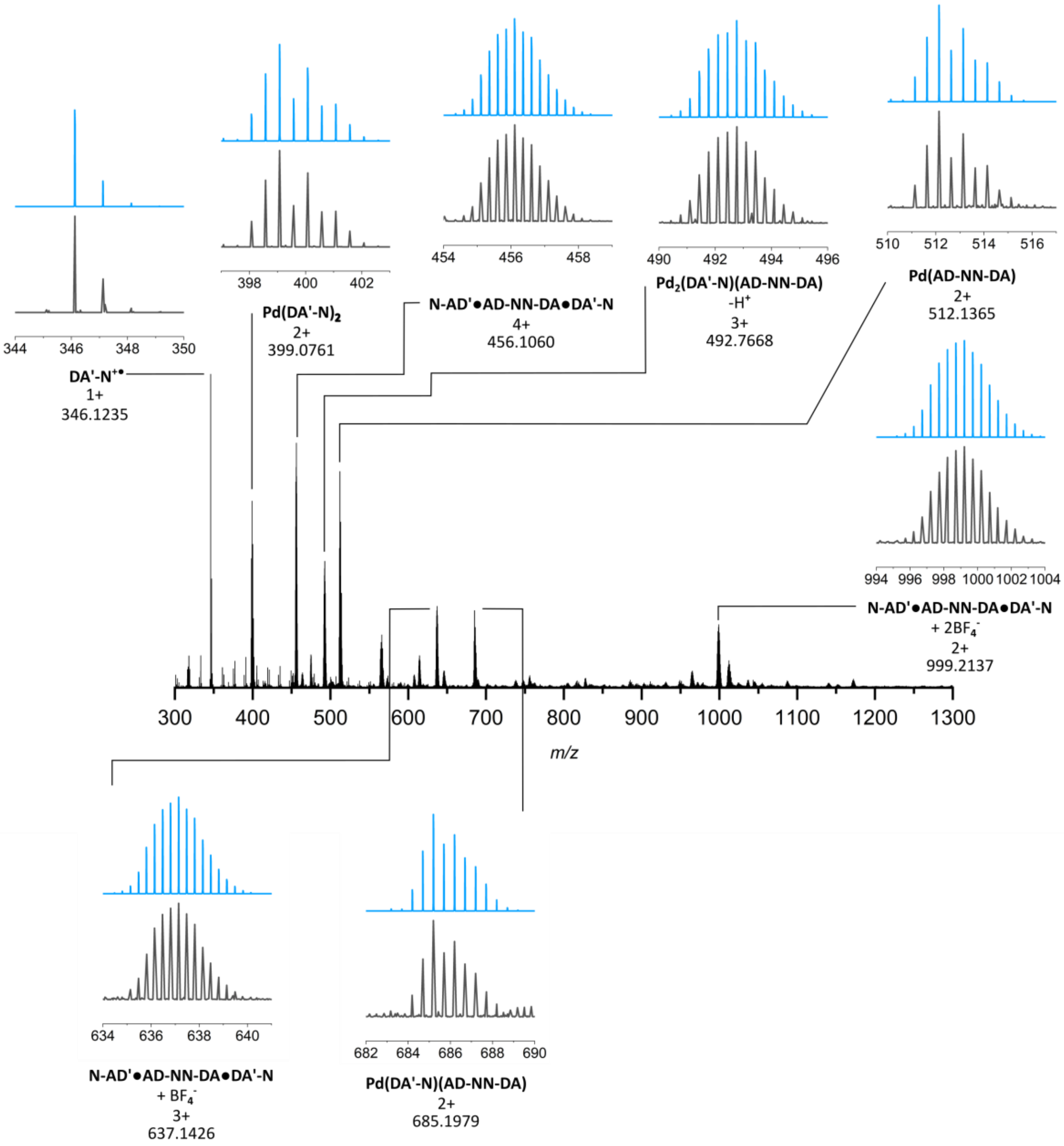

Figure S151 HR ESI-MS (DMSO/acetonitrile) of the attempted synthesis of **N-DA'●AD-NN-DA●DA'-N.**

2.1.8. *cyc*●DA'-NN-AD'●AD-NN-DA

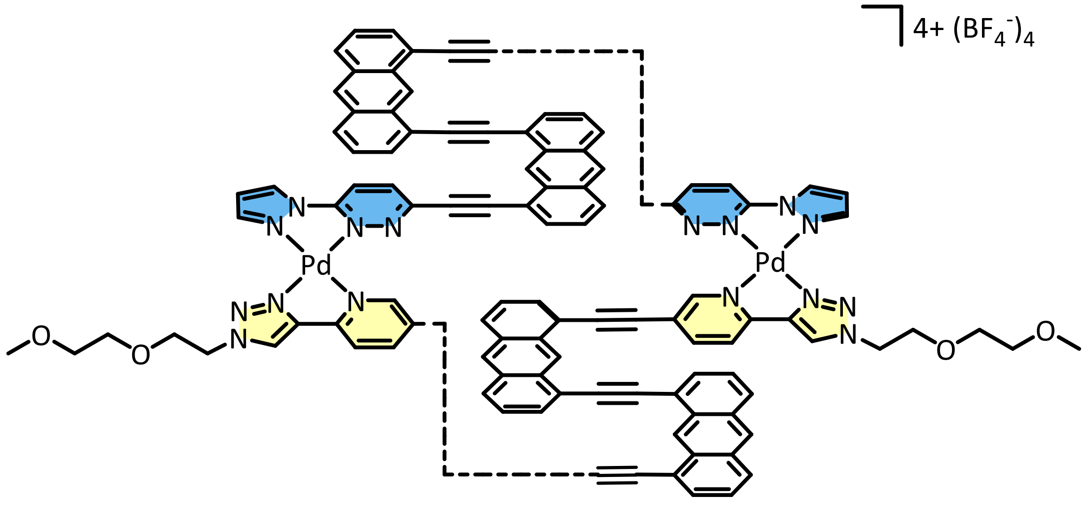

Using stock solutions, **AD-NN-DA** (0.60 mg, 0.70 µmol), **DA'-NN-AD'** (0.47 mg, 0.70 µmol) and $[Pd(CH_3CN)_4](BF_4)_2$ (0.58 mg, 1.3 µmol) were combined in $[D_6]$DMSO (550 µL). The combination did not result in the self-assembly of a single species. The intended **cyc●DA'-NN-AD'●AD-NN-DA** complex could not be identified via MS. The broadness of peaks in the ^{1}H NMR and identifiable species in the MS suggest polymeric **AD-NN-DA** species may be present, but identification of significantly larger species was not possible.

HR ESI-MS (DMSO/acetonitrile) *m/z* = 512.1326 [Pd(**AD-NN-DA**)]$^{2+}$ (calc. for $C_{58}H_{46}N_8O_4Pd^{2+}$, 512.1349), 566.0870 [$Pd_2$(**AD-NN-DA**) – 2H$^+$]$^{2+}$ (calc. for $C_{58}H_{44}N_8O_4Pd_2^{2+}$, 566.0863), 683.5140 [$Pd_2$(**AD-NN-DA**)$_2$ – H$^+$]$^{3+}$ (calc. for $C_{116}H_{91}N_{16}O_8Pd_2^{2+}$, 683.5107).

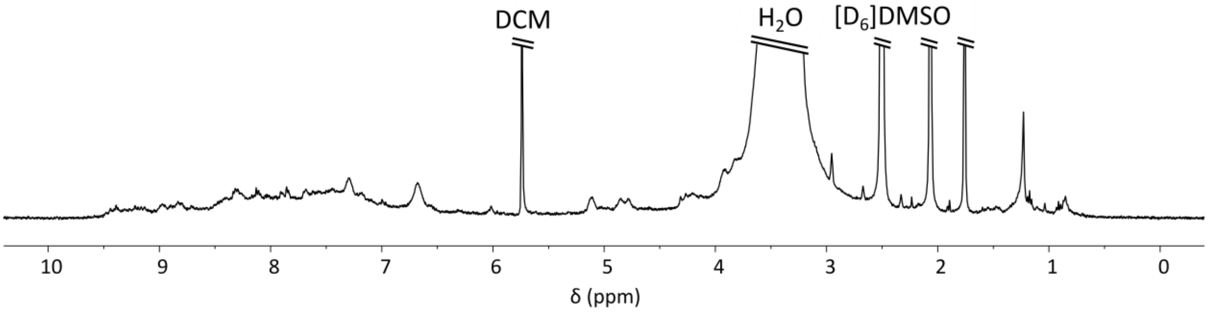

Figure S152 ^{1}H NMR spectrum (400 MHz, $[D_6]$DMSO, 298 K) of the attempted synthesis of **cyc●DA'-NN-AD'●AD-NN-DA**.

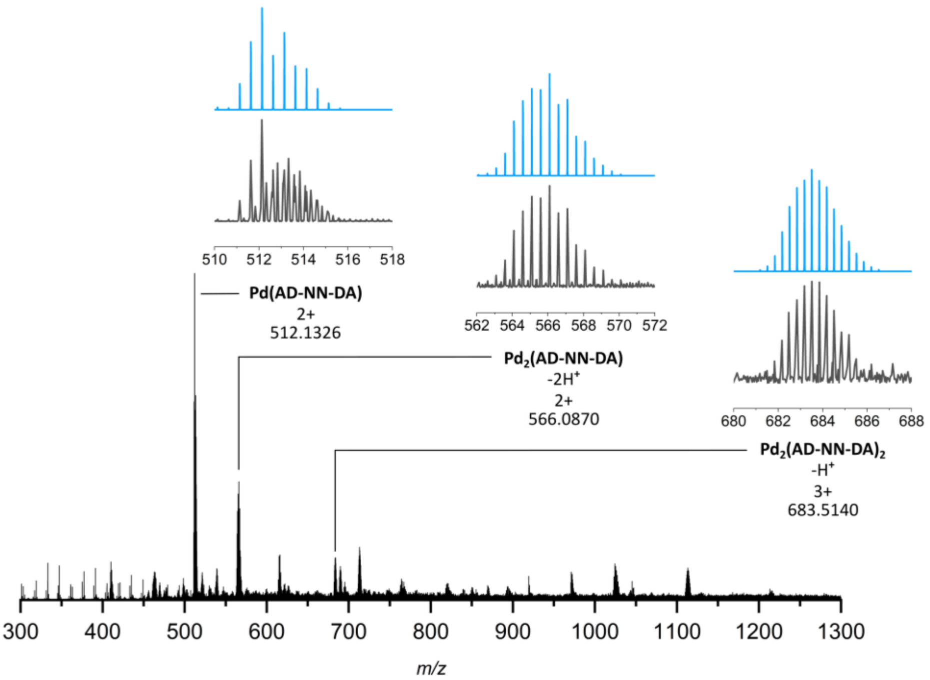

Figure S153 HR ESI-MS (DMSO/acetonitrile) of the attempted synthesis of **cyc●DA'-NN-AD'●AD-NN-DA.**

3. Stacked Spectra

3.1.1. [2]Clippane mixing studies

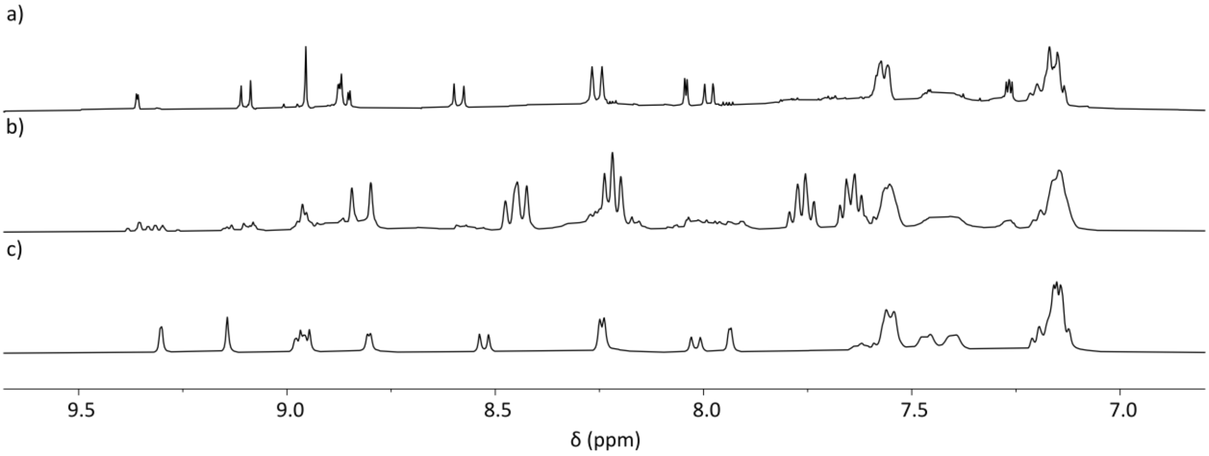

Figure S154 Partial ^{1}H NMR spectrum (400 MHz, [D$_6$]DMSO, 298 K) of a) [Pd(**AD--N'**)(**DA'--N'**)]$_2$(BF$_4$)$_4$, b) the mixture resulting from the combination of [Pd(**DD--N'**)(**AA'--N'**)]$_2$(BF$_4$)$_4$, **AD--N'** and **DA'--N'** in a 1:2:2 ratio, and c) [Pd(**DD--N'**)(**AA'--N'**)]$_2$(BF$_4$)$_4$. In competition for Pd(II), there is liberation of some **DD--N'** and **AA'--N'** and formation of [Pd(**AD--N'**)(**DA'--N'**)]$_2$(BF$_4$)$_4$.

3.1.2. Quantitative NMR for -N- type complexes

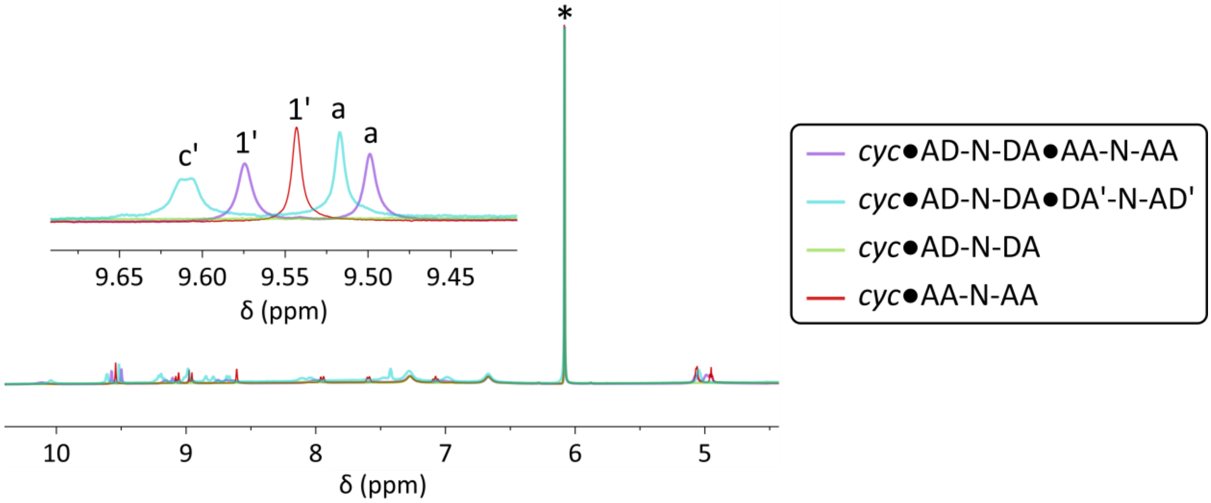

Figure S155 Superimposed partial ^{1}H NMR spectra (400 MHz, [D$_6$]DMSO, 298 K) of ***cyc*●AD-N-DA●AA-N-AA**, **cyc●AD-N-DA●DA'-N-AD'**, **cyc●AD-N-DA** and **cyc●AA-N-AA** relative to the aromatic signal of the reference standard trimethoxybenzene (*).

Table S1 Relative proportion of ***cyc*●AD-N-DA●DA'-N-AD'**, **cyc●AD-N-DA●AA-N-AA**, **cyc●AA-N-AA** and **cyc●AD-N-DA** produced from the respective groups of ligands and Pd(II), as determined via ^{1}H QNMR displayed in Figure S130.

Ligands	Proportion of Macrocycle (%)
AD-N-DA + DA-N-AD	22
AD-N-DA + AA-N-AA	19
AA-N-AA	17
AD-N-DA	0

4. Combinatorial Studies

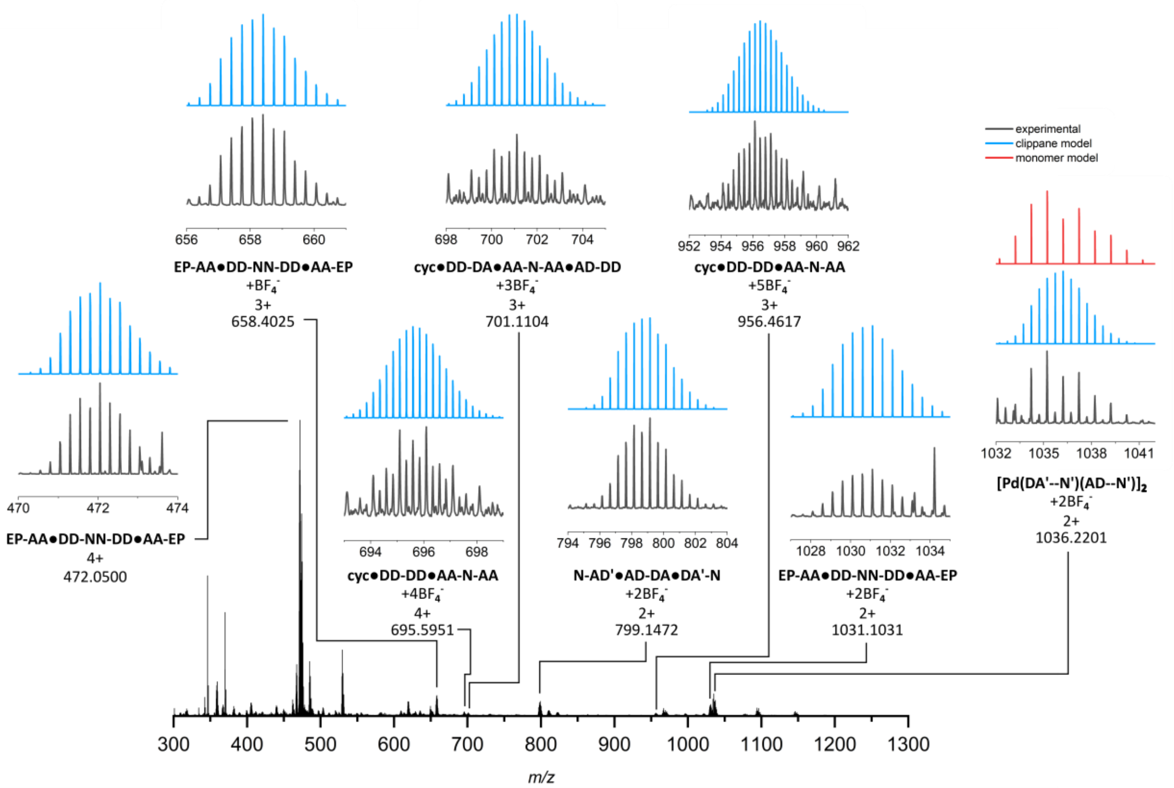

Figure S156 HR-ESI MS (DMSO/acetonitrile) of the combination of **EP-AA●DD-NN-DD●AA-EP, cyc●DD-DD●AA-N-AA, cyc●DD-DA●AA-N-AA●AD-DD, N-AD'●AD-DA●DA'-N** and **[Pd(DA'--N')(AD--N')]$_2$**.

5. Calculations

To investigate the differences in energy which arise from steric and electronic contributions between homoleptic, heteroleptic structures and their various isomers, idealised representations of each were first constructed in *Spartan24*,[15] omitting counter ions and solubilising chains for simplicity. These structures were then optimised using the quantum mechanical extended tight binding method GFN2-xTB 6.4.1,[16] as implemented in the *xtb* code. Optimisations were performed using GFN 2, a "normal" optimization [normal: Econv (energy convergence) = 5×10^{-6} Eh; Gconv (gradient convergence) = 5×10^{-3} Eh·α^{-1}; accuracy (for integral cutoffs and SCF criteria) = 1.00] and the solvent DMSO was represented with an implicit solvation model (ALPB, as implemented in the xTB code).[17] Lebedev grid level used was "normal" with 230 grid points (as implemented in the xTB code).[18]

6. Crystallography

6.1. Experimental data

Single crystal data were collected using mirror-monochromated Cu Kα radiation on an Agilent SuperNova diffractometer at 150 K. Raw frame data (including data reduction, interframe scaling, unit cell refinement and absorption corrections) were processed using CrysAlis Pro.[19] All structures were solved by direct methods using SHELXT2014[20] and refined against all F2 data using SHELXL2014[20b] and the OLEX2[21] interface. The data are summarised in Table S2.

Table S2 Summary of crystallographic data.

	**$[[Pd(DA'\text{--}N')(AD\text{--}N')]_2]^{4+}$**	**EP-AA'●DD-NN-DD●AA'-EP**
**CCDC No.**	**2522355**	**2522356**
Formula	$C_{64}H_{69}N_8O_7PdS_7$	$C_{106}H_{88}F_{13.5}N_{16}O_9Pd_2S_9$
M	1393.09	2487.76
T (K)	150	150
Crystal system	tetragonal	triclinic
Space group	$I\bar{4}2d$	$P\bar{1}$
a (Å)	19.9140(5)	10.3474(6)
b (Å)	19.9140(5)	21.4832(17)
c (Å)	36.7007(15)	25.9846(12)
α (°)	90	108.312(5)
β (°)	90	94.174(4)
γ (°)	90	95.090(6)
V (Å^3)	14554.4(9)	5431.1(6)
Z [*Z′*]	8(0.5)	2(1)
Crystal description	orange block	orange plate
Crystal size (mm^3)	0.5 x 0.4 x 0.4	0.1 x 0.07 x 0.03
μ (mm^{-1})	4.359	5.043
$2\vartheta_{full}$, $2\vartheta_{max}$ (°)	134.00, 117.31	79.90, 77.10
$N_{measured\ refl}$	23485	19734
$N_{independent\ refl}$ [R_{int}]	4051 [0.0375]	4357 [0.0768]
$N_{observed\ refl}$ [$I > 2\sigma(I)$]	4334	3406
$N_{parameters}$	301	1081
$N_{restraints}$	559	1203
R [$I > 2\sigma(I)$]	0.1119	0.1244
wR [all data]	0.3386	0.3386
GOF	1.133	1.322
$\Delta\rho_{max}$, $\Delta\rho_{min}$ (e Å^{-3})	1.984, -0.566	1.100, -0.587
Solvent mask	8093 A^3, 2336 e$^-$	2352 A^3, 774 e$^-$

6.1. ORTEP diagrams

Structure of [[Pd(**DA'--N'**)(**AD--N'**)]$_2$]$^{4+}$: Despite a strongly diffracting crystal, the data quality appears poor due to twinning or the apparent symmetry of the structure. The square-planar Pd complex is disordered over two positions, that coincide with improper rotation axis. Reducing the symmetry of the data did not improve the quality of the solution, so the pyridyl-triazole and pyridazine-pyrazole fragments are modelled in the same position as different parts, that do not correlate correctly across the symmetry element. As a result of this disorder, heavy geometric and ADP restraints were required for these and the anthracene fragments. Disordered solvent and counterions were also present in the structure and were accounted for by use of a solvent mask. It is important to note that due to the poor data quality we cannot determine whether the homoleptic or heteroleptic complexes are preferred in the solid state, but the complexes do interlock to form a [2]clippane structure as expected.

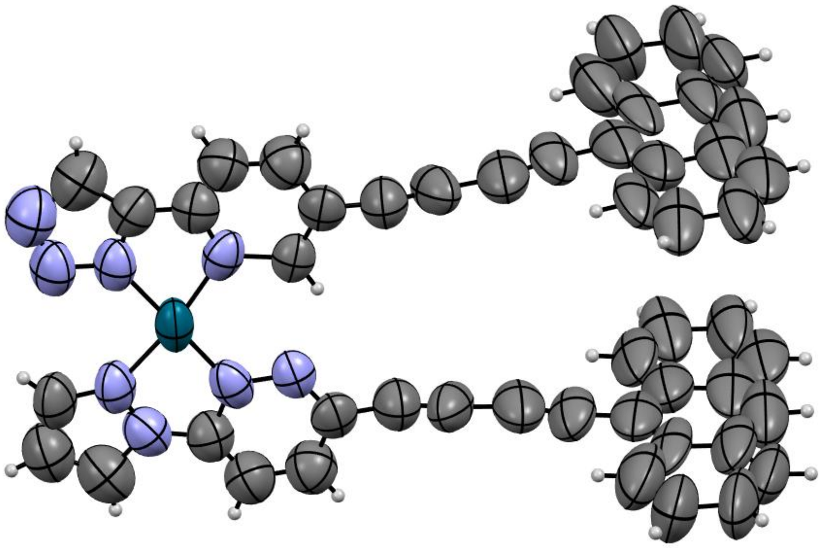

Figure S157 ORTEP diagram of the molecular unit of [[Pd(**DA'--N'**)(**AD--N'**)]$_2$]$^{4+}$. The ligands are disordered about an improper rotation axis at the Pd(II) atom; this representation shows the expected heteroleptic complex. The second component of disorder is omitted for clarity. Ellipsoids are shown to the 50% probability level.

Structure of **EP-AA'•DD-NN-DD•AA'-EP**: The crystals of this complex were very small and diffracted poorly, so data was collected to 1.2 Å. Despite this, the resulting structure is unambiguous in confirming the conformation of the foldamer. The position and ADPs of many atoms required heavy restraints, particularly those of the perfluoronapthyl groups that are likely disordered across two positions due to rotation about the alkynyl groups. Disordered solvent and counterions were also present in the structure and were accounted for by use of a solvent mask.

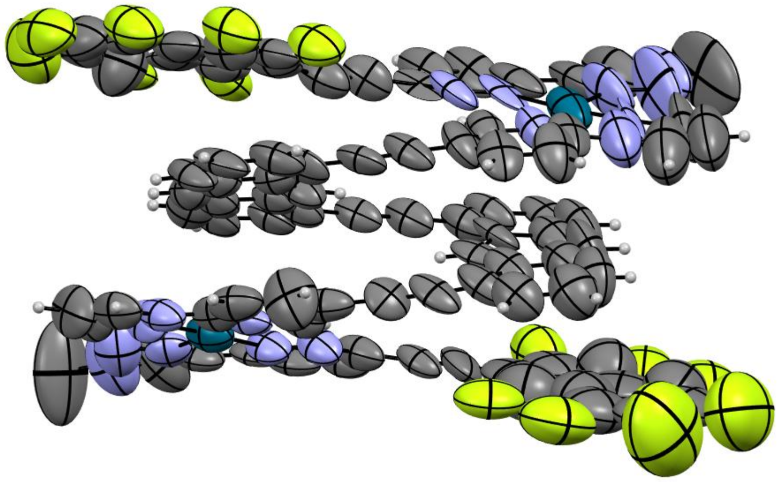

Figure S158 ORTEP diagram of the ASU of **EP-AA'•DD-NN-DD•AA'-EP**. Ellipsoids are shown to the 50% probability level.

7. References

[1] Y. Kawada, T. Kodama, K. Miyashita, T. Imanishi, S. Obika, *Heterocycles* **2010**, *80*, 1249-1265.

[2] R. Hooper, L. J. Lyons, M. K. Mapes, D. Schumacher, D. A. Moline, R. West, *Macromolecules* **2001**, *34*, 931-936.

[3] C. Huang, C. G. Zhen, S. P. Su, K. P. Loh, Z. K. Chen, *Org. Lett.* **2005**, *7*, 391-394.

[4] P. I. Dron, K. Zhao, J. Kaleta, Y. Shen, J. Wen, R. K. Shoemaker, C. T. Rogers, J. Michl, *Adv. Funct. Mater.* **2016**, *26*, 5718-5732.

[5] Q. Xiao, R. T. Ranasinghe, A. M. P. Tang, T. Brown, *Tetrahedron* **2007**, *63*, 3483-3490.

[6] J. L. Algar, J. N. Smith, D. Preston, *Inorg. Chem.* **2025**, *64*, 22545-22555.

[7] J. N. Smith, Y. Yau, N. R. Lawson, R. J. Goodwin, D. Preston, *Chem. Eur. J.* **2025**, e02411.

[8] K. Stott, J. Stonehouse, J. Keeler, T.-L. Hwang, A. J. Shaka, *J. Am. Chem. Soc.* **1995**, *117*, 4199.

[9] a) J. Jeener, B. H. Meier, P. Bachmann, R. R. Ernst, *J. Chem. Phys.* **1979**, *71*, 4546; b) R. Wagner, S. Berger, *J. Magn. Reson. A* **1996**, *123*, 119-121.

[10] a) A. Jerschow, N. Müller, *J. Magn. Reson., Ser. A* **1996**, *123*, 222-225; b) A. Jerschow, N. Müller, *J. Magn. Reson.* **1997**, *125*, 372-375.

[11] J. K. Kendall, H. Shechter, *J. Org. Chem.* **2001**, *66*, 6643-6649.

[12] J. M. Lovell, J. A. Joule, *Synth. Commun.* **1997**, *27*, 1209-1215.

[13] H. Dang, M. A. Garcia-Garibay, *J. Am. Chem. Soc.* **2001**, *123*, 355-356.

[14] K. Takahashi, S. Ito, K. Nakamoto, Y. Ito, Y. Ueno, *J. Org. Chem.* **2015**, *80*, 8561-8570.

[15] Wavefunction Inc., **2025**.

[16] C. Bannwarth, S. Ehlert, S. Grimme, *J. Chem. Theory Comput.* **2019**, *15*, 1652-1671.

[17] G. Sigalov, A. Fenley, A. Onufriev, *J. Chem. Phys.* **2006**, *124*, 124902.

[18] V. I. Lebedev, *Sib. Math. J.* **1977**, *18*, 99-107.

[19] Agilent Technologies, Yarnton, Oxfordshire, UK, **2019**.

[20] a) G. M. Sheldrick, *Acta Crystallogr. A* **2008**, *64*, 112-122; b) G. M. Sheldrick, *Acta Crystallogr. A, Found. Adv.* **2015**, *71*, 3-8.

[21] O. V. Dolomanov, L. J. Bourhis, R. J. Gildea, J. A. K. Howard, H. Puschmann, *J. Appl. Cryst.* **2009**, *42*, 339-341.
